# *Bacillus* G7 improves adaptation to salt stress in *Olea europaea* L. plantlets, enhancing water use efficiency and preventing oxidative stress

**DOI:** 10.1038/s41598-023-49533-z

**Published:** 2023-12-15

**Authors:** Estrella Galicia-Campos, Ana García-Villaraco, Ma. Belén Montero-Palmero, F. Javier Gutiérrez-Mañero, Beatriz Ramos-Solano

**Affiliations:** Facultad de Farmacia, Universidad San Pablo-CEU Universities, Ctra. Boadilla del Monte km 5.3, Boadilla del Monte, 28668 Madrid, Spain

**Keywords:** Biotechnology, Microbiology, Plant sciences

## Abstract

In addition to genetic adaptative mechanisms, plants retrieve additional help from the surrounding microbiome, especially beneficial bacterial strains (PGPB) that contribute to plant fitness by modulating plant physiology to fine-tune adaptation to environmental changes. The aim of this study was to determine the mechanisms by which the PGPB *Bacillus* G7 stimulates the adaptive mechanisms of *Olea europaea* plantlets to high-salinity conditions, exploring changes at the physiological, metabolic and gene expression levels. On the one hand, G7 prevented photosynthetic imbalance under saline stress, increasing the maximum photosynthetic efficiency of photosystem II (Fv/Fm) and energy dissipation (NPQ) and protecting against photooxidative stress. On the other hand, despite the decrease in effective PSII quantum yield (ΦPSII), net carbon fixation was significantly improved, resulting in significant increases in osmolytes and antioxidants, suggesting an improvement in the use of absorbed energy. Water use efficiency (WUE) was significantly improved. Strong genetic reprogramming was evidenced by the transcriptome that revealed involvement of the ABA-mediated pathway based on upregulation of ABA synthesis- and ABA-sensing-related genes together with a strong downregulation of the PLC2 phosphatase family, repressors of ABA-response elements and upregulation of ion homeostasis-related genes. The ion homeostasis response was activated faster in G7-treated plants, as suggested by qPCR data. All these results reveal the multitargeted improvement of plant metabolism under salt stress by *Bacillus* G7, which allows growth under water limitation conditions, an excellent trait to develop biofertilizers for agriculture under harsh conditions supporting the use of biofertilizers among the new farming practices to meet the increasing demand for food.

## Introduction

Soil salinity is one of the major abiotic stresses adversely affecting crop growth and yield, not only because of the high concentration of ions that limit plant water absorption but also because soil structure is negatively affected^1^. Crop yield is compromised from 4 dS/m electric conductivity (ECe). The latest studies report that approximately 13% of the total land as salinity-affected soils, increasing up to 23% during the XXI century. Furthermore, the predicted population increase by 2050 will cause a similar increase in food demand, obliging farmers to modify their farming strategies to remain productive within a water-limiting context due to limited water availability and soil salinity^2^.

Among these new farming strategies, plants have to be considered active players because they are naturally endowed with adaptive mechanisms to overcome changing environmental conditions. As plants cannot escape from adverse conditions, they need to deal with them on site by adjusting growth and activating adaptive/defense responses to biotic and abiotic stress^3^.

Water stress due either to lack of water (drought) or high salt concentrations (salinity)^4,5^ is the most limiting factor for plant growth, compromising crop yields^6^. Adaptation to water stress involves many events that can be organized into perception, upstream and downstream signaling, functional gene expression and metabolic adjustments, involving a marked reprogramming of plant metabolism^7^.

Under drought/salinity conditions, plants sense water stress when the water potential drops and start urgent modifications at the physiological level consisting of stomatal closure, leading to photosynthesis arrest and a concomitant loss of primary production^7^. Upon drought sensing, plants produce signal molecules and activate reactive oxygen species metabolism (ROS), which refers to generation and scavenging processes^8,9^. Indirectly, drought stress signals induce an expression of regulatory and functional downstream genes is triggered, leading to osmolyte and antioxidant synthesis, further enabling plants to successfully survive in the arid environment. In the case of salinity, the plant response follows a similar pattern, although a subset of genes related to ion balance are activated^10,11^.

Among the signaling molecules, ABA is involved in regulating plant responses to various external stresses (drought, high salt, low temperature, other), increasing its concentration upon sensing^12^. Under nonstressing conditions, the PP2C phosphatase family is a natural repressor of the SnRK2 kinase family, and the ABA response is blocked. Under drought and salt stress conditions, de novo synthesized ABA finds PYR/PYL/RCAR receptors, forming a complex that binds PP2C phosphatases, releasing SnrK2 kinases that immediately activate stomatal closure and downstream signal transduction pathways, leading to stress-counteracting changes^13^. Among the downstream transcription factors are ABA response element (ABRE)-binding proteins (AREB)/ABRE-binding factors (ABF)^14^. This will, in turn, induce functional stress response genes, enhancing drought tolerance. Therefore, the ABA response is based on increasing its synthesis and/or preventing degradation, in addition to sensitivity control through PYR/PYL/RCAR receptors. Nevertheless, there is also an ABA-independent pathway controlling the expression of some other drought-related genes to ensure plant responses to drought^15^. In addition, extensive cross-talk networks exist between ROS and hormonal signaling pathways to coordinate plant development and stress responses^16–19^. Moreover, ABA plays a pivotal role in connecting aboveground and belowground plant responses^20^.

Irrespective of the plant’s genetic endowment to overcome drought stress and survive, they increase their options by engaging rhizosphere microorganisms that can further enhance their metabolism for survival ^21,22^. Specific plant growth-promoting rhizobacteria (PGPR) are able to bind plant receptors, triggering a ROS-mediated systemic response in the aboveground parts of the plant involving modification of gene expression that results in an increased metabolic adaptation that best serves the plant^23,24^. This ROS burst has been described for biotic^25–27^ and abiotic stress^24,26^; therefore, the ROS burst is common to the perception of any stimuli by plants. It becomes evident that plant metabolism can be modulated through the microbiome, boosting plant innate responses to stress, that is, enhancing adaptive metabolism^28^.

A relevant feature of PGPR effects is that after several doses of PGPR, only slight metabolic/genetic changes will occur. Protection will be evidenced only upon stress challenge, with a faster and more intense adaptive response than that in the non-PGPR-treated plants; the prechallenge physiological status is known as priming. Therefore, treating plants with PGPR prior to stress is a wonderful tool to study the integrated response of plants to stress^29,30^.

Based on this potential, the aim of this study was to determine the mechanisms by which the PGPR *Bacillus* G7 improves the adaptation of *Olea europaea* var Arbequina plantlets to saline stress. To achieve this goal, olive plantlets grown in open air for 12 months with monthly inoculations will be studied by evaluating the following markers: (1) photosynthesis as a physiological marker; (2) photosynthetic pigments, antioxidant enzymes (SOD, APX), antioxidant molecules (phenols and flavonols), and osmolytes (proline, soluble sugars) as metabolic markers; and (3) genetic reprogramming by transcriptome and qPCR analysis of specific markers of salinity-related stress and pathogenesis-related proteins (PR) related to abiotic stress.

## Results

Photosynthetic parameters are shown in Table 1. G7-inoculated plants showed significant increases in maximum PSII efficiency (Fv/Fm) and in nonphotochemical quenching (NPQ) of 3.8% and 528.57%, respectively, while the minimum fluorescence (F_0_) was not affected, and the effective PSII yield (ϕPSII) significantly decreased by 18.18%.

**Table 1:** Effects of G7 on photosynthetic parameters related to photosystems and light reactions in inoculated and noninoculated controls and relative change (%) of inoculated vs. control.

Parameters	Control	G7	% G7 vs Control
F_0_	219.33 ± 6.4	209.00 ± 6.48	− 4.71%
Fv/Fm	0.79 ± 0.01	0.82 ± 0.01	3.80%*
ϕPSII	0.77 ± 0.01	0.63 ± 0.02	− 18.18%*
NPQ	0.14 ± 0.01	0.88 ± 0.28	528.57%*

F0, minimal fluorescence after 20 min of dark adaptation; Fv/Fm, maximal PSII quantum yield; ΦPSII, effective PSII quantum yield; NPQ. Non-photochemical quenching coefficient. For each treatment and parameter, the average value ± standard error value (n = 6) is presented. Asterisks (*) represent significant differences between treatment and the control according to Student’s t-test (p < 0.05).

Regarding carbon fixation (Table 2), control plants achieved 2.53 μmol CO_2_/m^2^s, while G7 inoculated plants showed significantly higher values (70%). However, the transpiration rate was similar in both groups (0.59 mmol H_2_O/m^2^s), resulting in a higher WUE in G7-treated plants (60%).

**Table 2:** Effects of G7 on photosynthetic parameters related to C fixation measured in olive tree plants treated with G7 and non-inoculated controls and relative change (%) of inoculated vs. control.

Parameters	Control	G7	% G7 vs Control
Pn	2.53 ± 0.09	4.35 ± 0.47	72%*
E	0.59 ± 0.04	0.63 ± 0.01	5%
WUE	4.26	6.95	63%*

Pn, net photosynthesis, measured as the CO_2_ fixed by the leaves (μmol CO_2_/m^2^ s). E, Transpiration rate, measured as the amount of water released by transpiration (mmol H_2_O/m^2^ s), and WUE (μmol CO_2_/m s/mmol H_2_O/m s), water use efficiency calculated as Pn divided by transpiration rate. Average values of the replicates with standard error bars are represented (n = 6). Asterisks (*) represent significant differences from the control according to Student’s t-test (p < 0.05).

**Figure 1:**
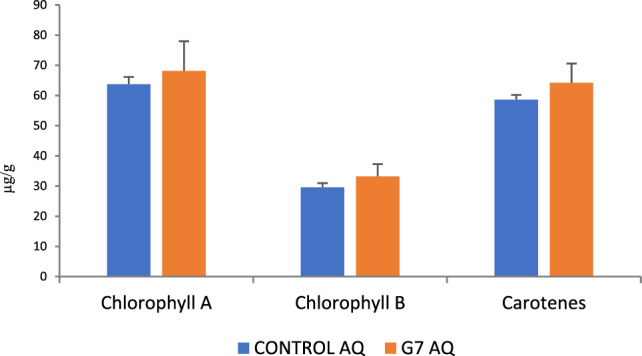
Concentration of photosynthetic pigments (μg/g FW). Chlorophyll a, chlorophyll b, and carotenoids measured in olive tree leaves treated with G7 (orange bars) and non-inoculated controls (blue bars). For each treatment and parameter, the average value ± SE value is presented (*n* = 6). Non-significant differences were found according to Student’s t-test (*p* < 0.05).

Regarding photosynthetic pigments, no significant changes were detected under G7 d (Fig. 1).

The osmolyte concentration is shown in Table 3. G7-inoculated plants showed a significant increase in proline (77.42%), while the soluble sugar (42.3%) concentration was not affected.

**Table 3:** Concentration of proline (nmol/g FW) and soluble sugars (mg/g FW), measured in olive tree leaves treated with G7 and non-inoculated controls, and relative change (%) of inoculated vs. control.

Parameters	Control AQ	G7 AQ	% G7 vs Control
Proline (nmol/g FW)	0.31 ± 0.04	0.55 ± 0.04	77.42%*
Soluble sugars (mg/g FW)	3.05 ± 0.51	4.34 ± 0.76	42.30%

For each treatment and parameter, the average value ± SE value is presented (n = 6). Asterisks (*) represent significant differences from the control according to Student’s t-test (p < 0.05).

**Figure 2:**
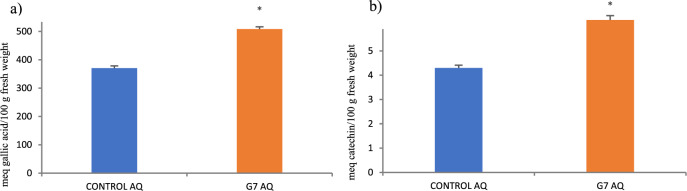
Concentration of (**a**) total phenols (meq gallic acid/100 g fresh weight) and (**b**) flavonol (meq catechin/100 g fresh weight) in olive tree leaves treated with G7 and non-inoculated controls. For each treatment, the average value ± SE value is presented (*n* = 6). Asterisks (*) represent significant differences from the control according to Student’s t-test (*p* < 0.05).

The total phenol concentration (Fig. 2a) and total flavonol content (45.81%) were significantly increased by G7 (37.18%) (Fig. 2b). Antioxidant enzymes (SOD and APX) were not affected by G7 (Table S1).

The expression of genes involved in ion homeostasis (antiport Na^+^/H^+^ SOS1 and symport Na^+^/H^+^ NHX), ABA receptor (PYL-8) and pathogenesis-related proteins (PR5 (Thaumatin-like protein) and PR10)) was lower in G7-treated plants than in controls (Fig. S1), with SOS1 being most similar to controls, while the expression of all other genes was decreased by at least 50%.

**Figure 3:**
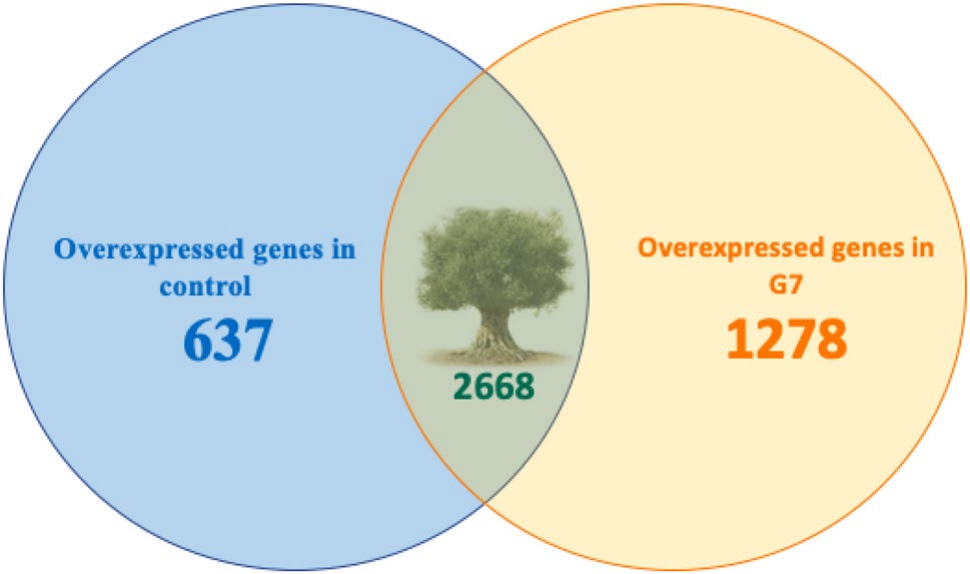
Venn diagram of unaffected and overexpressed genes specific to control or treatment.

The transcriptome revealed 1278 genes overexpressed in G7-treated plants, 637 in control plants and 2668 genes not affected by inoculation (Fig. 3 and Table S2).

Data from the transcriptome were manually curated, grouping genes according to functional classification, and metabolic analysis was carried out. The following groups were defined: (1) transcription factors, (2) hormone synthesis and response, (3) secondary metabolism-related genes, (4) ion homeostasis, and (5) others. Great activity is observed in cells of G7-treated plants, where strong genetic reprogramming is detected, based on the abundance of many transcription factors, as well as higher metabolic activity revealed by abundant transcripts related to cell vesicular trafficking and signaling (Ca^2+^-calmodulin, kinases) (Tables 4 and S2 “differential expression between control (AQRNA control) and G7 (AQRNA G7)-treated plants”.

**Table 4:** Cluster of upregulated and downregulated genes in samples inoculated with G7 compared to the control.

TRANSCRIPT FACTORS	BTB (cromatin binding)	Upregulated
	PRE6 (ABA-dependent response to salinity)	Upregulated
	SRM-1 (ABA biosynthesis)	Upregulated
SYNTHESIS AND SENSITIVITY TO HORMONES	Xantoin dehydrogenase (ABA biosynthesis)	Upregulated
	Phosphatases C2 (ABA degradation)	Downregulated
	Ethylene/auxin responsive genes AP2/ERF (stress response)	Downregulated
SECONDARY METABOLISM	8-hydroxygeraniol dehydrogenase-like (terpenes)	Upregulated
	1-deoxy-d-xylulose-5-phosphate synthase (terpenes)	Upregulated
	Aspartate aminotransferase (sikimates)	Upregulated
	glutamate/aspartate-prefenate aminotransferase 2C (sikimates)	Upregulated
WATER HOMEOSTASIS	V-ATPase	Upregulated
	ABC Transporters	Upregulated
	Na^+^ Transporters	Upregulated
	K^+^ Transporters	Upregulated
OTHER GENES OF INTEREST	Calmodulin-binding protein 60 (signaling)	Upregulated
	MLO-like protein (biotic stress protection)	Upregulated
	TL29 (ROS protection chloroplastic)	Upregulated

The genes overexpressed in G7-treated plants were 39 transcription factors, while 12 were downregulated. Among the overexpressed genes, the BTB type, which is involved in chromatin regulation and defense against biotic stress, was 5 times more highly expressed. Expression of the PRE6 gene is also outstanding, a gene that has been reported to be involved in hormone response regulation, especially in the ABA-dependent response to salt stress. Consistent with this ABA-dependent response is the overexpression of SRM-1, a transcription factor that activates ABA biosynthesis, and Xantoine dehydrogenase, a functional gene involved in ABA synthesis.

Regarding sensitivity to hormones, 38 genes were upregulated and 19 were downregulated in G7-treated plants. Among the downregulated genes, the family of phosphatases 2C-type are outstanding, as they are involved in repressing the ABA-mediated response. Among the overexpressed genes, AP2/ERF, a network of transcription factors involving ethylene and auxin stress responses to abiotic stress, was found. In summary, severe hormone-mediated reprogramming of gene expression due to ethylene, auxins and ABA is evidenced.

Among the secondary metabolism-related genes, terpene synthesis is activated mainly in the DOXP pathway, according to overexpression of 1-deoxy-d-xylulose-5-phosphate synthase and 8-hydroxygeraniol dehydrogenase-like; phenolic compound synthesis is also activated, especially the shikimate pathway based on upregulation of aspartate aminotransferase and glutamate/aspartate-prephenate aminotransferase.

Among the ion homeostasis-related genes, overexpression of proton pumps associated with vacuoles (V-ATPase) and ion exchangers in the plasma membrane and tonoplast, as ABC carriers, and sodium and potassium transporters.

## Discussion

The results presented here provide evidence about the ability of *Bacillus* G7 to improve the adaptation of olive plantlets to saline stress when delivered through the roots, therefore inducing a systemic adaptation, as modifications have been evaluated in leaves that were never in contact with G7. The strain modifies plant physiology by increasing the maximum photosynthetic potential (Fv/Fm), energy dissipation (NPQ), net carbon fixation (Pn) and water use efficiency (WUE). At the metabolic level, antioxidant molecules (phenols, flavonols and proline) increased, contributing to lower oxidative stress and maintaining plant water homeostasis. In addition to these targets, strong genetic reprogramming was detected, revealing activation of the ABA-dependent pathway to improve plant adaptation.

Drought or salinity are stress factors that limit plant growth and development, compromising photosynthesis, which is easily unbalanced upon stress^7^. Interestingly, G7 was able to counteract this imbalance, as indicated by photosynthetic data (Table 1); G7 enhanced the maximum photosynthetic yield (Fv/Fm), reaching healthy values, while controls remained below reference values, confirming that soil and water salinity in the marshes was toxic for olive plantlets. Interestingly, and contrary to our expectations, no significant increases in photosynthetic pigments were found. An increase in chlorophyll a would be expected to support a higher capability to absorb energy (Fv/Fm), while an increase in carotenes would support an increase in energy dissipation (NPQ)^31–33^; therefore, energy dissipation is not associated with an increase in carotenes, suggesting the involvement of alternative mechanisms to minimize photooxidative damage under stress. In support of this putative alternative mechanism, transcriptomic analysis revealed marked overexpression of thylakoid lumenal 29 kDa protein 2C chloroplastic (TL29), one of the most abundant proteins in the thylakoid lumen involved in ROS defense, suggesting complementary protection against ROS created upon photosynthesis. Furthermore, the energy effectively transformed into chemical energy (ϕPSII) is lower than in controls, suggesting a better use of this energy, as there is a real increase in the synthesis of organic molecules such as proline (Table 3)^34^ and phenols and flavonols (Fig. 2)^35^. The better use of the absorbed light energy is further improved by a significantly enhanced capacity to fix carbon (+ 72%) (Table 2).

In addition to preventing the production of ROS by energy dissipation, G7 preferentially enhances antioxidant molecule contents rather than ROS scavenging enzyme activity (Table S1) to remove ROS. In line with this statement, we speculate that the strategy of G7 could be described as preventing excess ROS synthesis by modulating photosynthesis and maintaining physiological levels of signaling H_2_O_2_ contributing to redox homeostasis and to activation of ROS responsive genes^7^.

The molecular mechanisms activated by G7 were mainly revealed by transcriptomic analysis, and the activation of the ABA-mediated pathway was unveiled. On the one hand, upregulation of transcription factors (PRE6, SRM-1) and functional genes (xanthoine dehydrogenase) involved in ABA biosynthesis was observed. On the other hand, not only is ABA synthesis increased, but its effects are also enhanced, as indicated by the downregulation of genes of the phosphatase PL2C family, which are responsible for impairing ABA-mediated responses^36,37^. In summary, G7 triggers the ABA-mediated pathway by activating its synthesis and downregulating repressor synthesis; this is consistent with the increase in water use efficiency. The relationship between environmental stress and Ca^2+^ signaling is also evidenced by transcriptomic data^36,37^; upregulation of the gene encoding calmodulin-binding protein 60, a protein involved in plant protection against abiotic stress, has been shown^38^. Interestingly, the expression of the MLO-like protein gene is also upregulated; as this protein is a Ca^2+^-calmodulin-regulated protein involved in pathogen defense^39^, it appears that this genetic reprogramming would also protect the plants against biotic stress, evidencing the efficiency of G7 in simultaneously activating defense pathways to overcome biotic and abiotic stress ^23^.

Interestingly, secondary metabolism-related genes involved in the terpene (DOXP) and shikimate pathways were also upregulated (Table 4). Within the DOXP pathway, 1-deoxy-D-xylulose-5-phosphate (DOXP) synthase and 8-hydroxygeraniol dehydrogenase-like were upregulated^40,41^. DOXP synthase is the first enzyme in the pathway leading to isoprene and is hence key for terpenoid biosynthesis; upregulation of this enzyme is consistent with the increase in ABA synthesis, as it provides precursors to enhance the ABA-mediated protection induced by G7. On the other hand, upregulation of hydroxygeraniol dehydrogenase leads to oleuropein and related iridoids, with antioxidant and osmotic roles in olive leaves^42,43^. Phenolic compound synthesis is also activated, especially the shikimate pathway based on upregulation of aspartate aminotransferase and glutamate/aspartate-prephenate aminotransferase, consistent with the reported flavonol increase (Fig. 2). In addition to their role as antioxidants and osmolytes, the accumulation of these secondary metabolites in leaves accounts for the enhanced nutritional and health-related value of the plant^30^.

Finally, specific mechanisms of adaptation to salt stress related to ion homeostasis were analyzed in the transcriptome (Table 4) and by qPCR (Fig. S1). The transcriptome revealed upregulation of ABC-type transporters, as well as those of Na^+^(NHX), K^+^ (HKT) and V-ATPases, all involved in ion homeostasis^11^. However, quantification by RT-qPCR revealed non-significant lower expression levels in G7 inoculated plants. This apparently inconsistent situation may be due to the dynamics of PGPR-induced responses^44^. The first stage of the response is known as *priming*, in which primed plants undergo some slight changes that are not evidenced until stress challenge, and a more intense and faster response to stress takes place (postchallenge primed state) ^45,46^. Consistent with the two stages, G7 primed plants would have responded faster to the summer stress period (higher temperature and salt concentration); therefore, the expression of specific genes will already be lower than naïve controls, still trying to overcome the situation^29^. Furthermore, a wave-like expression pattern has been described for plant adaptive responses, registering expression peaks and valleys to keep the adaptive response active^47^. The expression patterns of PR5 and PR10 follow the same rationale. These two PRs are involved in biotic and abiotic stress adaptation^48–50^, while PR10 is specifically involved in the control of secondary metabolism, flavonoid transport and ABA sensitivity, consistent with the proposed mechanism^12,51^. PR5 has been related to root colonization by PGPR, also playing a relevant role as an antioxidant and osmolyte^6^.

In summary, G7 prevents photooxidative imbalance, as shown by increases in the maximum photosynthetic potential (Fv/Fm), energy dissipation (NPQ), net carbon fixation (Pn) and water use efficiency (WUE). At the metabolic level, antioxidant molecules (phenols, flavonols and proline) rather than ROS scavenging enzymes contribute to lower oxidative stress. Strong genetic reprogramming was detected, revealing activation of the ABA-mediated pathway based on (1) upregulation of ABA biosynthesis-related genes and downregulation of repressing phosphatases of the PLC2 family and (2) upregulation of ion homeostasis-related genes to improve plant adaptation. As this strain triggers multiple mechanisms improving adaptation to abiotic stress in olive plantlets, this mechanism needs to be validated in other crop plants before it can be developed into marketable biofertilizers to improve crop yield under water-limited conditions. Furthermore, it supports its suitability to be included among the new farming practices to meet the increasing demand for food.

## Material and methods

### Beneficial strain and olive tree variety

The beneficial strain assayed in this study is a gram-positive sporulated bacilli isolated from the rhizosphere of *P. pinaster*^21^ that produces siderophores. The 16S rDNA sequence identifies it as *Bacillus simplex* (G7: OP324816).

*Olea europaea* L. plants were used in this study. *Olea europaea* var. Arbequina plants were purchased from a local grower devoted to reproducing certified olive plants for agriculture (Plantas Continental S.A., Cordoba, Spain). Voucher specimens are deposited at Banco de Germoplasma, Universidad de Cordoba, Campus Rabanales.

### Inocula preparation and delivery to plants

Bacterial strains were kept in broth containing 20% glycerol at − 80 °C. To prepare inocula, bacteria were streaked onto PCA plates, incubated at 28 °C for 24 h, and then grown in Luria Broth liquid medium for 24 h with shaking (at 1000 rpm) at 28 °C. The inoculum density was adjusted to 1 × 10^8^ cfu/mL, and 500 mL (cells and culture medium) was delivered to the roots of each plant every 15 days from October 2017 to October 2018.

### Experimental design

Six-month olive plantlets were transplanted into 5 L pots with soil from the Guadalquivir Marshes. Plants were arranged in lines on an experimental plot within the marshes (37°06′34.5′′ N, 6°20′22.7′′ W); pot position was changed every two weeks to avoid side effects. Plants were watered every two weeks with saline water available on site. The electrical conductivities of the water and soil were 8.20 dS/m and 6.07 dS/m, respectively.

Bacteria were root-inoculated by soil drenching every 15 days from October 2017 to October 2018, so plants received 500 mL of water every week, alternating inoculum and water. Six plants per treatment (G7) were inoculated, leaving a noninoculated control (six plants); each plant was a biological replicate. In October 2018, photosynthesis was measured (fluorescence and CO_2_ fixation), and samples were taken. All determinations were carried out in six biological replicates, except for gene expression analysis, for which leaves from two plants in each treatment were pooled, constituting a replicate. Samples were brought to the lab and powdered in liquid nitrogen; ground plant material was stored at − 80 °C until analysis. The metabolic markers analyzed were photosynthetic pigments (chlorophyll a, b and carotenoids), ROS scavenging enzymes (SOD and APX), antioxidant molecules (total phenols, total flavonols) and osmolytes (proline and soluble sugars). The expression of genes involved in ion homeostasis and abiotic stress responses was analyzed by RT-qPCR, and a transcriptomic analysis was carried out.

### Photosynthesis (chlorophyll fluorescence)

Photosynthetic efficiency was determined through the chlorophyll fluorescence emitted by photosystem II. Chlorophyll fluorescence was measured with a pulse amplitude modulated (PAM) fluorometer (Hansatech FM2, Hansatech, Inc., UK). After dark adaptation of leaves, the minimal fluorescence (Fo; dark-adapted minimum fluorescence) was measured with weak modulated irradiation (1 μmol/m^2^ s). Maximum fluorescence (Fm) was determined for the dark-adapted state by applying a 700 ms saturating flash (9000 μmol/m^2^ s). The variable fluorescence (Fv) was calculated as the difference between the maximum fluorescence (Fm) and the minimum fluorescence (Fo). The maximum photosynthetic efficiency of photosystem II (maximal PSII quantum yield) was calculated as Fv/Fm. Immediately, the leaf was continuously irradiated with red-blue actinic beams (80 μmol/m^2^ s) and equilibrated for 15 s to record Fs (steady-state fluorescence signal). Following this, another saturation flash (9000 μmol μmol/m^2^ s) was applied, and then Fm′ (maximum fluorescence under light-adapted conditions) was determined. Other fluorescent parameters were calculated as follows: the effective PSII quantum yield ΦPSII = (Fm′−Fs)/Fm′^52^ and the nonphotochemical quenching coefficient NPQ = (Fm−Fm′)/Fm′. All measurements were carried out in the 6 plants of each treatment.

### Photosynthesis (CO_2_ fixation)

The leaf photosynthetic rate, Pn (μmol CO_2_/m^2^ s) and transpiration rate, E (mmol H_2_O/m^2^ s), were measured in fully expanded leaves (3rd leaf from apex) with a portable photosynthetic open system (CI-340, CID, Camas, WA, USA)^53^.

Water use efficiency, WUE (μmol CO_2_/m s/mmol H_2_O/m s), was calculated as net photosynthesis (Pn) divided by transpiration (E) as an indicator of stomatal efficiency to maximize photosynthesis, minimizing water loss due to transpiration.

All measurements were carried out in the 6 plants of each treatment.

### Photosynthetic pigments: chlorophylls and carotenoids

Extraction was performed according to^54^ on 6 biological replicates. One hundred milligrams of leaves powdered in liquid nitrogen was dissolved in 1 mL of 80% (v/v) acetone, incubated overnight at 4 °C and then centrifuged for 5 min at 10,000 rpm in a Hermle Z233 M-2 centrifuge. One milliliter of 80% acetone was added to the supernatant and mixed with a vortex. Immediately afterwards, absorbance at 647, 663, and 470 nm was measured on a Biomate 5 spectrophotometer to calculate chlorophyll a, chlorophyll b, and carotenoids (xanthophylls and carotenes) using the formulas indicated below^54,55^.

$$ {\mathbf{Chl}} {\mathbf{a}} \left( {{\mathbf{\mu g}}/{\mathbf{g}} {\mathbf{FW}}} \right) = \left[ {\left( {12.25 \times {\mathbf{Abs}} 663} \right) - \left( {2.55 \times {\mathbf{Abs}} 647} \right)} \right] \times {\mathbf{V}}\left( {{\mathbf{mL}}} \right)/{\mathbf{weight}} \left( {\mathbf{g}} \right) $$

$$ {\mathbf{Chl}} {\mathbf{b}} \left( {{\mathbf{\mu g}}/{\mathbf{g}} {\mathbf{FW}}} \right) = \left[ {\left( {20.31 \times {\mathbf{Abs}} 647} \right) - \left( {4.91 \times {\mathbf{Abs}} 663} \right)} \right] \times {\mathbf{V}}\left( {{\mathbf{mL}}} \right)/{\mathbf{weight}} \left( {\mathbf{g}} \right) $$

$$ \begin{aligned} {\mathbf{Carotenoids}} \left( {{\mathbf{\mu g}}/{\mathbf{g}} {\mathbf{FW}}} \right) & = [\left( {1000 \times {\mathbf{Abs}} 470} \right) - \left( {1.82 \times {\mathbf{Chl}} {\mathbf{a}}} \right) \\ & \;\; - \left( {85.02 \times {\mathbf{Chl}} {\mathbf{b}}} \right))/198 ] \times {\mathbf{V}}\left( {{\mathbf{mL}}} \right)/{\mathbf{weight}} \left( {\mathbf{g}} \right) \\ \end{aligned} $$

Tubes were protected from light throughout the whole process.

### Total phenols and flavonols

To prepare the leaf extracts (n = 6), 0.25 g of leaves (powdered in liquid nitrogen) was mixed with 2.5 mL of 80% methanol, sonicated for 10 min and centrifuged for 5 min at 5000 rpm. Total phenols were quantified with Folin-Ciocalteu reagent (Sigma. Aldrich, St Louis, MO) following the protocol described by^56^ with slight modifications. Twenty microlitres of extract was mixed with 0.250 mL of Folin-Ciocalteu 2 N and 0.75 mL of 20% Na_2_CO_3_ solution. The reaction was allowed to proceed for 30 min, and the absorbance at 760 nm was measured. A calibration curve was made (r = 0.99) with gallic acid (Sigma-Aldrich, St Louis, MO, USA) as a standard. The results are obtained in mg of gallic acid equivalents per 100 g of leaves (fresh weight). Total flavonols were quantified as described by^57^ using catechin as a standard (Sigma-Aldrich, St Louis, MO, USA). One milliliter of the extracts was diluted with 4 mL of distilled water and mixed. Then, 0.3 mL of NaNO_2_ 5% was added, mixed and incubated for 5 min. Next, 0.3 mL of 10% AlCl_3_ was added and incubated for 5 min. Finally, 2 mL of 1 M NaOH was added, and distilled water was added to a final volume of 10 mL and mixed. Absorbance was measured at 510 nm. A calibration curve was made with catechin as the standard (r = 0.99). The results are obtained as mg of catechin equivalents per 100 g of leaves (fresh weight).

### Transcriptome analysis (RNA-seq)

Plant material from the 3 biological replicates was used for transcriptome analysis. The whole process was carried out by AllGenetics & Biology SL as follows.

#### RNA extraction

Following the manufacturer's instructions, total RNA was extracted and purified from each replicate using the Total RNA Isolation kit (NZYTech); a negative control was included to check contamination during the process. RNA was eluted in a 40 mL final volume. RNA integrity and quantity were confirmed on an Agilent 2100 analyzer using an Agilent RNA 6000 Nano kit.

#### Library preparation

Libraries for Illumina sequencing were prepared with the directional RNA NEBNext® Ultra™ II kit according to the manufacturer’s instructions. Briefly, samples were enriched in mRNA by selecting 3′ end-poly(A) tails with the magnetic isolation module NEBNext Poly(A). The mRNA was transformed to cDNA, and sequencing adaptors were added prior to sequencing. Samples were dual indexed for demultiplexation after sequencing. A Bioanalyzer Agilent 2100 was used to check the fragment size distribution and concentration (Agilent HS kit). Libraries were quantified with the assay kit Qubit dsDNA HS (Thermo Fisher Scientific) and grouped in equimolar amounts according to Qubit. These groups were sequenced on a NovaSeq PE150 flow cell to achieve a total of 60 gigabases.

#### Sequencing

The whole genome was sequenced on the Illumina NovaSeq PE150 platform, resulting in a net amount of paired-end readings between 54,375,466 and 70,710,620 per sample. The quality of fastq files was verified prior to processing with FastQC v0.11.5 software. Adaptors were removed with Trimmomatic v0.39; low-quality readings (HEADCROP:12 and TRAILING:25) and sequences below 40 base pairs (bp) (MINLEN:40) were also removed. A second quality check was run with FastQC to ensure that only high-quality readings were used for mapping. Filtered readings were aligned to the *Olea europaea* L. var. sylvestris genome (assemblage O_europaea_v1; GenBank: GCA_002742605.1) using STAR 2.7.8a. Secondary alignments were discarded, and only unique alignment readings were used for further analysis.

#### Quantification of gene expression and differential expression analysis

Readings were quantified using Htseq-counts v0.13.5. Counts were normalized by Trimmed Mean of M-values (TMM). Differential expression analysis was calculated with Bioconductor NOISeq and NOISeqBIO, which are especially suitable for analyzing data from biological replicates (q = 0.95).

### RNA extraction and RT-qPCR analysis

Samples stored in a − 80 °C freezer were ground to a fine powder with liquid nitrogen in a sterile mortar and pestle before RNA extraction. Total RNA was isolated from each replicate (n = 3) with a GeneJET Plant RNA Purification Mini Kit (Thermo Scientific) (DNase treatment included), and after confirmation of RNA integrity using NanodropTM, retrotranscription was carried out. All products from retrotranscription were pooled, and three analytical replicates were used for RT-qPCR analysis.

The iScript tm cDNA Synthesis Kit from Bio-Rad was used to carry out the retrotranscription. An Applied Biosystems GeneAmp PCR System 2700 was used for all retrotranscriptions. The samples were incubated for 5 min at 25 °C, 30 min at 42 °C, 5 min at 85 °C, and then held at 4 °C. Amplification was carried out using a MiniOpticon Real-Time PCR System (Bio-Rad): a melting curve was used to verify the results after 39 cycles of 15 s at 95 °C, 30 s at 55 °C, and 30 s at 72 °C. The cycle threshold (Ct) was utilized to describe the analyses' expression. For each gene, standard curves were produced, and the efficiency ranged from 80 to 120%. The primers used appear in Table S3 of the supplementary material, and the reference gene used was GADPH2. The primers for SOS1 (*OeSOS1*; XM_023036083.1), NHX (*OeNHX*; XM_022986695), PYL-8 (*OePYL-8*, XM_023040693.1), and PR5 (*OePR5*; XM_023041217.1) were designed in PRIMER3 based on the genomes of *Olea europaea* L. var sylvestris and *Olea europaea* L. var koronieki. Primers for PR10 (*OePR10*) were obtained from^58^. According to^59^, the results of gene expression were expressed as a differential expression relative to controls. The control expression was set to 1; therefore, only changes above or below 2 are considered.

### Statistics

To evaluate treatment effects, Student’s t-test (Statgraphics Centurion XVIII) was performed for each of the variables. Differences were significant at p < 0.05.

### International, national and/or institutional guidelines

Authors reporting experiments confirmed that the use of plants in the present study complies with international, national and/or institutional guidelines.

### Supplementary Information

Supplementary Information.

## Data Availability

The original contributions presented in the study are included in the article/supplementary material, and further inquiries can be directed to the corresponding author. Raw data from this study were deposited in the NCBI SRA (Sequence Read Archive) database under number PRJNA961039. The SRA records are accessible at the following link: https://www.ncbi.nlm.nih.gov/sra/PRJNA961039.
