# Supplementary material for: Bacillus G7 improves adaptation to salt stress in Olea europaea L. plantlets, enhancing water use efficiency and preventing oxidative stress
Source: Sci Rep. 2023 Dec 15;13:22507. doi: 10.1038/s41598-023-49533-z (PMC10728083; doi:10.1038/s41598-023-49533-z)
Supplement: Supplementary file 1 — Supplementary Information. [file 41598_2023_49533_MOESM1_ESM.zip › suplementary material/table S2.pdf]

AQRNA_Control_vs_AQRNA_G7

	GeneID	AQRNA___Control _mean	AQRNA__G7_m ean	theta	prob	log2FC	Biotype	Access_number	Product
1	gene-LOC11136526	376.366974833433	471.26825340433	-1.752078340	0.984973699	-0.32440845757	protein_codin	XM_022985702.1	4-coumarate--CoA ligase-like 5
2	gene-LOC11136528	3.17339399523333	0.8599173533333	0.7099566251	0.953129748	1.883756737162	protein_codin	XM_022985724.1	protein TIFY 6B-like%2C transcript variant X2
3	gene-LOC11136529	18.6532289106667	7.5706834381	1.0289320801	0.968697773	1.300929935972	protein_codin	XM_022985735.1	ras-related protein RABC1-like
4	gene-LOC11136529	2.77009559313333	0.5	0.8575891631	0.961149347	2.469935763046	protein_codin	XM_022985742.1	cinnamoyl-CoA reductase 1-like
5	gene-LOC11136531	7.72750065056667	20.741701569566	-1.427108550	0.997944966	-1.42446047651	protein_codin	XM_022985761.1	protein decapping 5-like
6	gene-LOC11136532	75.4466724947	63.0782415495	0.8531492292	0.960966100	0.258314831173	protein_codin	XM_022985769.1	patellin-3-like
7	gene-LOC11136533	14.8889314987333	5.2789507694666	0.8820310137	0.962674250	1.495917106033	protein_codin	XM_022985796.1	putative late blight resistance protein homolog R1A-10
8	gene-LOC11136533	6.65904686843333	0.8863065918666	1.2751667783	0.983847826	2.909437945902	protein_codin	XM_022985800.1	acyl-coenzyme A thioesterase 13
9	gene-LOC11136537	0.6358831613	2.7253931664666	-0.744393455	0.951819460	-2.09963075803	lncRNA	XR_002697426.1	uncharacterized LOC111365371
10	gene-LOC11136538	0.938432989266667	5.9458122176	-1.022159519	0.984515577	-2.66354826509	protein_codin	XM_022985882.1	putative protein tag-278%2C transcript variant X1
11	gene-LOC11136538	0.5	4.1118336471333	-0.965162924	0.955510225	-3.03978189845	protein_codin	XM_022985886.1	histone acetyltransferase HAC1-like%2C transcript variant X2
12	gene-LOC11136538	1.85599181786667	6.1585986341666	-0.774426788	0.954755558	-1.73041175809	protein_codin	XM_022985890.1	peptidyl-prolyl cis-trans isomerase Pin1-like
13	gene-LOC11136543	2.5612481251	6.5583444657333	-0.761019943	0.953613817	-1.35648465564	protein_codin	XM_022985993.1	ethylene-insensitive protein 2-like%2C transcript variant X3
14	gene-LOC11136544	2.7636413018	6.9317619515	-0.795314536	0.957172046	-1.32665173297	protein_codin	XM_022985984.1	DEAD-box ATP-dependent RNA helicase 16
15	gene-LOC11136544	3.11979256253333	0.5	1.1572135605	0.983113460	2.641450106346	protein_codin	XR_002697449.1	succinate dehydrogenase [ubiquinone] flavoprotein subunit 1%2C mitochondrial-like%2C transcript variant X1
16	gene-LOC11136545	143.6979244622	192.24089703896	-1.985773776	0.999999707	-0.41987606152	protein_codin	XR_002697454.1	NAC domain-containing protein 82-like%2C transcript variant X1
17	gene-LOC11136547	0.5	2.5127285353666	-0.801158618	0.957947684	-2.32925481692	protein_codin	XM_022986027.1	NAC domain-containing protein 104-like%2C transcript variant X2
18	gene-LOC11136548	14.2202172545333	6.2912288491333	0.7309572436	0.953321544	1.176529758864	protein_codin	XM_022986034.1	uncharacterized LOC111365480
19	gene-LOC11136553	3.15343919836667	0.6531665342333	0.8191240094	0.960204235	2.271403337091	protein_codin	XM_022986091.1	uncharacterized mitochondrial protein AtMg00860-like
20	gene-LOC11136559	0.629428869966667	8.4026608299	-1.105395350	0.974230184	-3.73873099423	protein_codin	XM_022986141.1	uncharacterized LOC111365593
21	gene-LOC11136560	2.55479383373333	0.6531665342333	0.6953689135	0.952227925	1.967684093826	protein_codin	XM_022986147.1	putative receptor protein kinase ZmPK1
22	gene-LOC11136560	1.96905705183333	0.5	0.6815674662	0.950366313	1.977504912765	protein_codin	XM_022986150.1	glycerol-3-phosphate 2-O-acyltransferase 6-like
23	gene-LOC11136562	7.51727800333333	2.1393110495666	0.8468850487	0.960749709	1.813064097055	protein_codin	XM_022986161.1	uncharacterized LOC111365622
24	gene-LOC11136565	0.798567038966667	5.1265900431	-0.981199623	0.967000374	-2.68251410391	protein_codin	XM_022986197.1	uncharacterized LOC111365657
25	gene-LOC11136566	6.09365651153333	0.9729997351	1.1233642856	0.978381570	2.646796862845	protein_codin	XM_022986205.1	transcription factor DIVARICATA-like
26	gene-LOC11136572	0.5	2.4055599655666	-0.761230258	0.953631963	-2.26637276323	protein_codin	XM_022986259.1	uncharacterized LOC111365722
27	gene-LOC11136574	1.96905705183333	5.4652870547333	-0.736712916	0.950632128	-1.47279236017	protein_codin	XM_022986281.1	7-deoxyloganetic acid glucosyltransferase-like
28	gene-LOC11136576	44.9121840273	21.732425257966	0.9215729814	0.969416437	1.047257697492	protein_codin	XM_022986302.1	heme-binding-like protein At3g10130%2C chloroplastic
29	gene-LOC11136587	0.5	3.5726161952333	-1.317115159	0.971158587	-2.83698093408	protein_codin	XM_022986379.1	insulin-degrading enzyme-like 1%2C peroxisomal
30	gene-LOC11136589	0.5	2.8325008435666	-0.841171671	0.957201585	-2.50207638570	lncRNA	XR_002697521.1	uncharacterized LOC111365894
31	gene-LOC11136589	8.08266836023333	1.1329467819	1.3658040239	0.956739695	2.834751558897	protein_codin	XM_022986447.1	zinc finger CCCH domain-containing protein 30-like%2C transcript variant X1
32	gene-LOC11136591	2.46109147383333	0.5	0.9626316258	0.976207875	2.299298280873	lncRNA	XR_002697522.1	uncharacterized LOC111365918
33	gene-LOC11136592	4.05463439093333	0.7331400576333	0.9065261498	0.966029053	2.467411092119	protein_codin	XM_022986482.1	OTU domain-containing protein At3g57810%2C transcript variant X1
34	gene-LOC11136594	12.5479514730333	5.8378987326666	0.7262870882	0.953311277	1.103930764533	protein_codin	XM_022986522.1	uncharacterized LOC111365947
35	gene-LOC11136595	0.5	3.5317871276333	-0.794464778	0.957058227	-2.82039838965	protein_codin	XM_022986528.1	villin-3-like%2C transcript variant X2
36	gene-LOC11136595	1.36395739586667	6.6910355734333	-1.122216820	0.975640554	-2.29443093295	protein_codin	XM_022986535.1	thioredoxin reductase NTRC-like
37	gene-LOC11136595	769.9935453672	606.732274836	1.0716612354	0.967390595	0.343786295272	protein_codin	XM_022986539.1	zeatin O-glucosyltransferase-like
38	gene-LOC11136597	10.2153928407333	4.4785924057333	0.7880607842	0.958091615	1.189627406690	protein_codin	XM_022986575.1	translation initiation factor IF3-4%2C chloroplastic-like%2C transcript variant X2
39	gene-LOC11136598	12.9929262762333	4.8792049378666	0.9923883361	0.973302188	1.413008406029	protein_codin	XM_022986581.1	UDP-glucuronate 4-epimerase 1-like
40	gene-LOC11136598	3.11979256253333	0.6531665342333	0.9422451419	0.974196602	2.255927326641	protein_codin	XM_022986582.1	tetraspanin-8-like
41	gene-LOC11136598	2.57415670776667	7.7041803537	-0.787638998	0.956173991	-1.58154159320	protein_codin	XM_022986593.1	protein SRC2 homolog
42	gene-LOC11136599	120.813540375167	99.467175018	0.9067147075	0.966066640	0.280489747994	protein_codin	XM_022986603.1	inositol transporter 4-like%2C transcript variant X2
43	gene-LOC11136599	122.224557389867	64.644009615466	1.3472145603	0.965181762	0.918945590226	protein_codin	XR_002697559.1	methyltransferase-like protein 13%2C transcript variant X3
44	gene-LOC11136600	3.9858531679	0.5	1.0423329263	0.967676383	2.994888564706	protein_codin	XM_022986615.1	pentatricopeptide repeat-containing protein At3g06430%2C chloroplastic
45	gene-LOC11136601	1.82273681016667	0.5	0.7409573471	0.953455353	1.866106261827	protein_codin	XM_022986627.1	cullin-1-like%2C transcript variant X2
46	gene-LOC11136602	2.08916849996667	0.5	0.7050067510	0.952935613	2.062928856341	protein_codin	XM_022986641.1	zinc finger CCCH domain-containing protein 34-like%2C transcript variant X1
47	gene-LOC11136602	6.89084837136667	15.1436016217	-0.744135504	0.951783207	-1.13595484676	protein_codin	XM_022986657.1	uncharacterized LOC111366029%2C transcript variant X1

48	gene-LOC11136603	5.4885568556	1.8730012409	0.7117231553	0.953178901	1.551075005863	protein_codin	XM_022986660.1	myosin-binding protein 7-like%2C transcript variant X2
49	gene-LOC11136605	2.64066672316667	0.5	0.7971634153	0.959049456	2.400902231389	protein_codin	XM_022986696.1	sodium/hydrogen exchanger 1-like%2C transcript variant X2
50	gene-LOC11136605	11.8117988883	3.2067850299	0.9146892103	0.967768553	1.881029148375	protein_codin	XM_022986700.1	probable serine/threonine-protein kinase At1g54610
51	gene-LOC11136609	6.239585125	1.9325602304666	0.7183554512	0.953283161	1.690936728226	protein_codin	XM_022986744.1	sucrose synthase-like
52	gene-LOC11136609	0.5	2.2396991068	-0.844441436	0.956384897	-2.16330492590	protein_codin	XM_022986750.1	proteasome subunit alpha type-6
53	gene-LOC11136610	11.1617680726667	4.4266197365333	0.7981343468	0.959137317	1.334288223564	protein_codin	XM_022986754.1	20 kDa chaperonin%2C chloroplastic
54	gene-LOC11136610	2.7775334355	0.5	1.1021262443	0.972581813	2.473804278649	protein_codin	XM_022986763.1	protein PLASTID MOVEMENT IMPAIRED 1-RELATED 1-like
55	gene-LOC11136611	1.95614846913333	0.5	0.6824614660	0.950524364	1.968015873129	protein_codin	XM_022986769.1	U-box domain-containing protein 34-like%2C transcript variant X1
56	gene-LOC11136611	6.40266063083333	1.2333348391333	1.1102023445	0.974791660	2.376107011376	protein_codin	XM_022986771.1	THO complex subunit 6
57	gene-LOC11136611	58.3878825296	34.604115201133	0.8372194575	0.960513519	0.754725375223	protein_codin	XM_022986786.1	1-aminocyclopropane-1-carboxylate oxidase 1-like
58	gene-LOC11136613	2.86379795306667	0.6531665342333	0.7731620567	0.956164673	2.132406931336	protein_codin	XM_022986815.1	protein IQ-DOMAIN 31-like
59	gene-LOC11136619	2.51469290656667	0.6531665342333	0.7585585326	0.954481629	1.944859449660	protein_codin	XM_022986901.1	probable WRKY transcription factor 40
60	gene-LOC11136621	17.3626274496667	9.5985237216666	0.6799408328	0.950081505	0.855100846594	lncRNA	XR_002697604.1	uncharacterized LOC111366215%2C transcript variant X2
61	gene-LOC11136621	4.31708329173333	1.339697601	0.7651239881	0.955167121	1.688149538074	protein_codin	XM_022986926.1	transcription factor ICE1-like
62	gene-LOC11136625	23.3694275207667	13.304575939766	0.9268990898	0.970737743	0.812699865257	protein_codin	XM_022986978.1	myb-related protein 2-like
63	gene-LOC11136626	120.759579610933	44.548983147633	4.1665650550	0.999999999	1.438673232638	protein_codin	XR_002697619.1	uncharacterized LOC111366269%2C transcript variant X7
64	gene-LOC11136627	21.2835460760667	10.397270469666	0.7478341119	0.953711905	1.033533703570	protein_codin	XM_022986997.1	B3 domain-containing protein At2g36080-like%2C transcript variant X2
65	gene-LOC11136627	28.2625385444333	69.766920592066	-2.510929490	0.999999999	-1.30365210211	protein_codin	XM_022986999.1	TLD domain-containing protein 1%2C transcript variant X1
66	gene-LOC11136627	0.5	4.6444532645333	-1.106969266	0.974298338	-3.21550877551	protein_codin	XM_022987006.1	40S ribosomal protein S17-like
67	gene-LOC11136628	6.12730314736667	1.7131150868	0.7164719748	0.953266021	1.838630155491	protein_codin	XM_022987013.1	2-Cys peroxiredoxin BAS1%2C chloroplastic-like
68	gene-LOC11136632	5.25192512013333	0.5	1.2888306605	0.981576327	3.392846347003	protein_codin	XM_022987067.1	methyl-CpG-binding domain-containing protein 2-like
69	gene-LOC11136634	5.555066871	0.7331400576333	1.3173670480	0.975298962	2.921643539227	protein_codin	XM_022987090.1	lycopene epsilon cyclase%2C chloroplastic
70	gene-LOC11136634	1.06786185923333	5.0118960455666	-1.066932603	0.978230144	-2.23063146210	protein_codin	XM_022987091.1	uncharacterized LOC111366341
71	gene-LOC11136634	3.35583237506667	0.9729997351	0.6936925278	0.952062146	1.786159337140	protein_codin	XM_022987096.1	probable serine/threonine protein kinase IREH1
72	gene-LOC11136637	3.39366209463333	0.6531665342333	0.7510365707	0.953894577	2.377320143848	protein_codin	XM_022987144.1	uncharacterized LOC111366373%2C transcript variant X1
73	gene-LOC11136642	0.5	3.2850860448666	-0.980155061	0.966274670	-2.71593115894	protein_codin	XM_022987185.1	protein ARABIDILLO 1-like
74	gene-LOC11136644	5.593792619	0.7067508191	1.1876766155	0.982463472	2.984553213968	protein_codin	XM_022987200.1	ethylene-responsive transcription factor ERF114-like
75	gene-LOC11136645	29.8588893661333	11.0912660715	1.1486918727	0.982715033	1.428736444868	protein_codin	XM_022987211.1	probable beta-1%2C4-xylosyltransferase IRX10L
76	gene-LOC11136660	2.0762599173	0.5	0.6996756982	0.952603165	2.053987059267	protein_codin	XM_022987330.1	pentatricopeptide repeat-containing protein At5g15280%2C mitochondrial
77	gene-LOC11136662	2.7174777114	0.7067508191	0.7178740956	0.953278766	1.942994647192	protein_codin	XM_022987339.1	phosphatidylinositol 3%2C4%2C5-trisphosphate 3-phosphatase and protein-tyrosine-phosphatase PTEN2B-like
78	gene-LOC11136662	5.58088403633333	1.605946517	0.8777107586	0.962319463	1.797069821897	protein_codin	XM_022987343.1	heat shock 70 kDa protein 15-like%2C transcript variant X1
79	gene-LOC11136663	2.4417285998	0.5	0.8238133555	0.960296384	2.287902852461	protein_codin	XM_022987345.1	uncharacterized LOC111366630
80	gene-LOC11136679	2.46109147383333	0.5	0.9626316258	0.976207875	2.299298280873	protein_codin	XM_022987489.1	sm-like protein LSM1B%2C transcript variant X1
81	gene-LOC11136683	21.9971362540667	12.157067543333	0.7809075653	0.957190199	0.855520443230	protein_codin	XM_022987525.1	zinc finger MYM-type protein 1-like
82	gene-LOC11136684	7.4724895921	2.1861148219	0.7340913070	0.953341146	1.773219804415	protein_codin	XM_022987539.1	metal-nicotianamine transporter YSL3-like
83	gene-LOC11136685	7.07427030216667	1.4995837551	1.1055872440	0.973509902	2.238019242111	protein_codin	XM_022987549.1	protein STRICTOSIDINE SYNTHASE-LIKE 4-like
84	gene-LOC11136686	1.95614846913333	0.5	0.6824614660	0.950524364	1.968015873129	protein_codin	XM_022987550.1	protein SEH1
85	gene-LOC11136687	5.43397187183333	0.5	1.2870420353	0.981905478	3.442007097677	protein_codin	XM_022987559.1	uncharacterized LOC111366872
86	gene-LOC11136689	12.0158161238667	3.0121175089666	1.0495691702	0.967296888	1.996084682046	protein_codin	XM_022987579.1	methionine aminopeptidase 1D%2C chloroplastic/mitochondrial-like
87	gene-LOC11136690	0.662683877666667	4.2394776434	-1.039461071	0.984008771	-2.67749379183	protein_codin	XM_022987595.1	uncharacterized LOC111366906%2C transcript variant X2
88	gene-LOC11136692	21.0617025027667	32.739810552133	-0.845518458	0.956087717	-0.63642391376	protein_codin	XM_022987605.1	AP2-like ethylene-responsive transcription factor At1g16060
89	gene-LOC11136692	93.9413683148	112.55666890303	-0.788860726	0.956327054	-0.26081902536	protein_codin	XM_022987611.1	probable receptor-like protein kinase At5g61350
90	gene-LOC11136694	1.96260276046667	0.5	0.7250116886	0.953309787	1.972768194454	protein_codin	XM_022987624.1	ABC transporter A family member 2-like
91	gene-LOC11136699	48.2205651553667	32.601296111233	0.7891955515	0.958224567	0.564719237730	lncRNA	XR_002697700.1	uncharacterized LOC111366993
92	gene-LOC11136700	0.5	4.1059807277666	-1.309287209	0.970749809	-3.03772685588	protein_codin	XM_022987683.1	formin-like protein 4
93	gene-LOC11136702	256.000166701367	175.3393535301	1.5036972146	0.969210291	0.545994915751	protein_codin	XM_022987699.1	leucine-rich repeat protein 2-like
94	gene-LOC11136703	15.2576872365667	6.4239199568666	0.8357301475	0.960485744	1.248010472873	lncRNA	XR_002697706.1	uncharacterized LOC111367034
95	gene-LOC11136704	2.03556706723333	0.5	0.7088504665	0.953093597	2.025430755730	protein_codin	XM_022987766.1	putative cytochrome c oxidase subunit 5b-like
96	gene-LOC11136704	62.1146866242	114.85019586553	-2.375207528	0.999999999	-0.88674698709	protein_codin	XM_022987731.1	chloride channel protein CLC-b-like

97	gene-LOC11136705	6.54311850403333	1.6126661369	0.8577661738	0.961156983	2.020530605276	protein_codin	XM_022987753.1	uncharacterized LOC111367051%2C transcript variant X2
98	gene-LOC11136708	4.06813489646667	0.7067508191	1.0603803337	0.967047295	2.525093963528	protein_codin	XM_022987794.1	serine/threonine-protein kinase D6PK%2C transcript variant X2
99	gene-LOC11136709	0.765312031266667	3.3854741021	-0.776110368	0.954912384	-2.14523789791	protein_codin	XM_022987805.1	protein PLASTID TRANSCRIPTIONALLY ACTIVE 10-like
100	gene-LOC11136710	1.96905705183333	0.5	0.6815674662	0.950366313	1.977504912765	protein_codin	XM_022987819.1	B3 domain-containing protein Os02g0598200-like
101	gene-LOC11136710	12.3600423529	4.9583726534	0.9169631136	0.968300571	1.317745078420	protein_codin	XM_022987822.1	callose synthase 10%2C transcript variant X1
102	gene-LOC11136712	6.05296366143333	0.5	1.5121390263	0.969203418	3.597641689949	protein_codin	XM_022987852.1	probable pectate lyase 18
103	gene-LOC11136713	2.5612481251	11.5151055034	-0.990454852	0.973058405	-2.16860870115	protein_codin	XM_022987859.1	uncharacterized LOC111367134
104	gene-LOC11136715	3.02648183073333	0.7067508191	0.7929120671	0.958639195	2.098368134753	protein_codin	XM_022987882.1	protein JINGUBANG-like
105	gene-LOC11136716	2.74786958863333	0.5	0.7977912973	0.959106193	2.458313536799	protein_codin	XM_022987901.1	vesicle transport v-SNARE 13-like
106	gene-LOC11136718	0.5	3.5316653422333	-0.794451279	0.957056426	-2.82034864084	protein_codin	XM_022987931.1	uncharacterized LOC111367183
107	gene-LOC11136718	0.9255244066	4.8587904040666	-0.830561758	0.959032374	-2.39225425918	protein_codin	XM_022987932.1	uncharacterized LOC111367186%2C transcript variant X2
108	gene-LOC11136722	40.3897958434333	29.778738119	1.0021265417	0.971845170	0.439708233055	protein_codin	XM_022987976.1	cytochrome P450 81E8-like
109	gene-LOC11136722	2.79689630953333	0.6531665342333	0.7816986035	0.957295145	2.098303989485	protein_codin	XM_022987984.1	probable glutamate carboxypeptidase AMP1%2C transcript variant X1
110	gene-LOC11136724	5.5421582883	0.9729997351	0.9733470323	0.975740410	2.509936598542	protein_codin	XM_022988008.1	calcium-transporting ATPase 2%2C plasma membrane-type-like
111	gene-LOC11136726	6.35649704046667	0.6531665342333	1.3581700674	0.960293464	3.282709159610	protein_codin	XM_022988033.1	basic leucine zipper 4-like
112	gene-LOC11136728	9.31868483746667	1.605946517	1.1924848982	0.982417740	2.536702511698	protein_codin	XM_022988062.1	60S ribosomal protein L28-2-like
113	gene-LOC11136728	3.34937808373333	8.3173966242333	-0.739641690	0.951118383	-1.31223879095	protein_codin	XM_022988066.1	RWD domain-containing protein 1%2C transcript variant X2
114	gene-LOC11136729	3.9858531679	0.9398908767333	0.7550435885	0.954180094	2.084323392946	protein_codin	XM_022988101.1	nucleolar and coiled-body phosphoprotein 1-like
115	gene-LOC11136731	0.5	4.3713629432333	-1.048545228	0.982428624	-3.12808316642	protein_codin	XM_022988127.1	pectinesterase 1-like
116	gene-LOC11136736	2.08916849996667	0.5	0.7050067510	0.952935613	2.062928856341	protein_codin	XM_022988181.1	U-box domain-containing protein 4
117	gene-LOC11136736	3.99517058973333	1.0801683049	0.7153459942	0.953249031	1.887000984688	protein_codin	XM_022988189.1	E3 ubiquitin-protein ligase SINA-like 7
118	gene-LOC11136736	0.629428869966667	2.6658950696	-0.753199172	0.952881292	-2.08250473939	protein_codin	XM_022988196.1	sugar transporter ERD6-like 16%2C transcript variant X1
119	gene-LOC11136738	0.5	4.1118336471333	-0.965162924	0.955510225	-3.03978189845	protein_codin	XM_022988228.1	uncharacterized LOC111367384%2C transcript variant X3
120	gene-LOC11136740	8.1084855256	22.8765886375	-1.110457415	0.974494641	-1.49636754990	protein_codin	XM_022988261.1	interferon-related developmental regulator 2-like%2C transcript variant X3
121	gene-LOC11136741	6.97997601936667	0.9729997351	1.0091967781	0.970867184	2.842710762529	protein_codin	XM_022988271.1	WD40 repeat-containing protein HOS15-like%2C transcript variant X2
122	gene-LOC11136741	9.1915271751	1.9801698106666	0.8096647991	0.959892867	2.214680430859	protein_codin	XM_022988272.1	putative receptor protein kinase ZmPK1
123	gene-LOC11136742	38.0268063962333	15.2212795069	1.1080970481	0.974201992	1.320927144789	protein_codin	XR_002697815.1	putative disease resistance protein At3g14460%2C transcript variant X8
124	gene-LOC11136743	7.03357745213333	1.7327238127333	0.8980622350	0.964557368	2.021216951841	protein_codin	XR_002697818.1	putative disease resistance RPP13-like protein 1%2C transcript variant X9
125	gene-LOC11136748	5.1711313429	1.55316804	0.6997020794	0.952604958	1.735266022770	protein_codin	XM_022988390.1	uncharacterized LOC111367484
126	gene-LOC11136748	29.6594145458	17.3305831442	0.8472233587	0.960760521	0.775169920862	protein_codin	XM_022988391.1	DELLA protein GAI-like
127	gene-LOC11136748	1.08820828426667	4.7243658952	-0.814136522	0.959294129	-2.11816598723	protein_codin	XM_022988392.1	probable rhamnogalacturonate lyase B
128	gene-LOC11136750	3.1598934897	8.4781494789	-0.793991017	0.956995158	-1.42387347247	protein_codin	XM_022988412.1	UDP-glycosyltransferase 91A1-like
129	gene-LOC11136751	72.6320210818	37.083172641133	1.2074670808	0.982856984	0.969841046868	protein_codin	XM_022988431.1	mechanosensitive ion channel protein 2%2C chloroplastic-like%2C transcript variant X3
130	gene-LOC11136752	5.27833420836667	1.55316804	0.8684813144	0.961705683	1.764868774799	protein_codin	XM_022988449.1	ethylene-responsive transcription factor RAP2-13-like
131	gene-LOC11136754	140.306925203267	77.314255345	2.6345734032	1	0.859779868017	protein_codin	XM_022988478.1	uncharacterized LOC111367540
132	gene-LOC11136757	1.22409144556667	4.212282597	-0.829587807	0.959127259	-1.78289088992	protein_codin	XM_022988527.1	probable protein phosphatase 2C 27
133	gene-LOC11136760	4.33742971676667	1.1329467819	0.8111660571	0.959956010	1.936760285815	protein_codin	XM_022988568.1	receptor protein-tyrosine kinase CEPR1-like
134	gene-LOC11136760	2.5612481251	0.5	0.8691339282	0.961743624	2.356847023146	protein_codin	XM_022988575.1	zinc-finger homeodomain protein 2-like%2C transcript variant X2
135	gene-LOC11136766	3.94058560596667	9.3577364509	-0.737879584	0.950825939	-1.24774955465	protein_codin	XM_022988648.1	alanine aminotransferase 2%2C mitochondrial-like
136	gene-LOC11136769	0.5	2.9857282704666	-1.042489693	0.983556510	-2.57808287254	protein_codin	XM_022988700.1	beta-galactosidase-like
137	gene-LOC11136770	10.6241619831667	2.3000030115333	1.1933613907	0.982421583	2.207641392978	protein_codin	XM_022988707.1	inactive protein kinase SELMODRAFT_444075-like%2C transcript variant X2
138	gene-LOC11136770	2.2716068798	0.5	0.7301479895	0.953318698	2.183713186235	protein_codin	XM_022988713.1	CLP protease regulatory subunit CLPX1%2C mitochondrial-like%2C transcript variant X2
139	gene-LOC11136770	11.9554562942333	0.5	2.0483193592	1	4.579597288162	protein_codin	XM_022988727.1	phytolongin Phyl2.2-like
140	gene-LOC11136772	0.992034422	4.9658371884333	-0.847858169	0.955418570	-2.32357487582	protein_codin	XM_022988757.1	uncharacterized LOC111367728%2C transcript variant X2
141	gene-LOC11136773	0.6358831613	2.1861148219	-0.772262385	0.954561439	-1.78153556734	protein_codin	XM_022988765.1	protein PLASTID MOVEMENT IMPAIRED 1-RELATED 1-like
142	gene-LOC11136773	5.0422943958	12.104349959033	-0.841304058	0.957171800	-1.26337334630	protein_codin	XR_002697880.1	two-component response regulator-like APRR3%2C transcript variant X2
143	gene-LOC11136774	2.1520873545	10.290907707733	-1.523039478	0.999999816	-2.25756169646	protein_codin	XM_022988781.1	60S ribosomal protein L9-like
144	gene-LOC11136775	117.2848682184	146.3055343034	-0.963357964	0.954243208	-0.31896745067	protein_codin	XM_022988796.1	uncharacterized LOC111367754
145	gene-LOC11136775	3.2457663791	0.5	0.8667081315	0.961603331	2.698559162595	protein_codin	XM_022988801.1	60S ribosomal export protein NMD3

146	gene-LOC11136778	0.5	2.5127285353666	-0.801158618	0.957947684	-2.32925481692	protein_codin	XM_022988842.1	probable mediator of RNA polymerase II transcription subunit 26c%2C transcript variant X3
147	gene-LOC11136780	5.27187991703333	1.0801683049	1.0095130671	0.970826122	2.287061387593	protein_codin	XR_002697890.1	putative receptor-like protein kinase At5g39000%2C transcript variant X3
148	gene-LOC11136780	20.9147903382667	12.344270529233	0.9557221917	0.975956297	0.760681951099	protein_codin	XM_022988871.1	probable pectinesterase/pectinesterase inhibitor 51%2C transcript variant X2
149	gene-LOC11136781	0.5	5.9237860680666	-1.354785605	0.977731713	-3.56651954000	protein_codin	XM_022988878.1	mitogen-activated protein kinase kinase kinase 3-like%2C transcript variant X2
150	gene-LOC11136782	1.85599181786667	0.5	0.9346285653	0.972602995	1.892190350363	protein_codin	XM_022988889.1	NADP-dependent malic enzyme%2C chloroplastic-like
151	gene-LOC11136784	4.91572865633333	0.5	1.6356651542	0.980946447	3.297405282479	protein_codin	XM_022988903.1	uncharacterized LOC111367840
152	gene-LOC11136784	4.6538716784	0.5	1.3668264921	0.956270066	3.218431431738	protein_codin	XM_022988909.1	alcohol dehydrogenase 1B-like
153	gene-LOC11136784	1396.02160236817	1740.3212748433	-1.098893787	0.974144193	-0.31803239540	protein_codin	XM_022988915.1	beta-1%2C3-galactosyltransferase 7-like%2C transcript variant X2
154	gene-LOC11136785	10.0974973743	2.4055599655666	0.9275250834	0.970892569	2.069553102546	protein_codin	XM_022988934.1	aspartic proteinase Asp1%2C transcript variant X4
155	gene-LOC11136785	3.0403739644	0.5	0.9963016230	0.972709801	2.604248785314	protein_codin	XM_022988935.1	NAC domain-containing protein 2-like
156	gene-LOC11136787	2.9312915195	0.7067508191	0.7169287613	0.953270184	2.052262897239	protein_codin	XM_022988961.1	protein TIC 20-I%2C chloroplastic-like%2C transcript variant X1
157	gene-LOC11136788	2.03556706723333	0.5	0.7088504665	0.953093597	2.025430755730	protein_codin	XM_022988967.1	OTU domain-containing protein 3%2C transcript variant X3
158	gene-LOC11136788	40.2763875662667	11.065804426266	1.5045457747	0.969228803	1.863825960877	protein_codin	XM_022988976.1	PI-PLC X domain-containing protein At5g67130
159	gene-LOC11136788	3.47535190033333	0.5	0.9394101047	0.973640509	2.797159066803	protein_codin	XM_022988979.1	DNA-directed RNA polymerase 2B%2C chloroplastic/mitochondrial-like%2C transcript variant X1
160	gene-LOC11136789	12.5541016588333	6.1313426950666	0.7028310136	0.952813103	1.033883848924	protein_codin	XM_022988991.1	protein furry homolog-like%2C transcript variant X1
161	gene-LOC11136791	2.0762599173	0.5	0.6996756982	0.952603165	2.053987059267	protein_codin	XM_022989027.1	transmembrane protein 33 homolog%2C transcript variant X1
162	gene-LOC11136792	3.65838220303333	0.5	1.2189499540	0.983729315	2.871205805982	protein_codin	XM_022989035.1	elongation of fatty acids protein 3-like
163	gene-LOC11136793	2.0762599173	0.5	0.6996756982	0.952603165	2.053987059267	protein_codin	XR_002697923.1	uncharacterized LOC111367934%2C transcript variant X3
164	gene-LOC11136800	16.8389134938333	5.1241726195	1.0466282668	0.967435077	1.716408071528	protein_codin	XM_022989162.1	heat shock 70 kDa protein 15-like%2C transcript variant X1
165	gene-LOC11136800	2.66845099056667	0.6531665342333	0.7381393366	0.953394432	2.030479734970	protein_codin	XR_002697936.1	uncharacterized LOC111368009%2C transcript variant X3
166	gene-LOC11136801	2.5483395424	0.5	0.7722236578	0.956042524	2.349557516016	protein_codin	XM_022989181.1	30S ribosomal protein S17%2C chloroplastic-like
167	gene-LOC11136802	77.6106848809	60.198876040933	1.1236377544	0.978451888	0.366518734946	protein_codin	XM_022989183.1	agamous-like MADS-box protein AGL65
168	gene-LOC11136802	11.0813659236	3.8652543497666	0.9205877212	0.969174183	1.519500465718	protein_codin	XM_022989184.1	chlorophyll a-b binding protein 4%2C chloroplastic-like
169	gene-LOC11136802	6.76526618286667	2.3000030115333	0.8179619771	0.960176554	1.556510948715	protein_codin	XM_022989187.1	cyclic nucleotide-gated ion channel 17-like
170	gene-LOC11136802	2.0762599173	0.5	0.6996756982	0.952603165	2.053987059267	protein_codin	XM_022989185.1	uncharacterized LOC111368023
171	gene-LOC11136803	2.00974990186667	0.5	0.7043739485	0.952899766	2.007015980117	protein_codin	XM_022989197.1	ABC transporter A family member 7-like
172	gene-LOC11136805	0.5	3.6849536618666	-0.840606792	0.957328213	-2.88164647760	protein_codin	XM_022989230.1	DNA-directed RNA polymerase V subunit 7%2C transcript variant X2
173	gene-LOC11136808	23.1634431832	13.1150773157	0.7311813883	0.953322719	0.820623409414	protein_codin	XM_022989270.1	peroxidase 31-like
174	gene-LOC11136808	50.4872932177333	37.347809941366	0.7706320057	0.955837573	0.434896685540	protein_codin	XR_002697949.1	AUGMIN subunit 8-like%2C transcript variant X4
175	gene-LOC11136808	2.96813768803333	0.5	0.7266124400	0.953311660	2.569558018250	lncRNA	XR_002697947.1	uncharacterized LOC111368086
176	gene-LOC11136814	3.10688397983333	0.5	1.1604430177	0.983150623	2.635468367998	protein_codin	XM_022989378.1	dof zinc finger protein DOF1.6-like
177	gene-LOC11136816	4.85567293223333	1.55316804	0.7276651877	0.953312905	1.644457322106	protein_codin	XM_022989410.1	BRI1 kinase inhibitor 1-like
178	gene-LOC11136817	2.5612481251	11.250468203133	-1.142399043	0.978735335	-2.13506611409	protein_codin	XM_022989418.1	ER membrane protein complex subunit 7 homolog
179	gene-LOC11136817	0.6358831613	4.0675081911	-0.761653372	0.953668570	-2.67731164046	protein_codin	XM_022989422.1	G-type lectin S-receptor-like serine/threonine-protein kinase SD2-5
180	gene-LOC11136818	2.4185190443	0.5	0.7545467141	0.954141492	2.274123898134	protein_codin	XM_022989433.1	pre-mRNA-splicing factor CWC25 homolog%2C transcript variant X1
181	gene-LOC11136819	14.8114800026667	3.7936729546333	0.9779478850	0.975285235	1.965048588560	protein_codin	XM_022989442.1	transmembrane 9 superfamily member 9-like
182	gene-LOC11136822	5.2648337028	0.7331400576333	1.0468476560	0.967423816	2.844227222990	protein_codin	XM_022989469.1	zinc finger MYM-type protein 1-like
183	gene-LOC11136825	0.5	2.5127285353666	-0.801158618	0.957947684	-2.32925481692	protein_codin	XM_022989499.1	uncharacterized LOC111368259
184	gene-LOC11136826	12.0445839422333	5.7587310171333	0.9178046931	0.968500026	1.064561716914	protein_codin	XM_022989506.1	peptidyl-prolyl cis-trans isomerase CYP28%2C chloroplastic
185	gene-LOC11136836	27.0332804641	16.2886317018	0.8623419901	0.961373361	0.730871174368	protein_codin	XM_022989593.1	transcription factor BIM1-like
186	gene-LOC11136838	1.96260276046667	0.5	0.7250116886	0.953309787	1.972768194454	protein_codin	XM_022989619.1	uncharacterized LOC111368386%2C transcript variant X2
187	gene-LOC11136841	0.5	2.8852793205666	-0.859212762	0.951607986	-2.52871099127	protein_codin	XM_022989636.1	oleosin 16 kDa
188	gene-LOC11136846	1.22161994323333	6.6927080818666	-0.970796260	0.959550786	-2.45379456937	protein_codin	XM_022989712.1	squamosa promoter-binding protein 1-like
189	gene-LOC11136847	0.5	2.2125040604	-0.824359966	0.959476976	-2.14568010284	protein_codin	XM_022989717.1	uncharacterized LOC111368472
190	gene-LOC11136849	0.5	4.3722296438	-1.667648783	0.999991447	-3.12836917806	protein_codin	XM_022989752.1	RNA-binding protein NOB1%2C transcript variant X1
191	gene-LOC11136849	0.5	7.2048522727666	-1.381278556	0.986432414	-3.84896884989	protein_codin	XM_022989764.1	beta-amylase-like
192	gene-LOC11136849	2.25224400576667	0.5	0.7304862915	0.953319521	2.171363135961	protein_codin	XM_022989766.1	transcription factor ILR3-like%2C transcript variant X1
193	gene-LOC11136851	2.69426815593333	0.5	0.8963939323	0.964311812	2.429893446933	protein_codin	XM_022989788.1	probable alpha%2Calpha-trehalose-phosphate synthase [UDP-forming] 7
194	gene-LOC11136852	1.96905705183333	0.5	0.6815674662	0.950366313	1.977504912765	protein_codin	XM_022989793.1	uncharacterized LOC111368520

195	gene-LOC11136854	10.4643412359667	4.5321157979	0.8893694288	0.963405112	1.207224863800	protein_codin	XM_022989849.1	mediator of RNA polymerase II transcription subunit 21-like%2C transcript variant X2
196	gene-LOC11136856	0.5	2.8257812237	-1.119397939	0.975307321	-2.49864977434	protein_codin	XM_022989886.1	nuclear transcription factor Y subunit C-2-like%2C transcript variant X2
197	gene-LOC11136857	5.73848880183333	0.6531665342333	1.0881644253	0.969421217	3.135148081735	protein_codin	XM_022989887.1	protein MOR1-like%2C transcript variant X1
198	gene-LOC11136857	39.5089470759	73.498738918933	-1.538048158	0.999999979	-0.89554009798	protein_codin	XM_022989894.1	protein MOR1-like%2C transcript variant X1
199	gene-LOC11136858	33.2803034315	1.292832936	3.5032312114	1	4.686060824593	protein_codin	XM_022989910.1	clathrin light chain 2-like
200	gene-LOC11136858	3.66138144103333	0.5	0.8085780699	0.959839781	2.872388080777	protein_codin	XM_022989922.1	calmodulin-binding protein 60 C-like%2C transcript variant X1
201	gene-LOC11136858	3.3529692446	0.5	0.9892636348	0.973770638	2.745439249947	protein_codin	XM_022989926.1	F-box protein SKIP27-like
202	gene-LOC11136859	6.11947367686667	12.6888813529	-0.775107561	0.954818691	-1.05208540713	protein_codin	XM_022989939.1	probable protein phosphatase 2C 52
203	gene-LOC11136860	5.0090393881	0.6531665342333	1.2811476243	0.982928445	2.939011176430	protein_codin	XM_022989946.1	uncharacterized LOC111368601%2C transcript variant X2
204	gene-LOC11136860	2.79689630953333	0.5	0.9413997486	0.974035097	2.483826769189	protein_codin	XM_022989949.1	CHD3-type chromatin-remodeling factor PICKLE-like%2C transcript variant X1
205	gene-LOC11136861	0.5	1.8126973361	-0.775838238	0.954886876	-1.85813806012	protein_codin	XM_022989972.1	cysteine proteinase 3-like%2C transcript variant X2
206	gene-LOC11136869	3.05973683843333	7.6785969230666	-0.819454182	0.959520819	-1.32743514250	protein_codin	XM_022990100.1	primase homolog protein-like
207	gene-LOC11136870	9.33257697116667	19.8382036754	-0.767806503	0.954181547	-1.08793398997	lncRNA	XR_002698036.1	uncharacterized LOC111368701
208	gene-LOC11136871	4.94612053356667	1.0801683049	0.7817511074	0.957301853	2.195041276250	protein_codin	XM_022990145.1	fructose-bisphosphate aldolase-lysine N-methyltransferase%2C chloroplastic%2C transcript variant X3
209	gene-LOC11136872	0.5	2.4599500583333	-0.805478126	0.958483519	-2.29862902640	protein_codin	XM_022990147.1	DAG protein%2C chloroplastic-like%2C transcript variant X2
210	gene-LOC11136873	70.2403190420667	53.0766269794	0.8642379615	0.961470032	0.404222707500	protein_codin	XM_022990151.1	uncharacterized LOC111368731%2C transcript variant X2
211	gene-LOC11136873	3.7585388543	0.9729997351	0.6975739086	0.952430759	1.949660600583	protein_codin	XM_022990154.1	uncharacterized LOC111368736
212	gene-LOC11136874	3.43623452416667	8.8500162416333	-0.755870091	0.953147849	-1.36485159858	protein_codin	XM_022990157.1	CBS domain-containing protein CBSX5-like
213	gene-LOC11136874	0.662683877666667	4.9855068070666	-1.361036900	0.979567640	-2.91134744494	protein_codin	XM_022990175.1	stem-specific protein TSJT1-like
214	gene-LOC11136876	343.921948781833	461.4263367699	-1.015117473	0.983439225	-0.42401916061	protein_codin	XM_022990188.1	asparagine--tRNA ligase%2C chloroplastic/mitochondrial-like
215	gene-LOC11136877	4.51153422576667	0.9729997351	0.8009302031	0.959364344	2.213106813144	protein_codin	XM_022990203.1	autophagy-related protein 8f-like
216	gene-LOC11136877	2.8702522444	10.237262530166	-1.040160522	0.983915284	-1.83458055165	protein_codin	XM_022990216.1	uncharacterized LOC111368776
217	gene-LOC11136879	2.00974990186667	6.6655130355	-0.850553786	0.954587791	-1.72969994175	protein_codin	XM_022990254.1	small ubiquitin-related modifier 1-like
218	gene-LOC11136879	7.4775687043	1.9325602304666	0.8818161057	0.962655313	1.952055882860	protein_codin	XM_022990256.1	small ubiquitin-related modifier 1-like%2C transcript variant X2
219	gene-LOC11136880	2.03556706723333	0.5	0.7088504665	0.953093597	2.025430755730	protein_codin	XM_022990261.1	ethylene-responsive transcription factor 3-like
220	gene-LOC11136881	2.7636413018	0.5	0.9758625769	0.975506199	2.466570377775	protein_codin	XM_022990279.1	heterogeneous nuclear ribonucleoprotein 1-like
221	gene-LOC11136882	9.55023746073333	2.6734204973	0.9404865693	0.973861590	1.836849831084	protein_codin	XM_022990285.1	probable serine/threonine-protein kinase At1g54610%2C transcript variant X2
222	gene-LOC11136882	0.765312031266667	3.6848927691333	-0.862478323	0.950406724	-2.26750265213	protein_codin	XM_022990298.1	SPX domain-containing membrane protein At4g11810-like
223	gene-LOC11136884	0.6358831613	2.4795587843	-0.779630440	0.955261826	-1.96324981782	protein_codin	XM_022990317.1	uncharacterized LOC111368841
224	gene-LOC11136885	0.5	3.0452263673666	-0.959484033	0.951604986	-2.60654947507	protein_codin	XM_022990325.1	cyclin-D3-3-like
225	gene-LOC11136885	2.18534236223333	6.3992641195	-0.756019383	0.953162469	-1.55004669825	protein_codin	XM_022990329.1	SUMO-conjugating enzyme SCE1-like%2C transcript variant X2
226	gene-LOC11136887	2.08916849996667	0.5	0.7050067510	0.952935613	2.062928856341	protein_codin	XM_022990352.1	ultraviolet-B receptor UVR8-like%2C transcript variant X2
227	gene-LOC11136889	11.0426401755667	5.2245606767	0.7740935570	0.956286934	1.079703512159	protein_codin	XM_022990403.1	PRA1 family protein H-like%2C transcript variant X2
228	gene-LOC11136894	0.5	3.5316653422333	-0.794451279	0.957056426	-2.82034864084	protein_codin	XM_022990435.1	NHP2-like protein 1 homolog
229	gene-LOC11136907	1.4175588286	6.1057592644	-0.997301741	0.976914016	-2.10676210046	protein_codin	XM_022990557.1	outer envelope pore protein 16%2C chloroplastic
230	gene-LOC11136908	2.18534236223333	0.5	0.9277477865	0.970947782	2.127859314327	protein_codin	XM_022990565.1	putative late blight resistance protein homolog R1B-16
231	gene-LOC11136909	57.4294718142	41.963061601466	0.6841302981	0.950790516	0.452671353166	protein_codin	XM_022990579.1	transcription repressor OFP3
232	gene-LOC11136910	1.0614075679	4.3261707866666	-0.772360156	0.954570038	-2.02711188010	protein_codin	XM_022990584.1	uncharacterized LOC111369100
233	gene-LOC11136911	10.0739514165333	18.795385532466	-0.824834551	0.959461915	-0.89974882989	protein_codin	XM_022990595.1	uncharacterized LOC111369112
234	gene-LOC11136912	19.9461015792667	52.2993128373	-1.249378784	0.980619712	-1.39068518847	protein_codin	XM_022990605.1	uncharacterized LOC111369124
235	gene-LOC11136912	2.63519598286667	0.5	0.8648891327	0.961504917	2.397910260912	protein_codin	XM_022990608.1	gamma-tubulin complex component 4 homolog
236	gene-LOC11136914	9.3229231471	27.913384091266	-1.429699113	0.998234370	-1.58210276139	protein_codin	XM_022990628.1	protein NRT1/ PTR FAMILY 3.1-like
237	gene-LOC11136915	68.9749265353	41.348294607866	1.1090107647	0.974456767	0.738244187670	protein_codin	XM_022990635.1	peroxidase A2-like
238	gene-LOC11136916	4.88247364863333	1.605946517	0.7604066739	0.954661014	1.604188410278	protein_codin	XM_022990639.1	lysM domain receptor-like kinase 3
239	gene-LOC11136916	7.84889975526667	2.4590833577666	0.7188632241	0.953287816	1.674369793668	protein_codin	XM_022990642.1	proline-rich receptor-like protein kinase PERK12
240	gene-LOC11136918	8.21568839106667	2.9396694133333	0.7291987527	0.953316403	1.482727540502	protein_codin	XM_022990662.1	calmodulin-binding protein 25-like
241	gene-LOC11136922	3.36874095773333	0.5	1.0112400630	0.970600888	2.752209495434	lncRNA	XR_002698077.1	uncharacterized LOC111369223
242	gene-LOC11136925	3.53599954726667	0.5	0.9186549279	0.968704859	2.822118090013	protein_codin	XM_022990711.1	50S ribosomal protein 5 alpha%2C chloroplastic-like
243	gene-LOC11136929	8.5140551353	2.1861148219	1.0777983633	0.967917295	1.961477254689	protein_codin	XM_022990745.1	calmodulin-binding transcription activator 4-like%2C transcript variant X5

244	gene-LOC11136929	5.17955273626667	1.55316804	0.7074345633	0.9530417157	1.737613598067	protein_codin	XM_022990760.1	histone-lysine N-methyltransferase%2C H3 lysine-9 specific SUVH1-like%2C transcript variant X2
245	gene-LOC11136929	2.7020976264	0.5	0.6901994521	0.9516553100	2.434079799970	lncRNA	XR_002698099.1	uncharacterized LOC111369294
246	gene-LOC11136929	0.5	4.8044003113	-0.9977386338	0.9771374716	-3.264356364004	protein_codin	XM_022990754.1	DEAD-box ATP-dependent RNA helicase 21-like
247	gene-LOC11136932	0.9255244066	4.75323345	-0.8309643456	0.9589832454	-2.360566319420	protein_codin	XM_022990803.1	uncharacterized LOC111369322
248	gene-LOC11136934	0.5	4.6460039875666	-1.2157152249	0.9852347771	-3.215990392381	protein_codin	XM_022990824.1	beta-glucuronosyltransferase GlcAT14A%2C transcript variant X2
249	gene-LOC11136934	3.62324761586667	0.5	1.0543600224	0.9671298057	2.857283404183	protein_codin	XM_022990827.1	uncharacterized LOC111369346
250	gene-LOC11136934	1.75981795563333	6.7182306198	-0.9851197901	0.9696656031	-1.932655123486	protein_codin	XM_022990828.1	receptor protein kinase TMK1-like
251	gene-LOC11136935	2.00974990186667	0.5	0.7043739485	0.9528997665	2.007015980117	protein_codin	XM_022990843.1	uncharacterized LOC111369358%2C transcript variant X2
252	gene-LOC11136936	4.96287579773333	0.5	1.6625279099	0.9727518413	3.311176349812	lncRNA	XR_002698109.1	uncharacterized LOC111369361
253	gene-LOC11136936	831.043281847567	404.3877623733	4.2081823966	1	1.039184277536	lncRNA	XR_002698111.1	uncharacterized LOC111369363%2C transcript variant X2
254	gene-LOC11136936	7.3038558234	2.4599500583333	0.7964122816	0.9589819717	1.570029260694	protein_codin	XM_022990848.1	uncharacterized LOC111369364%2C transcript variant X1
255	gene-LOC11136937	4.12819062053333	0.9729997351	0.7432161241	0.9535224110	2.084998271863	protein_codin	XM_022990864.1	serine/threonine-protein kinase ATG1c-like%2C transcript variant X2
256	gene-LOC11136938	17.3510065236	7.5706225454	0.9035845900	0.9654767353	1.196535509768	protein_codin	XM_022990872.1	L-type lectin-domain containing receptor kinase S.4-like
257	gene-LOC11136940	0.5	2.1129218111	-0.7406900641	0.9512779248	-2.079239381174	protein_codin	XM_022990903.1	auxin response factor 18-like%2C transcript variant X2
258	gene-LOC11136944	59.4166270126667	35.590247613166	1.2791274964	0.9832535105	0.739384737806	lncRNA	XR_002698133.1	uncharacterized LOC111369445
259	gene-LOC11136944	0.5	2.2456129188	-0.7730286608	0.9546290333	-2.167109268675	protein_codin	XM_022990967.1	uncharacterized LOC111369449%2C transcript variant X2
260	gene-LOC11136946	28.1714990202667	38.549647537833	-0.7653648907	0.9539793070	-0.452481337220	protein_codin	XM_022990980.1	vacuolar protein-sorting-associated protein 37 homolog 1-like
261	gene-LOC11136947	0.5	3.3063672792	-1.0745368542	0.9766455611	-2.725246991553	lncRNA	XR_002698139.1	uncharacterized LOC111369475%2C transcript variant X3
262	gene-LOC11136949	3.3890873828	0.7067508191	0.9588106419	0.9761238079	2.261623279937	protein_codin	XM_022991024.1	putative HVA22-like protein g%2C transcript variant X4
263	gene-LOC11136949	3.0532825471	0.7067508191	0.8792503919	0.9624410826	2.111087545784	protein_codin	XM_022991032.1	lipid phosphate phosphatase delta-like%2C transcript variant X1
264	gene-LOC11136950	36.7969401048	51.664481282733	-0.7680476560	0.9542017575	-0.489586983441	protein_codin	XR_002698149.1	uncharacterized LOC111369501%2C transcript variant X1
265	gene-LOC11136953	12.9125241271333	4.8042785259	0.8332090936	0.9604425210	1.426379346596	protein_codin	XM_022991090.1	uncharacterized LOC111369530%2C transcript variant X3
266	gene-LOC11136953	3.26064206383333	0.7067508191	0.7957230126	0.9589194717	2.205882523087	protein_codin	XM_022991092.1	probable 1-deoxy-D-xylulose-5-phosphate synthase 2%2C chloroplastic%2C transcript variant X1
267	gene-LOC11136954	3.8320950839	0.5	1.0246831335	0.9690945926	2.938133358404	protein_codin	XM_022991105.1	inorganic pyrophosphatase 1-like
268	gene-LOC11136954	7.47013086193333	1.5259729936333	1.1085149904	0.9743183123	2.291404086534	protein_codin	XM_022991110.1	transmembrane 9 superfamily member 7
269	gene-LOC11136954	27.8473151107	11.838101043033	1.4401535804	0.9581698347	1.234100561384	protein_codin	XM_022991114.1	LMBR1 domain-containing protein 2 homolog A-like%2C transcript variant X1
270	gene-LOC11136955	62.1818760850667	37.672417096766	1.0764470847	0.9677736188	0.722985543898	protein_codin	XM_022991120.1	protein DETOXIFICATION 27-like
271	gene-LOC11136956	75.2746226103	48.152017620733	0.9283033311	0.9710858186	0.644567320217	protein_codin	XM_022991134.1	putative late blight resistance protein homolog R1A-3
272	gene-LOC11136957	2.774670305	0.5	0.7303734358	0.9533192466	2.472316356111	protein_codin	XM_022991141.1	uncharacterized LOC111369571%2C transcript variant X2
273	gene-LOC11136962	6.239585125	1.605946517	0.9604807064	0.9761808233	1.958026258894	protein_codin	XM_022991204.1	squamosa promoter-binding-like protein 1
274	gene-LOC11136962	12.3387123768667	23.8065366629	-1.0755223429	0.9764651297	-0.948165906956	protein_codin	XM_022991210.1	L-cysteine desulfhydrase%2C transcript variant X2
275	gene-LOC11136964	3.6990750531	0.9729997351	0.7424515269	0.9534964465	1.926653255255	protein_codin	XM_022991238.1	chitinase 2-like
276	gene-LOC11136967	969.081705540233	1170.9413364559	-1.3857042742	0.9879756827	-0.272978586521	lncRNA	XR_002698213.1	uncharacterized LOC111369677
277	gene-LOC11136969	9.07717428456667	0.9729997351	1.4810706898	0.9676873539	3.221731940512	protein_codin	XM_022991303.1	cytochrome P450 86A1-like
278	gene-LOC11136969	2.15854164583333	0.6531665342333	0.7183454324	0.9532830702	1.724534148007	protein_codin	XM_022991308.1	UPF0503 protein At3g09070%2C chloroplastic-like%2C transcript variant X2
279	gene-LOC11136970	0.5	3.9527533008666	-1.1184581565	0.9752060236	-2.982857916522	protein_codin	XM_022991310.1	probable anion transporter 4%2C chloroplastic%2C transcript variant X1
280	gene-LOC11136970	2.08916849996667	0.5	0.7050067510	0.9529356131	2.062928856341	protein_codin	XR_002698230.1	serine/arginine-rich splicing factor SR45a-like%2C transcript variant X4
281	gene-LOC11136971	2.8379807877	0.5	0.7388008329	0.9534055307	2.504864822807	protein_codin	XM_022991331.1	MLO-like protein 1
282	gene-LOC11136971	2.46109147383333	0.5	0.9626316258	0.9762078754	2.299298280873	protein_codin	XR_002698235.1	uncharacterized LOC111369719%2C transcript variant X2
283	gene-LOC11136972	86.4183154656	60.063706616933	1.2715833396	0.9843290603	0.524843600635	protein_codin	XM_022991350.1	uncharacterized LOC111369723%2C transcript variant X1
284	gene-LOC11136972	4.36363851023333	1.0801683049	0.7199146579	0.9532945593	2.014275470391	protein_codin	XM_022991363.1	asparagine--tRNA ligase%2C cytoplasmic 1-like%2C transcript variant X1
285	gene-LOC11136975	4.065271766	0.5	0.9329147418	0.9721993877	3.023351800604	protein_codin	XM_022991389.1	protein NRT1/ PTR FAMILY 5.2-like
286	gene-LOC11136975	0.9255244066	3.9527533008666	-0.7547162205	0.9530354852	-2.094514976131	protein_codin	XM_022991390.1	protein NRT1/ PTR FAMILY 5.2-like
287	gene-LOC11136976	0.662683877666667	3.0452263673666	-0.7999300656	0.9577877031	-2.200156749223	protein_codin	XR_002698250.1	ETHYLENE INSENSITIVE 3-like 3 protein%2C transcript variant X2
288	gene-LOC11136978	5.51437402093333	2.1861148219	0.7850662723	0.9577263781	1.334827945624	protein_codin	XM_022991425.1	ras GTPase-activating protein-binding protein 1%2C transcript variant X2
289	gene-LOC11136986	0.5	3.0452263673666	-0.9594840334	0.9516049861	-2.606549475073	protein_codin	XM_022991560.1	GATA transcription factor 15-like
290	gene-LOC11136986	1.81628251883333	6.8781776666333	-1.0129931145	0.9829384396	-1.921037753662	protein_codin	XM_022991572.1	importin subunit alpha-like
291	gene-LOC11136987	3.71843792713333	0.5	1.3818228423	0.9500488924	2.894696689057	protein_codin	XM_022991608.1	N-terminal acetyltransferase A complex auxiliary subunit NAA15-like%2C transcript variant X1
292	gene-LOC11136989	152.341495990033	193.52065609143	-1.3031811048	0.9707124722	-0.345178597474	lncRNA	XR_002698324.1	uncharacterized LOC111369895

293	gene-LOC11136991	6.5485892443	17.249058897766	-1.186424725	0.985115545	-1.39726160417	protein_codin	XM_022991636.1	SNF1-related protein kinase regulatory subunit beta-3-like%2C transcript variant X2
294	gene-LOC11136992	0.9255244066	4.4333393564333	-0.772546561	0.954586453	-2.26005085953	protein_codin	XM_022991652.1	protein disulfide-isomerase 5-1%2C transcript variant X1
295	gene-LOC11136993	2.69426815593333	0.5	0.8963939323	0.964311812	2.429893446933	protein_codin	XM_022991666.1	protein disulfide isomerase-like 1-4
296	gene-LOC11136996	4.455965691	0.5	0.9752194555	0.975570468	3.155738124595	protein_codin	XR_002698338.1	uncharacterized LOC111369964%2C transcript variant X4
297	gene-LOC11137002	5.56092923943333	1.0930574109666	0.9623569629	0.976204397	2.346956801467	protein_codin	XM_022991737.1	zinc finger MYM-type protein 1-like
298	gene-LOC11137015	36.8121198950667	24.4214254419	0.8532296855	0.960969268	0.592033421693	protein_codin	XM_022991850.1	kinesin-like protein KIN-14N
299	gene-LOC11137016	2.89803651176667	0.5	0.8284192969	0.960368959	2.535075771227	protein_codin	XM_022991857.1	basic leucine zipper 61-like
300	gene-LOC11137022	3.04584470473333	0.5	0.8021320923	0.959452673	2.606842386532	protein_codin	XM_022991910.1	villin-3-like%2C transcript variant X1
301	gene-LOC11137023	3.63256503766667	0.5	1.0317381756	0.968454409	2.860988628189	protein_codin	XM_022991923.1	thioredoxin reductase NTRC-like
302	gene-LOC11137029	0.5	2.3000030115333	-0.746106397	0.952051421	-2.20163575017	protein_codin	XM_022991968.1	nuclear pore complex protein NUP93A-like
303	gene-LOC11137030	4.96834653803333	1.2333348391333	0.7628059965	0.954909623	2.010201272412	protein_codin	XM_022991977.1	cytokinin dehydrogenase 6-like
304	gene-LOC11137031	26.3697880806	2.5059480228	1.9992533254	1	3.395457580729	protein_codin	XM_022991987.1	uncharacterized LOC111370311
305	gene-LOC11137035	0.5	2.8852793205666	-0.859212762	0.951607986	-2.52871099127	protein_codin	XM_022992022.1	uncharacterized LOC111370353%2C transcript variant X2
306	gene-LOC11137038	4.6445542566	1.1329467819	0.6826305353	0.950551517	2.035460051298	protein_codin	XM_022992063.1	putative UDP-glucose flavonoid 3-O-glucosyltransferase 3
307	gene-LOC11137039	2.44818289113333	0.5	0.9691321958	0.976035677	2.291711338371	protein_codin	XM_022992072.1	scarecrow-like protein 23
308	gene-LOC11137039	9.36036123853333	17.755912406366	-0.846619859	0.955778402	-0.92366338365	protein_codin	XM_022992084.1	2%2C3-bisphosphoglycerate-independent phosphoglycerate mutase
309	gene-LOC11137042	0.5	3.1319804033333	-0.747528801	0.952234103	-2.64707518577	protein_codin	XM_022992116.1	sulfiredoxin%2C chloroplastic/mitochondrial
310	gene-LOC11137043	2.1520873545	0.6531665342333	0.7201465146	0.953295569	1.720213859224	protein_codin	XM_022992145.1	putative UPF0481 protein At3g02645
311	gene-LOC11137045	0.5	2.9329497934333	-1.062324105	0.979286965	-2.55235237508	protein_codin	XM_022992164.1	transforming growth factor-beta receptor-associated protein 1 homolog
312	gene-LOC11137045	3.07362897213333	0.9398908767333	0.7269702201	0.953312082	1.709377851313	protein_codin	XM_022992171.1	vacuolar protein sorting-associated protein 2 homolog 2
313	gene-LOC11137048	1.96905705183333	0.5	0.6815674662	0.950366313	1.977504912765	protein_codin	XM_022992202.1	cationic amino acid transporter 1-like%2C transcript variant X1
314	gene-LOC11137050	2.66845099056667	0.5	0.8904698014	0.963535251	2.416002514674	protein_codin	XR_002698424.1	probable alpha%2Calpha-trehalose-phosphate synthase [UDP-forming] 7%2C transcript variant X2
315	gene-LOC11137051	29.5509240237333	15.0877216986	0.9750332902	0.975589011	0.969828272478	protein_codin	XM_022992229.1	heat stress transcription factor A-4a-like%2C transcript variant X3
316	gene-LOC11137051	2.2121430786	0.5	0.7760830569	0.956551078	2.145444700270	protein_codin	XM_022992231.1	GDSL esterase/lipase At5g45920-like
317	gene-LOC11137051	0.938432989266667	6.7438140504666	-1.154783191	0.980910834	-2.84523912164	protein_codin	XM_022992238.1	protein MAIN-LIKE 2-like%2C transcript variant X1
318	gene-LOC11137056	2.46109147383333	0.5	0.9626316258	0.976207875	2.299298280873	protein_codin	XM_022992324.1	uncharacterized LOC111370561
319	gene-LOC11137058	26.7465898719	14.981298044	0.9309239661	0.971725185	0.836192332464	protein_codin	XM_022992373.1	lipase-like PAD4
320	gene-LOC11137060	88.3614126035667	111.86440670236	-1.369699389	0.982391018	-0.34026268113	protein_codin	XM_022992394.1	ras-related protein RABA1f-like
321	gene-LOC11137061	4.37851419493333	0.8599173533333	0.7574526169	0.954382405	2.348171474563	protein_codin	XM_022992406.1	vesicle-associated protein 4-2-like
322	gene-LOC11137064	4.0209877551	0.5	1.3811414973	0.950292194	3.007549942782	protein_codin	XM_022992461.1	protein GID8 homolog
323	gene-LOC11137064	7.05490742816667	1.1797505542	1.3183780245	0.975031741	2.580145303250	protein_codin	XM_022992464.1	uncharacterized LOC111370646
324	gene-LOC11137065	8.43881962093333	3.5190319103333	0.8044004361	0.959608165	1.261862619813	protein_codin	XM_022992476.1	1-aminocyclopropane-1-carboxylate oxidase-like
325	gene-LOC11137066	5.3587363575	10.823405539733	-0.744932947	0.951895541	-1.01418976433	protein_codin	XM_022992486.1	chaperonin-like RBCX protein 1%2C chloroplastic
326	gene-LOC11137066	118.999224958333	228.29140837316	-3.321835976	0.999999999	-0.93992438822	lncRNA	XR_002698475.1	uncharacterized LOC111370667
327	gene-LOC11137069	2.85088937036667	0.5	0.8783512168	0.962369055	2.511412056140	protein_codin	XM_022992524.1	peroxisomal membrane protein PMP22-like
328	gene-LOC11137069	2.60194097513333	0.5	0.7922052050	0.958561254	2.379588234981	protein_codin	XM_022992532.1	protein phosphatase 1 regulatory inhibitor subunit PPP1R8 homolog
329	gene-LOC11137072	0.9255244066	4.0063984784666	-0.753902439	0.952956905	-2.11396297909	protein_codin	XM_022992562.1	uncharacterized LOC111370721%2C transcript variant X1
330	gene-LOC11137072	0.5	5.7587310171333	-1.896715540	0.996329674	-3.52575093719	protein_codin	XM_022992575.1	F-box protein At-B
331	gene-LOC11137075	3.58999260816667	0.8599173533333	0.7885018342	0.958143138	2.061710959373	protein_codin	XM_022992649.1	protein disulfide isomerase-like 1-6
332	gene-LOC11137077	434.039908294133	295.8447146334	4.7937964166	1	0.552987577928	lncRNA	XR_002698497.1	uncharacterized LOC111370772
333	gene-LOC11137077	1.85599181786667	6.4834789464333	-0.805347371	0.958468877	-1.80457780100	protein_codin	XM_022992642.1	65-kDa microtubule-associated protein 1-like
334	gene-LOC11137077	3.30321449333333	0.6531665342333	0.7797013041	0.957031001	2.338347873970	protein_codin	XM_022992643.1	uncharacterized LOC111370779
335	gene-LOC11137078	450.220728910467	524.2453436437	-1.209966694	0.985496238	-0.21960965993	protein_codin	XM_022992651.1	probable galacturonosyltransferase 12
336	gene-LOC11137079	0.5	2.5127285353666	-0.801158618	0.957947684	-2.32925481692	protein_codin	XM_022992669.1	autophagy-related protein 8C
337	gene-LOC11137081	3.94802344833333	0.5	1.2970917348	0.979958663	2.981130558359	protein_codin	XM_022992691.1	glutamate receptor 2.8-like
338	gene-LOC11137083	0.5	3.0468379831	-0.840950890	0.957251166	-2.60731278614	protein_codin	XM_022992727.1	alpha-1%2C3/1%2C6-mannosyltransferase ALG2-like%2C transcript variant X2
339	gene-LOC11137083	3.488260483	0.5	0.8151360748	0.960099072	2.802507776210	protein_codin	XM_022992731.1	F-box/LRR-repeat protein At4g14103-like
340	gene-LOC11137086	19.6832610503333	4.2658668818666	1.9159161851	0.999999998	2.206058409289	protein_codin	XM_022992764.1	fatty-acid-binding protein 3%2C chloroplastic%2C transcript variant X4
341	gene-LOC11137087	2.0762599173	0.5	0.6996756982	0.952603165	2.053987059267	protein_codin	XM_022992779.1	uncharacterized LOC111370877

342	gene-LOC11137090	9.64256464146667	3.8652543497666	0.9172263525	0.9683626634	1.318853559781	protein_codin	XM_022992810.1	glucose-6-phosphate 1-dehydrogenase%2C chloroplastic-like
343	gene-LOC11137092	18.7507779520667	35.108049941866	-1.0238272077	0.9846527582	-0.90485141180	protein_codin	XM_022992827.1	cation/H(+) antiporter 15-like
344	gene-LOC11137103	3.26064206383333	0.5	0.9928877293	0.9732266044	2.705156078291	protein_codin	XM_022992952.1	uncharacterized LOC111371032
345	gene-LOC11137115	84.9907045437667	57.421966040366	0.9728430488	0.9757816551	0.565702334869	protein_codin	XM_022993062.1	uncharacterized LOC111371151
346	gene-LOC11137117	0.662683877666667	3.4721672453333	-0.7793950917	0.9552370158	-2.38944371450	protein_codin	XM_022993076.1	uncharacterized LOC111371171
347	gene-LOC11137117	53.0062244339333	79.553148643933	-1.8712096659	0.9837147227	-0.58575724935	protein_codin	XM_022993077.1	uncharacterized LOC111371172
348	gene-LOC11137125	5.7009954845	1.0801683049	0.9077664942	0.9662769911	2.399957737383	protein_codin	XM_022993152.1	protein LURP-one-related 7-like
349	gene-LOC11137126	0.5	2.3000030115333	-0.7461063970	0.9520514212	-2.20163575017	protein_codin	XM_022993156.1	uncharacterized LOC111371260
350	gene-LOC11137127	0.5	2.2456129188	-0.7730286608	0.9546290333	-2.16710926867	protein_codin	XM_022993166.1	ubiquitin-like-conjugating enzyme ATG10%2C transcript variant X1
351	gene-LOC11137128	4.14109920323333	0.5	1.1011804887	0.9723362974	3.050013763996	protein_codin	XM_022993175.1	uncharacterized LOC111371280
352	gene-LOC11137129	2.80433415186667	0.7331400576333	0.7242711561	0.9533083903	1.935497525233	protein_codin	XM_022993190.1	60S ribosomal protein L32-1
353	gene-LOC11137130	271.824350265233	205.4272057769	0.9989851075	0.9723077329	0.404047441987	protein_codin	XM_022993217.1	formin-like protein 14
354	gene-LOC11137135	4.30417470906667	0.7067508191	1.1774924492	0.9827471405	2.606463083747	protein_codin	XM_022993283.1	probable serine/threonine-protein kinase PBL9
355	gene-LOC11137136	25.2932170106333	12.556935160366	0.9321093717	0.9720110922	1.010266162384	lncRNA	XR_002698558.1	uncharacterized LOC111371364
356	gene-LOC11137137	145.181712129933	106.50022131336	0.9441450439	0.9745371975	0.447003306689	protein_codin	XM_022993321.1	histone H2A-like
357	gene-LOC11137142	1.85599181786667	0.5	0.9346285653	0.9726029951	1.892190350363	protein_codin	XM_022993410.1	U5 small nuclear ribonucleoprotein 40 kDa protein-like%2C transcript variant X1
358	gene-LOC11137143	1.95614846913333	0.5	0.6824614660	0.9505243641	1.968015873129	protein_codin	XM_022993420.1	potassium transporter 4-like
359	gene-LOC11137144	4.65327975553333	0.8599173533333	0.9452034515	0.9747164449	2.435978010485	protein_codin	XM_022993443.1	uncharacterized LOC111371449
360	gene-LOC11137147	13.0025801002667	4.8067568422333	0.7227300858	0.9533054560	1.435662196135	protein_codin	XM_022993494.1	uncharacterized LOC111371475%2C transcript variant X3
361	gene-LOC11137149	6.865031206	2.8257812237	0.7739809824	0.9562721071	1.280616504026	protein_codin	XM_022993520.1	epoxide hydrolase A-like
362	gene-LOC11137149	4.2505732763	1.2861133161333	0.7194511370	0.9532925458	1.724639670744	lncRNA	XR_002698591.1	uncharacterized LOC111371495
363	gene-LOC11137151	3.92122273196667	0.5	1.1694289130	0.9830164823	2.971303591657	protein_codin	XM_022993549.1	uncharacterized LOC111371514%2C transcript variant X4
364	gene-LOC11137153	1.36395739586667	5.1530401743	-0.7605142363	0.9535703204	-1.91762525861	protein_codin	XM_022993573.1	protein STRUBBELIG-RECEPTOR FAMILY 6-like
365	gene-LOC11137154	17.2564081352	7.9447849463333	0.9552928737	0.9759271395	1.119052129942	protein_codin	XM_022993587.1	uncharacterized LOC111371543%2C transcript variant X2
366	gene-LOC11137155	2.7537319571	0.5	0.6975232012	0.9524265585	2.461388137184	protein_codin	XM_022993610.1	uncharacterized LOC111371555%2C transcript variant X4
367	gene-LOC11137156	3.46303524053333	0.5	1.0479147668	0.9673686938	2.792037068760	protein_codin	XM_022993627.1	uncharacterized LOC111371569%2C transcript variant X1
368	gene-LOC11137157	7.7071542255	1.6126661369	1.0323314680	0.9684057426	2.256750465910	protein_codin	XM_022993633.1	nucleolin-like
369	gene-LOC11137157	3.72489221846667	0.8599173533333	0.8969393752	0.9643908961	2.114928766956	protein_codin	XM_022993636.1	protein PHR1-LIKE 3-like%2C transcript variant X2
370	gene-LOC11137158	3.6990750531	0.9729997351	0.7424515269	0.9534964465	1.926653255255	protein_codin	XM_022993643.1	F-box protein At2g02240-like
371	gene-LOC11137158	0.5	2.9857282704666	-1.0424896930	0.9835565106	-2.57808287254	protein_codin	XM_022993645.1	F-box protein PP2-B10-like
372	gene-LOC11137159	8.62125800073333	2.6198362124333	1.0281543737	0.9687658688	1.718421780703	protein_codin	XM_022993665.1	malate dehydrogenase-like
373	gene-LOC11137162	8.89473590476667	2.0865325725666	0.9254513492	0.9703784427	2.091844328318	protein_codin	XM_022993690.1	probable glutathione S-transferase
374	gene-LOC11137164	4291.5054621358	3238.0897474179	2.1085949355	1	0.406340862451	lncRNA	XR_002698613.1	uncharacterized LOC111371643
375	gene-LOC11137165	3.7784936512	10.690653539333	-1.0375309328	0.9842563625	-1.50046694653	protein_codin	XM_022993743.1	homocysteine S-methyltransferase 2-like%2C transcript variant X2
376	gene-LOC11137165	0.5	5.3061458158333	-1.3269398824	0.9721547963	-3.40766432217	lncRNA	XR_002698615.1	uncharacterized LOC111371652
377	gene-LOC11137168	2.1317409295	8.9571848114333	-1.0406668509	0.9838376804	-2.07101325526	protein_codin	XM_022993804.1	pre-mRNA-processing factor 19-like
378	gene-LOC11137171	2.08916849996667	0.5	0.7050067510	0.9529356131	2.062928856341	lncRNA	XR_002698642.1	uncharacterized LOC111371716%2C transcript variant X9
379	gene-LOC11137175	59.6125981947333	78.965698482133	-0.7400189388	0.9511765094	-0.40560885017	lncRNA	XR_002698651.1	uncharacterized LOC111371752
380	gene-LOC11137175	12.1346951413	3.5990054337666	0.9932123113	0.9731772980	1.753467678711	protein_codin	XM_022993896.1	NAC domain-containing protein 43-like
381	gene-LOC11137176	2.74786958863333	0.5	0.7977912973	0.9591061931	2.458313536799	protein_codin	XM_022993906.1	50S ribosomal protein L11%2C chloroplastic
382	gene-LOC11137176	35.2980830986333	20.583427031233	1.0245798730	0.9691049276	0.778106635188	protein_codin	XM_022993913.1	catalase isozyme 3-like
383	gene-LOC11137177	1.85599181786667	0.5	0.9346285653	0.9726029951	1.892190350363	protein_codin	XM_022993927.1	reticulon-like protein B2
384	gene-LOC11137179	2.77107914416667	0.5	0.9413317527	0.9740221446	2.470447915895	protein_codin	XM_022993944.1	uncharacterized LOC111371790
385	gene-LOC11137181	3.16634778103333	0.5	0.9642200631	0.9761966175	2.662819724968	protein_codin	XM_022993992.1	60S ribosomal protein L24-like
386	gene-LOC11137187	3.46303524053333	0.8599173533333	0.7722794216	0.9560497553	2.009767154607	protein_codin	XM_022994033.1	uncharacterized LOC111371870
387	gene-LOC11137191	3.97482416473333	0.6531665342333	1.1533008645	0.9829937029	2.605368261251	protein_codin	XM_022994063.1	trans-resveratrol di-O-methyltransferase-like
388	gene-LOC11137194	47.5742196641667	21.6228392646	1.2830315340	0.9826140054	1.137624019130	protein_codin	XM_022994093.1	putative UPF0481 protein At3g02645
389	gene-LOC11137206	1.85599181786667	0.5	0.9346285653	0.9726029951	1.892190350363	protein_codin	XM_022994217.1	glutaredoxin-C9-like
390	gene-LOC11137206	37.9671260120667	22.499947920233	0.9270100395	0.9707651444	0.754829133673	protein_codin	XM_022994224.1	serine carboxypeptidase-like 18

391	gene-LOC11137207	2.25224400576667	0.5	0.7304862915	0.9533195212	2.171363135961	protein_codin	XM_022994249.1	probable WRKY transcription factor 28%2C transcript variant X2
392	gene-LOC11137210	52.8945343791333	81.818552966733	-1.3766291315	0.9847983504	-0.629309366536	protein_codin	XM_022994276.1	probable glutathione S-transferase%2C transcript variant X2
393	gene-LOC11137211	1.51771547986667	7.4725910191	-0.8630174338	0.9502062153	-2.299707204850	protein_codin	XR_002698687.1	protein DENND6A%2C transcript variant X8
394	gene-LOC11137211	2.72393200276667	0.5	0.9684666577	0.9760725167	2.445690689952	protein_codin	XM_022994293.1	lipase 3
395	gene-LOC11137211	58.2041564932	37.2703147343	1.5168792013	0.9689724105	0.643095180841	protein_codin	XM_022994294.1	protein ASPARTIC PROTEASE IN GUARD CELL 1-like
396	gene-LOC11137213	2.9645465272	0.5	0.9493068166	0.9753215323	2.567811439343	lncRNA	XR_002698692.1	uncharacterized LOC111372131%2C transcript variant X2
397	gene-LOC11137213	5.1850234766	1.6126661369	0.6826201133	0.9505498664	1.684902726580	protein_codin	XM_022994322.1	methyltransferase-like protein 1%2C transcript variant X2
398	gene-LOC11137217	2.5806109991	0.5	0.7018832565	0.9527544811	2.367712686195	lncRNA	XR_002698706.1	uncharacterized LOC111372173
399	gene-LOC11137219	330.0139904052	238.48691042263	1.0604206329	0.9670473504	0.468617101736	protein_codin	XM_022994408.1	protein ENHANCED DISEASE RESISTANCE 2-like%2C transcript variant X1
400	gene-LOC11137219	2.64066672316667	0.5	0.7971634153	0.9590494566	2.400902231389	protein_codin	XM_022994426.1	uncharacterized LOC111372199
401	gene-LOC11137220	38.5318369235333	8.5317337638	2.5567414905	1	2.175140113448	protein_codin	XM_022994433.1	protein NUCLEAR FUSION DEFECTIVE 4-like
402	gene-LOC11137220	14.2893025831	4.4845062177333	0.7956452971	0.9589116541	1.671914458634	protein_codin	XM_022994434.1	protein NUCLEAR FUSION DEFECTIVE 4-like
403	gene-LOC11137221	38.2742182552667	26.7650089943	0.7298711502	0.9533180275	0.516024773746	protein_codin	XM_022994442.1	uncharacterized protein C57A10.07-like
404	gene-LOC11137222	28.9190236514333	4.9855068070666	2.2224785327	0.9999999999	2.536206769705	lncRNA	XR_002698718.1	uncharacterized LOC111372229
405	gene-LOC11137223	9.57605462606667	3.8388651112333	0.9735981419	0.9757198000	1.318751513941	protein_codin	XM_022994474.1	tryptophan aminotransferase-related protein 4-like
406	gene-LOC11137227	111.015586949867	55.6699225818	1.8942491686	0.9999999511	0.995792267865	protein_codin	XR_002698738.1	TOM1-like protein 4%2C transcript variant X2
407	gene-LOC11137227	1.22161994323333	5.2058186513333	-0.7607577973	0.9535912461	-2.091329535458	protein_codin	XM_022994527.1	TITAN-like protein
408	gene-LOC11137228	7.42396727153333	2.8852793205666	0.7449329824	0.9535824186	1.363479359100	protein_codin	XM_022994545.1	anthocyanidin 3-O-glucosyltransferase 2-like
409	gene-LOC11137229	16.8908519301	5.7842535551	0.8814606380	0.9626240400	1.546039395803	protein_codin	XM_022994554.1	septum-promoting GTP-binding protein 1-like
410	gene-LOC11137229	25.0626479384	13.010326169533	0.8003694582	0.9593197056	0.945881716505	protein_codin	XM_022994555.1	uncharacterized LOC111372292
411	gene-LOC11137230	3.10402084933333	0.5	0.7724037531	0.9560658904	2.634138247966	protein_codin	XM_022994579.1	uncharacterized protein At1g76660-like%2C transcript variant X3
412	gene-LOC11137235	4.96287579773333	1.605946517	0.8018475384	0.9594332374	1.627752502376	protein_codin	XM_022994653.1	DExH-box ATP-dependent RNA helicase DExH6-like%2C transcript variant X2
413	gene-LOC11137239	2.0762599173	0.5	0.6996756982	0.9526031653	2.053987059267	protein_codin	XM_022994731.1	uncharacterized LOC111372391%2C transcript variant X3
414	gene-LOC11137239	1.54353264523333	7.2063421031	-0.8126313849	0.9591880301	-2.223031145910	protein_codin	XM_022994742.1	isoaspartyl peptidase/L-asparaginase 1
415	gene-LOC11137250	14.3510213036667	0.8863065918666	1.8646964564	0.9999966308	4.017203757742	protein_codin	XM_022994852.1	protein UPSTREAM OF FLC-like
416	gene-LOC11137253	0.5	3.2051734141666	-0.8581777332	0.9519824316	-2.680402415187	protein_codin	XR_002698791.1	BTB/POZ domain-containing protein At2g30600%2C transcript variant X3
417	gene-LOC11137253	39.9167326673	30.2849076052	0.9168959191	0.9682847360	0.398394625618	protein_codin	XM_022994888.1	protein NLP5-like
418	gene-LOC11137254	1.85599181786667	0.5	0.9346285653	0.9726029951	1.892190350363	protein_codin	XM_022994890.1	SRSF protein kinase 1-like%2C transcript variant X2
419	gene-LOC11137255	2.72393200276667	0.7067508191	0.7396357793	0.9534231965	1.946417134747	protein_codin	XM_022994902.1	UDP-rhamnose/UDP-galactose transporter 2
420	gene-LOC11137255	8.82404280563333	22.553532205133	-1.4497452862	0.9995451096	-1.353841703210	protein_codin	XM_022994914.1	calcium-binding protein CML38-like
421	gene-LOC11137256	2.73496100596667	0.5	0.7931074243	0.9586591417	2.451520263767	protein_codin	XM_022994925.1	uncharacterized LOC111372562%2C transcript variant X1
422	gene-LOC11137257	25.4582043926	14.981237151266	0.7922875533	0.9585703104	0.764973900725	lncRNA	XR_002698798.1	uncharacterized LOC111372575
423	gene-LOC11137257	17.3561731583333	7.1972050596333	1.1818240712	0.9826043431	1.269940216068	protein_codin	XM_022994946.1	receptor-like protein kinase 2
424	gene-LOC11137258	35.7617413303667	21.086129715133	0.8538757417	0.9609947709	0.762122668896	protein_codin	XM_022994956.1	subtilisin-like protease SBT1.6
425	gene-LOC11137259	0.5	2.8069783056333	-0.7568365263	0.9532417792	-2.489017913698	protein_codin	XM_022994962.1	uncharacterized LOC111372592
426	gene-LOC11137260	12.2773689962	2.2456129188	1.2542318810	0.9856799734	2.450820254768	protein_codin	XR_002698801.1	cystathionine gamma-synthase 1%2C chloroplastic%2C transcript variant X2
427	gene-LOC11137265	152.8287554054	210.41593581546	-1.3497023206	0.9763965124	-0.461327952675	protein_codin	XM_022995032.1	NAP1-related protein 2-like
428	gene-LOC11137265	12.4339902106667	5.2041461429	0.8151668882	0.9601000910	1.256555966630	protein_codin	XM_022995040.1	1-phosphatidylinositol-3-phosphate 5-kinase FAB1A-like
429	gene-LOC11137268	0.629428869966667	5.0646745226333	-1.2825095163	0.9727659790	-3.008354301079	protein_codin	XR_002698810.1	probable starch synthase 4%2C chloroplastic/amyloplastic%2C transcript variant X4
430	gene-LOC11137268	2.0762599173	0.5	0.6996756982	0.9526031653	2.053987059267	protein_codin	XM_022995066.1	TORTIFOLIA1-like protein 2%2C transcript variant X2
431	gene-LOC11137269	7.3887451618	0.8599173533333	1.0988172386	0.9717363461	3.103059456636	protein_codin	XM_022995085.1	uncharacterized LOC111372695
432	gene-LOC11137269	2.21859736993333	0.5	0.7780618088	0.9568138950	2.149647871633	protein_codin	XM_022995086.1	uncharacterized LOC111372696
433	gene-LOC11137274	5.77721454986667	1.0801683049	1.0079730632	0.9710279738	2.419117952005	protein_codin	XM_022995131.1	shaggy-related protein kinase eta-like
434	gene-LOC11137274	0.5	2.4055599655666	-0.7612302589	0.9536319638	-2.266372763231	protein_codin	XR_002698820.1	D-aminoacyl-tRNA deacylase%2C transcript variant X3
435	gene-LOC11137277	0.5	2.9329497934333	-1.0623241055	0.9792869654	-2.552352375089	protein_codin	XM_022995179.1	tobamovirus multiplication protein 3-like%2C transcript variant X2
436	gene-LOC11137281	2.51469290656667	0.7067508191	0.7020470682	0.9527658136	1.831108674160	protein_codin	XM_022995216.1	uncharacterized LOC111372811
437	gene-LOC11137283	0.5	4.3194511668	-1.3229410006	0.9716882579	-3.110848013954	lncRNA	XR_002698829.1	uncharacterized LOC111372830
438	gene-LOC11137288	2.69426815593333	0.5	0.8963939323	0.9643118126	2.429893446933	protein_codin	XM_022995303.1	mediator of RNA polymerase II transcription subunit 33B-like
439	gene-LOC11137288	6.0455258191	1.5259729936333	0.7354387329	0.9533550084	1.986138395729	protein_codin	XM_022995307.1	zinc finger MYM-type protein 1-like

AQRNA_Control_vs_AQRNA_G7

440	gene-LOC11137289	0.5	4.3261707866666	-1.1683845458	0.9831263119	-3.113090620504	protein_codin	XM_022995311.1	uncharacterized LOC111372892
441	gene-LOC11137289	2.63519598286667	6.8245933817	-0.7579710370	0.9533439747	-1.372832830002	protein_codin	XM_022995325.1	uncharacterized LOC111372899
442	gene-LOC11137291	52.1101958761333	36.575269753766	0.7623839818	0.9548637759	0.510697172982	lncRNA	XR_002698846.1	uncharacterized LOC111372917
443	gene-LOC11137293	13.5101859407	2.9125352596666	1.3742530157	0.9529707387	2.213700110106	protein_codin	XM_022995362.1	probable (S)-N-methylcoclaurine 3'-hydroxylase isozyme 2
444	gene-LOC11137294	1.22161994323333	6.245169992	-0.8481175909	0.9553400082	-2.353945322397	protein_codin	XM_022995373.1	probable 6-phosphogluconolactonase 5%2C chloroplastic
445	gene-LOC11137296	4.8159636332	0.6531665342333	1.1219173629	0.9780117724	2.882301718207	protein_codin	XM_022995397.1	protein HOTHEAD-like
446	gene-LOC11137299	21.8146978742667	10.5562899232	0.8950528869	0.9641183385	1.047197613828	protein_codin	XM_022995431.1	elongation factor 2-like%2C transcript variant X1
447	gene-LOC11137300	2.87670653573333	0.5	0.8103728623	0.9599240265	2.524418053528	protein_codin	XM_022995455.1	probable serine/threonine-protein kinase At1g09600
448	gene-LOC11137301	5.84372456526667	12.3425980208	-0.8318608356	0.9588731191	-1.078686016824	protein_codin	XM_022995461.1	ubiquitin carboxyl-terminal hydrolase 27%2C transcript variant X2
449	gene-LOC11137302	9.83987870603333	3.4118633405666	0.7967549412	0.9590127054	1.528080670069	protein_codin	XR_002698856.1	auxin response factor 17-like%2C transcript variant X2
450	gene-LOC11137303	2.0762599173	0.5	0.6996756982	0.9526031653	2.053987059267	lncRNA	XR_002698860.1	uncharacterized LOC111373031%2C transcript variant X1
451	gene-LOC11137304	1.96905705183333	0.5	0.6815674662	0.9503663139	1.977504912765	protein_codin	XM_022995501.1	histidinol-phosphate aminotransferase%2C chloroplastic-like
452	gene-LOC11137304	2.66845099056667	0.5	0.8904698014	0.9635352514	2.416002514674	protein_codin	XM_022995508.1	uncharacterized LOC111373048%2C transcript variant X2
453	gene-LOC11137305	1.66650722383333	5.9722014561	-0.8430821070	0.9567334387	-1.841435260584	protein_codin	XM_022995524.1	protein GIGANTEA-like%2C transcript variant X2
454	gene-LOC11137305	0.5	2.5867273540666	-0.7713494860	0.9544815042	-2.371127999262	protein_codin	XM_022995527.1	S-adenosylmethionine carrier 1%2C chloroplastic/mitochondrial-like%2C transcript variant X1
455	gene-LOC11137307	6.72457333283333	0.5	1.6440397840	0.9795342469	3.749442733076	protein_codin	XR_002698883.1	DExH-box ATP-dependent RNA helicase DExH5%2C mitochondrial-like%2C transcript variant X18
456	gene-LOC11137309	3.0310565426	0.6531665342333	0.7600840146	0.9546289124	2.214297985307	protein_codin	XM_022995561.1	alanine--glyoxylate aminotransferase 2 homolog 1%2C mitochondrial
457	gene-LOC11137310	4.83631005823333	1.0801683049	0.8243830666	0.9603060226	2.162650618050	protein_codin	XM_022995579.1	probable GTP diphosphokinase CRSH%2C chloroplastic
458	gene-LOC11137310	4.38359330713333	1.0801683049	0.7836560918	0.9575472820	2.020857835434	protein_codin	XM_022995581.1	CBL-interacting serine/threonine-protein kinase 1-like%2C transcript variant X2
459	gene-LOC11137310	1.08820828426667	6.7182915125333	-1.2138080810	0.9853324634	-2.626139680286	protein_codin	XM_022995585.1	5'-nucleotidase domain-containing protein 4
460	gene-LOC11137310	23.1666427158667	41.644034208433	-1.4075904705	0.9945014963	-0.846060848913	protein_codin	XM_022995587.1	1%2C4-dihydroxy-2-naphthoyl-CoA thioesterase 1-like
461	gene-LOC11137311	3.1941320484	0.5	0.8612726627	0.9613200286	2.675423956341	protein_codin	XR_002698892.1	uncharacterized LOC111373119%2C transcript variant X3
462	gene-LOC11137312	5.56797545366667	2.2456129188	0.6850645822	0.9509407986	1.310043582426	protein_codin	XM_022995608.1	GDP-L-galactose phosphorylase 1-like
463	gene-LOC11137321	1.4175588286	5.6244282937333	-0.8047257643	0.9583972311	-1.988297849997	protein_codin	XM_022995720.1	pyruvate kinase isozyme A%2C chloroplastic
464	gene-LOC11137322	3.78494794253333	0.7067508191	0.9115953057	0.9670774016	2.420999902585	protein_codin	XM_022995733.1	probable serine/threonine-protein kinase irlF
465	gene-LOC11137324	2.08916849996667	0.5	0.7050067510	0.9529356131	2.062928856341	protein_codin	XM_022995752.1	50S ribosomal protein L18
466	gene-LOC11137325	0.629428869966667	3.4926426718333	-0.9805242612	0.9665376782	-2.472203792597	protein_codin	XM_022995764.1	probable pectinesterase/pectinesterase inhibitor 36
467	gene-LOC11137326	0.5	3.7053073029666	-0.9973615916	0.9769459458	-2.889593198693	protein_codin	XM_022995775.1	tetraspanin-6-like
468	gene-LOC11137328	12.5479514730333	5.1989772460666	0.7724395342	0.9560705371	1.271152108717	protein_codin	XM_022995787.1	casein kinase 1-like protein HD16%2C transcript variant X2
469	gene-LOC11137330	1.85599181786667	6.6111229427333	-1.0204815898	0.9843403088	-1.832704993429	protein_codin	XM_022995814.1	casein kinase 1-like protein 10%2C transcript variant X2
470	gene-LOC11137331	0.629428869966667	3.946033681	-1.1388299861	0.9781252844	-2.648288012850	protein_codin	XM_022995831.1	cysteine-rich and transmembrane domain-containing protein WIH2-like
471	gene-LOC11137332	2.69426815593333	11.142432932733	-1.1363567520	0.9777132287	-2.048098925224	protein_codin	XM_022995832.1	GEM-like protein 5
472	gene-LOC11137338	3.15343919836667	0.6531665342333	0.8191240094	0.9602042356	2.271403337091	protein_codin	XM_022995896.1	UDP-glucuronate 4-epimerase 3-like
473	gene-LOC11137338	0.5	2.6734204973	-0.7967574353	0.9573659775	-2.418686774228	protein_codin	XM_022995905.1	uncharacterized LOC111373382%2C transcript variant X8
474	gene-LOC11137340	3.3290316587	0.5	1.0469300488	0.9674195806	2.735102590403	protein_codin	XM_022995951.1	transcription factor UNE12-like
475	gene-LOC11137344	3.44308044363333	0.5	0.9404902875	0.9738622947	2.783699888219	protein_codin	XM_022995977.1	uncharacterized LOC111373445
476	gene-LOC11137345	0.5	2.5127285353666	-0.8011586181	0.9579476842	-2.329254816926	protein_codin	XR_002698948.1	WD repeat-containing protein 48-like%2C transcript variant X4
477	gene-LOC11137346	2.74786958863333	0.5	0.7977912973	0.9591061931	2.458313536799	protein_codin	XM_022995997.1	8-hydroxygeraniol dehydrogenase-like
478	gene-LOC11137348	2.73496100596667	0.5	0.7931074243	0.9586591417	2.451520263767	protein_codin	XM_022996017.1	serine/arginine-rich splicing factor SR45-like
479	gene-LOC11137350	1.24098281723333	4.3722296438	-0.7588765726	0.9534262652	-1.816886038148	lncRNA	XR_002698952.1	uncharacterized LOC111373501
480	gene-LOC11137353	2.87670653573333	0.6531665342333	0.6888147768	0.9514756554	2.138895273824	protein_codin	XR_002698955.1	serine/threonine-protein phosphatase PP1 isozyme 3-like%2C transcript variant X5
481	gene-LOC11137354	3.9286605743	11.4102934645	-0.9715714834	0.9601147778	-1.538226463852	protein_codin	XM_022996086.1	probable protein phosphatase 2C 50
482	gene-LOC11137356	40.1334648317333	16.047905323733	1.1756275089	0.9828125356	1.322420713541	protein_codin	XM_022996120.1	hsp70-Hsp90 organizing protein 3-like
483	gene-LOC11137362	10.1960299667333	1.5327535062	1.1628396288	0.9831429838	2.733809908691	protein_codin	XM_022996176.1	protein FAR1-RELATED SEQUENCE 5-like
484	gene-LOC11137365	9.17275622396667	21.299721939533	-1.1240556174	0.9758774004	-1.215407392526	protein_codin	XM_022996225.1	sorbitol dehydrogenase-like
485	gene-LOC11137365	55.6258489983667	40.737373975466	0.7475110231	0.9536956176	0.449402466626	protein_codin	XM_022996226.1	uncharacterized LOC111373657
486	gene-LOC11137371	1.85599181786667	0.5	0.9346285653	0.9726029951	1.892190350363	protein_codin	XM_022996291.1	serine/threonine-protein kinase/endoribonuclease IRE1a-like
487	gene-LOC11137372	2.2121430786	0.5	0.7760830569	0.9565510789	2.145444700270	protein_codin	XM_022996294.1	probable serine/threonine-protein kinase PIX13%2C transcript variant X2
488	gene-LOC11137378	39.1046651227667	26.313351386233	1.0943974824	0.9706940722	0.571545720659	protein_codin	XM_022996343.1	uncharacterized protein At3g17950-like

489	gene-LOC1113737	12.6357914645	6.9308952509	0.6971387790	0.952394764	0.866402413037	protein_codin	XM_022996361.1	ATP-dependent zinc metalloprotease FTSH%2C chloroplastic-like
490	gene-LOC1113738	0.5	2.2456129188	-0.773028660	0.954629033	-2.16710926867	protein_codin	XR_002699031.1	uncharacterized LOC111373805%2C transcript variant X7
491	gene-LOC11137384	11.6467562804667	4.3245591709	0.8146749815	0.960083859	1.429303225608	protein_codin	XR_002699034.1	putative late blight resistance protein homolog R1B-17%2C transcript variant X2
492	gene-LOC11137384	1.2305457369	7.7033745458333	-1.150912712	0.980232145	-2.64619229145	protein_codin	XM_022996433.1	uncharacterized LOC111373849%2C transcript variant X2
493	gene-LOC1113738	1.8291911015	0.5	0.7379989413	0.953392084	1.871205805969	protein_codin	XM_022996443.1	UPF0503 protein At3g09070%2C chloroplastic-like
494	gene-LOC11137387	12.7281186453	5.7851202556333	0.8595535379	0.961238714	1.137600334798	protein_codin	XM_022996464.1	acyltransferase-like protein At1g54570%2C chloroplastic
495	gene-LOC1113738	22.4081766041	11.036936871433	1.1846242728	0.982526814	1.021685428480	protein_codin	XM_022996488.1	protein NRT1/ PTR FAMILY 5.10-like
496	gene-LOC11137392	7.63320636773333	3.1251998907666	0.6886944466	0.951460133	1.288340726995	protein_codin	XM_022996521.1	uncharacterized LOC111373928
497	gene-LOC1113739	0.5	5.2873428977666	-1.031046319	0.984758789	-3.40254289223	protein_codin	XM_022996557.1	mitochondrial import receptor subunit TOM20-like%2C transcript variant X2
498	gene-LOC1113739	2.1520873545	0.5	0.9667103261	0.976141508	2.105736638929	protein_codin	XM_022996589.1	F-box protein SKIP23-like%2C transcript variant X2
499	gene-LOC1113740	6.72555688383333	14.210491257533	-0.853872737	0.953492561	-1.07923079496	protein_codin	XM_022996609.1	uncharacterized LOC111374004
500	gene-LOC11137401	15.3200141682	35.567203391533	-1.309563706	0.970759579	-1.21512990990	protein_codin	XM_022996617.1	cytochrome P450 94A1-like
501	gene-LOC11137402	1.95614846913333	0.5	0.6824614660	0.950524364	1.968015873129	protein_codin	XM_022996637.1	serine/threonine-protein phosphatase 4 regulatory subunit 2%2C transcript variant X1
502	gene-LOC1113740	2.7020976264	0.5	0.6901994521	0.951655310	2.434079799970	protein_codin	XM_022996639.1	cytokinin riboside 5'-monophosphate phosphoribohydrolase LOG3-like
503	gene-LOC1113740	7.4368758542	1.1329467819	1.2517425352	0.985725451	2.714616594251	protein_codin	XM_022996682.1	NEDD8-conjugating enzyme Ubc12-like%2C transcript variant X1
504	gene-LOC1113740	1.95614846913333	7.5706834381	-0.829269602	0.959156333	-1.95240767123	protein_codin	XM_022996684.1	30S ribosomal protein S13%2C chloroplastic-like
505	gene-LOC1113740	30.7882281575667	14.047381872	0.9925568767	0.973276717	1.132077575249	protein_codin	XR_002699074.1	CTP synthase 1-like%2C transcript variant X2
506	gene-LOC1113741	4.2366811426	0.7067508191	0.9244576869	0.970130910	2.583660998727	protein_codin	XM_022996795.1	F-box protein At4g05010-like
507	gene-LOC1113741	5.897325998	2.4259744994333	0.7280112030	0.953313556	1.281496561346	protein_codin	XM_022996798.1	glycerophosphodiester phosphodiesterase GDPDL4-like
508	gene-LOC1113741	2.72393200276667	7.1716216289666	-0.847226243	0.955606576	-1.39660868442	protein_codin	XR_002699085.1	cleavage and polyadenylation specificity factor subunit CG7185%2C transcript variant X3
509	gene-LOC1113741	11.7281419806	4.6384785598	1.1835715545	0.982553208	1.338250895820	protein_codin	XM_022996811.1	nuclear transcription factor Y subunit B-3-like
510	gene-LOC1113741	77.5967927472	53.8731389819	0.8098059271	0.959899796	0.526430895809	protein_codin	XM_022996814.1	uncharacterized LOC111374182%2C transcript variant X3
511	gene-LOC1113741	0.798567038966667	3.7853417190666	-0.849940707	0.954781833	-2.24493811362	protein_codin	XR_002699092.1	uncharacterized LOC111374196%2C transcript variant X5
512	gene-LOC1113742	0.9255244066	4.75323345	-0.830964345	0.958983245	-2.36056631942	protein_codin	XM_022996846.1	grpE protein homolog 2%2C mitochondrial-like%2C transcript variant X2
513	gene-LOC11137424	12.3119116605	5.8395103484333	0.6870041325	0.951223789	1.076135478119	protein_codin	XM_022996897.1	dnaJ homolog subfamily B member 13-like
514	gene-LOC1113742	102.189952382067	158.61690823846	-1.376233910	0.984658720	-0.63429321437	protein_codin	XR_002699099.1	uncharacterized LOC111374252%2C transcript variant X3
515	gene-LOC1113743	5.555066871	1.1797505542	1.0546852103	0.967122163	2.235322429453	protein_codin	XM_022997006.1	FT-interacting protein 1-like
516	gene-LOC1113743	1.8291911015	5.3317292464666	-0.824932664	0.959456566	-1.54339771401	protein_codin	XM_022997013.1	uncharacterized LOC111374358
517	gene-LOC1113743	2.7020976264	0.5	0.6901994521	0.951655310	2.434079799970	protein_codin	XM_022997017.1	RING-H2 finger protein ATL48-like%2C transcript variant X2
518	gene-LOC1113743	4.93001241816667	1.1329467819	0.7095368852	0.953115993	2.121511185807	protein_codin	XM_022997047.1	extensin-2-like%2C transcript variant X2
519	gene-LOC1113744	2.2716068798	0.5	0.7301479895	0.953318698	2.183713186235	protein_codin	XM_022997164.1	AP-2 complex subunit mu
520	gene-LOC11137451	2.08916849996667	0.5	0.7050067510	0.952935613	2.062928856341	lncRNA	XR_002699157.1	uncharacterized LOC111374518
521	gene-LOC1113745	272.869522977733	237.1152931498	0.8363405958	0.960497093	0.202622552945	protein_codin	XM_022997235.1	probable endo-1%2C3(4)-beta-glucanase ARB_01444
522	gene-LOC1113745	3.17339399523333	0.5	0.9806593731	0.974964276	2.666026651316	protein_codin	XR_002699161.1	UDP-glucose 6-dehydrogenase 2-like%2C transcript variant X4
523	gene-LOC1113745	3.99517058973333	1.0801683049	0.7153459942	0.953249031	1.887000984688	protein_codin	XR_002699162.1	ribosome biogenesis protein WDR12 homolog%2C transcript variant X2
524	gene-LOC11137457	2.96813768803333	0.5	0.7266124400	0.953311660	2.569558018250	protein_codin	XM_022997272.1	E3 ubiquitin-protein ligase Praja-2-like%2C transcript variant X2
525	gene-LOC1113746	2.8107884432	9.5976570211	-1.143828804	0.978983823	-1.77170738635	protein_codin	XM_022997395.1	uncharacterized LOC111374684
526	gene-LOC1113747	3.4065706773	0.5	0.8714594633	0.961886645	2.768320139169	protein_codin	XM_022997407.1	transcription initiation factor TFIID subunit 12b-like%2C transcript variant X1
527	gene-LOC11137474	0.5	1.8126973361	-0.775838238	0.954886876	-1.85813806012	protein_codin	XM_022997482.1	uncharacterized LOC111374745
528	gene-LOC1113747	11.7946519960333	3.4654476254666	1.2559281365	0.985624887	1.767019232386	protein_codin	XM_022997490.1	metallothionein-like protein type 2
529	gene-LOC11137477	8.72329423146667	3.0920301396666	0.6891851993	0.951523528	1.496318669686	protein_codin	XM_022997511.1	KH domain-containing protein At2g38610-like
530	gene-LOC1113747	5.32548134976667	1.55316804	0.8341959816	0.960459205	1.777698004985	protein_codin	XM_022997525.1	AP2-like ethylene-responsive transcription factor TOE3
531	gene-LOC1113748	8.1427240843	2.2456129188	0.9636941360	0.976203030	1.858402248844	protein_codin	XM_022997539.1	transcription factor bHLH78-like%2C transcript variant X2
532	gene-LOC1113748	5.82337814023333	1.605946517	0.8645746115	0.961488050	1.858432455576	protein_codin	XM_022997600.1	pentatricopeptide repeat-containing protein At3g51320
533	gene-LOC1113748	2.15854164583333	0.5	0.9576639473	0.976078277	2.110056927711	protein_codin	XM_022997576.1	uncharacterized LOC111374835%2C transcript variant X5
534	gene-LOC11137484	0.5	2.1928344418	-0.754119384	0.952977800	-2.13279689317	protein_codin	XM_022997587.1	protein Asterix
535	gene-LOC11137484	5.1378763352	1.1797505542	0.8030823005	0.959517905	2.122690316881	protein_codin	XM_022997593.1	pre-mRNA-splicing factor ATP-dependent RNA helicase DEAH1%2C transcript variant X2
536	gene-LOC1113748	1.8291911015	0.5	0.7379989413	0.953392084	1.871205805969	protein_codin	XM_022997619.1	TOM1-like protein 9
537	gene-LOC1113748	0.5	3.0452263673666	-0.959484033	0.951604986	-2.60654947507	protein_codin	XM_022997625.1	uncharacterized LOC111374869

538	gene-LOC11137487	1.6507355107	8.0256251703	-0.9869035844	0.9708374271	-2.2815047951222	protein_codin	XM_022997631.1	histidine-containing phosphotransfer protein 4-like
539	gene-LOC11137487	205.250171382533	480.0027029246	-1.7634694799	0.9747649128	-1.2256591031160	protein_codin	XM_022997632.1	cell division control protein 2 homolog
540	gene-LOC11137489	6.85956046566667	2.1861148219	0.8275336591	0.9603556599	1.649746958931	protein_codin	XM_022997653.1	ethylene-responsive transcription factor RAP2-12-like%2C transcript variant X1
541	gene-LOC11137491	0.5	2.1928344418	-0.7541193844	0.9529778007	-2.1327968931744	protein_codin	XM_022997679.1	uncharacterized LOC111374918
542	gene-LOC11137494	85.3081300564667	121.57183239493	-1.1080238391	0.9743445373	-0.5110538567544	protein_codin	XM_022997703.1	probable carboxylesterase 8
543	gene-LOC11137494	2.74786958863333	0.5	0.7977912973	0.9591061931	2.458313536799	lncRNA	XR_002699219.1	uncharacterized LOC111374947
544	gene-LOC11137495	2.66845099056667	0.5	0.8904698014	0.9635352514	2.416002514674	protein_codin	XM_022997723.1	ATP-dependent RNA helicase DEAH11%2C chloroplastic-like
545	gene-LOC11137497	5.22571632666667	1.2861133161333	0.9071040016	0.9661443614	2.022611050962	protein_codin	XM_022997732.1	delta(24)-sterol reductase%2C transcript variant X1
546	gene-LOC11137498	13.6432059715333	5.6252341015333	0.9856735503	0.9742930381	1.278197655955	protein_codin	XM_022997760.1	haloacid dehalogenase-like hydrolase domain-containing protein 3%2C transcript variant X5
547	gene-LOC11137498	6.21376795966667	2.2133098683	0.7141637161	0.9532313551	1.489262922207	protein_codin	XR_002699235.1	uncharacterized LOC111374988%2C transcript variant X2
548	gene-LOC11137498	13.6206435648	5.3325959470666	0.8029282037	0.9595072916	1.352884947484	protein_codin	XM_022997763.1	oxygen-dependent coproporphyrinogen-III oxidase%2C chloroplastic-like
549	gene-LOC11137500	10.3442297878333	4.0523964429	0.9015061172	0.9651145030	1.351979004871	protein_codin	XM_022997801.1	serine/threonine-protein kinase HT1-like%2C transcript variant X1
550	gene-LOC11137501	0.5	2.1928344418	-0.7541193844	0.9529778007	-2.1327968931744	protein_codin	XM_022997805.1	uncharacterized LOC111375011
551	gene-LOC11137504	0.629428869966667	2.5662519275666	-0.7385719999	0.9509403294	-2.0275475487211	protein_codin	XM_022997835.1	probable E3 ubiquitin-protein ligase BAH1-like
552	gene-LOC11137510	0.992034422	5.4983350204333	-0.8438826820	0.9565287655	-2.4705327289500	protein_codin	XM_022997894.1	transcription factor GLABRA 3-like
553	gene-LOC11137511	6.0271464961	1.6126661369	0.7240305479	0.9533079297	1.902027336764	protein_codin	XM_022997904.1	uncharacterized LOC111375114%2C transcript variant X2
554	gene-LOC11137511	2.51469290656667	0.6531665342333	0.7585585326	0.9544816290	1.944859449660	protein_codin	XM_022997911.1	uncharacterized LOC111375119%2C transcript variant X3
555	gene-LOC11137512	0.5	2.6198362124333	-0.8581252509	0.9520013524	-2.3894766198177	protein_codin	XM_022997920.1	uncharacterized LOC111375125%2C transcript variant X3
556	gene-LOC11137513	2.94321655113333	9.2785687353666	-0.8273987694	0.9593203831	-1.6565085853255	protein_codin	XM_022997929.1	probable LRR receptor-like serine/threonine-protein kinase At1g63430
557	gene-LOC11137516	0.938432989266667	4.2190022168666	-0.7633628492	0.9538126833	-2.1685762103711	protein_codin	XM_022997959.1	vacuolar protein sorting-associated protein 60.1-like
558	gene-LOC11137516	3.0403739644	0.5	0.9963016230	0.9727098011	2.604248785314	protein_codin	XM_022997964.1	uncharacterized LOC111375167%2C transcript variant X1
559	gene-LOC11137518	0.5	2.1861148219	-1.0350065102	0.9845091010	-2.1283691780611	protein_codin	XM_022997983.1	plastidial pyruvate kinase 2-like
560	gene-LOC11137518	3.0403739644	0.5	0.9963016230	0.9727098011	2.604248785314	protein_codin	XM_022997984.1	G-type lectin S-receptor-like serine/threonine-protein kinase At5g24080%2C transcript variant X1
561	gene-LOC11137518	36.069513019	71.8483451605	-1.7568993669	0.9810472243	-0.9941749831711	protein_codin	XM_022997991.1	anthocyanidin 3-O-glucoside 2''-O-glucosyltransferase-like%2C transcript variant X3
562	gene-LOC11137520	155.7699885663	207.30692835813	-0.9829780113	0.9682272664	-0.4123510299888	protein_codin	XM_022998005.1	protein indeterminate-domain 12-like%2C transcript variant X1
563	gene-LOC11137523	6.88537763103333	14.451217635633	-0.7967568453	0.9573658993	-1.0695833701633	protein_codin	XM_022998030.1	non-specific phospholipase C1
564	gene-LOC11137524	0.5	3.5317871276333	-0.7944647782	0.9570582274	-2.8203983896560	protein_codin	XM_022998042.1	rho GDP-dissociation inhibitor 1-like
565	gene-LOC11137527	23.902546421	10.450732969133	1.0842413877	0.9687590799	1.193560191455	protein_codin	XM_022998071.1	protein GAMETE EXPRESSED 1
566	gene-LOC11137542	6.1408036529	1.339697601	0.9457177679	0.9748039823	2.196520085184	lncRNA	XR_002699304.1	uncharacterized LOC111375421
567	gene-LOC11137542	2.64066672316667	0.5	0.7971634153	0.9590494566	2.400902231389	protein_codin	XM_022998241.1	uncharacterized LOC111375425
568	gene-LOC11137544	5.23862490933333	1.1329467819	0.9808007882	0.9749462398	2.209108072536	protein_codin	XM_022998274.1	protein CHROMATIN REMODELING 5-like
569	gene-LOC11137546	4.3299918744	1.0801683049	0.7496204275	0.9538100977	2.003108196218	protein_codin	XM_022998284.1	mitochondrial adenine nucleotide transporter ADNT1-like%2C transcript variant X1
570	gene-LOC11137546	6.85956046566667	1.8126973361	0.9111061920	0.9669725066	1.919978076863	lncRNA	XR_002699311.1	uncharacterized LOC111375467%2C transcript variant X3
571	gene-LOC11137548	18.5577021971667	6.1857327877666	1.0638582078	0.9670767329	1.585001669820	protein_codin	XM_022998314.1	luminal-binding protein 5-like
572	gene-LOC11137549	0.9255244066	3.5726161952333	-0.7853201129	0.9558932156	-1.9486379936900	protein_codin	XM_022998334.1	ORM1-like protein 2
573	gene-LOC11137558	1.53309556486667	5.4916154005666	-0.7763277792	0.9549328078	-1.8407829618255	protein_codin	XM_022998421.1	probable protein phosphatase 2C 33
574	gene-LOC11137558	0.6358831613	2.1861148219	-0.7722623857	0.9545614391	-1.7815355673422	lncRNA	XR_002699341.1	uncharacterized LOC111375586
575	gene-LOC11137561	0.5	3.4654476254666	-1.1236655386	0.9758268246	-2.7930417146511	protein_codin	XM_022998464.1	heat shock 70 kDa protein%2C mitochondrial
576	gene-LOC11137563	2.0762599173	0.5	0.6996756982	0.9526031653	2.053987059267	protein_codin	XM_022998488.1	COX assembly mitochondrial protein 2 homolog%2C transcript variant X1
577	gene-LOC11137565	10.161791408	1.8730012409	1.4337644936	0.9555453128	2.439730994714	protein_codin	XM_022998509.1	40S ribosomal protein S3a-like
578	gene-LOC11137567	0.5	2.8069783056333	-0.7568365263	0.9532417792	-2.4890179136988	protein_codin	XM_022998539.1	nucleobase-ascorbate transporter 12-like
579	gene-LOC11137571	5.7624263877	1.3133083625333	0.8461811875	0.9607292499	2.133470717598	protein_codin	XM_022998583.1	probable lysophospholipase BODYGUARD 3
580	gene-LOC11137574	4.93509153033333	1.55316804	0.6847615949	0.9508919439	1.667862916457	protein_codin	XM_022998616.1	histone deacetylase 19-like%2C transcript variant X2
581	gene-LOC11137577	0.5	5.6056253756333	-1.3063734286	0.9706981621	-3.4868753321733	lncRNA	XR_002699365.1	uncharacterized LOC111375779%2C transcript variant X3
582	gene-LOC11137582	7.98935762843333	16.369411033066	-1.0163270311	0.9836773489	-1.0348509996399	protein_codin	XM_022998736.1	probable polyamine transporter At3g19553
583	gene-LOC11137589	0.5	2.1861148219	-1.0350065102	0.9845091010	-2.1283691780611	protein_codin	XM_022998804.1	putative hydrolase YtaP
584	gene-LOC11137589	2.54149362293333	0.5	0.8159166805	0.9601229231	2.345676610808	protein_codin	XR_002699374.1	GDP-Man:Man(3)GlcNAc(2)-PP-Dol alpha-1%2C2-mannosyltransferase-like%2C transcript variant X2
585	gene-LOC11137589	0.5	2.2456129188	-0.7730286608	0.9546290333	-2.1671092686755	protein_codin	XM_022998811.1	cell division control protein 2 homolog A-like%2C transcript variant X1
586	gene-LOC11137591	6.31580419036667	1.0801683049	0.9680820937	0.9760877013	2.547710322449	protein_codin	XM_022998827.1	NAC domain-containing protein 83-like

587	gene-LOC11137592	4.55312310426667	1.1329467819	0.7838498035	0.9575724623	2.006776371705	protein_codin	XM_022998847.1	4-sulfomuconolactone hydrolase
588	gene-LOC11137598	2.25224400576667	0.5	0.7304862915	0.9533195212	2.171363135961	protein_codin	XM_022998913.1	uncharacterized LOC111375984
589	gene-LOC11137599	31.6742435598667	17.301776482033	1.4584491006	0.9641198704	0.872389989497	protein_codin	XM_022998918.1	universal stress protein PHOS34-like
590	gene-LOC11137599	2.8440434509	7.3579579143	-0.7834630975	0.9556789946	-1.371361918055	protein_codin	XM_022998919.1	beta-glucosidase 11-like
591	gene-LOC11137599	2.44531976066667	0.5	0.6834549292	0.9506825516	2.290023130602	lncRNA	XR_002699393.1	uncharacterized LOC111375996
592	gene-LOC11137601	0.662683877666667	3.2067850299	-0.7588333683	0.9534223243	-2.274734918639	protein_codin	XM_022998943.1	acid phosphatase 1-like
593	gene-LOC11137603	3.38263309146667	0.9729997351	0.7334088131	0.9533345123	1.797635381564	protein_codin	XM_022998961.1	protein LONGIFOLIA 1-like%2C transcript variant X1
594	gene-LOC11137603	99.5225564567	151.140201521	-1.2272057585	0.9843002670	-0.602792001641	protein_codin	XM_022998963.1	beta-carotene isomerase D27%2C chloroplastic-like%2C transcript variant X1
595	gene-LOC11137604	6.56248137803333	2.6658950696	0.6813585281	0.9503295319	1.299621427170	protein_codin	XM_022998970.1	uncharacterized LOC111376044
596	gene-LOC11137607	9.1127004999	1.4928641352333	1.2662325988	0.9849296688	2.609795779780	protein_codin	XM_022999000.1	xyloglucan galactosyltransferase XLT2-like
597	gene-LOC11137611	15.3694325172333	6.7718149046666	0.7612889192	0.9547492414	1.182449452223	protein_codin	XM_022999043.1	vacuolar-processing enzyme-like
598	gene-LOC11137613	20.7465481977	7.2498617512	0.8982635384	0.9645890125	1.516845931893	protein_codin	XM_022999077.1	protein DA1-related 1-like%2C transcript variant X14
599	gene-LOC11137616	2.74786958863333	0.5	0.7977912973	0.9591061931	2.458313536799	protein_codin	XM_022999107.1	1-aminocyclopropane-1-carboxylate oxidase homolog 1-like
600	gene-LOC11137621	3.32715207923333	0.5	0.9823641090	0.9747454313	2.734287811198	protein_codin	XM_022999164.1	protein EMBRYONIC FLOWER 1-like%2C transcript variant X1
601	gene-LOC11137621	1.95614846913333	0.5	0.6824614660	0.9505243641	1.968015873129	protein_codin	XM_022999171.1	eukaryotic translation initiation factor 3 subunit L-like
602	gene-LOC11137623	3.9044674678	10.876123124066	-0.8276349731	0.9593042203	-1.477966691692	protein_codin	XM_022999189.1	omega-6 fatty acid desaturase%2C chloroplastic-like
603	gene-LOC11137624	2.00974990186667	0.5	0.7043739485	0.9528997665	2.007015980117	protein_codin	XM_022999195.1	leucine aminopeptidase 1-like%2C transcript variant X3
604	gene-LOC11137628	0.5	3.2051734141666	-0.8581777332	0.9519824316	-2.680402415187	protein_codin	XM_022999232.1	protein SCAI-like
605	gene-LOC11137629	0.992034422	5.9186780639666	-1.1260961244	0.9761516648	-2.576812900277	protein_codin	XM_022999238.1	alpha-glucan phosphorylase%2C H isozyme-like
606	gene-LOC11137630	2.40561046163333	0.5	0.7495359276	0.9538054195	2.266403047099	lncRNA	XR_002699450.1	uncharacterized LOC111376305%2C transcript variant X2
607	gene-LOC11137631	12.8556679358	6.0249799331	0.8025502323	0.9594813159	1.093376228137	protein_codin	XM_022999264.1	dirigent protein 19-like
608	gene-LOC11137634	4.9435129237	1.55316804	0.6915393449	0.9518197555	1.670322677944	protein_codin	XM_022999294.1	E3 ubiquitin-protein ligase rnf8-B-like
609	gene-LOC11137634	49.6022613664667	77.289660400333	-1.4260546292	0.9978181043	-0.639869532696	protein_codin	XM_022999305.1	uncharacterized LOC111376349
610	gene-LOC11137635	28.8570008253	17.117979405766	0.7958983443	0.9589360397	0.753408947912	protein_codin	XM_022999306.1	GRIP and coiled-coil domain-containing protein 2-like
611	gene-LOC11137636	7.7672099496	2.8333675441	0.7500002635	0.9538311675	1.454878702804	protein_codin	XM_022999326.1	60S ribosomal protein L3
612	gene-LOC11137637	5.22473277563333	0.8599173533333	1.1555630526	0.9830788874	2.603087336795	protein_codin	XR_002699464.1	mitogen-activated protein kinase kinase kinase 9-like%2C transcript variant X5
613	gene-LOC11137637	64.1714961449	25.9669462687	1.8187505453	0.9995048842	1.305256257715	protein_codin	XR_002699465.1	sterol 3-beta-glucosyltransferase UGT80A2%2C transcript variant X2
614	gene-LOC11137641	0.5	2.3000030115333	-0.7461063970	0.9520514212	-2.201635750178	protein_codin	XM_022999375.1	uncharacterized LOC111376414
615	gene-LOC11137644	22.7491480745333	13.916302380033	0.7200937662	0.9532953393	0.709036587329	protein_codin	XM_022999404.1	7-deoxyloganetic acid glucosyltransferase-like
616	gene-LOC11137647	6.87246904836667	14.663015566133	-0.7682852387	0.9542215726	-1.093281426350	protein_codin	XM_022999439.1	probable calcium-binding protein CML35
617	gene-LOC11137648	4.37068472446667	0.5	1.4824532551	0.9678217932	3.127859314327	protein_codin	XM_022999447.1	serine/threonine-protein phosphatase PP-X isozyme 2-like
618	gene-LOC11137653	2.00974990186667	0.5	0.7043739485	0.9528997665	2.007015980117	protein_codin	XM_022999492.1	probable galacturonosyltransferase 7%2C transcript variant X1
619	gene-LOC11137655	4.94996721503333	0.8599173533333	1.0795146621	0.9681091956	2.525149055712	protein_codin	XM_022999524.1	probable lysophospholipase BODYGUARD 1
620	gene-LOC11137657	0.5	2.1928344418	-0.7541193844	0.9529778007	-2.132796893174	protein_codin	XM_022999540.1	15-cis-zeta-carotene isomerase%2C chloroplastic-like
621	gene-LOC11137657	3.46948953186667	0.5	1.0971500597	0.9713315259	2.794723413806	protein_codin	XM_022999544.1	histone-lysine N-methyltransferase ATX2-like
622	gene-LOC11137658	2.66845099056667	0.5	0.8904698014	0.9635352514	2.416002514674	protein_codin	XM_022999551.1	uncharacterized WD repeat-containing protein C2A9.03-like%2C transcript variant X5
623	gene-LOC11137663	0.662683877666667	2.9329497934333	-0.8590891664	0.9516528053	-2.145959649239	protein_codin	XM_022999607.1	actin-related protein 2/3 complex subunit 4
624	gene-LOC11137666	5.0422943958	0.5	1.3707961626	0.9544641904	3.334080352817	protein_codin	XM_022999635.1	8-hydroxygeraniol dehydrogenase-like
625	gene-LOC11137667	2.5483395424	0.5	0.7722236578	0.9560425242	2.349557516016	protein_codin	XM_022999642.1	CO(2)-response secreted protease-like
626	gene-LOC11137669	7.3361272801	0.5	1.5475121741	0.9634903369	3.875018669573	protein_codin	XM_022999670.1	protein IQ-DOMAIN 1
627	gene-LOC11137671	9.9054051704	28.9296912103	-1.2675177279	0.9761192046	-1.546263030923	protein_codin	XM_022999683.1	E3 ubiquitin-protein ligase ATL23-like
628	gene-LOC11137671	3.34937808373333	0.5	1.2130486363	0.9832369298	2.743893239209	protein_codin	XM_022999695.1	mechanosensitive ion channel protein 6-like
629	gene-LOC11137672	0.5	2.1928344418	-0.7541193844	0.9529778007	-2.132796893174	protein_codin	XM_022999701.1	putative clathrin assembly protein At2g01600%2C transcript variant X2
630	gene-LOC11137675	0.5	2.1928344418	-0.7541193844	0.9529778007	-2.132796893174	protein_codin	XM_022999731.1	pentatricopeptide repeat-containing protein At1g31790
631	gene-LOC11137675	9.54827035866667	2.7321736790666	0.8237805412	0.9602958009	1.805190220902	protein_codin	XM_022999736.1	cryptochrome DASH%2C chloroplastic/mitochondrial%2C transcript variant X4
632	gene-LOC11137677	2.0762599173	0.5	0.6996756982	0.9526031653	2.053987059267	protein_codin	XM_022999775.1	uncharacterized LOC111376779
633	gene-LOC11137678	3.42978023283333	10.8794072482	-0.8273164745	0.9593249824	-1.665411913069	protein_codin	XM_022999782.1	guanosine nucleotide diphosphate dissociation inhibitor At5g09550
634	gene-LOC11137678	2.18534236223333	0.5	0.9277477865	0.9709477825	2.127859314327	protein_codin	XM_022999783.1	beta-ureidopropionase
635	gene-LOC11137678	3.72489221846667	0.7331400576333	0.9378111554	0.9733089539	2.345037941687	protein_codin	XM_022999785.1	nicotinamidase 1-like%2C transcript variant X1

636	gene-LOC11137678	6.73944901753333	0.9729997351	1.1531146851	0.982986750	2.792119331605	protein_codin	XM_022999790.1	heatshock 70 kDa protein%2C mitochondrial-like
637	gene-LOC11137678	12.4814414576333	5.1785018195333	0.6961560942	0.952305569	1.269177877292	protein_codin	XM_022999792.1	K(+) efflux antiporter 3%2C chloroplastic-like
638	gene-LOC11137681	8.9838635528	15.2500252763	-0.839384195	0.957599757	-0.76340371256	protein_codin	XM_022999813.1	dihydrolipoyl dehydrogenase%2C mitochondrial-like
639	gene-LOC11137684	2.69426815593333	0.5	0.8963939323	0.964311812	2.429893446933	protein_codin	XM_022999841.1	uncharacterized LOC111376849%2C transcriptvariant X2
640	gene-LOC11137685	0.5	2.1928344418	-0.754119384	0.952977800	-2.13279689317	protein_codin	XM_022999865.1	mitochondrial importinner membrane translocase subunitTIM50-like
641	gene-LOC11137686	1232.29262295457	1113.4568115392	1.2087483028	0.982934974	0.146299281949	protein_codin	XM_022999868.1	fasciclin-like arabinogalactan protein 1
642	gene-LOC11137686	13.3480939859333	7.3587028294666	0.6849490111	0.950922150	0.859110370140	protein_codin	XM_022999870.1	uncharacterized LOC111376865
643	gene-LOC11137689	3.0403739644	7.8648723156333	-0.817723120	0.959470757	-1.37117455869	protein_codin	XM_022999898.1	two-componentresponse regulator-like APRR7
644	gene-LOC11137690	6.17893747806667	0.5	1.2726077460	0.984195848	3.627358775522	protein_codin	XM_022999905.1	hydroxyproline O-galactosyltransferase GALT6-like
645	gene-LOC11137692	1.96905705183333	0.5	0.6815674662	0.950366313	1.977504912765	protein_codin	XM_022999932.1	serine/threonine protein phosphatase 2A regulatory subunitB"beta-like%2C transcriptvariant X1
646	gene-LOC11137692	5.08298724586667	1.1329467819	0.7772862194	0.956710353	2.165596516015	protein_codin	XM_022999941.1	phosphoenolpyruvate carboxylase kinase 2-like
647	gene-LOC11137692	3.5903842363	0.6531665342333	0.7010809021	0.952699183	2.458615467169	protein_codin	XM_022999942.1	zinc finger protein ZAT11-like
648	gene-LOC11137697	0.992034422	4.0795914893	-0.770124906	0.954376383	-2.03996260908	protein_codin	XM_022999983.1	katanin p60 ATPase-containing subunit A1-like
649	gene-LOC11137697	11.9147634441333	3.732563242	1.0626298259	0.967057639	1.674511698808	protein_codin	XM_022999992.1	50S ribosomal protein L12%2C chloroplastic-like
650	gene-LOC11137706	5.5660958742	1.55316804	0.7165678073	0.953266924	1.841451832938	protein_codin	XM_023000113.1	uncharacterized LOC111377065
651	gene-LOC11137709	4.0552263138	1.0801683049	0.7004844621	0.952658300	1.908526309122	protein_codin	XM_023000143.1	nuclear pore complex protein NUP1-like
652	gene-LOC11137716	5.87697957296667	1.2861133161333	1.0357363607	0.968135142	2.192057123446	protein_codin	XM_023000222.1	ABC transporter G family member 14-like
653	gene-LOC11137716	131.0859670526	77.129652460866	0.9264297680	0.970622025	0.765155735811	lncRNA	XR_002699667.1	uncharacterized LOC111377168%2C transcriptvariant X4
654	gene-LOC11137717	3.74563027163333	0.5	1.0271752284	0.968858085	2.905208499258	protein_codin	XM_023000231.1	40S ribosomal protein S26-1-like
655	gene-LOC11137723	60.7168822977333	129.86197308013	-2.749933962	1	-1.09680941649	protein_codin	XM_023000291.1	granule-bound starch synthase 1%2C chloroplastic/amyloplastic-like
656	gene-LOC11137723	10.7003810485333	3.9196444425333	0.9098884638	0.966712532	1.448867476863	protein_codin	XM_023000295.1	uncharacterized LOC111377235
657	gene-LOC11137723	3.02648183073333	0.7067508191	0.7929120671	0.958639195	2.098368134753	protein_codin	XM_023000298.1	uncharacterized protein At2g29880-like
658	gene-LOC11137728	0.629428869966667	2.9593390319666	-0.959475580	0.951599362	-2.23315972880	protein_codin	XM_023000362.1	phosphatidate cytidylyltransferase 4%2C chloroplastic-like
659	gene-LOC11137730	6.57637351173333	2.1928344418	0.7626893115	0.954896930	1.584495347485	protein_codin	XM_023000381.1	uncharacterized LOC111377305%2C transcriptvariant X4
660	gene-LOC11137731	16.7103806523333	8.5573780871666	0.9173856934	0.968400291	0.965503858376	protein_codin	XM_023000394.1	rootphototropism protein 2-like
661	gene-LOC11137732	0.5	3.0468379831	-0.840950890	0.957251166	-2.60731278614	protein_codin	XM_023000403.1	GTPase-activating protein GYP1-like%2C transcriptvariant X2
662	gene-LOC11137734	0.938432989266667	4.3722296438	-0.962427457	0.953600101	-2.22004354281	protein_codin	XM_023000419.1	fructose-bisphosphate aldolase%2C cytoplasmic isozyme-like
663	gene-LOC11137739	0.5	2.9601448398666	-0.816059269	0.959408011	-2.56566776863	protein_codin	XM_023000485.1	zinc finger MYM-type protein 1-like
664	gene-LOC11137744	2.2716068798	0.5	0.7301479895	0.953318698	2.183713186235	protein_codin	XM_023000543.1	glucan endo-1%2C3-beta-glucosidase 3-like
665	gene-LOC11137747	3.15343919836667	11.198434641266	-1.018611392	0.984070383	-1.82829905920	protein_codin	XM_023000578.1	PRA1 family protein F4-like
666	gene-LOC11137748	26.5760765237	12.2090402125	1.2747665622	0.983905315	1.122178342136	protein_codin	XM_023000594.1	auxin-responsive protein IAA16-like
667	gene-LOC11137749	4.94292100083333	1.4664748967666	0.7031277652	0.952829690	1.753011473961	protein_codin	XM_023000604.1	uncharacterized LOC111377493%2C transcriptvariant X1
668	gene-LOC11137750	2.37235545393333	0.5	0.8089369960	0.959857263	2.246320187458	protein_codin	XM_023000622.1	serine/threonine-protein kinase AFC1-like
669	gene-LOC11137751	3.34937808373333	0.9729997351	0.6939564371	0.952089784	1.783381921865	protein_codin	XM_023000627.1	two-componentresponse regulator-like APRR7%2C transcriptvariant X1
670	gene-LOC11137754	413.3605495573	465.71773173006	-2.482659184	0.999999999	-0.17205510543	lncRNA	XR_002699744.1	uncharacterized LOC111377545
671	gene-LOC11137754	0.5	3.3206732195333	-0.770359075	0.954396269	-2.73147575724	protein_codin	XR_002699745.1	factor of DNA methylation 3-like%2C transcriptvariant X2
672	gene-LOC11137755	0.938432989266667	4.3722296438	-0.962427457	0.953600101	-2.22004354281	protein_codin	XM_023000682.1	dynamin-related protein 1E-like
673	gene-LOC11137758	4.28382828403333	15.6497102152	-1.161679985	0.982084746	-1.86916338673	protein_codin	XM_023000718.1	probable inactive purple acid phosphatase 9
674	gene-LOC11137761	26.1620855206333	14.5559687818	0.8718739984	0.961912538	0.845866688259	protein_codin	XM_023000740.1	7-deoxyloganetic acid glucosyltransferase-like%2C transcriptvariant X2
675	gene-LOC11137768	2.66845099056667	0.5	0.8904698014	0.963535251	2.416002514674	lncRNA	XR_002699760.1	uncharacterized LOC111377682
676	gene-LOC11137768	4.85665648326667	1.2333348391333	0.7762228576	0.956569502	1.977398915106	protein_codin	XR_002699762.1	sucrose nonfermenting 4-like protein%2C transcriptvariant X5
677	gene-LOC11137769	0.5	3.5726161952333	-1.317115159	0.971158587	-2.83698093408	protein_codin	XM_023000826.1	bax inhibitor 1-like
678	gene-LOC11137772	5.25897133436667	1.8126973361	0.8350956125	0.960474517	1.536642572859	lncRNA	XR_002699768.1	uncharacterized LOC111377722
679	gene-LOC11137773	4.37068472446667	0.9729997351	0.8966970601	0.964355734	2.167347996983	protein_codin	XM_023000861.1	ABC transporter B family member 15-like
680	gene-LOC11137773	0.5	4.1051140272333	-1.144674813	0.979132492	-3.03742229611	protein_codin	XM_023000862.1	uncharacterized LOC111377734
681	gene-LOC11137773	1.6600529325	8.6389023335666	-1.149429990	0.979969244	-2.37961876982	protein_codin	XM_023000894.1	GDSL esterase/lipase CPRD49-like%2C transcriptvariant X1
682	gene-LOC11137774	1.85599181786667	0.5	0.9346285653	0.972602995	1.892190350363	protein_codin	XM_023000873.1	probable serine/threonine-protein kinase PBL19%2C transcriptvariant X2
683	gene-LOC11137775	22.8941483629	40.095107472	-1.034002126	0.984592941	-0.80844730600	protein_codin	XM_023000881.1	uncharacterized LOC111377757
684	gene-LOC11137777	3.7585388543	0.5	1.0886615056	0.969514727	2.910171917927	protein_codin	XM_023000898.1	F-box/kelch-repeatprotein SKIP30-like

685	gene-LOC11137777	2.08916849996667	0.5	0.7050067510	0.952935613	2.062928856341	protein_codin	XM_023000903.1	auxin-responsive protein SAUR32-like
686	gene-LOC11137780	0.5	3.6865043849	-1.033761570	0.984607952	-2.88225347297	protein_codin	XM_023000929.1	putative ubiquitin-conjugating enzyme E2 38
687	gene-LOC11137780	6.09365651153333	2.1861148219	0.8277700500	0.960359201	1.478939002127	protein_codin	XM_023000931.1	transcription factor bHLH162-like
688	gene-LOC11137782	1.96905705183333	0.5	0.6815674662	0.950366313	1.977504912765	protein_codin	XM_023000956.1	sialyltransferase-like protein 2
689	gene-LOC11137784	3.15343919836667	0.8599173533333	0.6994403851	0.952586546	1.874656202642	protein_codin	XM_023000977.1	serine/threonine-protein phosphatase PP1 isozyme 3%2C transcript variant X2
690	gene-LOC11137784	74.6611015609667	93.838839470366	-0.807902519	0.958755272	-0.32982837675	protein_codin	XM_023000979.1	peroxisomal nicotinamide adenine dinucleotide carrier
691	gene-LOC11137786	0.5	3.1991987094666	-1.273863894	0.974570555	-2.67771060428	protein_codin	XM_023000996.1	uncharacterized LOC111377860%2C transcript variant X1
692	gene-LOC11137796	5.4123377902	13.383804548	-0.817093707	0.959448366	-1.30616449458	protein_codin	XM_023001121.1	aspartyl protease family protein At5g10770-like%2C transcript variant X2
693	gene-LOC11137803	78.6314385364	105.78578159163	-0.964171774	0.954812057	-0.42796757799	protein_codin	XM_023001197.1	cysteine protease RD19A-like
694	gene-LOC11137805	15.7930773443667	4.1586983121333	1.1725942607	0.982917758	1.925088378214	protein_codin	XM_023001217.1	fatty-acid-binding protein 1-like%2C transcript variant X2
695	gene-LOC11137806	3.64547362036667	0.6531665342333	1.0253687609	0.969026422	2.480583482136	protein_codin	XM_023001237.1	uncharacterized LOC111378069
696	gene-LOC11137808	0.5	3.5317871276333	-0.794464778	0.957058227	-2.82039838965	protein_codin	XM_023001249.1	V-type proton ATPase subunit F-like
697	gene-LOC11137808	0.5	3.6865043849	-1.033761570	0.984607952	-2.88225347297	protein_codin	XM_023001258.1	uncharacterized LOC111378089
698	gene-LOC11137812	2.7775334355	0.5	1.1021262443	0.972581813	2.473804278649	protein_codin	XM_023001291.1	uncharacterized LOC111378120%2C transcript variant X2
699	gene-LOC11137815	351.037232048033	382.0917425233	-0.741088695	0.951338437	-0.12229502445	protein_codin	XM_023001322.1	probable plastid-lipid-associated protein 8%2C chloroplastic
700	gene-LOC11137817	0.5	2.9125352596666	-0.850415837	0.954632262	-2.54227551539	protein_codin	XM_023001336.1	alpha-L-fucosidase 1-like%2C transcript variant X2
701	gene-LOC11137818	0.5	2.2456129188	-0.773028660	0.954629033	-2.16710926867	protein_codin	XM_023001349.1	exocyst complex component EXO70A1-like
702	gene-LOC11137821	57.9080057307	25.566455522	1.7498613485	0.977137639	1.179510650991	protein_codin	XM_023001378.1	probable WRKY transcription factor 11
703	gene-LOC11137821	134.502301725933	87.1732046495	1.6393044547	0.980514065	0.625674209892	protein_codin	XM_023001383.1	cytoplasmic 60S subunit biogenesis factor REI1 homolog 1-like
704	gene-LOC11137822	2.57415670776667	0.5	0.7724889366	0.956076955	2.364099883642	protein_codin	XM_023001390.1	uncharacterized protein At4g22758-like
705	gene-LOC11137824	10.0952813925667	21.8058914257	-1.049762483	0.982170477	-1.11103684461	protein_codin	XM_023001410.1	erythromycin 3''-O-methyltransferase-like%2C transcript variant X1
706	gene-LOC11137824	10.1210985579333	5.4125085777333	0.6897298935	0.951594172	0.902996579297	protein_codin	XM_023001414.1	uncharacterized LOC111378242%2C transcript variant X3
707	gene-LOC11137825	4.08690584763333	0.9398908767333	0.6851016772	0.950946787	2.120443835798	protein_codin	XM_023001438.1	pathogenesis-related homeodomain protein-like%2C transcript variant X2
708	gene-LOC11137830	1.06786185923333	3.8916435882333	-0.736003274	0.950506351	-1.86565455942	protein_codin	XM_023001477.1	uncharacterized LOC111378303
709	gene-LOC11137833	4.8169471842	0.9729997351	0.7713183864	0.955925614	2.307607788123	protein_codin	XM_023001519.1	uncharacterized LOC111378338
710	gene-LOC11137833	3.65650262356667	0.5	1.0146125804	0.970186963	2.870464397170	protein_codin	XM_023001522.1	MADS-box protein 04g005320-like%2C transcript variant X3
711	gene-LOC11137834	0.5	3.2051734141666	-0.858177733	0.951982431	-2.68040241518	protein_codin	XM_023001563.1	cytochrome P450 77A2-like
712	gene-LOC11137838	4.00162488106667	0.9729997351	0.8273783742	0.960353337	2.040074615619	protein_codin	XM_023001573.1	regulator of nonsense transcripts 1 homolog
713	gene-LOC11137838	3.06619112976667	0.5	0.9928071491	0.973238823	2.616447629603	protein_codin	XM_023001577.1	E3 ubiquitin-protein ligase AIP2
714	gene-LOC11137841	0.5	4.4333393564333	-1.092915347	0.974338586	-3.14839379992	lncRNA	XR_002699858.1	uncharacterized LOC111378418
715	gene-LOC11137844	10.2198800300333	3.2850860448666	0.8446902636	0.960686802	1.637375196692	protein_codin	XM_023001647.1	inactive glucose-1-phosphate adenylyltransferase small subunit 2%2C chloroplastic
716	gene-LOC11137845	2.00974990186667	0.5	0.7043739485	0.952899766	2.007015980117	protein_codin	XM_023001654.1	protein PEROXIN-4-like
717	gene-LOC11137846	2.66845099056667	0.5	0.8904698014	0.963535251	2.416002514674	protein_codin	XM_023001662.1	ELMO domain-containing protein C-like%2C transcript variant X3
718	gene-LOC11137846	2.25224400576667	0.5	0.7304862915	0.953319521	2.171363135961	protein_codin	XM_023001665.1	pentatricopeptide repeat-containing protein At1g11290%2C chloroplastic
719	gene-LOC11137848	0.5	2.2456129188	-0.773028660	0.954629033	-2.16710926867	protein_codin	XM_023001688.1	N-terminal acetyltransferase B complex auxiliary subunit NAA25-like
720	gene-LOC11137849	10.7852703869667	20.340344122266	-0.738942800	0.951001866	-0.91528174187	protein_codin	XM_023001704.1	calmodulin-binding receptor-like cytoplasmic kinase 2
721	gene-LOC11137859	2.60194097513333	0.5	0.7922052050	0.958561254	2.379588234981	protein_codin	XM_023001825.1	uncharacterized LOC111378591
722	gene-LOC11137862	2.08916849996667	0.5	0.7050067510	0.952935613	2.062928856341	protein_codin	XM_023001852.1	kinesin-like protein KIN-14F
723	gene-LOC11137862	20.7400939063333	9.8638450443666	0.9044352571	0.965634857	1.072200385276	protein_codin	XM_023001853.1	heat shock cognate protein 80
724	gene-LOC11137862	0.5	2.9329497934333	-1.062324105	0.979286965	-2.55235237508	protein_codin	XM_023001855.1	calcineurin B-like protein 3%2C transcript variant X2
725	gene-LOC11137870	0.5	2.0865325725666	-0.745004129	0.951905606	-2.06110744248	protein_codin	XM_023001932.1	tobamovirus multiplication protein 3-like%2C transcript variant X2
726	gene-LOC11137871	82.3704717682	62.381767631366	0.9927144991	0.973252861	0.401002818555	protein_codin	XM_023001952.1	inorganic phosphate transporter 1-4-like%2C transcript variant X1
727	gene-LOC11137873	0.6358831613	3.5726161952333	-1.097629595	0.974163926	-2.49014732336	protein_codin	XM_023001972.1	uncharacterized LOC111378738
728	gene-LOC11137875	0.5	3.5462269567333	-1.112668084	0.974659693	-2.82628487094	protein_codin	XM_023001993.1	protein FATTY ACID EXPORT 5-like%2C transcript variant X1
729	gene-LOC11137877	0.798567038966667	3.2850860448666	-0.754866035	0.953050011	-2.04044572819	protein_codin	XM_023002007.1	UPF0481 protein At3g47200-like
730	gene-LOC11137878	3.52348259276667	0.5	0.9646329821	0.976191565	2.817002086672	protein_codin	XM_023002036.1	fasciclin-like arabinogalactan protein 16
731	gene-LOC11137879	43.4537157656333	32.470216619266	0.7562687710	0.954277215	0.420362539103	protein_codin	XM_023002047.1	dihydroneopterin aldolase 2-like%2C transcript variant X3
732	gene-LOC11137880	2.3058454385	0.5	0.7515754292	0.953930229	2.205295811956	protein_codin	XM_023002056.1	beta-glucosidase BoGH3B-like
733	gene-LOC11137888	2.03556706723333	0.5	0.7088504665	0.953093597	2.025430755730	protein_codin	XM_023002165.1	uncharacterized LOC111378881

734	gene-LOC11137889	1.2305457369	5.4916762932666	-1.098139389	0.974155992	-2.15794830751	protein_codin	XM_023002174.1	ethylene-responsive transcription factor CRF6-like
735	gene-LOC11137890	0.5	2.1928344418	-0.754119384	0.952977800	-2.13279689317	protein_codin	XM_023002181.1	SNF1-related protein kinase catalytic subunit alpha KIN10-like
736	gene-LOC11137897	2.08916849996667	0.5	0.7050067510	0.952935613	2.062928856341	protein_codin	XM_023002281.1	uncharacterized LOC111378976%2C transcript variant X1
737	gene-LOC11137897	2.15854164583333	0.5	0.9576639473	0.976078277	2.110056927711	protein_codin	XM_023002285.1	zinc finger protein CONSTANS-LIKE 5-like
738	gene-LOC11137900	1.96905705183333	0.5	0.6815674662	0.950366313	1.977504912765	protein_codin	XM_023002311.1	nipped-B-like protein B
739	gene-LOC11137903	2.08916849996667	0.5	0.7050067510	0.952935613	2.062928856341	protein_codin	XM_023002362.1	gamma aminobutyrate transaminase 1%2C mitochondrial-like
740	gene-LOC11137914	7.78110208326667	2.8257812237	0.7523810913	0.953983835	1.461324732729	protein_codin	XM_023002478.1	protein ACTIVITY OF BC1 COMPLEX KINASE 8%2C chloroplastic-like
741	gene-LOC11137915	253.9408330526	340.36134922123	-1.106022730	0.974257244	-0.42257482125	protein_codin	XM_023002489.1	probable ribosome biogenesis protein RLP24
742	gene-LOC11137916	0.927995908933333	6.7702032889666	-1.222653178	0.984737622	-2.86700880390	protein_codin	XM_023002498.1	uncharacterized LOC111379162
743	gene-LOC11137917	43.0055414297333	15.675354538533	2.0918093236	1	1.456024494945	protein_codin	XM_023002519.1	scarecrow-like protein 15
744	gene-LOC11137918	12.1959832960667	5.1454538538666	0.8063302465	0.959721986	1.245035838998	lncRNA	XR_002700026.1	uncharacterized LOC111379185%2C transcript variant X16
745	gene-LOC11137918	6.22159743016667	0.5	1.1987321871	0.982491268	3.637285047991	protein_codin	XM_023002534.1	basic endochitinase-like
746	gene-LOC11137919	4.26348185896667	1.0801683049	0.7400561737	0.953433387	1.980775996598	protein_codin	XM_023002542.1	uncharacterized LOC111379196
747	gene-LOC11137920	0.5	2.5127285353666	-0.801158618	0.957947684	-2.32925481692	protein_codin	XM_023002553.1	uncharacterized LOC111379207%2C transcript variant X1
748	gene-LOC11137921	2.5612481251	0.5	0.8691339282	0.961743624	2.356847023146	protein_codin	XM_023002558.1	ankyrin repeat-containing protein At5g02620-like
749	gene-LOC11137923	0.5	2.1928344418	-0.754119384	0.952977800	-2.13279689317	protein_codin	XM_023002582.1	salicylic acid-binding protein 2-like
750	gene-LOC11137927	2.2586982971	0.5	0.7919045808	0.958528247	2.175491577350	protein_codin	XM_023002630.1	uncharacterized LOC111379272
751	gene-LOC11137929	3.7655850685	1.1797505542	0.6888363463	0.951478439	1.674392188303	protein_codin	XM_023002659.1	trafficking protein particle complex subunit 11
752	gene-LOC11137931	17.4753010554667	30.284101797333	-1.021822848	0.984480285	-0.79324331300	protein_codin	XM_023002673.1	3-oxo-Delta(4%2C5)-steroid 5-beta-reductase-like
753	gene-LOC11137934	14.3135832122	4.2914503125333	0.9973704614	0.972548642	1.737847676778	protein_codin	XM_023002698.1	U-box domain-containing protein 19-like
754	gene-LOC11137934	1.8291911015	6.1329543107666	-0.770631674	0.954419469	-1.74537639727	protein_codin	XM_023002704.1	probable strigolactone esterase DAD2
755	gene-LOC11137935	3.66138144103333	0.5	0.8085780699	0.959839781	2.872388080777	protein_codin	XM_023002709.1	mitogen-activated protein kinase homolog MMK1-like
756	gene-LOC11137935	3.2725670955	0.5	0.9406593252	0.973894339	2.710422771193	protein_codin	XM_023002710.1	AP2/ERF and B3 domain-containing transcription factor RAV1-like
757	gene-LOC11137935	15.6751818779	6.9317010587666	0.9311987150	0.971792156	1.177200840292	protein_codin	XM_023002717.1	probable galacturonosyltransferase-like 3
758	gene-LOC11137936	4.96287579773333	0.5	1.6625279099	0.972751841	3.311176349812	protein_codin	XM_023002727.1	UPF0481 protein At3g47200-like%2C transcript variant X1
759	gene-LOC11137938	5.5867464048	0.5	1.1445687614	0.982341427	3.482008334549	protein_codin	XM_023002903.1	protein BTR1-like%2C transcript variant X3
760	gene-LOC11137939	2.57415670776667	0.5	0.7724889366	0.956076955	2.364099883642	protein_codin	XM_023002754.1	uncharacterized LOC111379397
761	gene-LOC11137942	12.4940459347333	4.4845062177333	0.8953756670	0.964164781	1.478219693126	protein_codin	XM_023002789.1	photosynthetic NDH subunit of subcomplex B 5%2C chloroplastic%2C transcript variant X1
762	gene-LOC11137944	1.85599181786667	8.3785063368666	-0.982057074	0.967590240	-2.17450272262	protein_codin	XM_023002817.1	chalcone synthase J-like
763	gene-LOC11137947	2.57474863063333	0.5	0.7362066657	0.953362977	2.364431590726	protein_codin	XM_023002844.1	ubiquitin-conjugating enzyme E2 32-like
764	gene-LOC11137948	3.16634778103333	0.5	0.9642200631	0.976196617	2.662819724968	protein_codin	XM_023002866.1	uncharacterized LOC111379489%2C transcript variant X1
765	gene-LOC11137950	4.51153422576667	1.2861133161333	0.6892623003	0.951533510	1.810600370076	protein_codin	XM_023002880.1	protein CDI-like
766	gene-LOC11137950	5.6848873691	13.890718949366	-0.805454284	0.958480848	-1.28891760023	protein_codin	XM_023002888.1	glucan endo-1%2C3-beta-glucosidase 14%2C transcript variant X1
767	gene-LOC11137950	0.5	3.2595026142	-1.004866787	0.980330375	-2.70465183231	protein_codin	XM_023002893.1	ferredoxin%2C root R-B1%2C transcript variant X3
768	gene-LOC11137951	2.0623677836	0.5	0.7505681576	0.953863717	2.044301632542	protein_codin	XR_002700055.1	protein-lysine methyltransferase METTL21D%2C transcript variant X2
769	gene-LOC11137951	0.5	2.4863392968666	-0.852188209	0.954055966	-2.31402318637	protein_codin	XM_023002907.1	DDB1- and CUL4-associated factor 13
770	gene-LOC11137954	3.2457663791	0.7067508191	0.7075837095	0.953048176	2.199285607391	protein_codin	XM_023002941.1	pentatricopeptide repeat-containing protein At3g20730-like%2C transcript variant X1
771	gene-LOC11137954	3.7784936512	0.5	1.1979156329	0.982471102	2.917811198712	lncRNA	XR_002700062.1	uncharacterized LOC111379549
772	gene-LOC11137962	8.4646367863	2.485533489	0.7985809351	0.959177970	1.767892622404	protein_codin	XM_023003037.1	beta-glucuronosyltransferase GlcAT14B-like
773	gene-LOC11137962	21.4488927895333	7.4651264840666	1.1153502047	0.976233342	1.522664566745	protein_codin	XM_023003042.1	DUF21 domain-containing protein At4g14240-like%2C transcript variant X2
774	gene-LOC11137964	3.10688397983333	11.9429130819	-1.127681717	0.976373101	-1.94261450454	protein_codin	XM_023003073.1	thioredoxin-like fold domain-containing protein MRL7L%2C chloroplastic%2C transcript variant X2
775	gene-LOC11137967	61.2681316412667	16.658886848833	1.9652487175	0.999999999	1.878844854640	lncRNA	XR_002700081.1	uncharacterized LOC111379678
776	gene-LOC11137970	2.57415670776667	0.5	0.7724889366	0.956076955	2.364099883642	protein_codin	XM_023003143.1	glycerol-3-phosphate acyltransferase 9
777	gene-LOC11137973	10.3913769292	3.9452278731333	0.9104908595	0.966840928	1.397206297321	protein_codin	XM_023003176.1	DEAD-box ATP-dependent RNA helicase 13%2C transcript variant X1
778	gene-LOC11137975	960.979184473467	1699.0911470586	-4.928327932	0.999999999	-0.82218616079	protein_codin	XM_023003194.1	uncharacterized LOC111379753
779	gene-LOC11137978	7.3028722724	2.1392501568666	0.7238798104	0.953307641	1.771358801611	protein_codin	XM_023003219.1	21 kDa protein-like
780	gene-LOC11137981	3.15343919836667	0.5	0.9662992048	0.976157527	2.656926116795	protein_codin	XM_023003253.1	mitogen-activated protein kinase kinase kinase 17-like
781	gene-LOC11137985	85.9430363077333	116.0957653744	-0.785391767	0.955901803	-0.43386269791	protein_codin	XM_023003287.1	myosin-12-like%2C transcript variant X1
782	gene-LOC11137991	61.9196437672333	40.5288601691	0.7295902091	0.953317347	0.611447569772	protein_codin	XM_023003358.1	glucomannan 4-beta-mannosyltransferase 2-like%2C transcript variant X3

AQRNA_Control_vs_AQRNA_G7

783	gene-LOC11137991	0.9255244066	5.8658995869	-1.110000031	0.974466192	-2.66400943470	protein_codin	XM_023003364.1	serine carboxypeptidase-like 51
784	gene-LOC11137991	1.85599181786667	0.5	0.9346285653	0.972602995	1.892190350363	protein_codin	XM_023003365.1	UDP-D-apiose/UDP-D-xylose synthase 2-like
785	gene-LOC11137992	3.0532825471	0.5	1.1838123257	0.982546110	2.610361100988	protein_codin	XM_023003366.1	uncharacterized LOC111379920
786	gene-LOC11137995	0.5	2.5127285353666	-0.801158618	0.957947684	-2.32925481692	protein_codin	XM_023003395.1	zinc finger protein 1-like
787	gene-LOC11137996	0.938432989266667	3.6253946722666	-0.775768242	0.954880325	-1.94981242490	protein_codin	XM_023003401.1	ankyrin repeat-containing protein At5g02620-like%2C transcript variant X2
788	gene-LOC11137996	0.5	1.8126973361	-0.775838238	0.954886876	-1.85813806012	protein_codin	XM_023003405.1	UDP-glucuronic acid decarboxylase 1-like
789	gene-LOC11137998	6.16016652693333	2.4599500583333	0.6958383413	0.952274171	1.324340325216	protein_codin	XM_023003439.1	uncharacterized LOC111379988%2C transcript variant X1
790	gene-LOC11138002	3.0532825471	8.8516278574	-0.989785012	0.972652075	-1.53558169723	protein_codin	XM_023003488.1	acidic leucine-rich nuclear phosphoprotein 32-related protein
791	gene-LOC11138005	2.03556706723333	9.3313472124	-1.240000285	0.982497220	-2.19665462934	protein_codin	XM_023003522.1	nitrate regulatory gene2 protein-like
792	gene-LOC11138006	12.1175482490333	24.953984166566	-1.468745088	0.999906929	-1.04217234845	protein_codin	XM_023003532.1	F-box protein PP2-B11-like%2C transcript variant X2
793	gene-LOC11138007	0.5	2.5127285353666	-0.801158618	0.957947684	-2.32925481692	protein_codin	XM_023003538.1	polyadenylate-binding protein-interacting protein 12-like
794	gene-LOC11138011	484.374348823767	562.5858345025	-0.763984102	0.953864174	-0.21595076134	protein_codin	XM_023003592.1	BOI-related E3 ubiquitin-protein ligase 1-like%2C transcript variant X3
795	gene-LOC11138012	260.999745919167	333.8216545816	-1.351749089	0.976915369	-0.35502914116	lncRNA	XR_002700141.1	uncharacterized LOC111380129
796	gene-LOC11138013	1.95614846913333	0.5	0.6824614660	0.950524364	1.968015873129	protein_codin	XM_023003623.1	B3 domain-containing protein Os01g0234100-like
797	gene-LOC11138015	73.0876172516333	144.0883171812	-1.850242563	0.962732742	-0.97925445937	protein_codin	XM_023003644.1	subtilisin-like protease SBT4.14
798	gene-LOC11138017	2.7507327191	0.6531665342333	0.8776423496	0.962314421	2.074293184173	protein_codin	XM_023003667.1	enoyl-[acyl-carrier-protein] reductase [NADH]%2C chloroplastic%2C transcript variant X2
799	gene-LOC11138017	5.7643059672	1.4995837551	0.8420285991	0.960617874	1.942584813061	protein_codin	XR_002700144.1	uncharacterized LOC111380178%2C transcript variant X2
800	gene-LOC11138019	9.1985733893	3.2067850299	0.7141876787	0.953231711	1.520282485834	protein_codin	XM_023003683.1	cysteine synthase%2C chloroplastic/chromoplastic-like%2C transcript variant X2
801	gene-LOC11138020	13.8330821937	3.3063672792	1.5244213780	0.968222280	2.064803747178	protein_codin	XM_023003702.1	casein kinase 1-like protein HD16
802	gene-LOC11138022	0.5	3.5726161952333	-1.317115159	0.971158587	-2.83698093408	protein_codin	XM_023003722.1	E3 ubiquitin-protein ligase TRIM39-like%2C transcript variant X1
803	gene-LOC11138022	2.7537319571	0.5	0.6975232012	0.952426558	2.461388137184	protein_codin	XM_023003726.1	equilibrative nucleotide transporter 1-like
804	gene-LOC11138024	0.629428869966667	5.2509499152	-1.189514131	0.985308951	-3.06046317771	protein_codin	XM_023003745.1	uncharacterized LOC111380240
805	gene-LOC11138024	0.5	2.8852793205666	-0.859212762	0.951607986	-2.52871099127	protein_codin	XM_023003758.1	thiamine phosphate phosphatase-like protein
806	gene-LOC11138027	1.85599181786667	0.5	0.9346285653	0.972602995	1.892190350363	protein_codin	XM_023003784.1	glycerol-3-phosphate 2-O-acyltransferase 6-like
807	gene-LOC11138029	11.9587110528333	1.2333348391333	1.5380586450	0.965706176	3.277425463199	lncRNA	XR_002700165.1	uncharacterized LOC111380290
808	gene-LOC11138029	0.5	3.3055614713666	-1.185795681	0.985069879	-2.72489534379	protein_codin	XM_023003825.1	putative disease resistance protein RGA3
809	gene-LOC11138030	2.18534236223333	0.6531665342333	0.7111472224	0.953165623	1.742336534623	protein_codin	XM_023003826.1	uncharacterized LOC111380300
810	gene-LOC11138033	3.09299184616667	0.7331400576333	0.8230451414	0.960282762	2.076842290736	protein_codin	XM_023003859.1	uncharacterized LOC111380331
811	gene-LOC11138033	0.938432989266667	4.700454973	-0.736468648	0.950588748	-2.32447477165	protein_codin	XM_023003865.1	vesicle-associated membrane protein 727-like%2C transcript variant X3
812	gene-LOC11138034	0.5	5.3928389591	-1.290892779	0.971515854	-3.43104495274	protein_codin	XM_023003873.1	uncharacterized LOC111380343
813	gene-LOC11138034	2.08916849996667	0.5	0.7050067510	0.952935613	2.062928856341	protein_codin	XM_023003905.1	probable small nuclear ribonucleoprotein F
814	gene-LOC11138034	0.5	3.1251389980666	-0.795942190	0.957256694	-2.64392035853	protein_codin	XM_023003876.1	iron-sulfur assembly protein IscA-like 3%2C mitochondrial%2C transcript variant X2
815	gene-LOC11138036	2.74786958863333	0.5	0.7977912973	0.959106193	2.458313536799	protein_codin	XM_023003896.1	protein NRT1/ PTR FAMILY 8.3-like
816	gene-LOC11138036	6.58282780306667	13.649125870733	-0.761945637	0.953693934	-1.05202919514	protein_codin	XM_023003902.1	probable manganese-transporting ATPase PDR2%2C transcript variant X1
817	gene-LOC11138038	6.74531138596667	2.1061412985333	0.7495127003	0.953804134	1.679282815985	protein_codin	XM_023003928.1	uncharacterized LOC111380384
818	gene-LOC11138038	6.3823142058	1.9257797179	0.8292648743	0.960381739	1.728636946645	protein_codin	XM_023003931.1	integrator complex subunit 3%2C transcript variant X3
819	gene-LOC11138042	14.5046918651	5.6523682552333	1.1121799725	0.975345368	1.359592281217	lncRNA	XR_002700187.1	uncharacterized LOC111380420
820	gene-LOC11138042	42.0449699585333	67.047319452166	-1.284084205	0.972485935	-0.67324644014	protein_codin	XM_023003981.1	COMPASS-like H3K4 histone methylase component WDR5A
821	gene-LOC11138046	0.5	2.3000030115333	-0.746106397	0.952051421	-2.20163575017	protein_codin	XM_023004050.1	ran-binding protein 1 homolog a-like
822	gene-LOC11138047	2.57415670776667	0.5	0.7724889366	0.956076955	2.364099883642	protein_codin	XM_023004060.1	WAT1-related protein At1g25270-like
823	gene-LOC11138049	3.0403739644	0.5	0.9963016230	0.972709801	2.604248785314	protein_codin	XM_023004072.1	pleiotropic drug resistance protein 1-like
824	gene-LOC11138049	7.01331854966667	0.6531665342333	1.3154618882	0.975789584	3.424574477978	protein_codin	XM_023004083.1	probable translation initiation factor eIF-2B subunit delta
825	gene-LOC11138051	6.4100984732	1.8458061945333	0.7732634000	0.956177927	1.796095438754	protein_codin	XM_023004097.1	UDP-glycosyltransferase 86A1-like
826	gene-LOC11138056	8.9218407267	2.1861148219	0.8599828205	0.961258737	2.028972215190	protein_codin	XM_023004181.1	ubiquitin fusion degradation protein 1 homolog%2C transcript variant X3
827	gene-LOC11138056	166.971161832467	293.14252260036	-1.639984026	0.999999825	-0.81200330597	protein_codin	XM_023004169.1	arogenate dehydratase/prephenate dehydratase 1%2C chloroplastic-like
828	gene-LOC11138056	4.00162488106667	11.224823879766	-1.022688889	0.984571194	-1.48803497082	protein_codin	XM_023004170.1	lactoylglutathione lyase GLX1-like%2C transcript variant X1
829	gene-LOC11138057	6.20085937696667	0.8599173533333	1.2191948540	0.983751165	2.850198258277	protein_codin	XM_023004175.1	protein indeterminate-domain 11-like
830	gene-LOC11138059	0.5	2.3000030115333	-0.746106397	0.952051421	-2.20163575017	protein_codin	XM_023004212.1	ultraviolet-B receptor UVR8-like%2C transcript variant X10
831	gene-LOC11138063	0.5	2.2456129188	-0.773028660	0.954629033	-2.16710926867	protein_codin	XM_023004252.1	aquaporin AQPAn.G-like

832	gene-LOC11138063	3.46948953186667	0.5	1.0971500597	0.971331525	2.794723413806	protein_codin	XM_023004249.1	serine/threonine-protein kinase STY8-like%2C transcript variant X1
833	gene-LOC11138067	17.6373930102333	8.4509544325333	0.9505060271	0.975467880	1.061451140781	protein_codin	XM_023004301.1	uncharacterized LOC111380671
834	gene-LOC11138070	32.4411310650333	46.677545538	-0.733846898	0.950125599	-0.52490458107	protein_codin	XM_023004340.1	formin-like protein 6
835	gene-LOC11138071	3.14053061566667	15.142917599233	-1.341109562	0.974464976	-2.26956295870	protein_codin	XM_023004350.1	GATA transcription factor 7-like
836	gene-LOC11138076	0.662683877666667	3.7069798114	-0.773405895	0.954662478	-2.48385153226	protein_codin	XM_023004399.1	probable protein phosphatase 2C 35
837	gene-LOC11138077	2.7775334355	0.5	1.1021262443	0.972581813	2.473804278649	protein_codin	XM_023004409.1	premnaspirodiene oxygenase-like
838	gene-LOC11138077	2.48143789886667	0.5	1.0599079230	0.967046652	2.311176349812	protein_codin	XM_023004411.1	premnaspirodiene oxygenase-like
839	gene-LOC11138078	6.2663858414	0.9729997351	1.1410416618	0.981912223	2.687122286443	protein_codin	XM_023004423.1	ribonuclease P protein subunit p25-like protein
840	gene-LOC11138080	31.1282160769333	20.3418339526	0.9207990172	0.969226026	0.613773146263	protein_codin	XM_023004451.1	BTB/POZ domain-containing protein At1g21780-like
841	gene-LOC11138081	1.06786185923333	4.3245591709	-0.750779574	0.952620688	-2.01782804825	protein_codin	XM_023004471.1	glucan endo-1%2C3-beta-D-glucosidase-like
842	gene-LOC11138082	10.8537475043667	2.8529762700666	1.2508965781	0.985728148	1.927653599803	protein_codin	XM_023004480.1	prolyl endopeptidase-like%2C transcript variant X2
843	gene-LOC11138086	2.00974990186667	8.1575713628666	-1.019437105	0.984195303	-2.02112372302	protein_codin	XM_023004526.1	transcription initiation factor TFIID subunit 9-like%2C transcript variant X2
844	gene-LOC11138089	3.0403739644	0.5	0.9963016230	0.972709801	2.604248785314	protein_codin	XM_023004554.1	pentatricopeptide repeat-containing protein At3g26540
845	gene-LOC11138092	1.96905705183333	0.5	0.6815674662	0.950366313	1.977504912765	protein_codin	XM_023004595.1	tyrosine--tRNA ligase 1%2C cytoplasmic%2C transcript variant X1
846	gene-LOC11138092	2.46109147383333	6.0513691716333	-0.749302821	0.952448549	-1.29796331996	protein_codin	XM_023004597.1	3-hydroxyisobutyryl-CoA hydrolase-like protein 5%2C transcript variant X1
847	gene-LOC11138093	2.44818289113333	0.5	0.9691321958	0.976035677	2.291711338371	protein_codin	XR_002700259.1	C2 domain-containing protein At1g53590-like%2C transcript variant X3
848	gene-LOC11138093	4.4214230267	0.7067508191	0.7724942677	0.956077647	2.645237217840	lncRNA	XR_002700260.1	uncharacterized LOC111380939
849	gene-LOC11138094	7.42396727153333	2.3000030115333	0.9187219525	0.968721046	1.690554600197	protein_codin	XM_023004617.1	uncharacterized GPI-anchored protein At4g28100-like
850	gene-LOC11138094	2.03556706723333	0.5	0.7088504665	0.953093597	2.025430755730	protein_codin	XM_023004622.1	Niemann-Pick C1 protein-like
851	gene-LOC11138095	1.06786185923333	3.8916435882333	-0.736003274	0.950506351	-1.86565455942	protein_codin	XM_023004631.1	autophagy-related protein 9-like%2C transcript variant X3
852	gene-LOC11138095	7.0752538532	2.8521704622	0.7383890285	0.953398614	1.310721703205	protein_codin	XM_023004638.1	major pollen allergen Ole e 10-like%2C transcript variant X1
853	gene-LOC11138098	469.944201059933	374.87273396123	1.1873035657	0.982469263	0.326088571922	lncRNA	XR_002700265.1	uncharacterized LOC111380984
854	gene-LOC11138100	3.7585388543	0.7331400576333	0.8637998104	0.961446635	2.358011178506	protein_codin	XM_023004690.1	mediator of RNA polymerase II transcription subunit 13%2C transcript variant X1
855	gene-LOC11138100	2.54794791426667	0.5	0.8179957286	0.960177446	2.349335785994	protein_codin	XM_023004689.1	L-aminoadipate-semialdehyde dehydrogenase-phosphopantetheinyl transferase-like%2C transcript variant X3
856	gene-LOC11138100	0.938432989266667	4.4266197365333	-0.782368896	0.955555077	-2.23787981052	protein_codin	XM_023004694.1	multiple myeloma tumor-associated protein 2 homolog
857	gene-LOC11138100	0.5	2.2464187266666	-0.794443451	0.957055382	-2.16762686744	protein_codin	XM_023004697.1	hippocampus abundant transcript-like protein 1%2C transcript variant X3
858	gene-LOC11138100	2.42138217476667	0.5	0.8950319652	0.964115330	2.275830802644	lncRNA	XR_002700266.1	uncharacterized LOC111381008
859	gene-LOC11138100	4.88991149096667	10.956963348066	-0.842359259	0.956916780	-1.16396776277	lncRNA	XR_002700267.1	uncharacterized LOC111381009
860	gene-LOC11138104	4.8685815149	0.5	1.2505834148	0.985729140	3.283501497254	protein_codin	XM_023004741.1	mental retardation GTPase activating protein homolog 4-like
861	gene-LOC11138106	6.1730751096	0.5	1.9825847996	1	3.625989345157	protein_codin	XM_023004775.1	uncharacterized LOC111381065%2C transcript variant X2
862	gene-LOC11138106	2.97001726753333	0.7067508191	0.7223405095	0.953304720	2.071197763603	protein_codin	XM_023004782.1	UDP-glycosyltransferase 73D1-like
863	gene-LOC11138107	3.8528331371	0.5	1.0550328079	0.967113968	2.945919705465	protein_codin	XR_002700271.1	telomere repeat-binding protein 2-like%2C transcript variant X3
864	gene-LOC11138108	1.68039935753333	5.5987839703333	-0.738091881	0.950860940	-1.73630937523	protein_codin	XM_023004800.1	probable WRKY transcription factor 53
865	gene-LOC11138108	78.6342627292333	59.396662490666	0.8250106378	0.960315948	0.404776196373	protein_codin	XM_023004808.1	uncharacterized LOC111381087%2C transcript variant X2
866	gene-LOC11138110	0.629428869966667	3.8916435882333	-1.030717957	0.984767231	-2.62826433132	protein_codin	XM_023004823.1	NDR1/HIN1-like protein 26
867	gene-LOC11138114	4.2773739927	0.5	1.4861639291	0.968161447	3.096725354940	protein_codin	XM_023004881.1	RING-H2 finger protein ATL57-like
868	gene-LOC11138114	3.36972450876667	0.5	1.1259951247	0.979031988	2.752630648949	protein_codin	XM_023004884.1	RING-H2 finger protein ATL57-like
869	gene-LOC11138114	7.96999475443333	3.1991987094666	0.8147992574	0.960087953	1.316868170397	protein_codin	XM_023004885.1	uncharacterized LOC111381144
870	gene-LOC11138118	4.36363851023333	0.5	1.1615441983	0.983153372	3.125531591786	protein_codin	XM_023004923.1	probable transcription factor At5g28040
871	gene-LOC11138123	0.662683877666667	3.8125367654333	-0.962467445	0.953627650	-2.52435852412	protein_codin	XM_023004990.1	uncharacterized LOC111381237
872	gene-LOC11138124	2.86478150406667	0.5	0.7482930893	0.953736995	2.518425109306	protein_codin	XM_023005002.1	uncharacterized LOC111381248
873	gene-LOC11138128	4.08104347916667	0.5	1.2745233897	0.983939575	3.028938081055	protein_codin	XM_023005045.1	syntaxin-32%2C transcript variant X1
874	gene-LOC11138128	82.4832005998667	47.399481101366	1.7173386698	0.962217995	0.799229049237	protein_codin	XM_023005058.1	2-hydroxyisoflavanone dehydratase-like
875	gene-LOC11138129	2.85088937036667	7.3051794372666	-0.735650816	0.950444164	-1.35750765427	protein_codin	XM_023005094.1	serine carboxypeptidase-like 40%2C transcript variant X5
876	gene-LOC11138129	5.50146543826667	2.1861148219	0.7893448088	0.958242150	1.331446785832	protein_codin	XM_023005067.1	uncharacterized LOC111381295
877	gene-LOC11138141	5.40913825746667	0.7067508191	0.9716755912	0.975876572	2.936125218015	protein_codin	XM_023005186.1	N-terminal acetyltransferase B complex auxiliary subunit NAA25-like
878	gene-LOC11138143	4.92218294766667	1.605946517	0.7523994413	0.953985060	1.615874433437	protein_codin	XM_023005213.1	plastocyanin-like
879	gene-LOC11138144	8.28545316506667	3.8652543497666	0.7483386073	0.953739488	1.100017253861	protein_codin	XM_023005218.1	DNA-directed RNA polymerase 3%2C chloroplastic
880	gene-LOC11138144	16.0655716973333	25.088347782733	-0.838725010	0.957740934	-0.64304514342	protein_codin	XM_023005220.1	uncharacterized LOC111381443%2C transcript variant X2

881	gene-LOC11138145	13.1366389080333	2.5595323076666	1.2180612677	0.983650430	2.359644078284	protein_codin	XM_023005240.1	extra-large guanine nucleotide-binding protein 1-like
882	gene-LOC11138146	2.91641583476667	0.5	0.7549277249	0.954171078	2.544196439823	protein_codin	XM_023005245.1	probable LRR receptor-like serine/threonine-protein kinase At1g06840%2C transcript variant X1
883	gene-LOC11138146	18.4427021579	4.5857000827666	1.0162511896	0.969993292	2.007836142759	protein_codin	XM_023005250.1	extra-large guanine nucleotide-binding protein 1-like
884	gene-LOC11138146	7.1278717349	1.9257797179	1.0590255496	0.967048948	1.888028687380	protein_codin	XM_023005249.1	uncharacterized LOC111381464%2C transcript variant X1
885	gene-LOC11138147	8.0161583448	2.9652528439666	0.8518204673	0.960916075	1.434755879568	protein_codin	XM_023005262.1	long chain acyl-CoA synthetase 2
886	gene-LOC11138148	4.51153422576667	1.0801683049	0.7436166310	0.953536077	2.062362009093	protein_codin	XM_023005270.1	ethylene-responsive transcription factor 4-like
887	gene-LOC11138155	0.6358831613	3.9196444425333	-0.997722728	0.977129728	-2.62388918028	protein_codin	XM_023005341.1	DUF21 domain-containing protein At2g14520-like%2C transcript variant X3
888	gene-LOC11138156	3.32715207923333	0.5	0.9823641090	0.974745431	2.734287811198	protein_codin	XM_023005364.1	putative transferase At1g60990%2C chloroplastic
889	gene-LOC11138157	4.6167699892	0.9729997351	0.8057895342	0.959689914	2.246372540729	protein_codin	XM_023005365.1	uncharacterized LOC111381572
890	gene-LOC11138157	5.17856918526667	0.9398908767333	1.1520476731	0.982932023	2.461988371236	protein_codin	XM_023005370.1	uncharacterized LOC111381576
891	gene-LOC11138157	4.0209877551	0.9729997351	0.8268469631	0.960345264	2.047038625438	protein_codin	XM_023005371.1	uncharacterized RNA-binding protein C1827.05c
892	gene-LOC11138158	11.0297315929	3.0393125553666	1.1798990663	0.982665590	1.859580732775	protein_codin	XM_023005376.1	transcription factor SRM1-like
893	gene-LOC11138159	2.44818289113333	0.7331400576333	0.6796074784	0.950022873	1.739550598950	protein_codin	XM_023005391.1	protein CNGC15b-like
894	gene-LOC11138160	6.02526691663333	1.7131150868	0.7410749096	0.953458231	1.814403078802	protein_codin	XM_023005402.1	uncharacterized methyltransferase At2g41040%2C chloroplastic
895	gene-LOC11138162	6.13238225956667	1.605946517	0.8405165936	0.960582131	1.933023782175	protein_codin	XM_023005428.1	protein EXORDIUM-like 2
896	gene-LOC11138163	13.3813489936333	36.8704471173	-2.520284821	0.999999999	-1.46224134814	protein_codin	XM_023005432.1	sucrose synthase-like
897	gene-LOC11138163	0.5	2.1928344418	-0.754119384	0.952977800	-2.13279689317	protein_codin	XM_023005438.1	7-deoxyloganetin glucosyltransferase-like
898	gene-LOC11138164	18.5534638875667	12.316269675	0.7745153923	0.956342620	0.591123197871	protein_codin	XM_023005450.1	nitronate monooxygenase
899	gene-LOC11138164	7.07427030216667	2.6198362124333	0.7774198805	0.956728148	1.433104724489	protein_codin	XM_023005449.1	uncharacterized LOC111381647
900	gene-LOC11138166	5.26444207466667	0.9729997351	0.9218365988	0.969481478	2.435769325253	protein_codin	XM_023005463.1	vacuolar protein sorting-associated protein 8 homolog%2C transcript variant X1
901	gene-LOC11138167	30.8769964741333	17.462468444033	0.9781119500	0.975266460	0.822274914277	protein_codin	XM_023005469.1	uncharacterized LOC111381671
902	gene-LOC11138168	3.30966878466667	0.7331400576333	0.7715037602	0.955949480	2.174526107028	protein_codin	XM_023005491.1	putative late blight resistance protein homolog R1A-10
903	gene-LOC11138168	114.0640782021	125.16731013366	-0.793513828	0.956931873	-0.13401330343	protein_codin	XM_023005499.1	protein ATAF2-like
904	gene-LOC11138173	0.5	2.3000030115333	-0.746106397	0.952051421	-2.20163575017	protein_codin	XM_023005548.1	uncharacterized LOC111381734
905	gene-LOC11138175	4.03389633776667	0.9729997351	0.7314148617	0.953323945	2.051662693004	protein_codin	XM_023005565.1	uncharacterized LOC111381753
906	gene-LOC11138176	5.08298724586667	0.5	1.2399412903	0.985446968	3.345676610808	protein_codin	XM_023005586.1	putative serine/threonine-protein kinase-like protein CCR3
907	gene-LOC11138178	4.4436490312	0.9729997351	0.7895772435	0.958269575	2.191233556776	protein_codin	XM_023005612.1	thylakoidal processing peptidase 1%2C chloroplastic-like
908	gene-LOC11138180	0.5	2.8257812237	-1.119397939	0.975307321	-2.49864977434	protein_codin	XM_023005653.1	uncharacterized protein At4g06598-like%2C transcript variant X3
909	gene-LOC11138182	31.4262787156667	13.677126724933	1.3719212208	0.953975631	1.200206265862	protein_codin	XM_023005658.1	LEAF RUST 10 DISEASE-RESISTANCE LOCUS RECEPTOR-LIKE PROTEIN KINASE-like 1.5
910	gene-LOC11138183	7.6729156668	1.5327535062	1.0654250198	0.967108636	2.323649193451	protein_codin	XM_023005671.1	zinc finger MYND domain-containing protein 15%2C transcript variant X8
911	gene-LOC11138184	8.91636998636667	3.4721672453333	0.6844334823	0.950839166	1.360620042376	protein_codin	XM_023005689.1	aldehyde dehydrogenase-like
912	gene-LOC11138187	7.78011853226667	3.2527829943333	0.8306984603	0.960403399	1.258117557385	protein_codin	XM_023005720.1	60S acidic ribosomal protein P0-like
913	gene-LOC11138188	5.79012313253333	0.5	1.5356140165	0.966245092	3.533594028837	protein_codin	XM_023005727.1	monooxygenase 1-like
914	gene-LOC11138188	534.2309301154	708.4789466722	-2.296005528	0.999999999	-0.40726147689	lncRNA	XR_002700423.1	uncharacterized LOC111381885
915	gene-LOC11138189	3.29936781186667	0.5	0.9977881427	0.972486662	2.722189617786	protein_codin	XM_023005742.1	NAC domain-containing protein 35-like
916	gene-LOC11138191	4.38359330713333	0.7331400576333	1.0455521054	0.967489966	2.579953217408	protein_codin	XM_023005765.1	RNA-binding protein 24-like%2C transcript variant X4
917	gene-LOC11138193	3.11979256253333	0.5	1.1572135605	0.983113460	2.641450106346	protein_codin	XM_023005786.1	protein NRT1/ PTR FAMILY 4.3-like
918	gene-LOC11138193	46.4308344732333	62.089190369566	-0.778353482	0.955132434	-0.41925890954	protein_codin	XM_023005787.1	transcription factor GTE2
919	gene-LOC11138195	3.15343919836667	0.5	0.9662992048	0.976157527	2.656926116795	protein_codin	XM_023005798.1	uncharacterized LOC111381955
920	gene-LOC11138195	0.992034422	4.1059807277666	-0.802229875	0.958087000	-2.04926477009	protein_codin	XR_002700463.1	exocyst complex component SEC8-like%2C transcript variant X4
921	gene-LOC11138198	3.4759438232	0.5	1.0983450117	0.971621135	2.797404766086	protein_codin	XM_023005835.1	AUGMIN subunit 8-like%2C transcript variant X6
922	gene-LOC11138198	10.9128196774667	5.2518166157666	0.7287166939	0.953315244	1.055135470588	protein_codin	XM_023005843.1	transmembrane 9 superfamily member 8-like
923	gene-LOC11138199	4.62100829883333	0.9729997351	0.6918994052	0.951860008	2.247696363087	protein_codin	XM_023005852.1	NADPH-dependent pterin aldehyde reductase
924	gene-LOC11138199	9.3196683885	4.0523964429	0.8951268646	0.964128975	1.201503303148	protein_codin	XM_023005856.1	2-hydroxyisoflavanone dehydratase-like%2C transcript variant X3
925	gene-LOC11138200	20.2680142812333	3.2799780407333	1.7094891283	0.961037479	2.627446689297	protein_codin	XM_023005865.1	2-hydroxyisoflavanone dehydratase-like
926	gene-LOC11138202	0.6358831613	2.8852793205666	-0.741086078	0.951338040	-2.18187738055	protein_codin	XM_023005880.1	uncharacterized LOC111382021%2C transcript variant X1
927	gene-LOC11138203	8.8336966297	2.9865340783333	0.7411301482	0.953459584	1.564545103645	protein_codin	XM_023005896.1	CBL-interacting serine/threonine-protein kinase 6-like
928	gene-LOC11138207	2.87670653573333	0.5	0.8103728623	0.959924026	2.524418053528	protein_codin	XM_023005934.1	adenylate kinase-like
929	gene-LOC11138210	2.57415670776667	0.5	0.7724889366	0.956076955	2.364099883642	protein_codin	XM_023005973.1	probable serine/threonine-protein kinase PBL11

930	gene-LOC11138211	0.5	3.5454211488666	-1.2674448684	0.9761375080	-2.825957010622	protein_codin	XM_023005986.1	dnaJ protein homolog ANJ1-like
931	gene-LOC11138211	2.0762599173	0.5	0.6996756982	0.9526031653	2.053987059267	protein_codin	XM_023005990.1	FT-interacting protein 1-like%2C transcript variant X2
932	gene-LOC11138212	2.08916849996667	0.5	0.7050067510	0.9529356131	2.062928856341	protein_codin	XM_023005993.1	uncharacterized LOC111382123%2C transcript variant X1
933	gene-LOC11138212	39.3290840092333	58.1686183338	-0.8165175380	0.9594279912	-0.564644450856	protein_codin	XM_023005996.1	X-linked retinitis pigmentosa GTPase regulator-like
934	gene-LOC11138217	2.08916849996667	0.5	0.7050067510	0.9529356131	2.062928856341	protein_codin	XM_023006064.1	mitogen-activated protein kinase 15-like%2C transcript variant X2
935	gene-LOC11138218	25.3839524292333	51.078581843966	-1.3766738832	0.9848141899	-1.008801747631	protein_codin	XM_023006080.1	uncharacterized LOC111382181
936	gene-LOC11138218	49.9154485695	19.891848853	1.5029988528	0.9691893667	1.327309017155	protein_codin	XM_023006087.1	protein NRT1/ PTR FAMILY 6.3-like
937	gene-LOC11138218	27.6319258287	11.675797465366	1.2588191305	0.9854930132	1.242815025005	protein_codin	XR_002700470.1	putative late blight resistance protein homolog R1B-17%2C transcript variant X2
938	gene-LOC11138219	55.7204473867667	24.739707919766	2.4186049454	0.9999999999	1.171378373804	protein_codin	XM_023006104.1	phosphoprotein ECPP44-like
939	gene-LOC11138220	2.69426815593333	0.5	0.8963939323	0.9643118126	2.429893446933	protein_codin	XM_023006107.1	mitochondrial arginine transporter BAC1
940	gene-LOC11138220	2.46109147383333	0.5	0.9626316258	0.9762078754	2.299298280873	protein_codin	XM_023006108.1	uncharacterized LOC111382204
941	gene-LOC11138221	3.2457663791	0.5	0.8667081315	0.9616033311	2.698559162595	protein_codin	XM_023006115.1	30S ribosomal protein S5%2C chloroplastic
942	gene-LOC11138224	15.9722609655667	6.4503091953333	0.9136409024	0.9675321304	1.308128326160	protein_codin	XM_023006158.1	transcription factor MYB61-like%2C transcript variant X2
943	gene-LOC11138224	2.03556706723333	0.5	0.7088504665	0.9530935972	2.025430755730	protein_codin	XM_023006156.1	alpha-glucosidase-like
944	gene-LOC11138225	0.5	2.9857282704666	-1.0424896930	0.9835565106	-2.578082872542	protein_codin	XM_023006170.1	uncharacterized LOC111382256
945	gene-LOC11138226	3.468897609	0.5	0.9969335846	0.9726133258	2.794477257410	protein_codin	XM_023006179.1	organic cation/carnitine transporter 3-like
946	gene-LOC11138227	0.5	5.2262331851	-1.3530032344	0.9772492346	-3.385771496545	protein_codin	XM_023006196.1	inositol transporter 1-like
947	gene-LOC11138230	3.12624685386667	0.6531665342333	0.8606822829	0.9612914755	2.258908920830	protein_codin	XM_023006224.1	uncharacterized LOC111382303
948	gene-LOC11138230	2.0623677836	0.5	0.7505681576	0.9538637171	2.044301632542	protein_codin	XM_023006227.1	DDB1- and CUL4-associated factor 8-like%2C transcript variant X2
949	gene-LOC11138232	23.5618238302	9.2529853047333	0.8124976397	0.9600101625	1.348460412226	protein_codin	XM_023006256.1	ATP-dependent RNA helicase glh-2-like
950	gene-LOC11138233	6.1065650942	1.0801683049	1.0915003344	0.9700716777	2.499104979593	protein_codin	XM_023006293.1	enhancer of polycomb-like protein 1%2C transcript variant X3
951	gene-LOC11138234	6.36068012416667	15.1685010299	-0.7771259208	0.9550104756	-1.253825582761	protein_codin	XM_023006288.1	squamosa promoter-binding-like protein 6
952	gene-LOC11138236	6.84468478093333	1.6126661369	0.8884576051	0.9633055036	2.085536307743	protein_codin	XM_023006310.1	endoglucanase 25-like
953	gene-LOC11138237	16.3413208089333	10.0501813297	0.8482807203	0.9607944722	0.701303064572	protein_codin	XM_023006317.1	heat stress transcription factor B-2b-like
954	gene-LOC11138239	0.5	2.4599500583333	-0.8054781261	0.9584835197	-2.298629026407	protein_codin	XM_023006359.1	WAT1-related protein At4g01440-like%2C transcript variant X1
955	gene-LOC11138242	2.57415670776667	0.5	0.7724889366	0.9560769550	2.364099883642	protein_codin	XR_002700507.1	probable glucuronoxylan glucuronosyltransferase IRX7%2C transcript variant X2
956	gene-LOC11138243	6.3361506154	1.0801683049	1.0053959457	0.9713797639	2.552350508452	protein_codin	XM_023006406.1	uncharacterized LOC111382432%2C transcript variant X1
957	gene-LOC11138245	1.96905705183333	0.5	0.6815674662	0.9503663139	1.977504912765	protein_codin	XM_023006436.1	ubiquitin carboxyl-terminal hydrolase 18-like%2C transcript variant X2
958	gene-LOC11138245	6.84468478093333	13.569274132733	-0.7905153414	0.9565380295	-0.987287540565	protein_codin	XR_002700515.1	chaperone protein dnaJ C76%2C chloroplastic-like%2C transcript variant X3
959	gene-LOC11138247	2.3058454385	0.5	0.7515754292	0.9539302290	2.205295811956	lncRNA	XR_002700517.1	uncharacterized LOC111382471
960	gene-LOC11138252	15.3781027902667	32.312626103333	-1.0718463729	0.9771754784	-1.071220477950	protein_codin	XM_023006534.1	zingipain-2
961	gene-LOC11138255	19.5286069378667	35.109661557633	-1.3045539983	0.9707018299	-0.846279050252	protein_codin	XM_023006576.1	glucose-1-phosphate adenylyltransferase large subunit 3%2C chloroplastic/amyloplastic-like
962	gene-LOC11138260	933.122021885	825.69173261	0.7972845980	0.9590603838	0.176462490765	protein_codin	XM_023006632.1	myosin-2-like%2C transcript variant X8
963	gene-LOC11138260	0.5	3.2059183293333	-0.9585334287	0.9509747899	-2.680737673350	protein_codin	XM_023006636.1	ferritin-3%2C chloroplastic-like
964	gene-LOC11138261	0.798567038966667	4.5857000827666	-1.1390063349	0.9781550405	-2.521656571080	protein_codin	XM_023006646.1	cytochrome P450 76A2-like
965	gene-LOC11138263	0.5	1.8662816210333	-0.7698099290	0.9543497001	-1.900166704631	protein_codin	XM_023006673.1	remorin-like%2C transcript variant X3
966	gene-LOC11138267	2.14563306316667	8.0238917691666	-0.7613612683	0.9536432840	-1.902898772428	lncRNA	XR_002700588.1	uncharacterized LOC111382676%2C transcript variant X2
967	gene-LOC11138270	5.67517831913333	1.7131150868	0.7410555541	0.9534577571	1.728043649170	protein_codin	XM_023006746.1	putative ubiquitin-conjugating enzyme E2 38
968	gene-LOC11138271	3.14053061566667	12.4226324369	-1.1025060652	0.9741520704	-1.983890683725	protein_codin	XM_023006752.1	mitogen-activated protein kinase kinase kinase 1-like%2C transcript variant X5
969	gene-LOC11138277	0.5	3.5793358151	-1.0503349647	0.9820484490	-2.839691904484	protein_codin	XM_023006815.1	uncharacterized LOC111382779%2C transcript variant X1
970	gene-LOC11138278	3.44914310686667	0.9729997351	0.6993746830	0.9525810244	1.825726670916	protein_codin	XM_023006825.1	ATP-dependent Clp protease proteolytic subunit 3%2C chloroplastic-like
971	gene-LOC11138284	2.51469290656667	0.5	0.9399167264	0.9737462359	2.330382229365	protein_codin	XM_023006882.1	regulator of nonsense transcripts UPF3-like
972	gene-LOC11138288	4.3299918744	0.9729997351	0.8108532468	0.9599433726	2.153853000269	protein_codin	XM_023006921.1	uncharacterized LOC111382886
973	gene-LOC11138290	0.5	2.2456129188	-0.7730286608	0.9546290333	-2.167109268675	protein_codin	XM_023006937.1	probable prolyl 4-hydroxylase 4
974	gene-LOC11138291	2.08916849996667	0.5	0.7050067510	0.9529356131	2.062928856341	protein_codin	XM_023006947.1	serine/threonine-protein kinase HT1-like
975	gene-LOC11138292	79.2629083429333	127.80935517376	-1.9870694569	0.9999997562	-0.689275630838	protein_codin	XM_023006972.1	auxin-responsive protein SAUR36-like
976	gene-LOC11138295	41.6438875381	26.392275530933	0.7780276848	0.9568093249	0.657989008730	protein_codin	XM_023006997.1	transcription factor GTE9-like
977	gene-LOC11138296	1.85599181786667	0.5	0.9346285653	0.9726029951	1.892190350363	protein_codin	XM_023007006.1	probable E3 ubiquitin-protein ligase RHC2A
978	gene-LOC11138296	1.786618672	6.8254600822666	-0.8060842259	0.9585516308	-1.933694551956	protein_codin	XR_002700651.1	transcription factor TCP2-like%2C transcript variant X3

979	gene-LOC11138301	42.8799592412333	25.4065693679	1.1922878817	0.982418351	0.755101952074	protein_codin	XM_023007072.1	palmitoyl-acyl carrier protein thioesterase%2C chloroplastic-like
980	gene-LOC11138302	2.3381168952	0.5	0.7562683526	0.954277178	2.225347059856	protein_codin	XM_023007075.1	cell number regulator 6-like
981	gene-LOC11138304	4.94292100083333	1.0801683049	0.7814553913	0.957263042	2.194107727705	protein_codin	XM_023007106.1	receptor-like kinase TMK3
982	gene-LOC11138306	2517.11300693477	2973.1371980933	-1.643671300	0.999999698	-0.24021605083	lncRNA	XR_002700681.1	uncharacterized LOC111383064%2C transcript variant X5
983	gene-LOC11138306	0.927995908933333	4.2650610740333	-1.036442934	0.984371600	-2.20037605016	protein_codin	XM_023007135.1	protein STRICTOSIDINE SYNTHASE-LIKE 3-like
984	gene-LOC11138310	0.5	3.8448398159666	-1.047924091	0.982552493	-2.94292349375	protein_codin	XM_023007175.1	E3 ubiquitin-protein ligase PUB23-like
985	gene-LOC11138314	2.64066672316667	0.5	0.7971634153	0.959049456	2.400902231389	protein_codin	XM_023007219.1	tubby-related protein 1-like
986	gene-LOC11138315	5.22186964513333	1.605946517	0.6968892718	0.952374178	1.701142596299	protein_codin	XM_023007235.1	uncharacterized LOC111383157
987	gene-LOC11138315	8.0568511949	2.4599500583333	0.8548085385	0.961031778	1.711587083673	protein_codin	XM_023007236.1	GTP-binding nuclear protein Ran-3-like
988	gene-LOC11138316	3.26064206383333	0.5	0.9928877293	0.973226604	2.705156078291	lncRNA	XR_002700716.1	uncharacterized LOC111383160%2C transcript variant X3
989	gene-LOC11138316	0.5	2.4599500583333	-0.805478126	0.958483519	-2.29862902640	protein_codin	XM_023007238.1	cytochrome b-c1 complex subunit 7-like
990	gene-LOC11138318	2.00974990186667	0.5	0.7043739485	0.952899766	2.007015980117	protein_codin	XM_023007265.1	protein VAC14 homolog
991	gene-LOC11138321	1.85599181786667	0.5	0.9346285653	0.972602995	1.892190350363	protein_codin	XM_023007292.1	uncharacterized LOC111383212%2C transcript variant X2
992	gene-LOC11138323	0.5	2.5127285353666	-0.801158618	0.957947684	-2.32925481692	protein_codin	XM_023007316.1	beta-catenin-like protein 1
993	gene-LOC11138330	3.32715207923333	0.5	0.9823641090	0.974745431	2.734287811198	protein_codin	XM_023007389.1	uncharacterized LOC111383305
994	gene-LOC11138331	4.31422016123333	0.9729997351	0.7552529359	0.954196407	2.148588483960	protein_codin	XM_023007397.1	putative F-box/LRR-repeat protein 23
995	gene-LOC11138331	9.3057762548	2.8257812237	1.0444238478	0.967553332	1.719476725458	protein_codin	XM_023007400.1	stachyose synthase-like
996	gene-LOC11138334	1.4175588286	5.6515624473333	-0.981384329	0.967127175	-1.99524116616	protein_codin	XM_023007438.1	uncharacterized LOC111383343
997	gene-LOC11138334	0.5	4.212282597	-1.377930959	0.985255718	-3.07460222829	protein_codin	XR_002700733.1	small GTPase LIP1-like%2C transcript variant X9
998	gene-LOC11138337	69.1460480946	104.49925741	-0.984508711	0.969256933	-0.59577398583	protein_codin	XM_023007479.1	uncharacterized LOC111383371
999	gene-LOC11138337	0.5	2.4599500583333	-0.805478126	0.958483519	-2.29862902640	protein_codin	XM_023007484.1	ubiquitin-conjugating enzyme E2 32-like
1000	gene-LOC11138339	0.6358831613	5.8131211099	-1.206488024	0.985586079	-3.19247935510	protein_codin	XM_023007502.1	thiamine pyrophosphokinase 1%2C transcript variant X1
1001	gene-LOC11138340	11.2024609227667	18.715533794466	-0.809879022	0.958951161	-0.74042050261	protein_codin	XM_023007512.1	ferredoxin-B-like
1002	gene-LOC11138341	1.95614846913333	0.5	0.6824614660	0.950524364	1.968015873129	protein_codin	XM_023007523.1	pathogenesis-related leaf protein 6-like
1003	gene-LOC11138343	6.4617328039	0.8863065918666	1.2382481038	0.985348799	2.866043347204	protein_codin	XM_023007545.1	uncharacterized LOC111383435
1004	gene-LOC11138343	0.5	2.8069783056333	-0.756836526	0.953241779	-2.48901791369	protein_codin	XM_023007546.1	caltractin-like%2C transcript variant X1
1005	gene-LOC11138344	71.8418917244	98.0839577688	-1.363606059	0.980374648	-0.44919185484	protein_codin	XM_023007559.1	pentatricopeptide repeat-containing protein At5g16860
1006	gene-LOC11138346	3.4065706773	0.5	0.8714594633	0.961886645	2.768320139169	lncRNA	XR_002700744.1	uncharacterized LOC111383460
1007	gene-LOC11138348	2.73496100596667	0.5	0.7931074243	0.958659141	2.451520263767	protein_codin	XM_023007590.1	ruvB-like 2
1008	gene-LOC11138350	38.4211304197333	62.036290107133	-1.645017592	0.999999625	-0.69121244658	protein_codin	XM_023007619.1	thiamine thiazole synthase 1%2C chloroplastic-like
1009	gene-LOC11138350	4.04133418013333	0.5	1.5113729229	0.969220714	3.014831653695	lncRNA	XR_002700750.1	uncharacterized LOC111383504
1010	gene-LOC11138352	7.58058848603333	2.5859215462	0.8162074460	0.960130495	1.551631343950	protein_codin	XM_023007635.1	squamosa promoter-binding protein 1-like
1011	gene-LOC11138355	6.4876374918	1.2061397927333	0.9420965089	0.974168146	2.427296082037	protein_codin	XR_002700764.1	uncharacterized LOC111383555%2C transcript variant X3
1012	gene-LOC11138356	2.51469290656667	0.6531665342333	0.7585585326	0.954481629	1.944859449660	protein_codin	XM_023007677.1	probable Xaa-Pro aminopeptidase P
1013	gene-LOC11138356	0.629428869966667	3.7121487082333	-0.829021282	0.959178957	-2.56013924856	protein_codin	XM_023007683.1	calcium-dependent protein kinase 17-like
1014	gene-LOC11138357	13.0626358243667	5.4388978162333	0.6929148389	0.951974133	1.264059811901	protein_codin	XM_023007687.1	protein transport protein SEC23
1015	gene-LOC11138357	0.938432989266667	4.7515000489	-0.734717890	0.950280460	-2.34005740903	protein_codin	XM_023007691.1	3-ketoacyl-CoA synthase 11-like
1016	gene-LOC11138358	0.5	4.5849551676333	-1.119429518	0.975311005	-3.19690762700	protein_codin	XM_023007705.1	40S ribosomal protein S15
1017	gene-LOC11138358	0.5	3.3130868991	-0.994775317	0.975576033	-2.72817604442	protein_codin	XM_023007706.1	auxin-induced protein 22D-like
1018	gene-LOC11138360	40.7982608803	18.556270770133	1.1079837920	0.974170533	1.136600852284	protein_codin	XM_023007721.1	extensin-3-like
1019	gene-LOC11138363	0.5	3.8448398159666	-1.047924091	0.982552493	-2.94292349375	protein_codin	XM_023007746.1	probable sugar phosphate/phosphate translocator At3g11320
1020	gene-LOC11138363	3.0504194166	9.3321530202333	-0.817373112	0.959458289	-1.61320234424	protein_codin	XM_023007757.1	transcription factor MYB25-like
1021	gene-LOC11138365	3.06619112976667	0.5	0.9928071491	0.973238823	2.616447629603	protein_codin	XM_023007784.1	kunitz trypsin inhibitor 2-like
1022	gene-LOC11138366	0.5	2.8325008435666	-0.841171671	0.957201585	-2.50207638570	protein_codin	XM_023007799.1	serine/threonine protein phosphatase 2A 57 kDa regulatory subunit B' kappa isoform-like
1023	gene-LOC11138368	151.349261273267	184.4523954563	-0.744498408	0.951834233	-0.28536689175	protein_codin	XM_023007812.1	putative glycine-rich cell wall structural protein 1
1024	gene-LOC11138369	2.00974990186667	0.5	0.7043739485	0.952899766	2.007015980117	protein_codin	XM_023007827.1	citrate synthase%2C mitochondrial-like%2C transcript variant X2
1025	gene-LOC11138372	3.7119836358	0.9729997351	0.8068732315	0.959754336	1.931679033045	protein_codin	XM_023007871.1	expansin-like B1
1026	gene-LOC11138373	0.5	2.4055599655666	-0.761230258	0.953631963	-2.26637276323	protein_codin	XM_023007872.1	GATA transcription factor 27-like
1027	gene-LOC11138373	2.79689630953333	0.7067508191	0.7308685626	0.953321080	1.984553213985	protein_codin	XM_023007875.1	uncharacterized LOC111383734

1028	gene-LOC11138374	2.74427842776667	0.5	0.9806425169	0.974966424	2.456426861241	protein_codin	XM_023007925.1	clathrin heavy chain 2-like
1029	gene-LOC11138375	7.29740153206667	2.9329497934333	0.7463972797	0.953646414	1.315030463676	protein_codin	XM_023007893.1	uncharacterized LOC111383752
1030	gene-LOC11138375	2.08916849996667	0.5	0.7050067510	0.952935613	2.062928856341	protein_codin	XM_023007896.1	uncharacterized LOC111383754
1031	gene-LOC11138375	3.14053061566667	0.5	0.8434362497	0.960653022	2.651008333612	protein_codin	XM_023007898.1	uncharacterized LOC111383756
1032	gene-LOC11138376	0.5	2.9857282704666	-1.042489693	0.983556510	-2.57808287254	protein_codin	XM_023007913.1	biotin--protein ligase 2-like%2C transcript variant X2
1033	gene-LOC11138376	3.7562676467	0.5	0.8432284553	0.960647812	2.909299863526	protein_codin	XM_023007916.1	mitogen-activated protein kinase kinase kinase 1-like
1034	gene-LOC11138379	68.7007139599667	93.9229029258	-1.134827815	0.977464782	-0.45115190721	protein_codin	XM_023007958.1	kinesin-like protein KIN-7K%2C chloroplastic%2C transcript variant X1
1035	gene-LOC11138386	68.4540919982333	99.545536925633	-1.505521195	0.999998191	-0.54021985046	protein_codin	XR_002700816.1	E3 ubiquitin-protein ligase KEG-like%2C transcript variant X3
1036	gene-LOC11138386	0.5	2.2456129188	-0.773028660	0.954629033	-2.16710926867	protein_codin	XM_023008050.1	uncharacterized LOC111383866
1037	gene-LOC11138389	3.0403739644	0.5	0.9963016230	0.972709801	2.604248785314	protein_codin	XM_023008077.1	UDP-glucuronic acid decarboxylase 6%2C transcript variant X1
1038	gene-LOC11138392	3.7119836358	0.6531665342333	1.1754630923	0.982818258	2.506667570685	protein_codin	XM_023008117.1	ubiquitin carboxyl-terminal hydrolase 12-like
1039	gene-LOC11138396	4.65327975553333	0.5	1.2143581158	0.983339958	3.218247924638	protein_codin	XM_023008171.1	uncharacterized LOC111383969
1040	gene-LOC11138399	91.2643345738	57.480886516766	1.0659716328	0.967125626	0.666968863426	protein_codin	XM_023008216.1	5'-3' exoribonuclease 4-like%2C transcript variant X4
1041	gene-LOC11138399	9.35937768753333	1.8270032764333	1.0878329246	0.969359070	2.356933385850	protein_codin	XM_023008193.1	uncharacterized LOC111383992
1042	gene-LOC11138400	4.16144562826667	0.9729997351	0.7065642233	0.953004166	2.096573470261	protein_codin	XM_023008211.1	uncharacterized LOC111384004%2C transcript variant X2
1043	gene-LOC11138401	1.96260276046667	0.5	0.7250116886	0.953309787	1.972768194454	protein_codin	XM_023008220.1	DExH-box ATP-dependent RNA helicase DExH6-like%2C transcript variant X1
1044	gene-LOC11138405	1.51771547986667	5.5988448630666	-0.772992231	0.954625809	-1.88322784559	protein_codin	XM_023008265.1	proline iminopeptidase-like
1045	gene-LOC11138405	0.992034422	5.1266509358	-0.825681414	0.959415575	-2.36955458484	protein_codin	XM_023008268.1	ubiquitin receptor RAD23c-like%2C transcript variant X2
1046	gene-LOC11138405	1.95614846913333	0.5	0.6824614660	0.950524364	1.968015873129	protein_codin	XM_023008275.1	uncharacterized LOC111384058%2C transcript variant X2
1047	gene-LOC11138406	202.2178986975	372.67163299636	-3.400789003	0.999999999	-0.88199430962	protein_codin	XM_023008287.1	bark storage protein A-like
1048	gene-LOC11138406	0.5	2.2456129188	-0.773028660	0.954629033	-2.16710926867	protein_codin	XM_023008294.1	SUN domain-containing protein 2-like
1049	gene-LOC11138407	8.68033017383333	2.8860851284333	0.8336043422	0.960449188	1.588636065100	protein_codin	XM_023008296.1	major allergen Pru ar 1-like
1050	gene-LOC11138408	1.85599181786667	0.5	0.9346285653	0.972602995	1.892190350363	protein_codin	XM_023008308.1	phospho-2-dehydro-3-deoxyheptonate aldolase 1%2C chloroplastic-like
1051	gene-LOC11138408	17.6790694113333	6.5575386578666	1.2723859234	0.984225176	1.430816023751	protein_codin	XM_023008313.1	UPF0051 protein ABCI8%2C chloroplastic-like
1052	gene-LOC11138416	1.82273681016667	8.1575713628666	-1.151699866	0.980370141	-2.16203344131	protein_codin	XM_023008420.1	cellulose synthase-like protein G2
1053	gene-LOC11138417	2.42138217476667	7.5978784845	-0.788372262	0.956265379	-1.64976583602	protein_codin	XM_023008437.1	proteasome assembly chaperone 2
1054	gene-LOC11138417	5.32907251063333	0.5	1.1351670127	0.980980677	3.413884463426	protein_codin	XM_023008440.1	uncharacterized LOC111384179%2C transcript variant X2
1055	gene-LOC11138419	1.43046741126667	5.5996506709	-0.819311262	0.959519952	-1.96885019802	protein_codin	XM_023008454.1	uncharacterized protein At5g39865-like
1056	gene-LOC11138421	3.8528331371	0.9729997351	0.6901414878	0.951647750	1.985408388120	protein_codin	XM_023008471.1	cleavage and polyadenylation specificity factor subunit 3-I-like%2C transcript variant X1
1057	gene-LOC11138422	648.504871633233	776.9381128978	-1.372030956	0.983191574	-0.26068227296	protein_codin	XM_023008486.1	rRNA-processing protein UTP23 homolog%2C transcript variant X4
1058	gene-LOC11138423	33.8379518405	11.3303199411	1.9194177501	0.999999999	1.578453646988	protein_codin	XM_023008497.1	protein PLASTID MOVEMENT IMPAIRED 1-RELATED 1-like
1059	gene-LOC11138424	4.38359330713333	1.1329467819	0.7549314689	0.954171369	1.952033862036	protein_codin	XM_023008510.1	transcriptional corepressor LEUNIG-like
1060	gene-LOC11138425	12.1191723078333	0.7331400576333	1.5750118779	0.961249808	4.047058527200	protein_codin	XM_023008512.1	gibberellin 3-beta-dioxygenase 1-like
1061	gene-LOC11138432	24.3962049017667	10.691520239933	1.2968245169	0.980013301	1.190189731985	protein_codin	XM_023008588.1	branched-chain-amino-acid aminotransferase-like protein 1%2C transcript variant X1
1062	gene-LOC11138434	2.86379795306667	0.6531665342333	0.7731620567	0.956164673	2.132406931336	protein_codin	XM_023008621.1	protein CMSS1%2C transcript variant X2
1063	gene-LOC11138435	0.5	2.9857282704666	-1.042489693	0.983556510	-2.57808287254	lncRNA	XR_002700893.1	uncharacterized LOC111384351
1064	gene-LOC11138438	17.7039030256667	6.2435583762333	0.9640839747	0.976198279	1.503627053497	protein_codin	XM_023008661.1	primary amine oxidase-like%2C transcript variant X2
1065	gene-LOC11138441	4.13464491186667	20.2091428449	-1.731151892	0.995696921	-2.28917279273	protein_codin	XM_023008683.1	uncharacterized LOC111384415
1066	gene-LOC11138443	1.95614846913333	0.5	0.6824614660	0.950524364	1.968015873129	protein_codin	XR_002700905.1	PHD finger-like domain-containing protein 5B%2C transcript variant X2
1067	gene-LOC11138449	122.400043719033	76.780085004566	1.0421544825	0.967687789	0.672800010803	protein_codin	XM_023008774.1	acetolactate synthase small subunit 1%2C chloroplastic-like
1068	gene-LOC11138450	3.9193431525	0.5	1.1116512714	0.975196636	2.970611891576	protein_codin	XM_023008786.1	probable leucine-rich repeat receptor-like protein kinase At5g49770%2C transcript variant X2
1069	gene-LOC11138455	3.6990750531	0.5	1.2249320160	0.984273993	2.887164572599	protein_codin	XM_023008856.1	protein SENSITIVITY TO RED LIGHT REDUCED 1%2C transcript variant X2
1070	gene-LOC11138456	12.0394173075	27.1401598812	-1.070551363	0.977443163	-1.17266365043	protein_codin	XM_023008855.1	thioredoxin F-type%2C chloroplastic-like
1071	gene-LOC11138456	4.94996721503333	1.7131150868	0.6836328595	0.950710941	1.530796895164	protein_codin	XM_023008863.1	protein CHLOROPLAST IMPORT APPARATUS 2-like
1072	gene-LOC11138458	153.388315690533	196.34371542796	-1.228401179	0.984165345	-0.35619283078	protein_codin	XM_023008879.1	lysM domain receptor-like kinase 3
1073	gene-LOC11138459	8.38620173923333	3.4654476254666	0.7133970152	0.953217836	1.274975821553	protein_codin	XM_023008897.1	spermatogenesis-associated protein 20-like%2C transcript variant X2
1074	gene-LOC11138462	2.66845099056667	0.5	0.8904698014	0.963535251	2.416002514674	protein_codin	XM_023008926.1	rhicadhesin receptor-like
1075	gene-LOC11138464	14.0433048409333	7.4107363913	0.6894675924	0.951560116	0.922193675374	protein_codin	XM_023008958.1	uncharacterized LOC111384647%2C transcript variant X3
1076	gene-LOC11138465	0.5	2.2456129188	-0.773028660	0.954629033	-2.16710926867	protein_codin	XM_023008971.1	protein PXR1

1077	gene-LOC11138466	4.60672453703333	0.9729997351	0.9718072048	0.975865915	2.243230016014	protein_codin	XM_023008977.1	endoribonuclease Dicer homolog 2-like
1078	gene-LOC11138466	1.85599181786667	0.5	0.9346285653	0.972602995	1.892190350363	protein_codin	XM_023008979.1	homeobox protein knotted-1-like 3%2C transcript variant X1
1079	gene-LOC11138466	11.3848993025667	4.8783382373	0.9209363264	0.969259748	1.222659835846	protein_codin	XM_023008984.1	aspartic proteinase-like protein 2
1080	gene-LOC11138467	475.804393072067	629.42797962483	-1.092629170	0.974355830	-0.40367271927	protein_codin	XM_023008992.1	pheophytinase%2C chloroplastic-like
1081	gene-LOC11138474	3.9470398973	0.9398908767333	0.6978914807	0.952457106	2.070205930533	protein_codin	XM_023009058.1	uncharacterized LOC111384741
1082	gene-LOC11138475	3.08008326346667	0.9398908767333	0.7952541554	0.958872385	1.712404179863	protein_codin	XM_023009078.1	helicase-like transcription factor CHR28%2C transcript variant X1
1083	gene-LOC11138476	272.0310692742	93.864605579133	3.8037987067	0.999999999	1.535118279526	protein_codin	XM_023009088.1	cellulose synthase A catalytic subunit 2 [UDP-forming]-like%2C transcript variant X1
1084	gene-LOC11138477	3.3290316587	0.6531665342333	0.8862832525	0.963073194	2.349579810699	protein_codin	XM_023009094.1	pentatricopeptide repeat-containing protein At4g19191%2C mitochondrial%2C transcript variant X2
1085	gene-LOC11138478	2.74786958863333	0.5	0.7977912973	0.959106193	2.458313536799	protein_codin	XM_023009106.1	N-carbamoylputrescine amidase-like%2C transcript variant X1
1086	gene-LOC11138481	3.6519279117	0.7067508191	0.9838878917	0.974540231	2.369384731897	protein_codin	XM_023009151.1	pollen-specific leucine-rich repeat extensin-like protein 1
1087	gene-LOC11138483	2.0762599173	0.5	0.6996756982	0.952603165	2.053987059267	lncRNA	XR_002700968.1	uncharacterized LOC111384832
1088	gene-LOC11138484	10.6177076918333	4.1059807277666	0.8974139910	0.964459895	1.370673568362	protein_codin	XM_023009174.1	protein DOWNY MILDEW RESISTANCE 6-like%2C transcript variant X1
1089	gene-LOC11138487	2.46109147383333	0.5	0.9626316258	0.976207875	2.299298280873	protein_codin	XM_023009213.1	PLASMODESMATA CALLOSE-BINDING PROTEIN 4-like
1090	gene-LOC11138488	9.49018173663333	1.55316804	1.3251557729	0.973126987	2.611221789168	protein_codin	XM_023009230.1	pentatricopeptide repeat-containing protein At1g06140%2C mitochondrial-like
1091	gene-LOC11138490	2.0762599173	0.5	0.6996756982	0.952603165	2.053987059267	protein_codin	XM_023009242.1	uncharacterized LOC111384901
1092	gene-LOC11138491	2.0762599173	0.5	0.6996756982	0.952603165	2.053987059267	protein_codin	XM_023009263.1	transcription factor ABA-INDUCIBLE bHLH-TYPE-like
1093	gene-LOC11138492	4.9757843804	0.7331400576333	1.2509627107	0.985727938	2.762763229956	protein_codin	XM_023009270.1	histone H2B-like
1094	gene-LOC11138494	6.54311850403333	21.006338869866	-1.435284925	0.998751037	-1.68277443598	protein_codin	XR_002700982.1	protein SUPPRESSOR OF QUENCHING 1%2C chloroplastic%2C transcript variant X4
1095	gene-LOC11138496	2.69426815593333	0.5	0.8963939323	0.964311812	2.429893446933	protein_codin	XM_023009316.1	pleiotropic drug resistance protein 1-like
1096	gene-LOC11138500	17.4130293496667	38.8454480311	-1.380506350	0.986162521	-1.15757834044	protein_codin	XM_023009352.1	uncharacterized LOC111385003
1097	gene-LOC11138501	0.5	2.6198362124333	-0.858125250	0.952001352	-2.38947661981	protein_codin	XM_023009364.1	jmjC domain-containing protein 4%2C transcript variant X2
1098	gene-LOC11138501	1.85599181786667	0.5	0.9346285653	0.972602995	1.892190350363	protein_codin	XM_023009371.1	pentatricopeptide repeat-containing protein At5g50280%2C chloroplastic%2C transcript variant X2
1099	gene-LOC11138502	117.3437467377	136.9287217775	-0.758963065	0.953434159	-0.22268413141	protein_codin	XM_023009387.1	uncharacterized LOC111385023%2C transcript variant X2
1100	gene-LOC11138502	3.0643115503	9.6767029512	-0.783621912	0.955697081	-1.65895259143	protein_codin	XM_023009395.1	cellulose synthase A catalytic subunit 3 [UDP-forming]-like%2C transcript variant X1
1101	gene-LOC11138503	16.1052809963667	10.263651768633	0.7168621374	0.953269582	0.649989704571	protein_codin	XM_023009396.1	high-affinity nitrate transporter 3.1-like
1102	gene-LOC11138508	3.74563027163333	0.5	1.0271752284	0.968858085	2.905208499258	lncRNA	XR_002701011.1	uncharacterized LOC111385089
1103	gene-LOC11138515	3.2457663791	0.5	0.8667081315	0.961603331	2.698559162595	lncRNA	XR_002701019.1	uncharacterized LOC111385154
1104	gene-LOC11138516	1.54353264523333	6.8254600822666	-0.854869452	0.953151015	-2.14469030090	protein_codin	XM_023009540.1	protein RER1B-like
1105	gene-LOC11138517	72.2742068246	107.54378437146	-1.255218964	0.979245589	-0.57337136878	protein_codin	XM_023009548.1	cysteine proteinase 3-like
1106	gene-LOC11138523	2.3649176116	10.718715286266	-1.125304191	0.976042273	-2.18027016965	protein_codin	XM_023009609.1	VAN3-binding protein-like
1107	gene-LOC11138523	0.5	3.0920910324	-0.824169473	0.959481544	-2.62858279340	protein_codin	XM_023009611.1	cation/H(+) antiporter 28
1108	gene-LOC11138524	2.03556706723333	0.5	0.7088504665	0.953093597	2.025430755730	protein_codin	XR_002701028.1	vacuolar-processing enzyme-like%2C transcript variant X4
1109	gene-LOC11138524	3.0532825471	0.5	1.1838123257	0.982546110	2.610361100988	protein_codin	XM_023009624.1	zinc finger CCCH domain-containing protein 20-like
1110	gene-LOC11138525	0.5	3.5317871276333	-0.794464778	0.957058227	-2.82039838965	protein_codin	XM_023009633.1	calcineurin B-like protein 10
1111	gene-LOC11138527	2.08916849996667	0.5	0.7050067510	0.952935613	2.062928856341	protein_codin	XM_023009656.1	K(+) efflux antiporter 4-like%2C transcript variant X1
1112	gene-LOC11138534	2.08916849996667	0.5	0.7050067510	0.952935613	2.062928856341	protein_codin	XM_023009724.1	expansin-A8-like
1113	gene-LOC11138537	9.9848237685	3.0393125553666	1.0204121243	0.969531672	1.715991918748	protein_codin	XM_023009754.1	putative late blight resistance protein homolog R1A-10
1114	gene-LOC11138538	8.3991103219	3.0452263673666	0.8242657606	0.960304172	1.463687043036	protein_codin	XM_023009768.1	uncharacterized LOC111385382%2C transcript variant X3
1115	gene-LOC11138539	3.75814722616667	0.9398908767333	0.7975931794	0.959088258	1.999456413970	protein_codin	XM_023009785.1	uncharacterized LOC111385399
1116	gene-LOC11138540	2.5612481251	0.5	0.8691339282	0.961743624	2.356847023146	protein_codin	XM_023009787.1	protein COFACTOR ASSEMBLY OF COMPLEX C SUBUNIT B CCB4%2C chloroplastic
1117	gene-LOC11138540	3.11979256253333	9.9711353995666	-0.845895239	0.955982303	-1.67630768534	protein_codin	XM_023009790.1	glutamate-1-semialdehyde 2%2C1-aminomutase%2C chloroplastic-like
1118	gene-LOC11138540	2.64066672316667	0.5	0.7971634153	0.959049456	2.400902231389	lncRNA	XR_002701062.1	uncharacterized LOC111385407
1119	gene-LOC11138541	9.19211909796667	3.3130868991	0.7539472071	0.954095132	1.472221446000	protein_codin	XM_023009793.1	probable WRKY transcription factor 69
1120	gene-LOC11138541	9.20374002406667	2.2464187266666	0.9274801542	0.970881438	2.034593365216	protein_codin	XM_023009800.1	uncharacterized LOC111385414
1121	gene-LOC11138545	20.0726673187333	8.0248802551333	1.1463832154	0.982521676	1.322680569219	protein_codin	XM_023009845.1	EEF1A lysine methyltransferase 3-like
1122	gene-LOC11138548	0.629428869966667	3.3123419839666	-0.787640276	0.956174148	-2.39573637485	protein_codin	XM_023009874.1	trafficking protein particle complex subunit 6B-like
1123	gene-LOC11138556	0.629428869966667	3.2067850299	-0.785394033	0.955902075	-2.34901238723	protein_codin	XM_023009961.1	uncharacterized LOC111385560
1124	gene-LOC11138558	0.5	3.6848927691333	-1.071787357	0.977187220	-2.88162263731	protein_codin	XM_023009989.1	uncharacterized LOC111385582%2C transcript variant X1
1125	gene-LOC11138558	19.62738841	36.552164639433	-1.167443669	0.982991300	-0.89708862310	protein_codin	XR_002701079.1	uncharacterized LOC111385584%2C transcript variant X3

1126	gene-LOC11138558	2.3381168952	0.5	0.7562683526	0.9542771787	2.225347059856	protein_codin	XM_023009996.1	kinesin-like protein KIN-7E%2C chloroplastic%2C transcript variant X2
1127	gene-LOC11138562	0.629428869966667	2.6454196431	-0.7568338083	0.9532415356	-2.071381338054	protein_codin	XM_023010039.1	proton pump-interactor 1-like
1128	gene-LOC11138565	1.06786185923333	4.559371737	-0.8248182548	0.9594628035	-2.094110011387	protein_codin	XM_023010068.1	zinc finger MYM-type protein 1-like
1129	gene-LOC11138566	2.73496100596667	0.5	0.7931074243	0.9586591417	2.451520263767	protein_codin	XM_023010085.1	uncharacterized LOC111385665
1130	gene-LOC11138569	1.74690937296667	6.5583444657333	-1.0180153673	0.9839805495	-1.908526913445	protein_codin	XM_023010114.1	uncharacterized LOC111385692
1131	gene-LOC11138572	2.43886546933333	14.847056213266	-1.0971749821	0.9741709746	-2.605894825618	protein_codin	XM_023010140.1	putative pentatricopeptide repeat-containing protein At5g08310%2C mitochondrial%2C transcript variant X2
1132	gene-LOC11138572	12.5489350240667	33.776805361866	-1.8548794992	0.9679943923	-1.428467949461	protein_codin	XM_023010141.1	glyceraldehyde-3-phosphate dehydrogenase%2C cytosolic
1133	gene-LOC11138573	0.5	2.4055599655666	-0.7612302589	0.9536319638	-2.266372763231	protein_codin	XM_023010155.1	serine acetyltransferase 5
1134	gene-LOC11138576	4.01167033326667	0.5	1.0491916433	0.9673133071	3.004203054578	protein_codin	XM_023010176.1	uncharacterized LOC111385761
1135	gene-LOC11138578	1.96905705183333	0.5	0.6815674662	0.9503663139	1.977504912765	protein_codin	XM_023010197.1	GRF1-interacting factor 3-like
1136	gene-LOC11138580	0.5	3.4654476254666	-1.1236655386	0.9758268246	-2.793041714651	protein_codin	XM_023010244.1	protein phosphatase 2C 37-like
1137	gene-LOC11138580	2.2725904308	8.5564504938666	-0.7785804459	0.9551551301	-1.912674738640	protein_codin	XM_023010217.1	phenylalanine--tRNA ligase%2C chloroplastic/mitochondrial-like
1138	gene-LOC11138581	0.938432989266667	6.2377054569666	-1.1311817321	0.9768912232	-2.732689795341	protein_codin	XM_023010234.1	uncharacterized LOC111385813
1139	gene-LOC11138582	9.16728548363333	20.714445630466	-1.2201106506	0.9849421026	-1.176070703680	protein_codin	XM_023010247.1	oleosin 1-like
1140	gene-LOC11138584	4.3974854408	0.6531665342333	1.1441485502	0.9822970676	2.751156021524	protein_codin	XM_023010272.1	vacuolar cation/proton exchanger 3-like
1141	gene-LOC11138584	3.963931269	0.5	0.8397074612	0.9605644217	2.986931947707	protein_codin	XM_023010274.1	serine/threonine-protein kinase TOR-like
1142	gene-LOC11138589	127.963725637133	99.119584533666	1.0776727800	0.9679038706	0.368492856490	protein_codin	XM_023010320.1	auxin-responsive protein SAUR66-like
1143	gene-LOC11138591	10.6638712822	2.3000030115333	1.1475973882	0.9826314284	2.213023616364	protein_codin	XM_023010331.1	transmembrane 9 superfamily member 8-like%2C transcript variant X1
1144	gene-LOC11138595	0.5	2.4599500583333	-0.8054781261	0.9584835197	-2.298629026407	protein_codin	XM_023010382.1	1-phosphatidylinositol-3-phosphate 5-kinase FAB1B-like
1145	gene-LOC11138596	25.8756990339333	13.7299660947	1.0777041079	0.9679072182	0.914269774808	protein_codin	XM_023010409.1	UDP-glycosyltransferase 83A1-like
1146	gene-LOC11138598	7.88959260533333	3.3055614713666	0.7209721368	0.9532991808	1.255055461966	protein_codin	XM_023010402.1	gamma-glutamylcyclotransferase 2-1-like
1147	gene-LOC11138600	0.5	2.1928344418	-0.7541193844	0.9529778007	-2.132796893174	protein_codin	XM_023010441.1	E3 ubiquitin-protein ligase SINAT3-like
1148	gene-LOC11138603	3.9044674678	0.5	1.0594868120	0.9670460820	2.965125791912	protein_codin	XM_023010464.1	probable WRKY transcription factor 49
1149	gene-LOC11138606	2.03556706723333	0.5	0.7088504665	0.9530935972	2.025430755730	protein_codin	XM_023010494.1	casein kinase II subunit alpha-2
1150	gene-LOC11138607	6.11400293656667	0.5	1.5821103022	0.9629092882	3.612117245154	protein_codin	XM_023010502.1	DNA replication complex GINS protein SLD5-like
1151	gene-LOC11138608	1463.61875653603	834.7432484178	1.0850487502	0.9688848775	0.810135384681	lncRNA	XR_002701149.1	uncharacterized LOC111386081
1152	gene-LOC11138610	2.08916849996667	0.5	0.7050067510	0.9529356131	2.062928856341	protein_codin	XM_023010541.1	arabinosyltransferase RRA3-like
1153	gene-LOC11138611	6.47562493756667	2.6454196431	0.7038814121	0.9528719884	1.291522833140	protein_codin	XM_023010555.1	putative late blight resistance protein homolog R1B-12
1154	gene-LOC11138613	43.4601700569667	12.636969576466	2.3081633149	0.9999999999	1.782043281917	protein_codin	XM_023010578.1	enolase 2-like%2C transcript variant X4
1155	gene-LOC11138618	3.53599954726667	0.6531665342333	0.7963765602	0.9589787729	2.436595310309	protein_codin	XM_023010635.1	uncharacterized LOC111386180
1156	gene-LOC11138618	2.44818289113333	0.5	0.9691321958	0.9760356773	2.291711338371	protein_codin	XM_023010640.1	U-box domain-containing protein 44-like%2C transcript variant X1
1157	gene-LOC11138618	25.8305189945667	15.731356247066	0.7504390611	0.9538555921	0.715433575659	protein_codin	XM_023010642.1	uncharacterized LOC111386189%2C transcript variant X1
1158	gene-LOC11138619	0.5	2.1928344418	-0.7541193844	0.9529778007	-2.132796893174	protein_codin	XM_023010645.1	probable ATP synthase 24 kDa subunit%2C mitochondrial
1159	gene-LOC11138623	0.5	4.1135061555333	-1.0081192299	0.9815164102	-3.040368602387	protein_codin	XM_023010687.1	uncharacterized LOC111386234
1160	gene-LOC11138626	4.3299918744	0.7067508191	1.0181842022	0.9697750986	2.615090762409	protein_codin	XM_023010728.1	protein PPLZ12
1161	gene-LOC11138628	0.5	4.4333393564333	-1.0929153472	0.9743385867	-3.148393799928	protein_codin	XM_023010744.1	chloride conductance regulatory protein ICln%2C transcript variant X2
1162	gene-LOC11138636	4.14109920323333	0.5	1.1011804887	0.9723362974	3.050013763996	protein_codin	XM_023010818.1	telomere repeat-binding protein 4-like
1163	gene-LOC11138636	3.1598934897	0.7067508191	0.7860407066	0.9578464675	2.160602375200	protein_codin	XM_023010821.1	calcium-dependent protein kinase 34-like
1164	gene-LOC11138643	4.30417470906667	14.930252968133	-1.2467719270	0.9811891473	-1.794430065666	protein_codin	XM_023010901.1	eukaryotic peptide chain release factor subunit 1-3-like
1165	gene-LOC11138644	2.46109147383333	0.5	0.9626316258	0.9762078754	2.299298280873	protein_codin	XM_023010907.1	uncharacterized LOC111386440
1166	gene-LOC11138644	2.2121430786	0.5	0.7760830569	0.9565510789	2.145444700270	protein_codin	XM_023010915.1	exocyst complex component EXO70A1-like%2C transcript variant X1
1167	gene-LOC11138646	4.88345719963333	11.835622726733	-0.7369099526	0.9506668825	-1.277160857329	protein_codin	XM_023010935.1	protein DMR6-LIKE OXYGENASE 2-like
1168	gene-LOC11138647	0.5	2.8852793205666	-0.8592127622	0.9516079860	-2.528710991276	protein_codin	XM_023010936.1	protein DMR6-LIKE OXYGENASE 2-like
1169	gene-LOC11138650	2.03556706723333	0.5	0.7088504665	0.9530935972	2.025430755730	protein_codin	XM_023010976.1	B3 domain-containing transcription factor FUS3-like
1170	gene-LOC11138651	2.25224400576667	0.5	0.7304862915	0.9533195212	2.171363135961	protein_codin	XM_023010979.1	TVP38/TMEM64 family membrane protein slr0305-like
1171	gene-LOC11138653	2.1649959372	0.5	0.7870403322	0.9579706978	2.114364317613	protein_codin	XM_023010995.1	probable WRKY transcription factor 32
1172	gene-LOC11138654	8.80271282956667	2.4854725963	0.7640709088	0.9550480354	1.824428006730	protein_codin	XR_002701248.1	3-deoxy-manno-octulosonate cytidylyltransferase%2C mitochondrial-like%2C transcript variant X4
1173	gene-LOC11138657	3.10688397983333	0.7331400576333	0.8467665488	0.9607460332	2.083307628576	protein_codin	XM_023011034.1	transcription factor TCP2-like
1174	gene-LOC11138657	2.5612481251	0.5	0.8691339282	0.9617436241	2.356847023146	protein_codin	XM_023011039.1	ornithine aminotransferase%2C mitochondrial

AQRNA_Control_vs_AQRNA_G7

1175	gene-LOC11138657	4.2505732763	0.9729997351	0.8405327743	0.9605824862	2.127146113811	protein_codin	XM_023011043.1	50S ribosomal protein HLP%2C mitochondrial%2C transcript variant X1
1176	gene-LOC11138658	5.7568681248	0.9729997351	1.0059765893	0.9713008758	2.564772846816	protein_codin	XM_023011059.1	uncharacterized LOC111386584%2C transcript variant X2
1177	gene-LOC11138662	2.69426815593333	0.5	0.8963939323	0.9643118126	2.429893446933	protein_codin	XM_023011091.1	probable serine/threonine-protein kinase PIX7
1178	gene-LOC11138663	44.9290268140333	65.795987502833	-1.6361128725	0.9999999038	-0.550351792766	protein_codin	XM_023011100.1	beta-glucosidase 46-like
1179	gene-LOC11138663	45.8621893577333	10.585157477966	2.6837486860	1	2.115262494747	lncRNA	XR_002701267.1	uncharacterized LOC111386634%2C transcript variant X2
1180	gene-LOC11138665	2.88316082706667	0.5	0.7432816620	0.9535246442	2.527651314786	protein_codin	XM_023011115.1	BTB/POZ domain and ankyrin repeat-containing protein NPR2-like%2C transcript variant X2
1181	gene-LOC11138667	4.07458918783333	0.8863065918666	0.9735122341	0.9757268558	2.200776861460	protein_codin	XM_023011133.1	uncharacterized LOC111386671
1182	gene-LOC11138669	2.44531976066667	0.5	0.6834549292	0.9506825516	2.290023130602	protein_codin	XR_002701294.1	uncharacterized LOC111386691%2C transcript variant X4
1183	gene-LOC11138670	16.9067111658667	9.4912942591666	0.9558601918	0.9759656888	0.832919306333	protein_codin	XM_023011166.1	DNA ligase 1-like
1184	gene-LOC11138671	68.2813074424667	91.067326554033	-1.2021267478	0.9856352268	-0.415442848628	protein_codin	XM_023011184.1	ferric reduction oxidase 6-like
1185	gene-LOC11138673	5.593792619	1.6126661369	0.6907671638	0.9517295238	1.794378974506	protein_codin	XM_023011197.1	uncharacterized LOC111386736
1186	gene-LOC11138675	2.75718701046667	0.5	1.1062460770	0.9736904163	2.463197123685	protein_codin	XM_023011254.1	uncharacterized LOC111386757%2C transcript variant X5
1187	gene-LOC11138676	7.21571172636667	0.5	1.5039748298	0.9692186400	3.851141702888	protein_codin	XM_023011229.1	monooxygenase 2-like%2C transcript variant X1
1188	gene-LOC11138676	7.06781601083333	1.1329467819	1.2446801054	0.9856465367	2.641184389393	protein_codin	XM_023011239.1	homeobox-leucine zipper protein HAT4-like%2C transcript variant X1
1189	gene-LOC11138681	5.6215768864	1.1329467819	0.9675194258	0.9761098392	2.310894777128	protein_codin	XM_023011289.1	protein SRC2 homolog
1190	gene-LOC11138684	6.56893566936667	0.9729997351	1.1827408879	0.9825775985	2.755148319730	protein_codin	XM_023011328.1	steroid 5-alpha-reductase DET2
1191	gene-LOC11138685	2.74786958863333	0.5	0.7977912973	0.9591061931	2.458313536799	protein_codin	XM_023011344.1	E4 SUMO-protein ligase PIAL2-like%2C transcript variant X2
1192	gene-LOC11138687	226.9558097411	281.6271537832	-1.0953323131	0.9742281665	-0.311375022056	protein_codin	XM_023011360.1	MADS-box transcription factor 23-like%2C transcript variant X2
1193	gene-LOC11138690	9.22537410566667	3.3855349947666	0.7616588678	0.9547864352	1.446223584438	protein_codin	XM_023011410.1	peroxidase 55-like
1194	gene-LOC11138691	1.0614075679	4.4333393564333	-0.7376942124	0.9507954300	-2.062415059526	lncRNA	XR_002701338.1	uncharacterized LOC111386917
1195	gene-LOC11138694	295.456081338133	361.8142672811	-0.9862991887	0.9704544357	-0.292305604270	protein_codin	XM_023011444.1	protein NRT1/ PTR FAMILY 1.2-like
1196	gene-LOC11138695	2.5612481251	0.5	0.8691339282	0.9617436241	2.356847023146	protein_codin	XM_023011452.1	protein kinase PINOID-like%2C transcript variant X2
1197	gene-LOC11138699	47.5712690111	37.295837272266	0.8115824895	0.9599728834	0.351075896261	protein_codin	XM_023011502.1	pentatricopeptide repeat-containing protein At1g03560%2C mitochondrial-like
1198	gene-LOC11138699	2.9968179839	0.5	0.9546740034	0.9758852584	2.583431462330	protein_codin	XM_023011504.1	disease resistance protein RPS2-like%2C transcript variant X1
1199	gene-LOC11138699	7.92186406203333	2.1129218111	0.9108369457	0.9669148803	1.906600563855	protein_codin	XM_023011507.1	cyprosin-like%2C transcript variant X1
1200	gene-LOC11138700	8.3981267709	2.9329497934333	0.9235229900	0.9698993140	1.517715190931	lncRNA	XR_002701346.1	uncharacterized LOC111387008
1201	gene-LOC11138702	14.6677673709	7.0917089983	0.8596009020	0.9612409211	1.048444047455	protein_codin	XM_023011541.1	transcriptional repressor ILP1
1202	gene-LOC11138703	0.5	4.2650610740333	-1.3707894671	0.9827639207	-3.092566400529	protein_codin	XM_023011553.1	GTP-binding protein At2g22870-like
1203	gene-LOC11138704	7.3703658388	2.5127285353666	0.8282820978	0.9603668932	1.552481414431	protein_codin	XM_023011561.1	transcription factor bHLH143-like
1204	gene-LOC11138705	4.29126612636667	1.2061397927333	0.7520517603	0.9539618788	1.831006247132	protein_codin	XM_023011569.1	protein MAK16 homolog
1205	gene-LOC11138705	2.74786958863333	0.5	0.7977912973	0.9591061931	2.458313536799	protein_codin	XM_023011576.1	probable carboxylesterase 120
1206	gene-LOC11138708	22.5101576089333	12.424244052633	0.7504741666	0.9538575500	0.857418083769	protein_codin	XM_023011602.1	1-aminocyclopropane-1-carboxylate oxidase homolog 1-like
1207	gene-LOC11138712	0.938432989266667	4.700454973	-0.7364686489	0.9505887483	-2.324474771654	protein_codin	XM_023011639.1	probable ubiquitin-conjugating enzyme E2 25%2C transcript variant X2
1208	gene-LOC11138712	3.7585388543	0.5	1.0886615056	0.9695147271	2.910171917927	protein_codin	XM_023011659.1	probable ubiquitin-like-specific protease 2B%2C transcript variant X2
1209	gene-LOC11138713	2.33910044623333	0.5	0.8152677717	0.9601034279	2.225953815589	protein_codin	XM_023011816.1	ATP-dependent DNA helicase SRS2-like protein At4g25120%2C transcript variant X3
1210	gene-LOC11138714	0.629428869966667	3.679784765	-0.9579373763	0.9505818571	-2.547506126544	protein_codin	XM_023011681.1	vestitone reductase-like
1211	gene-LOC11138714	5.8834338643	1.5259729936333	0.8896713076	0.9634407392	1.946928999514	protein_codin	XM_023011680.1	UPF0505 protein%2C transcript variant X2
1212	gene-LOC11138716	2.66845099056667	0.6531665342333	0.7381393366	0.9533944324	2.030479734970	protein_codin	XM_023011704.1	protein decapping 5-like
1213	gene-LOC11138718	0.5	2.2456129188	-0.7730286608	0.9546290333	-2.167109268675	protein_codin	XM_023011723.1	serine/threonine-protein phosphatase 2A 65 kDa regulatory subunit A beta isoform-like%2C transcript variant X1
1214	gene-LOC11138719	2.03556706723333	0.5	0.7088504665	0.9530935972	2.025430755730	protein_codin	XM_023011737.1	transcription factor GTE4-like%2C transcript variant X4
1215	gene-LOC11138721	3.17339399523333	0.5	0.9806593731	0.9749642765	2.666026651316	protein_codin	XM_023011751.1	uncharacterized LOC111387210
1216	gene-LOC11138722	0.5	2.1928344418	-0.7541193844	0.9529778007	-2.132796893174	protein_codin	XM_023011767.1	transcription factor UNE10-like
1217	gene-LOC11138723	4.51439735623333	1.0930574109666	0.7074447444	0.9530421565	2.046164231725	protein_codin	XM_023011770.1	probable pectinesterase/pectinesterase inhibitor 25
1218	gene-LOC11138726	3.36040708693333	0.5	0.9285130187	0.9711380328	2.748636014767	protein_codin	XM_023011806.1	transcription factor DUO1-like
1219	gene-LOC11138726	2.7020976264	0.5	0.6901994521	0.9516553100	2.434079799970	protein_codin	XM_023011811.1	uncharacterized LOC111387269
1220	gene-LOC11138727	1.43046741126667	6.2435583762333	-0.7894256610	0.9563987287	-2.125881864469	protein_codin	XM_023011820.1	FGGY carbohydrate kinase domain-containing protein
1221	gene-LOC11138731	7.19438175033333	14.823890206233	-0.8165763165	0.9594300646	-1.042981481823	protein_codin	XM_023011870.1	exocyst complex component SEC6-like
1222	gene-LOC11138732	3.06619112976667	0.5	0.9928071491	0.9732388231	2.616447629603	protein_codin	XM_023011965.1	polyadenylate-binding protein 1-like%2C transcript variant X2
1223	gene-LOC11138736	17.6351770285667	3.732563242	1.4749079526	0.9669978422	2.240217448436	protein_codin	XM_023011917.1	probable carotenoid cleavage dioxygenase 4%2C chloroplastic

AQRNA_Control_vs_AQRNA_G7

1224	gene-LOC11138737	717.817586114633	768.6775040469	-0.771220527	0.954470264	-0.09876117869	lncRNA	XR_002701395.1	uncharacterized LOC111387374
1225	gene-LOC11138743	2.1520873545	0.5	0.9667103261	0.976141508	2.105736638929	protein_codin	XM_023011994.1	multiple organellar RNA editing factor 4%2C mitochondrial-like
1226	gene-LOC11138745	2.14563306316667	0.5	0.7875947307	0.958037379	2.101403373758	protein_codin	XM_023012018.1	uncharacterized LOC111387459%2C transcript variant X3
1227	gene-LOC11138747	0.5	3.6940907053333	-0.812063079	0.959141401	-2.88521929093	protein_codin	XM_023012027.1	probable receptor-like protein kinase At1g11050
1228	gene-LOC11138747	107.7967088097	151.2893077093	-1.697288621	0.999753004	-0.48899689827	protein_codin	XM_023012032.1	potassium channel AKT1-like
1229	gene-LOC11138747	8.60736586706667	17.5696370138	-0.977856300	0.964650761	-1.02944068694	protein_codin	XR_002701404.1	uncharacterized LOC111387478%2C transcript variant X5
1230	gene-LOC11138748	25.8702282936	6.7982041432	2.4542254418	1	1.928067194923	protein_codin	XM_023012035.1	beta-galactosidase-like
1231	gene-LOC11138748	479.565380499533	214.16493388103	3.7706921494	0.999999999	1.163005234390	lncRNA	XR_002701406.1	uncharacterized LOC111387482
1232	gene-LOC11138748	1.6507355107	6.4775651344333	-0.833045519	0.958726038	-1.97234263455	protein_codin	XM_023012039.1	beta-galactosidase-like
1233	gene-LOC11138749	4.5402145216	17.489785275833	-1.326475010	0.972094435	-1.94568020677	protein_codin	XM_023012055.1	UDP-glycosyltransferase 83A1-like
1234	gene-LOC11138751	0.5	2.4055599655666	-0.761230258	0.953631963	-2.26637276323	protein_codin	XM_023012071.1	EIN3-binding F-box protein 1-like
1235	gene-LOC11138752	8.527947269	0.7067508191	1.7105654385	0.961122635	3.592924962279	protein_codin	XM_023012088.1	uncharacterized LOC111387520
1236	gene-LOC11138753	0.9255244066	3.6262004801	-0.761743667	0.953676399	-1.97011574852	protein_codin	XM_023012112.1	nuclear envelope-associated protein 2-like%2C transcript variant X2
1237	gene-LOC11138755	14.0003407832667	5.7126721599666	0.8968833068	0.964382756	1.293224300614	protein_codin	XM_023012121.1	methylcrotonoyl-CoA carboxylase subunit alpha%2C mitochondrial%2C transcript variant X1
1238	gene-LOC11138755	64.6548925905667	93.5727732325	-0.982841734	0.968132773	-0.53332926511	protein_codin	XM_023012126.1	granule-bound starch synthase 1%2C chloroplastic/amyloplastic%2C transcript variant X1
1239	gene-LOC11138755	2.57415670776667	0.5	0.7724889366	0.956076955	2.364099883642	protein_codin	XM_023012130.1	uncharacterized LOC111387559%2C transcript variant X2
1240	gene-LOC11138757	392.8991554156	328.18669543323	1.1804527725	0.982647081	0.259642312836	protein_codin	XM_023012151.1	ubiquitin carboxyl-terminal hydrolase 9-like%2C transcript variant X4
1241	gene-LOC11138759	4.30417470906667	0.9729997351	0.9053594435	0.965807448	2.145225321607	lncRNA	XR_002701433.1	uncharacterized LOC111387598
1242	gene-LOC11138760	2.7636413018	0.5	0.9758625769	0.975506199	2.466570377775	protein_codin	XM_023012171.1	uncharacterized LOC111387600
1243	gene-LOC11138760	0.6358831613	5.1989772460666	-1.202997503	0.985630481	-3.03139423038	protein_codin	XM_023012177.1	leucine-rich repeat receptor-like serine/threonine-protein kinase BAM3
1244	gene-LOC11138761	3.58999260816667	0.5	1.0608343626	0.967047916	2.843980873526	protein_codin	XM_023012182.1	protein WVD2-like 5%2C transcript variant X1
1245	gene-LOC11138767	2.08916849996667	0.5	0.7050067510	0.952935613	2.062928856341	lncRNA	XR_002701445.1	uncharacterized LOC111387673%2C transcript variant X2
1246	gene-LOC11138770	0.5	2.8325008435666	-0.841171671	0.957201585	-2.50207638570	protein_codin	XM_023012284.1	probable protein phosphatase 2C 47
1247	gene-LOC11138772	16.2383010272	7.7577646386	0.9026418696	0.965311218	1.065687782861	protein_codin	XM_023012312.1	potassium channel AKT2/3%2C transcript variant X2
1248	gene-LOC11138775	3.0532825471	0.5	1.1838123257	0.982546110	2.610361100988	protein_codin	XM_023012338.1	beta-galactosidase-like
1249	gene-LOC11138775	0.9255244066	6.5039543729666	-1.137214701	0.977855064	-2.81297419615	protein_codin	XM_023012341.1	PHD finger protein Alfin1-like%2C transcript variant X1
1250	gene-LOC11138776	9.07071999323333	1.8126973361	1.3206630141	0.974413516	2.323079009857	protein_codin	XM_023012359.1	protein STICHEL-like
1251	gene-LOC11138777	17.7145956266333	7.6249517454333	0.9634857141	0.976205564	1.216138420648	protein_codin	XM_023012362.1	subtilisin-like protease SBT1.4
1252	gene-LOC11138778	0.5	2.4055599655666	-0.761230258	0.953631963	-2.26637276323	protein_codin	XM_023012375.1	protein NRT1/ PTR FAMILY 4.3-like
1253	gene-LOC11138779	4.39590996693333	0.5	0.9030698330	0.965385647	3.136161838609	protein_codin	XM_023012380.1	E3 ubiquitin-protein ligase RFI2-like%2C transcript variant X1
1254	gene-LOC11138781	5.07554940353333	1.0801683049	0.8127550945	0.960020699	2.232307874075	protein_codin	XM_023012401.1	alpha-xylosidase 1-like
1255	gene-LOC11138786	79.9726355511667	51.6117636984	1.0988077318	0.971734022	0.631806501816	protein_codin	XM_023012457.1	arogenate dehydratase/prephenate dehydratase 6%2C chloroplastic-like
1256	gene-LOC11138787	17.9935442709667	39.772294610466	-1.010278949	0.982193917	-1.14428441010	protein_codin	XM_023012467.1	EIN3-binding F-box protein 2-like
1257	gene-LOC11138789	0.938432989266667	4.2446465402333	-0.747497122	0.952230010	-2.17731878758	protein_codin	XM_023012494.1	DCN1-like protein 1
1258	gene-LOC11138790	5.95092743076667	2.1861148219	0.7899418787	0.958312708	1.444745346745	protein_codin	XM_023012513.1	phosphatidylinositol 3%2C4%2C5-trisphosphate 3-phosphatase and protein-tyrosine-phosphatase PTEN2A-like%2C transcript variant X1
1259	gene-LOC11138791	125.594906118167	156.3055372578	-1.269560215	0.975604176	-0.31559093540	protein_codin	XM_023012539.1	transcription initiation factor IIF subunit alpha-like%2C transcript variant X1
1260	gene-LOC11138791	157.864595509933	139.2691429911	0.9351263478	0.972713615	0.180812008192	protein_codin	XR_002701478.1	scopoletin glucosyltransferase-like%2C transcript variant X2
1261	gene-LOC11138791	4.928637239	17.513635305333	-1.280873588	0.973064910	-1.82921787128	protein_codin	XM_023012542.1	mavicyanin-like
1262	gene-LOC11138796	11.1895523400667	21.434085555666	-1.129660987	0.976662627	-0.93775454900	protein_codin	XM_023012609.1	protein NRT1/ PTR FAMILY 2.13
1263	gene-LOC11138805	14.5989861478667	6.2113162184333	1.0802851205	0.968206458	1.232897259656	protein_codin	XM_023012713.1	uncharacterized LOC111388051
1264	gene-LOC11138807	514.503018801867	592.5682246336	-0.848868464	0.955111411	-0.20380172450	protein_codin	XM_023012737.1	E3 ubiquitin-protein ligase RNF14-like
1265	gene-LOC11138818	6.70939354256667	1.8926708595333	0.7246236234	0.953309066	1.825758823741	protein_codin	XM_023012864.1	uncharacterized LOC111388180
1266	gene-LOC11138820	3.16634778103333	0.5	0.9642200631	0.976196617	2.662819724968	protein_codin	XM_023012891.1	serine/threonine-protein kinase WNK1-like
1267	gene-LOC11138823	1.95614846913333	0.5	0.6824614660	0.950524364	1.968015873129	protein_codin	XR_002701529.1	uncharacterized LOC111388231%2C transcript variant X2
1268	gene-LOC11138826	15.7297668616667	24.0480079561	-0.814421432	0.959310869	-0.61242010362	protein_codin	XM_023012950.1	uncharacterized LOC111388263
1269	gene-LOC11138828	2.69426815593333	0.5	0.8963939323	0.964311812	2.429893446933	protein_codin	XM_023012974.1	cyclase-associated protein 1-like
1270	gene-LOC11138830	3.0403739644	0.5	0.9963016230	0.972709801	2.604248785314	protein_codin	XM_023012986.1	uncharacterized LOC111388308%2C transcript variant X3
1271	gene-LOC11138832	2.44818289113333	9.9726252299	-0.964800524	0.955254390	-2.02626199650	protein_codin	XM_023013000.1	sucrose transport protein SUC4
1272	gene-LOC11138834	0.938432989266667	7.9992359318	-1.276086682	0.974064794	-3.09153656849	protein_codin	XM_023013024.1	B3 domain-containing protein Os01g0723500-like

1273	gene-LOC11138835	20.4149264457667	11.0361310636	0.8573108014	0.9611373576	0.887389874438	protein_codin	XM_023013032.1	geranylgeranyl diphosphate reductase%2C chloroplastic
1274	gene-LOC11138837	8.33905459783333	4.0260072044	0.7381215269	0.9533941344	1.050534080075	protein_codin	XM_023013051.1	cyclic dof factor 1-like
1275	gene-LOC11138837	0.5	2.3000030115333	-0.7461063970	0.9520514212	-2.20163575017	lncRNA	XR_002701555.1	uncharacterized LOC111388379%2C transcript variant X1
1276	gene-LOC11138839	1.96905705183333	7.8428461661	-0.8120052343	0.9591366693	-1.99387239011	protein_codin	XM_023013071.1	HVA22-like protein a%2C transcript variant X1
1277	gene-LOC11138839	3.16634778103333	0.5	0.9642200631	0.9761966175	2.662819724968	protein_codin	XM_023013084.1	probable inactive ATP-dependent zinc metalloprotease FTSHI 5%2C chloroplastic
1278	gene-LOC11138840	70.6435622183	28.044402690566	1.9600606726	0.9999999999	1.332845237246	protein_codin	XM_023013092.1	pathogenesis-related protein STH-21-like
1279	gene-LOC11138840	11588.3804766028	3040.2368404083	2.4227711086	1	1.930423335989	lncRNA	XR_002701559.1	uncharacterized LOC111388402
1280	gene-LOC11138841	36.9202510856333	10.478049800933	1.5892814699	0.9655339503	1.817042140339	protein_codin	XM_023013101.1	pathogenesis-related protein STH-2-like
1281	gene-LOC11138842	3.64547362036667	0.8599173533333	0.8936636643	0.9639339408	2.083836347687	protein_codin	XR_002701561.1	cell division protein FtsZ homolog 2-1%2C chloroplastic-like%2C transcript variant X3
1282	gene-LOC11138843	3.32715207923333	0.5	0.9823641090	0.9747454313	2.734287811198	protein_codin	XM_023013129.1	F-box/kelch-repeat protein At1g57790-like
1283	gene-LOC11138844	3.1941320484	0.5	0.8612726627	0.9613200286	2.675423956341	protein_codin	XM_023013152.1	protein SRG1-like
1284	gene-LOC11138849	2.44818289113333	0.5	0.9691321958	0.9760356773	2.291711338371	protein_codin	XM_023013227.1	probable ubiquitin-conjugating enzyme E2 18
1285	gene-LOC11138850	3.638035778	0.5	1.0053162632	0.9713911709	2.863159731139	protein_codin	XM_023013233.1	V-type proton ATPase 16 kDa proteolipid subunit
1286	gene-LOC11138850	1.24098281723333	5.1190646153666	-0.8584833526	0.9518720397	-2.04439707694	protein_codin	XM_023013240.1	uncharacterized TPR repeat-containing protein At1g05150-like
1287	gene-LOC11138854	0.662683877666667	6.4035054230666	-1.2151520772	0.9852640306	-3.27246915926	protein_codin	XM_023013290.1	basic helix-loop-helix protein A
1288	gene-LOC11138856	0.5	2.9857282704666	-1.0424896930	0.9835565106	-2.57808287254	protein_codin	XM_023013323.1	adenylate isopentenyltransferase 3%2C chloroplastic-like
1289	gene-LOC11138857	0.629428869966667	3.946033681	-1.1388299861	0.9781252844	-2.64828801285	protein_codin	XM_023013333.1	uncharacterized LOC111388576
1290	gene-LOC11138860	2.03556706723333	0.5	0.7088504665	0.9530935972	2.025430755730	protein_codin	XM_023013366.1	uncharacterized LOC111388609
1291	gene-LOC11138863	1.96905705183333	0.5	0.6815674662	0.9503663139	1.977504912765	lncRNA	XR_002701620.1	uncharacterized LOC111388635
1292	gene-LOC11138864	3.66483649436667	10.717848585733	-0.9820439696	0.9675812012	-1.54819460043	protein_codin	XM_023013396.1	dynamin-2A-like
1293	gene-LOC11138868	6.71166475013333	2.5126676426666	0.7034621379	0.9528484262	1.417450799814	protein_codin	XM_023013459.1	vacuolar-sorting receptor 3-like
1294	gene-LOC11138870	1.75981795563333	5.8642879712	-0.7624420752	0.9537368762	-1.73652975307	protein_codin	XM_023013481.1	V-type proton ATPase catalytic subunit A-like%2C transcript variant X1
1295	gene-LOC11138872	3.78632312166667	0.5	0.9192171608	0.9688408287	2.920797534690	protein_codin	XM_023013509.1	protein NRT1/ PTR FAMILY 2.11-like
1296	gene-LOC11138872	9.9793530282	4.0063984784666	0.7534934151	0.9540601960	1.316640367696	protein_codin	XM_023013510.1	protein NRT1/ PTR FAMILY 2.11-like
1297	gene-LOC11138872	61.9978299346333	29.274362926666	1.6551865640	0.9758248683	1.082579943858	protein_codin	XM_023013514.1	uncharacterized LOC111388725%2C transcript variant X2
1298	gene-LOC11138874	0.5	3.6253946722666	-1.2884881302	0.9718153763	-2.85813806015	protein_codin	XM_023013535.1	pentatricopeptide repeat-containing protein At5g02860
1299	gene-LOC11138875	0.5	3.0452263673666	-0.9594840334	0.9516049861	-2.60654947507	protein_codin	XM_023013546.1	ubiquitin carboxyl-terminal hydrolase 8-like
1300	gene-LOC11138875	3.10688397983333	0.7067508191	0.8762912559	0.9622152330	2.136194812794	protein_codin	XM_023013553.1	nitrate regulatory gene2 protein-like%2C transcript variant X2
1301	gene-LOC11138877	8.548293694	19.1168912418	-0.9823373543	0.9677837302	-1.16113955295	protein_codin	XM_023013579.1	uncharacterized LOC111388778
1302	gene-LOC11138878	20.8602053545	12.396988113566	0.7678757809	0.9554933704	0.750763704295	protein_codin	XM_023013589.1	protein ETHYLENE INSENSITIVE 3-like%2C transcript variant X3
1303	gene-LOC11138881	3.46303524053333	0.5	1.0479147668	0.9673686938	2.792037068760	protein_codin	XM_023013627.1	abscisate beta-glucosyltransferase-like
1304	gene-LOC11138885	2.69426815593333	9.2778238202	-1.0060913917	0.9808140962	-1.78389300368	protein_codin	XM_023013663.1	uncharacterized LOC111388851
1305	gene-LOC11138888	6.12592796823333	0.6531665342333	1.3734894250	0.9532920032	3.229405623392	protein_codin	XM_023013709.1	heterogeneous nuclear ribonucleoprotein 1-like%2C transcript variant X6
1306	gene-LOC11138891	5.8441161934	1.4995837551	0.7660309724	0.9552733047	1.962422760062	protein_codin	XM_023013736.1	probable serine/threonine-protein kinase PBL9
1307	gene-LOC11138891	1.1279175833	5.8115094941666	-1.1277524725	0.9763830582	-2.36525128689	protein_codin	XM_023013744.1	ras-related protein RABE1c-like%2C transcript variant X3
1308	gene-LOC11138892	25.5857536830667	15.7026104776	0.7949655893	0.9588434974	0.704336312012	lncRNA	XR_002701653.1	uncharacterized LOC111388923
1309	gene-LOC11138895	4.09493561283333	0.9398908767333	0.8658465158	0.9615564536	2.123275595911	lncRNA	XR_002701658.1	uncharacterized LOC111388959
1310	gene-LOC11138900	8.3241789131	16.075967070666	-0.8139228747	0.9592816070	-0.94952564872	protein_codin	XM_023013845.1	anaphase-promoting complex subunit 1%2C transcript variant X5
1311	gene-LOC11138901	0.5	2.1861148219	-1.0350065102	0.9845091010	-2.12836917806	protein_codin	XM_023013862.1	uncharacterized LOC111389016%2C transcript variant X2
1312	gene-LOC11138902	13.1785641888	1.9325602304666	1.3185816242	0.9749775834	2.769607913340	protein_codin	XM_023013873.1	protein RMD5 homolog
1313	gene-LOC11138902	7.36391154746667	2.0865325725666	1.0107075627	0.9706699249	1.819364855195	protein_codin	XM_023013879.1	hemiasterlin resistant protein 1-like
1314	gene-LOC11138905	23.1046198898	48.6830865151	-1.4199892161	0.9969647030	-1.07523928311	lncRNA	XR_002701669.1	uncharacterized LOC111389053%2C transcript variant X2
1315	gene-LOC11138910	13.2825675215333	5.0118960455666	1.0217889247	0.9693862844	1.406105650358	protein_codin	XM_023013960.1	nitrate reductase [NADH] 2-like
1316	gene-LOC11138910	10.6929432061667	4.6392843676666	0.8287282500	0.9603736193	1.204684821107	protein_codin	XM_023013965.1	transmembrane protein 50 homolog%2C transcript variant X2
1317	gene-LOC11138912	1.85599181786667	0.5	0.9346285653	0.9726029951	1.892190350363	protein_codin	XM_023013999.1	rac-like GTP-binding protein 5
1318	gene-LOC11138913	4.93607508136667	1.6126661369	0.6830392504	0.9506163780	1.613916544450	protein_codin	XM_023014020.1	probable carboxylesterase 11
1319	gene-LOC11138917	4.37068472446667	0.5	1.4824532551	0.9678217932	3.127859314327	protein_codin	XM_023014056.1	succinate dehydrogenase [ubiquinone] iron-sulfur subunit 2%2C mitochondrial-like
1320	gene-LOC11138918	2.39171832793333	0.5	0.8150955481	0.9600977330	2.258047493707	protein_codin	XR_002701691.1	uncharacterized LOC111389184%2C transcript variant X3
1321	gene-LOC11138920	0.5	3.6865043849	-1.0337615702	0.9846079526	-2.88225347297	protein_codin	XM_023014096.1	UDP-glycosyltransferase 85A8-like

1322	gene-LOC11138920	45.8256795914	12.9559969695	1.9347600978	0.999999999	1.822536240194	protein_codin	XM_023014097.1	scarecrow-like protein 4
1323	gene-LOC11138920	3.941569157	0.7067508191	0.9888475993	0.973832266	2.479496532432	lncRNA	XR_002701701.1	uncharacterized LOC111389207%2C transcript variant X1
1324	gene-LOC11138921	2.7537319571	0.5	0.6975232012	0.952426558	2.461388137184	protein_codin	XM_023014109.1	outer dense fiber protein 2%2C transcript variant X1
1325	gene-LOC11138922	3.39366209463333	0.7067508191	0.7139091711	0.953227570	2.263569368348	protein_codin	XM_023014114.1	glutathione S-transferase DHAR2-like
1326	gene-LOC11138926	2.7775334355	0.5	1.1021262443	0.972581813	2.473804278649	protein_codin	XM_023014187.1	probable protein phosphatase 2C 60%2C transcript variant X2
1327	gene-LOC11138926	2.51469290656667	0.7067508191	0.7020470682	0.952765813	1.831108674160	protein_codin	XM_023014180.1	9-cis-epoxycarotenoid dioxygenase NCED1%2C chloroplastic-like
1328	gene-LOC11138927	1.0614075679	4.1051140272333	-0.744666889	0.951857977	-1.95144355571	protein_codin	XM_023014186.1	1-deoxy-D-xylulose 5-phosphate reductoisomerase%2C chloroplastic
1329	gene-LOC11138927	1.96905705183333	0.5	0.6815674662	0.950366313	1.977504912765	protein_codin	XM_023014191.1	70 kDa peptidyl-prolyl isomerase-like
1330	gene-LOC11138927	2.40561046163333	7.3051794372666	-0.824839825	0.959461628	-1.60251666331	protein_codin	XM_023014195.1	V-type proton ATPase subunit B2
1331	gene-LOC11138930	4.3642304331	0.7067508191	1.1302527001	0.980010097	2.626453723339	protein_codin	XM_023014229.1	glycine-rich cell wall structural protein 1.0-like
1332	gene-LOC11138933	2.64066672316667	0.5	0.7971634153	0.959049456	2.400902231389	protein_codin	XM_023014258.1	F-box/kelch-repeat protein At1g55270-like%2C transcript variant X2
1333	gene-LOC11138934	1.85599181786667	0.5	0.9346285653	0.972602995	1.892190350363	protein_codin	XM_023014262.1	TNF receptor-associated factor 5-like%2C transcript variant X1
1334	gene-LOC11138936	2.46109147383333	0.5	0.9626316258	0.976207875	2.299298280873	protein_codin	XM_023014287.1	WRKY transcription factor 6-like
1335	gene-LOC11138938	1.96905705183333	0.5	0.6815674662	0.950366313	1.977504912765	protein_codin	XM_023014307.1	probable xyloglucan endotransglucosylase/hydrolase protein 8
1336	gene-LOC11138938	0.5	3.7853417190666	-1.078716400	0.975909185	-2.92042354437	protein_codin	XM_023014309.1	aspartic proteinase-like
1337	gene-LOC11138942	3.10688397983333	0.5	1.1604430177	0.983150623	2.635468367998	protein_codin	XM_023014346.1	uncharacterized LOC111389423
1338	gene-LOC11138942	4.31708329173333	0.7331400576333	1.1372607545	0.981340431	2.557896188312	protein_codin	XM_023014348.1	protein PHR1-LIKE 1-like
1339	gene-LOC11138945	3.14053061566667	0.7067508191	0.6861993763	0.951107846	2.151734778408	protein_codin	XM_023014377.1	uncharacterized LOC111389459%2C transcript variant X3
1340	gene-LOC11138946	6.30609514043333	1.1329467819	0.9645879681	0.976192116	2.476666841418	protein_codin	XM_023014378.1	uncharacterized LOC111389460
1341	gene-LOC11138947	3.67774507703333	0.7067508191	0.7808521076	0.957182844	2.379547927578	protein_codin	XM_023014387.1	calcium-dependent protein kinase 17-like
1342	gene-LOC11138948	0.5	2.2456129188	-0.773028660	0.954629033	-2.16710926867	protein_codin	XM_023014398.1	pre-mRNA cleavage factor Im 25 kDa subunit 1
1343	gene-LOC11138951	3.0403739644	7.3051794372666	-0.737735512	0.950802223	-1.26467092509	protein_codin	XM_023014445.1	triphosphate tunel metalloenzyme 3-like%2C transcript variant X2
1344	gene-LOC11138954	0.5	2.1928344418	-0.754119384	0.952977800	-2.13279689317	protein_codin	XM_023014471.1	40S ribosomal protein S5-like
1345	gene-LOC11138959	2.42138217476667	0.5	0.8950319652	0.964115330	2.275830802644	protein_codin	XM_023014520.1	homeobox protein SBH1-like
1346	gene-LOC11138959	2.9645465272	0.7067508191	0.7485387102	0.953750461	2.068537884139	protein_codin	XM_023014523.1	pyrophosphate--fructose 6-phosphate 1-phosphotransferase subunit beta-like
1347	gene-LOC11138962	1.54353264523333	10.264518469233	-1.366840299	0.981429611	-2.73335804815	protein_codin	XM_023014548.1	spindle and kinetochore-associated protein 1 homolog
1348	gene-LOC11138963	102.841270854333	4.3798159642333	8.6434226907	1	4.553405282774	protein_codin	XM_023014570.1	pyridine nucleotide-disulfide oxidoreductase domain-containing protein 2-like%2C transcript variant X1
1349	gene-LOC11138966	2.43886546933333	0.5	0.6809304862	0.950254362	2.286210179586	protein_codin	XM_023014609.1	rhomboid-like protein 19
1350	gene-LOC11138968	2.85734366173333	0.5	0.9248028319	0.970216734	2.514674564192	protein_codin	XM_023014626.1	uncharacterized LOC111389683%2C transcript variant X2
1351	gene-LOC11138969	4.6661883382	1.0930574109666	0.8369224444	0.960507956	2.093875360936	protein_codin	XM_023014636.1	protein PHOX1-like
1352	gene-LOC11138970	3.78632312166667	0.5	0.9192171608	0.968840828	2.920797534690	protein_codin	XM_023014648.1	zinc finger CCCH domain-containing protein 2-like
1353	gene-LOC11138971	7.17501887633333	0.7067508191	1.5471590706	0.963571225	3.343709072090	protein_codin	XM_023014661.1	polyadenylate-binding protein 2-like
1354	gene-LOC11138974	2.4185190443	0.5	0.7545467141	0.954141492	2.274123898134	protein_codin	XM_023014702.1	protein ABC transporter 1%2C mitochondrial
1355	gene-LOC11138978	9.43879628563333	3.4118633405666	0.7082917627	0.953075457	1.468043024933	protein_codin	XM_023014754.1	cation/calcium exchanger 1
1356	gene-LOC11138978	361.189855423667	306.6343568784	0.9452029806	0.974716364	0.236238020094	protein_codin	XM_023014759.1	protein PNS1-like
1357	gene-LOC11138983	0.662683877666667	3.1991987094666	-0.993536878	0.974869719	-2.27131787843	protein_codin	XM_023014809.1	histone H2AX-like
1358	gene-LOC11138984	0.5	5.7059525401	-1.490307952	0.999989440	-3.51246774719	protein_codin	XM_023014817.1	protein indeterminate-domain 2-like
1359	gene-LOC11138985	2.44531976066667	0.5	0.6834549292	0.950682551	2.290023130602	protein_codin	XM_023014828.1	uncharacterized MFS-type transporter YfnC-like
1360	gene-LOC11138986	304.2965363647	372.91576528403	-2.072008822	0.999999999	-0.29337187681	protein_codin	XM_023014839.1	DNA-directed RNA polymerase III subunit RPC5-like%2C transcript variant X1
1361	gene-LOC11138988	4.8760193573	13.463656285966	-1.086341339	0.974874609	-1.46529449321	protein_codin	XM_023014866.1	F-box protein PP2-B15-like
1362	gene-LOC11138989	2.48143789886667	8.7444592876	-1.295859009	0.971043879	-1.81719282824	protein_codin	XM_023014872.1	lon protease homolog 2%2C peroxisomal%2C transcript variant X2
1363	gene-LOC11138990	0.5	3.7853417190666	-1.078716400	0.975909185	-2.92042354437	protein_codin	XM_023014886.1	two-component response regulator-like APRR2
1364	gene-LOC11138990	3.0532825471	9.6512413059666	-1.060850294	0.979634205	-1.66035340727	protein_codin	XM_023014896.1	uncharacterized LOC111389909
1365	gene-LOC11138992	3.941569157	0.7331400576333	0.9618441900	0.976197923	2.426609348215	protein_codin	XM_023014911.1	uncharacterized LOC111389924
1366	gene-LOC11138995	48.8576321624333	30.125766366266	1.1143826859	0.975962186	0.697586004109	protein_codin	XM_023014946.1	nodulin-26-like
1367	gene-LOC11138997	362.0555363686	307.05249476256	0.7684196438	0.955560538	0.237725687100	protein_codin	XM_023014966.1	phospholipase A1-IIdelta-like
1368	gene-LOC11139000	1678.09076072117	2067.1849640351	-2.511202825	0.999999999	-0.30084673446	protein_codin	XM_023014988.1	DEAD-box ATP-dependent RNA helicase 8-like%2C transcript variant X1
1369	gene-LOC11139005	0.629428869966667	3.0656409012	-0.823559117	0.959496122	-2.28407345722	protein_codin	XM_023015034.1	proteinase inhibitor PSI-1.2-like
1370	gene-LOC11139006	2.57415670776667	0.5	0.7724889366	0.956076955	2.364099883642	protein_codin	XR_002701846.1	bifunctional aspartate aminotransferase and glutamate/aspartate-prephenate aminotransferase%2C transcript variant X3

1371	gene-LOC11139006	8.68678446516667	3.3055614713666	0.7691341862	0.955649260	1.393926898303	protein_codin	XM_023015052.1	cyclin-dependent kinase C-1-like
1372	gene-LOC11139007	93.4099124110667	177.74191845163	-4.327828013	1	-0.92813640692	protein_codin	XM_023015058.1	CMP-sialic acid transporter 5-like
1373	gene-LOC11139007	231.1959553045	457.72951933486	-3.287676077	0.999999999	-0.98537917687	lncRNA	XR_002701847.1	uncharacterized LOC111390073
1374	gene-LOC11139010	12.6422457558333	5.1981714382	0.9893693977	0.973754936	1.282176644692	protein_codin	XM_023015099.1	pre-mRNA-processing protein 40A-like%2C transcript variant X4
1375	gene-LOC11139011	12.9102529195333	4.212282597	0.9290104518	0.971261685	1.615843130768	protein_codin	XM_023015112.1	probable strigolactone esterase DAD2
1376	gene-LOC11139015	3.63256503766667	8.5845122408333	-0.738189962	0.950877131	-1.24074753658	protein_codin	XM_023015155.1	solute carrier family 40 member 3%2C chloroplastic
1377	gene-LOC11139018	0.6358831613	2.8852793205666	-0.741086078	0.951338040	-2.18187738055	protein_codin	XM_023015193.1	uncharacterized LOC111390188
1378	gene-LOC11139019	3.67227433673333	8.9052121422666	-0.785015698	0.955856938	-1.27797614088	protein_codin	XR_002701862.1	coiled-coil domain-containing protein 94 homolog%2C transcript variant X2
1379	gene-LOC11139019	7.20083604166667	17.276375729533	-0.748076485	0.952305039	-1.26256427475	protein_codin	XM_023015198.1	long chain acyl-CoA synthetase 4-like
1380	gene-LOC11139020	5.79110668356667	1.1329467819	0.9653599889	0.976182632	2.353758979557	protein_codin	XM_023015204.1	PHD finger protein At1g33420
1381	gene-LOC11139020	1.12146329196667	4.4045326943333	-0.750045364	0.952534831	-1.97360656379	protein_codin	XM_023015208.1	probable prolyl 4-hydroxylase 9
1382	gene-LOC11139021	2.89803651176667	0.5	0.8284192969	0.960368959	2.535075771227	protein_codin	XM_023015221.1	amino acid transporter AVT6A-like%2C transcript variant X2
1383	gene-LOC11139025	0.5	4.2190022168666	-1.113544141	0.974728242	-3.07690184561	protein_codin	XM_023015267.1	coiled-coil domain-containing protein 97
1384	gene-LOC11139025	5.55408331996667	11.915657142833	-0.774648815	0.954776106	-1.10123779254	protein_codin	XM_023015268.1	beta-amylase 3%2C chloroplastic-like
1385	gene-LOC11139028	3.33548595003333	0.7067508191	0.7926189248	0.958606815	2.238623409093	protein_codin	XM_023015301.1	uncharacterized LOC111390282
1386	gene-LOC11139030	6.71166475013333	14.7710508365	-0.854231672	0.953369978	-1.13802990582	protein_codin	XM_023015318.1	probable calcium-binding protein CML29
1387	gene-LOC11139031	4.03389633776667	0.5	1.1151596656	0.976179790	3.012174010348	protein_codin	XR_002701897.1	uncharacterized LOC111390318%2C transcript variant X2
1388	gene-LOC11139032	1.85599181786667	0.5	0.9346285653	0.972602995	1.892190350363	protein_codin	XM_023015343.1	tRNase Z TRZ2%2C chloroplastic%2C transcript variant X1
1389	gene-LOC11139034	8.9560792854	2.0857267647	0.9321898070	0.972029858	2.102317126317	protein_codin	XM_023015375.1	U-box domain-containing protein 9-like
1390	gene-LOC11139035	9.2263576567	22.660761667633	-1.514584818	0.999999426	-1.29636322926	protein_codin	XM_023015378.1	violaxanthin de-epoxidase%2C chloroplastic
1391	gene-LOC11139036	9.6906953339	3.0656409012	0.7972438769	0.959056710	1.660411472191	protein_codin	XM_023015403.1	50S ribosomal protein L12%2C chloroplastic-like
1392	gene-LOC11139037	3.73878435213333	0.9729997351	0.7828810707	0.957446952	1.942057943613	protein_codin	XM_023015414.1	uncharacterized LOC111390376
1393	gene-LOC11139041	3.1792563637	0.5	0.8447531289	0.960688583	2.668689354861	protein_codin	XM_023015446.1	uncharacterized LOC111390412
1394	gene-LOC11139043	0.629428869966667	4.6664794140666	-1.057346958	0.980458740	-2.89021927398	lncRNA	XR_002701917.1	uncharacterized LOC111390431
1395	gene-LOC11139043	4.8492186409	1.0801683049	0.8540443860	0.961001445	2.166496181840	protein_codin	XM_023015475.1	LEAF RUST 10 DISEASE-RESISTANCE LOCUS RECEPTOR-LIKE PROTEIN KINASE-like 1.1%2C transcript variant X1
1396	gene-LOC11139043	8.4174896449	3.5726161952333	0.6887281746	0.951464482	1.236409107185	protein_codin	XM_023015477.1	UDP-glycosyltransferase 74F2-like
1397	gene-LOC11139043	6.7052104588	2.5127285353666	0.7092317692	0.953106023	1.416027798290	protein_codin	XM_023015490.1	LEAF RUST 10 DISEASE-RESISTANCE LOCUS RECEPTOR-LIKE PROTEIN KINASE-like 1.1%2C transcript variant X1
1398	gene-LOC11139046	2.60194097513333	0.5	0.7922052050	0.958561254	2.379588234981	protein_codin	XM_023015512.1	beta-amylase 2%2C chloroplastic-like
1399	gene-LOC11139054	4.6871266861	0.5	1.3532238358	0.962554163	3.228703789256	protein_codin	XM_023015598.1	nardilysin-like
1400	gene-LOC11139055	2.5612481251	0.5	0.8691339282	0.961743624	2.356847023146	protein_codin	XM_023015618.1	haloacid dehalogenase-like hydrolase domain-containing protein At2g33255
1401	gene-LOC11139059	2.46109147383333	0.5	0.9626316258	0.976207875	2.299298280873	protein_codin	XM_023015652.1	S-adenosylmethionine synthase 2-like
1402	gene-LOC11139061	0.629428869966667	4.1051749199666	-1.055143152	0.980972127	-2.70532843875	protein_codin	XM_023015675.1	uncharacterized membrane protein At1g16860-like
1403	gene-LOC11139062	9.5964010511	4.2190022168666	0.7196132163	0.953293249	1.185591606129	protein_codin	XM_023015693.1	NDR1/HIN1-like protein 1
1404	gene-LOC11139063	2.03556706723333	0.5	0.7088504665	0.953093597	2.025430755730	lncRNA	XR_002701949.1	uncharacterized LOC111390636
1405	gene-LOC11139066	0.5	4.0591769555	-1.100087698	0.974137053	-3.02118723416	protein_codin	XM_023015729.1	proline transporter 1-like%2C transcript variant X3
1406	gene-LOC11139067	71.8676536639	151.42367132543	-2.816167319	1	-1.07517626163	lncRNA	XR_002701967.1	uncharacterized LOC111390673
1407	gene-LOC11139068	7.7071542255	14.076994341933	-0.821933350	0.959534522	-0.86906916325	protein_codin	XM_023015749.1	phospholipase A I-like
1408	gene-LOC11139068	8.53760109306667	2.1928344418	0.8544096339	0.961015925	1.961033863168	protein_codin	XM_023015757.1	bifunctional purple acid phosphatase 26-like%2C transcript variant X2
1409	gene-LOC11139071	5.79110668356667	12.636969576466	-0.807810461	0.958746239	-1.12573955832	protein_codin	XM_023015804.1	phosphopantothenoylcysteine decarboxylase subunit VHS3-like
1410	gene-LOC11139077	2.7636413018	10.0254037069	-0.996004711	0.976224728	-1.85901804997	protein_codin	XM_023015886.1	membrane steroid-binding protein 2-like
1411	gene-LOC11139078	0.938432989266667	8.8024066615	-1.107571019	0.974324633	-3.22957238894	protein_codin	XM_023015895.1	heat stress transcription factor B-2b-like
1412	gene-LOC11139082	2.7775334355	0.8599173533333	0.7367599939	0.953371484	1.691534364496	protein_codin	XM_023015943.1	uncharacterized LOC111390827
1413	gene-LOC11139085	4.37068472446667	0.5	1.4824532551	0.967821793	3.127859314327	protein_codin	XM_023015981.1	crocetin glucosyltransferase%2C chloroplastic-like
1414	gene-LOC11139088	54.8579779422	23.1132859762	2.4376188073	0.999999999	1.246979065753	protein_codin	XM_023016013.1	receptor-like protein 12
1415	gene-LOC11139088	4.67037142196667	0.5	1.0958473132	0.971021879	3.223537288119	protein_codin	XM_023016016.1	developmentally-regulated G-protein 3-like
1416	gene-LOC11139089	0.629428869966667	4.0327877169666	-1.000503533	0.978486103	-2.67966220806	lncRNA	XR_002702020.1	uncharacterized LOC111390892%2C transcript variant X2
1417	gene-LOC11139092	8.52469251036667	1.4928641352333	1.1409146347	0.981893983	2.513564923849	protein_codin	XM_023016046.1	uncharacterized LOC111390922
1418	gene-LOC11139093	2.15854164583333	6.9317619515	-1.088185300	0.974681865	-1.68316518303	protein_codin	XM_023016054.1	probable inactive receptor-like protein kinase At3g56050
1419	gene-LOC11139093	14.7449147613667	34.441994301633	-1.330961435	0.972705731	-1.22395119943	protein_codin	XM_023016055.1	peptide-N4-(N-acetyl-beta-glucosaminyl)asparagine amidase A-like

AQRNA_Control_vs_AQRNA_G7

1420	gene-LOC11139093	0.5	2.5059480228	-1.1346165103	0.9774307300	-2.325356491265	protein_codin	XM_023016062.1	probable methyltransferase PMT23
1421	gene-LOC11139093	11.2260621064	2.5595323076666	1.1978636807	0.9824702880	2.132899823956	protein_codin	XM_023016064.1	non-classical arabinogalactan protein 30
1422	gene-LOC11139094	2.08916849996667	0.5	0.7050067510	0.9529356131	2.062928856341	protein_codin	XM_023016084.1	RPM1-interacting protein 4-like
1423	gene-LOC11139097	9.42588770296667	3.2595635069	0.8021545344	0.9594542081	1.531949709738	protein_codin	XM_023016111.1	V-type proton ATPase subunit a3-like
1424	gene-LOC11139099	30.3781713585667	19.940354719833	0.7026015838	0.9528003061	0.607343953995	protein_codin	XM_023016125.1	cysteine-rich and transmembrane domain-containing protein WIH1-like%2C transcript variant X1
1425	gene-LOC11139101	2.03556706723333	0.5	0.7088504665	0.9530935972	2.025430755730	protein_codin	XM_023016147.1	uncharacterized LOC111391013
1426	gene-LOC11139107	2.3381168952	0.5	0.7562683526	0.9542771787	2.225347059856	protein_codin	XM_023016221.1	transcription initiation factor TFIID subunit 4b-like%2C transcript variant X2
1427	gene-LOC11139108	0.5	2.9329497934333	-1.0623241055	0.9792869654	-2.552352375089	protein_codin	XM_023016223.1	activating signal cointegrator 1 complex subunit 2
1428	gene-LOC11139109	8.41201890456667	18.740128739133	-0.8446734692	0.9563226248	-1.155606866523	protein_codin	XM_023016245.1	uncharacterized LOC111391098%2C transcript variant X1
1429	gene-LOC11139117	66.6414395778667	36.6594549946	1.0668729428	0.9671538292	0.862234227998	protein_codin	XM_023016323.1	SNF1-related protein kinase catalytic subunit alpha KIN10-like%2C transcript variant X7
1430	gene-LOC11139118	31.8909204984333	48.631113845966	-0.8560172661	0.9527526780	-0.608733895319	protein_codin	XM_023016341.1	arginine/serine-rich coiled-coil protein 2-like
1431	gene-LOC11139123	4.586378112	1.4664748967666	0.7898655759	0.9583036711	1.645002923919	protein_codin	XR_002702087.1	uncharacterized LOC111391231%2C transcript variant X2
1432	gene-LOC11139124	779.2035740049	985.3069766236	-1.3839786437	0.9873752038	-0.338572977739	protein_codin	XM_023016412.1	serine protease SPPA%2C chloroplastic-like
1433	gene-LOC11139124	2.69426815593333	0.5	0.8963939323	0.9643118126	2.429893446933	protein_codin	XM_023016418.1	tetratricopeptide repeat protein 4 homolog
1434	gene-LOC11139125	4.98164674886667	12.424244052633	-0.7951335792	0.9571477332	-1.318463446276	protein_codin	XM_023016422.1	uncharacterized LOC111391252%2C transcript variant X1
1435	gene-LOC11139125	3.39366209463333	0.7067508191	0.7139091711	0.9532275709	2.263569368348	protein_codin	XM_023016426.1	probable serine/threonine-protein kinase At1g54610
1436	gene-LOC11139129	2.69426815593333	0.5	0.8963939323	0.9643118126	2.429893446933	protein_codin	XM_023016470.1	protein AIR1-like%2C transcript variant X1
1437	gene-LOC11139133	1.8291911015	12.8760843388	-1.3448415808	0.9752538875	-2.815416220580	protein_codin	XM_023016530.1	uncharacterized LOC111391331
1438	gene-LOC11139134	2.5483395424	0.5	0.7722236578	0.9560425242	2.349557516016	protein_codin	XM_023016539.1	pleiotropic drug resistance protein 2-like%2C transcript variant X2
1439	gene-LOC11139137	13.6173888062	7.4642597835	0.8653397841	0.9615291391	0.867378983560	protein_codin	XM_023016593.1	(+)-neomenthol dehydrogenase-like%2C transcript variant X1
1440	gene-LOC11139138	106.942660758533	128.09697580296	-1.0620115009	0.9793608784	-0.260398939107	protein_codin	XM_023016607.1	BON1-associated protein 2-like
1441	gene-LOC11139139	18.9118863558333	9.3561248351666	0.8347127549	0.9604679890	1.015310255042	protein_codin	XM_023016612.1	uncharacterized LOC111391396
1442	gene-LOC11139143	2.69426815593333	0.5	0.8963939323	0.9643118126	2.429893446933	protein_codin	XM_023016663.1	probable xyloglucan endotransglucosylase/hydrolase protein 23
1443	gene-LOC11139150	1.88279253426667	0.5	0.7311818734	0.9533227219	1.912874037512	protein_codin	XM_023016737.1	uncharacterized LOC111391505
1444	gene-LOC11139151	9.8777084256	2.1393110495666	0.9009974053	0.9650267723	2.207030121867	protein_codin	XM_023016743.1	F-box/LRR-repeat MAX2 homolog A-like
1445	gene-LOC11139153	0.5	2.4055599655666	-0.7612302589	0.9536319638	-2.266372763231	protein_codin	XM_023016766.1	cytochrome b561 and DOMON domain-containing protein At2g04850
1446	gene-LOC11139155	0.992034422	4.6995882724333	-0.8088694424	0.9588506384	-2.244072283053	protein_codin	XM_023016785.1	ATPase GET3-like
1447	gene-LOC11139155	2.0762599173	0.5	0.6996756982	0.9526031653	2.053987059267	protein_codin	XM_023016791.1	homeobox-leucine zipper protein ANTHOCYANINLESS 2-like
1448	gene-LOC11139159	14.9370621911667	26.980212834433	-1.0577505245	0.9803641179	-0.853005301106	protein_codin	XM_023016838.1	alpha%2Calpha-trehalose-phosphate synthase [UDP-forming] 1-like
1449	gene-LOC11139163	10.0180787762333	23.0893141613	-1.0822566827	0.9753721841	-1.204619459076	protein_codin	XM_023016892.1	deacetoxyvindoline 4-hydroxylase-like
1450	gene-LOC11139169	4.18218368143333	0.9729997351	0.7494846777	0.9538025838	2.103745109115	protein_codin	XM_023016946.1	auxin-responsive protein IAA9-like%2C transcript variant X2
1451	gene-LOC11139173	5.56152116233333	1.605946517	0.9240749963	0.9700359428	1.792055688966	protein_codin	XM_023016982.1	probable ADP-ribosylation factor GTPase-activating protein AGD6
1452	gene-LOC11139179	0.5	4.212282597	-1.3779309593	0.9852557182	-3.074602228294	protein_codin	XM_023017061.1	40S ribosomal protein S12
1453	gene-LOC11139181	2.2586982971	0.5	0.7919045808	0.9585282475	2.175491577350	protein_codin	XM_023017072.1	rust resistance kinase Lr10-like%2C transcript variant X1
1454	gene-LOC11139182	5.6076847527	1.55316804	0.9105736353	0.9668586039	1.852191321718	protein_codin	XM_023017088.1	pectin acetylesterase 8-like
1455	gene-LOC11139189	5.93801884806667	0.5	1.4867432554	0.9682126282	3.569981672639	protein_codin	XM_023017172.1	glucosidase 2 subunit beta-like
1456	gene-LOC11139189	43.5525524636333	26.713158110533	1.1408076287	0.9818786306	0.705206726803	protein_codin	XM_023017180.1	receptor-like protein 2
1457	gene-LOC11139195	0.5	3.4654476254666	-1.1236655386	0.9758268246	-2.793041714651	protein_codin	XM_023017240.1	protein ETHYLENE INSENSITIVE 3-like
1458	gene-LOC11139196	18.5479931472333	2.2396991068	1.9562284936	0.9999999999	3.049886267787	protein_codin	XR_002702185.1	transcription factor HBI1-like%2C transcript variant X2
1459	gene-LOC11139197	3.3290316587	0.8863065918666	0.7448962559	0.9535808235	1.909224841981	protein_codin	XR_002702192.1	uncharacterized LOC111391970%2C transcript variant X8
1460	gene-LOC11139197	4.9757843804	14.025949266066	-0.7977669166	0.9575003858	-1.495102540201	protein_codin	XM_023017255.1	glucose-1-phosphate adenylyltransferase large subunit 1-like
1461	gene-LOC11139198	3.11979256253333	0.5	1.1572135605	0.9831134604	2.641450106346	protein_codin	XM_023017269.1	uncharacterized LOC111391981%2C transcript variant X4
1462	gene-LOC11139198	7.4765851533	2.5859215462	0.8546234004	0.9610244159	1.531700979561	protein_codin	XM_023017278.1	uncharacterized LOC111391988
1463	gene-LOC11139208	0.5	2.3000030115333	-0.7461063970	0.9520514212	-2.201635750178	protein_codin	XM_023017362.1	uncharacterized LOC111392083%2C transcript variant X1
1464	gene-LOC11139210	0.9255244066	5.2789507694666	-1.0276950434	0.9848016111	-2.511908271369	lncRNA	XR_002702213.1	uncharacterized LOC111392106
1465	gene-LOC11139217	5.01647723043333	1.1329467819	0.8245484185	0.9603086333	2.146594507180	protein_codin	XM_023017442.1	eukaryotic translation initiation factor 5B-like
1466	gene-LOC11139219	1069.95710718763	1453.6970173988	-3.4233845367	0.9999999999	-0.442173648516	protein_codin	XM_023017465.1	uncharacterized LOC111392190
1467	gene-LOC11139224	29.5124471553667	11.250468203133	1.4628234366	0.9650779190	1.391338510827	protein_codin	XM_023017532.1	L-galactono-1%2C4-lactone dehydrogenase%2C mitochondrial
1468	gene-LOC11139228	16.3574289243667	4.8536215072333	0.9400502782	0.9737741611	1.752812487533	protein_codin	XM_023017588.1	uncharacterized LOC111392282

1469	gene-LOC11139229	5.73105095946667	12.104410851733	-0.741400325	0.951385887	-1.0786612323	protein_codin	XM_023017601.1	probable galactinol--sucrose galactosyltransferase 1%2C transcript variant X1
1470	gene-LOC11139231	144.802014911433	181.86542453143	-0.754925945	0.953055825	-0.3287896125	protein_codin	XM_023017623.1	pentatricopeptide repeat-containing protein At3g48250%2C chloroplastic-like
1471	gene-LOC11139231	5.26680080483333	1.1329467819	0.6846195482	0.950869079	2.216846801700	protein_codin	XM_023017629.1	zinc finger CCCH domain-containing protein 32-like%2C transcript variant X1
1472	gene-LOC11139235	0.938432989266667	4.1587592048333	-0.802206295	0.958084104	-2.1478275190	protein_codin	XM_023017685.1	peptide deformylase 1B%2C chloroplastic%2C transcript variant X2
1473	gene-LOC11139242	3.35883161303333	0.5	0.7748239116	0.956383475	2.747959471329	protein_codin	XM_023017775.1	uncharacterized LOC111392429%2C transcript variant X2
1474	gene-LOC11139243	2.25224400576667	0.5	0.7304862915	0.953319521	2.171363135961	protein_codin	XM_023017879.1	65-kDa microtubule-associated protein 3-like%2C transcript variant X2
1475	gene-LOC11139245	2.00974990186667	0.5	0.7043739485	0.952899766	2.007015980117	protein_codin	XM_023017806.1	ras-related protein RABF2a-like
1476	gene-LOC11139246	6.3467879905	1.1329467819	0.9375650162	0.973258199	2.485946556530	protein_codin	XM_023017818.1	uncharacterized LOC111392469
1477	gene-LOC11139251	2.7537319571	0.5	0.6975232012	0.952426558	2.461388137184	protein_codin	XM_023017875.1	phospholipase A1-Igamma3%2C chloroplastic-like
1478	gene-LOC11139251	64.2642538914333	37.886009321166	1.2340557476	0.985058203	0.762351298111	protein_codin	XM_023017877.1	photosystem II 5 kDa protein%2C chloroplastic-like
1479	gene-LOC11139253	12.4468987933667	1.6867258483	1.6200732654	0.979355512	2.883488922835	lncRNA	XR_002702277.1	uncharacterized LOC111392535
1480	gene-LOC11139254	3.72489221846667	0.5	1.2200150692	0.983824698	2.897198681109	protein_codin	XM_023017905.1	PRA1 family protein B4-like
1481	gene-LOC11139254	18.5063167461333	9.4904275586	1.0396062606	0.967854240	0.963472799243	protein_codin	XM_023017906.1	high mobility group B protein 1-like
1482	gene-LOC11139254	2.2586982971	0.5	0.7919045808	0.958528247	2.175491577350	protein_codin	XM_023017910.1	BAG family molecular chaperone regulator 4-like%2C transcript variant X1
1483	gene-LOC11139256	4.30417470906667	17.007587604533	-1.323931651	0.971796644	-1.9823699762	protein_codin	XM_023017927.1	bifunctional monothiol glutaredoxin-S16%2C chloroplastic
1484	gene-LOC11139259	9.75203871456667	18.903359910133	-0.804607815	0.958382404	-0.9548669254	protein_codin	XM_023017946.1	chromosome transmission fidelity protein 18 homolog
1485	gene-LOC11139259	7.3633196246	1.1797505542	1.0425929836	0.967661216	2.641874477714	protein_codin	XM_023017954.1	transcription factor bHLH128-like%2C transcript variant X2
1486	gene-LOC11139264	3.941569157	1.0801683049	0.7081875182	0.953072081	1.867513966242	protein_codin	XM_023018025.1	1-aminocyclopropane-1-carboxylate oxidase homolog 1-like
1487	gene-LOC11139264	2.64066672316667	0.5	0.7971634153	0.959049456	2.400902231389	protein_codin	XM_023018026.1	1-aminocyclopropane-1-carboxylate oxidase homolog 1-like
1488	gene-LOC11139265	2.6387871437	0.5	0.6810383823	0.950273286	2.399874981163	protein_codin	XM_023018027.1	uncharacterized LOC111392650
1489	gene-LOC11139265	8.50858439496667	0.6531665342333	1.6655062578	0.971458296	3.703396345598	protein_codin	XM_023018030.1	RING-H2 finger protein ATL16-like
1490	gene-LOC11139269	8.63416658343333	2.9057547471	1.0715427086	0.967382707	1.571143986654	protein_codin	XM_023018090.1	UDP-glucuronate 4-epimerase 3-like
1491	gene-LOC11139275	47.8212009573333	70.3034474634	-1.445763687	0.999389821	-0.5559450747	protein_codin	XM_023018150.1	remorin 4.1-like
1492	gene-LOC11139279	6.07591769633333	1.605946517	0.7542039654	0.954114959	1.919678479959	protein_codin	XM_023018187.1	heterogeneous nuclear ribonucleoprotein Q-like%2C transcript variant X1
1493	gene-LOC11139283	2.0762599173	6.0513691716333	-0.761561932	0.953660647	-1.5432745415	protein_codin	XR_002702340.1	casein kinase 1-like protein 3%2C transcript variant X3
1494	gene-LOC11139284	2.66845099056667	0.5	0.8904698014	0.963535251	2.416002514674	protein_codin	XM_023018241.1	U1 small nuclear ribonucleoprotein 70 kDa-like
1495	gene-LOC11139285	1.85599181786667	0.5	0.9346285653	0.972602995	1.892190350363	protein_codin	XM_023018251.1	AT-rich interactive domain-containing protein 5-like
1496	gene-LOC11139287	3.48180619166667	0.5	0.8762433299	0.962211728	2.799835900259	protein_codin	XM_023018280.1	4-alpha-glucanotransferase DPE2-like%2C transcript variant X5
1497	gene-LOC11139289	1.85599181786667	0.5	0.9346285653	0.972602995	1.892190350363	protein_codin	XM_023018299.1	non-classical arabinogalactan protein 31-like
1498	gene-LOC11139289	4.6445542566	0.9729997351	0.7564162134	0.954290259	2.255028828747	protein_codin	XM_023018305.1	protein SMG7L-like%2C transcript variant X1
1499	gene-LOC11139293	2.08916849996667	0.5	0.7050067510	0.952935613	2.062928856341	protein_codin	XM_023018347.1	CBL-interacting protein kinase 2-like
1500	gene-LOC11139293	4.36363851023333	1.1329467819	0.6946511274	0.952157537	1.945451496993	protein_codin	XM_023018349.1	probable WRKY transcription factor 70
1501	gene-LOC11139296	862.5607617243	994.63318452526	-1.278657235	0.973513741	-0.2055384766	protein_codin	XM_023018378.1	MDIS1-interacting receptor like kinase 2-like
1502	gene-LOC11139298	2.25224400576667	0.5	0.7304862915	0.953319521	2.171363135961	protein_codin	XM_023018389.1	receptor-like serine/threonine-protein kinase NCRK
1503	gene-LOC11139298	5.2847884997	10.663458492933	-0.755180281	0.953080541	-1.0127577847	protein_codin	XM_023018392.1	double-stranded RNA-binding protein 2
1504	gene-LOC11139299	2.4185190443	0.5	0.7545467141	0.954141492	2.274123898134	protein_codin	XM_023018404.1	uncharacterized mitochondrial protein AtMg00810-like
1505	gene-LOC11139302	12.2334766134333	3.6848927691333	1.1151322018	0.976172077	1.731139917054	protein_codin	XM_023018424.1	probable methionine--tRNA ligase
1506	gene-LOC11139302	11.3064642555	5.3853135313333	0.7308735930	0.953321106	1.070045596323	protein_codin	XM_023018429.1	elongation factor 1-alpha-like
1507	gene-LOC11139303	1.96905705183333	0.5	0.6815674662	0.950366313	1.977504912765	protein_codin	XM_023018436.1	GATA transcription factor 1-like
1508	gene-LOC11139304	148.61574264	309.84371242396	-2.386507616	0.999999999	-1.0599537459	protein_codin	XM_023018448.1	uncharacterized LOC111393044
1509	gene-LOC11139304	0.9255244066	4.0599218706666	-0.736075430	0.950519105	-2.1331090239	protein_codin	XM_023018454.1	pentatricopeptide repeat-containing protein At2g29760%2C chloroplastic-like
1510	gene-LOC11139306	3.35583237506667	0.5	1.2082729630	0.982905940	2.746670654484	protein_codin	XM_023018468.1	pyruvate kinase isozyme A%2C chloroplastic
1511	gene-LOC11139312	29.5112147247	9.3585422587666	1.5564580833	0.961652396	1.656907575616	protein_codin	XM_023018519.1	protein BRASSINAZOLE-RESISTANT 1-like
1512	gene-LOC11139313	4.82241792453333	0.7331400576333	1.0425583634	0.967663237	2.717595945016	protein_codin	XM_023018523.1	TMV resistance protein N-like
1513	gene-LOC11139317	18.3238783663333	8.7972377646333	1.0828130446	0.968546286	1.058602381732	protein_codin	XM_023018573.1	zinc finger protein CONSTANS-LIKE 4-like
1514	gene-LOC11139321	19.2851292829667	11.783710950266	0.8087783585	0.959849530	0.710694870300	protein_codin	XM_023018652.1	trans-cinnamate 4-monooxygenase
1515	gene-LOC11139322	13.3758782533	6.0036986987666	0.6960518026	0.952295255	1.155710140351	protein_codin	XM_023018663.1	tubulin beta-1 chain-like
1516	gene-LOC11139322	7.0946167272	1.9325602304666	0.8760165479	0.962195157	1.876211369507	protein_codin	XM_023018662.1	pathogenesis-related genes transcriptional activator PTI6-like
1517	gene-LOC11139324	0.5	2.3000030115333	-0.746106397	0.952051421	-2.2016357501	protein_codin	XM_023018684.1	uncharacterized LOC111393244

AQRNA_Control_vs_AQRNA_G7

1518	gene-LOC11139325	12.7216643539333	6.0521749795333	0.8157012004	0.9601173227	1.071761824090	protein_codin	XM_023018704.1	sec-independent protein translocase protein TATB%2C chloroplastic%2C transcript variant X2
1519	gene-LOC11139325	5.80499881723333	1.55316804	0.7894914006	0.9582594405	1.902081847242	protein_codin	XM_023018700.1	putative uncharacterized protein MYH16%2C transcript variant X2
1520	gene-LOC11139325	0.5	2.1928344418	-0.7541193844	0.9529778007	-2.132796893174	protein_codin	XM_023018703.1	uncharacterized LOC111393257
1521	gene-LOC11139325	132.6733595041	108.0814215142	0.9420577548	0.9741607310	0.295760155455	protein_codin	XM_023018705.1	histone deacetylase 19-like%2C transcript variant X1
1522	gene-LOC11139326	1.96905705183333	0.5	0.6815674662	0.9503663139	1.977504912765	protein_codin	XM_023018723.1	scarecrow-like protein 8%2C transcript variant X2
1523	gene-LOC11139328	2.73496100596667	0.5	0.7931074243	0.9586591417	2.451520263767	protein_codin	XM_023018742.1	UPF0061 protein azo1574-like
1524	gene-LOC11139328	2.49434648156667	0.5	0.9009346311	0.9650159627	2.318661879168	protein_codin	XM_023018745.1	linoleate 13S-lipoxygenase 2-1%2C chloroplastic-like
1525	gene-LOC11139328	0.5	2.8852793205666	-0.8592127622	0.9516079860	-2.528710991276	protein_codin	XM_023018749.1	uncharacterized LOC111393288%2C transcript variant X2
1526	gene-LOC11139331	1.43046741126667	5.9262643844	-0.8278876552	0.9592815189	-2.050636358973	protein_codin	XM_023018775.1	multiple organellar RNA editing factor 2%2C chloroplastic-like
1527	gene-LOC11139333	2.6387871437	0.5	0.6810383823	0.9502732869	2.399874981163	protein_codin	XM_023018823.1	myosin-6-like
1528	gene-LOC11139334	5.6294063569	0.5	1.1701378869	0.9829958578	3.492982792445	protein_codin	XM_023018844.1	transcription factor MYB4-like
1529	gene-LOC11139335	2.00974990186667	0.5	0.7043739485	0.9528997665	2.007015980117	protein_codin	XM_023018863.1	cyclin-dependent kinase inhibitor 5-like
1530	gene-LOC11139336	0.6358831613	4.3314005762	-0.7867885801	0.9560703339	-2.767999991049	protein_codin	XM_023018870.1	sm-like protein LSM5
1531	gene-LOC11139336	3.77144743696667	10.772238678466	-0.7754597663	0.9548515031	-1.514127876634	protein_codin	XM_023018876.1	uncharacterized LOC111393369
1532	gene-LOC11139337	5.23862490933333	2.0525570136333	0.6869204573	0.9512117011	1.351765871199	protein_codin	XM_023018877.1	uncharacterized LOC111393370
1533	gene-LOC11139338	5.1989156103	1.2333348391333	0.9384355620	0.9734380469	2.075646205900	protein_codin	XM_023018890.1	probable 3-ketoacyl-CoA synthase 21
1534	gene-LOC11139338	2.81724273453333	0.8599173533333	0.7294514271	0.9533170126	1.712013957980	protein_codin	XM_023018897.1	ethylene-responsive transcription factor CRF4-like
1535	gene-LOC11139341	1591.5591194542	1467.6698141693	1.0925226854	0.9702848883	0.116913310274	protein_codin	XM_023018943.1	putative MO25-like protein At4g17270%2C transcript variant X1
1536	gene-LOC11139342	3.10402084933333	0.5	0.7724037531	0.9560658904	2.634138247966	protein_codin	XM_023018962.1	mediator of RNA polymerase II transcription subunit 33A-like
1537	gene-LOC11139343	34.8066405995	20.874331784566	0.9199477673	0.9690181393	0.737632561793	protein_codin	XM_023018972.1	stem-specific protein TSJT1-like
1538	gene-LOC11139345	107.2803774703	82.620583838933	0.8249470031	0.9603149399	0.376813058386	lncRNA	XR_002702422.1	uncharacterized LOC111393454
1539	gene-LOC11139350	0.5	2.5127285353666	-0.8011586181	0.9579476842	-2.329254816926	protein_codin	XM_023019049.1	auxin response factor 22-like
1540	gene-LOC11139350	2.3058454385	7.1452323904333	-0.8328714354	0.9587477629	-1.631685122118	protein_codin	XM_023019065.1	REF/SRPP-like protein At1g67360%2C transcript variant X2
1541	gene-LOC11139350	210.7193653641	132.39108710993	2.6783095331	1	0.670516905989	protein_codin	XM_023019064.1	D-inositol 3-phosphate glycosyltransferase 1%2C transcript variant X2
1542	gene-LOC11139353	3.97482416473333	10.983352586533	-1.0630394183	0.9791198694	-1.466355547409	protein_codin	XM_023019102.1	abscisic acid 8'-hydroxylase 2-like%2C transcript variant X2
1543	gene-LOC11139354	4.08104347916667	0.5	1.2745233897	0.9839395751	3.028938081055	protein_codin	XM_023019122.1	uncharacterized LOC111393541
1544	gene-LOC11139355	1.96905705183333	7.5186498762333	-0.9725802952	0.9608452046	-1.932968707683	protein_codin	XM_023019142.1	uncharacterized LOC111393553
1545	gene-LOC11139355	28.7349222751	10.0765705682	1.2642743274	0.9851079079	1.511800427259	protein_codin	XM_023019149.1	methionine aminopeptidase 2B-like%2C transcript variant X1
1546	gene-LOC11139356	5.68808690183333	12.582457698266	-0.7381783286	0.9508752105	-1.145398336017	protein_codin	XM_023019152.1	potassium transporter 4-like%2C transcript variant X1
1547	gene-LOC11139357	2.14563306316667	0.5	0.7875947307	0.9580373793	2.101403373758	protein_codin	XM_023019172.1	cytochrome P450 71A4-like
1548	gene-LOC11139357	4.65973404686667	1.1797505542	0.7710767010	0.9558945552	1.981765766581	protein_codin	XM_023019184.1	cysteine synthase%2C chloroplastic/chromoplastic%2C transcript variant X3
1549	gene-LOC11139359	7.42396727153333	3.0656409012	0.7387891276	0.9534053337	1.276001635897	protein_codin	XM_023019207.1	uncharacterized protein At3g49140
1550	gene-LOC11139360	61.8260289299	44.680123532333	1.2678412553	0.9847622881	0.468581170025	lncRNA	XR_002702453.1	uncharacterized LOC111393608
1551	gene-LOC11139362	0.5	2.1928344418	-0.7541193844	0.9529778007	-2.132796893174	protein_codin	XM_023019245.1	probable magnesium transporter NIPA1%2C transcript variant X4
1552	gene-LOC11139363	0.6358831613	3.8125367654333	-0.9901222337	0.9728564374	-2.583917639250	protein_codin	XM_023019264.1	probable leucine-rich repeat receptor-like protein kinase At2g33170%2C transcript variant X3
1553	gene-LOC11139363	2.0762599173	0.5	0.6996756982	0.9526031653	2.053987059267	protein_codin	XM_023019269.1	uncharacterized LOC111393634
1554	gene-LOC11139368	2.44531976066667	0.5	0.6834549292	0.9506825516	2.290023130602	protein_codin	XM_023019349.1	gamma-secretase subunit APH1-like
1555	gene-LOC11139369	2.78398772683333	0.8863065918666	0.7611469672	0.9547350010	1.651275102679	protein_codin	XM_023019354.1	serine hydroxymethyltransferase 2%2C mitochondrial-like
1556	gene-LOC11139369	1.1279175833	6.4527875116666	-1.0278740810	0.9848046966	-2.516260864589	protein_codin	XM_023019357.1	polypyrimidine tract-binding protein homolog 2%2C transcript variant X1
1557	gene-LOC11139369	4.6206166707	13.169467408466	-1.0962255011	0.9741955257	-1.511039689502	protein_codin	XM_023019355.1	inositol-tetrakisphosphate 1-kinase 3-like
1558	gene-LOC11139369	53.0265156330667	78.268357863433	-0.7924166602	0.9567864147	-0.561715223978	lncRNA	XR_002702478.1	uncharacterized LOC111393697%2C transcript variant X2
1559	gene-LOC11139369	6.49050062226667	1.0801683049	0.9707956126	0.9759347678	2.587073638402	protein_codin	XM_023019364.1	leucine-rich repeat-containing protein sog2%2C transcript variant X2
1560	gene-LOC11139369	89.6474716493333	58.038967779433	1.1042060978	0.9731352844	0.627241033605	lncRNA	XR_002702481.1	uncharacterized LOC111393699%2C transcript variant X3
1561	gene-LOC11139371	0.662683877666667	3.0452263673666	-0.7999300656	0.9577877031	-2.200156749223	protein_codin	XM_023019380.1	GDP-mannose 3%2C5-epimerase 2-like%2C transcript variant X1
1562	gene-LOC11139371	87.2332784372333	47.0523919614	1.3240093109	0.9734635550	0.890610544813	protein_codin	XM_023019383.1	CBL-interacting serine/threonine-protein kinase 7-like
1563	gene-LOC11139371	1.81628251883333	7.5186498762333	-1.0114025222	0.9825277837	-2.049484992258	protein_codin	XR_002702486.1	adenine DNA glycosylase%2C transcript variant X3
1564	gene-LOC11139373	0.5	2.1861148219	-1.0350065102	0.9845091010	-2.128369178061	protein_codin	XM_023019414.1	GPN-loop GTPase 3%2C transcript variant X1
1565	gene-LOC11139375	9.31868483746667	2.1928344418	1.0018276819	0.9718892197	2.087329465959	protein_codin	XM_023019448.1	serine/threonine-protein kinase OXI1-like
1566	gene-LOC11139377	2.69426815593333	0.7067508191	0.6954088369	0.9522318521	1.930619891729	protein_codin	XM_023019472.1	protein DETOXIFICATION 48-like

1567	gene-LOC11139378	4.9757843804	1.7131150868	0.7623543958	0.954860567	1.538301894676	protein_codin	XM_023019487.1	cellulose synthase A catalytic subunit 2 [UDP-forming]-like%2C transcript variant X1
1568	gene-LOC11139384	4.94996721503333	0.9729997351	0.9207077793	0.969203632	2.346907652521	protein_codin	XM_023019585.1	vacuolar protein sorting-associated protein 26A-like
1569	gene-LOC11139384	113.4470535145	164.29256428616	-1.031341012	0.984751199	-0.53424804752	protein_codin	XM_023019583.1	P-loop NTPase domain-containing protein LPA1 homolog 1-like
1570	gene-LOC11139385	25.8550485033333	49.563418402233	-1.340964973	0.974435506	-0.93882968269	protein_codin	XM_023019590.1	phosphomethylpyrimidine synthase%2C chloroplastic%2C transcript variant X6
1571	gene-LOC11139385	1265.48808030597	1654.6651434202	-0.838647001	0.957756108	-0.38684536793	lncRNA	XR_002702502.1	uncharacterized LOC111393851
1572	gene-LOC11139385	16.2405722348	4.1059807277666	1.4709247503	0.966465813	1.983803705621	protein_codin	XM_023019605.1	probable inactive receptor-like protein kinase At3g56050
1573	gene-LOC11139386	3.32715207923333	0.5	0.9823641090	0.974745431	2.734287811198	protein_codin	XM_023019608.1	40S ribosomal protein S4
1574	gene-LOC11139391	0.5	2.9857282704666	-1.042489693	0.983556510	-2.57808287254	protein_codin	XM_023019682.1	ribose-phosphate pyrophosphokinase 2%2C chloroplastic-like
1575	gene-LOC11139391	4.56316855646667	0.7331400576333	0.9738924541	0.975695591	2.637875206294	protein_codin	XM_023019691.1	pentatricopeptide repeat-containing protein At1g31920%2C transcript variant X2
1576	gene-LOC11139392	4.64643383606667	1.7131150868	0.7112489927	0.953167964	1.439501789716	protein_codin	XM_023019709.1	protein WVD2-like 5%2C transcript variant X2
1577	gene-LOC11139394	6.11301938553333	1.0801683049	1.0786224212	0.968005758	2.500629020772	protein_codin	XM_023019738.1	eukaryotic translation initiation factor 3 subunit J-A-like
1578	gene-LOC11139395	6.85024304386667	1.7131150868	0.7851809789	0.957740463	1.999533100518	protein_codin	XM_023019742.1	protein TIFY 8-like
1579	gene-LOC11139395	3.0643115503	0.5	0.8320601681	0.960424077	2.615562984346	lncRNA	XR_002702524.1	uncharacterized LOC111393955
1580	gene-LOC11139395	4.8685815149	1.3133083625333	0.7454104582	0.953603200	1.890295798947	protein_codin	XM_023019752.1	probable histone H2B.1
1581	gene-LOC11139396	13.4155875523667	6.0241741253	0.7861253636	0.957856947	1.155074864610	protein_codin	XM_023019753.1	50S ribosomal protein L9%2C chloroplastic
1582	gene-LOC11139399	0.5	7.0109296670333	-1.745038783	0.989700998	-3.80960576193	protein_codin	XM_023019791.1	endoglucanase 6-like
1583	gene-LOC11139413	2.1188323468	0.5	0.7806661116	0.957158201	2.083269438578	protein_codin	XM_023019915.1	stomatin-like protein 2%2C mitochondrial
1584	gene-LOC11139416	25.2221645800333	41.265720982733	-0.863534472	0.950012832	-0.71025175308	protein_codin	XM_023019933.1	uncharacterized LOC111394166
1585	gene-LOC11139418	0.5	2.1928344418	-0.754119384	0.952977800	-2.13279689317	protein_codin	XM_023019951.1	uncharacterized LOC111394183
1586	gene-LOC11139418	10.4342857609667	3.6253946722666	0.8420879309	0.960619348	1.525121884450	protein_codin	XM_023019952.1	polyadenylate-binding protein 2-like
1587	gene-LOC11139419	9.7239503416	3.4654476254666	0.7496815840	0.953813485	1.488500811159	protein_codin	XM_023019955.1	subtilisin-like protease SBT1.9
1588	gene-LOC11139427	6.11947367686667	0.5	1.6682839441	0.970257913	3.613407575070	protein_codin	XM_023020033.1	UDP-glucuronic acid decarboxylase 2-like
1589	gene-LOC11139428	14.4081263747	4.3714238359333	1.0307737465	0.968534410	1.720707572380	protein_codin	XM_023020040.1	uncharacterized LOC111394287
1590	gene-LOC11139431	2.72393200276667	0.5	0.9684666577	0.976072516	2.445690689952	protein_codin	XM_023020062.1	uncharacterized LOC111394313
1591	gene-LOC11139431	2.08916849996667	0.5	0.7050067510	0.952935613	2.062928856341	protein_codin	XM_023020068.1	pentatricopeptide repeat-containing protein At2g13420%2C mitochondrial-like
1592	gene-LOC11139432	3.63256503766667	0.5	1.0317381756	0.968454409	2.860988628189	protein_codin	XM_023020084.1	UPF0481 protein At3g47200-like
1593	gene-LOC11139435	5.84372456526667	1.339697601	1.0306864032	0.968542226	2.124980790931	protein_codin	XM_023020122.1	IAA-amino acid hydrolase ILR1-like 6%2C transcript variant X2
1594	gene-LOC11139436	1.96905705183333	0.5	0.6815674662	0.950366313	1.977504912765	protein_codin	XM_023020141.1	pentatricopeptide repeat-containing protein At3g02650%2C mitochondrial-like
1595	gene-LOC11139436	29.8288891170333	58.729238805466	-1.534793134	0.999999966	-0.97736868902	lncRNA	XR_002702576.1	uncharacterized LOC111394364
1596	gene-LOC11139438	12.0222704152	1.8270032764333	1.1811619320	0.982623545	2.718158249549	protein_codin	XM_023020174.1	protein SENSITIVE TO PROTON RHIZOTOXICITY 1-like%2C transcript variant X1
1597	gene-LOC11139439	9.07717428456667	3.5793358151	0.7650628371	0.955159991	1.342551353373	protein_codin	XM_023020205.1	ADP-ribosylation factor%2C transcript variant X1
1598	gene-LOC11139440	2.74786958863333	0.5	0.7977912973	0.959106193	2.458313536799	protein_codin	XM_023020202.1	carboxymethylenebutenolidase homolog
1599	gene-LOC11139440	0.938432989266667	6.0785642180333	-1.239326414	0.982614838	-2.69540495813	protein_codin	XM_023020207.1	nuclear cap-binding protein subunit 2
1600	gene-LOC11139440	3.3890873828	0.9729997351	0.7137132578	0.953224663	1.800385517797	protein_codin	XM_023020208.1	putative methylesterase 14%2C chloroplastic
1601	gene-LOC11139440	2.76821601366667	0.5	0.7280483209	0.953313645	2.468956525953	protein_codin	XM_023020209.1	elongation factor 1-gamma 2-like
1602	gene-LOC11139442	0.5	2.4055599655666	-0.761230258	0.953631963	-2.26637276323	protein_codin	XM_023020247.1	bifunctional nuclease 1-like
1603	gene-LOC11139444	4.06168060513333	0.9398908767333	0.9022165139	0.965237407	2.111511624371	protein_codin	XM_023020266.1	SKP1-like protein 1A
1604	gene-LOC11139444	6.4617328039	2.1928344418	0.7142546798	0.953232709	1.559124202452	protein_codin	XM_023020267.1	GDSL esterase/lipase EXL3-like
1605	gene-LOC11139444	3.85086603503333	0.7067508191	0.7726778741	0.956101525	2.445909379939	protein_codin	XM_023020276.1	E3 ubiquitin-protein ligase ZNF598
1606	gene-LOC11139447	1.3843038209	11.567762194966	-1.246050261	0.981337445	-3.06287728022	protein_codin	XM_023020307.1	protein GDAP2 homolog
1607	gene-LOC11139447	7.34258157143333	17.008210734266	-0.764342199	0.953893980	-1.21187208411	protein_codin	XR_002702599.1	uncharacterized LOC111394475%2C transcript variant X16
1608	gene-LOC11139448	231.141105432667	193.53850010963	0.7179498997	0.953279457	0.256153260644	lncRNA	XR_002702611.1	uncharacterized LOC111394481
1609	gene-LOC11139449	2.15854164583333	0.5	0.9576639473	0.976078277	2.110056927711	lncRNA	XR_002702620.1	uncharacterized LOC111394496%2C transcript variant X2
1610	gene-LOC11139452	62.9803556014667	49.9319401481	0.7315899810	0.953324866	0.334938938379	protein_codin	XM_023020378.1	CASP-like protein 5C1
1611	gene-LOC11139452	54.7724091583333	37.511846920233	0.9040715817	0.965567171	0.546103041764	lncRNA	XR_002702628.1	uncharacterized LOC111394522%2C transcript variant X2
1612	gene-LOC11139452	52.7786060147333	34.284403785666	0.9132777815	0.967450544	0.622400813200	lncRNA	XR_002702629.1	uncharacterized LOC111394523%2C transcript variant X1
1613	gene-LOC11139452	7.80307256713333	2.3000030115333	0.7332102810	0.953333452	1.762406566682	protein_codin	XM_023020379.1	heterodimeric geranylgeranyl pyrophosphate synthase small subunit%2C chloroplastic-like
1614	gene-LOC11139454	51.5951521933333	31.220739856466	0.6851263783	0.950950776	0.724730792330	protein_codin	XM_023020408.1	pollen-specific leucine-rich repeat extensin-like protein 3
1615	gene-LOC11139456	0.5	2.8333675441	-0.853196542	0.953722240	-2.50251776008	protein_codin	XM_023020430.1	auxin-responsive protein SAUR50-like

1616	gene-LOC11139457	2.08916849996667	0.5	0.7050067510	0.952935613	2.062928856341	protein_codin	XM_023020444.1	bifunctional phosphatase IMPL2%2C chloroplastic-like
1617	gene-LOC11139457	14.5531266630667	4.5321766906	0.9380397581	0.973356159	1.683053129901	protein_codin	XM_023020475.1	transmembrane protein 205-like%2C transcript variant X2
1618	gene-LOC11139460	2.66845099056667	7.1980108675	-0.830678395	0.959018161	-1.43159576575	protein_codin	XM_023020509.1	ATP-dependent Clp protease proteolytic subunit 6%2C chloroplastic%2C transcript variant X2
1619	gene-LOC11139461	78.5739028995667	117.9684325656	-2.788800015	0.999999999	-0.58627872884	protein_codin	XM_023020520.1	agamous-like MADS-box protein AGL29
1620	gene-LOC11139462	7.22990796563333	1.605946517	0.9037379441	0.965505188	2.170553434820	protein_codin	XM_023020532.1	U-box domain-containing protein 26-like
1621	gene-LOC11139462	2.74786958863333	0.5	0.7977912973	0.959106193	2.458313536799	protein_codin	XM_023020536.1	sodium/proton antiporter 2-like%2C transcript variant X1
1622	gene-LOC11139463	3.17339399523333	0.5	0.9806593731	0.974964276	2.666026651316	protein_codin	XM_023020546.1	3-oxo-Delta(4%2C5)-steroid 5-beta-reductase-like
1623	gene-LOC11139464	0.992034422	5.9202896796666	-0.859730421	0.951419631	-2.57720568283	protein_codin	XM_023020555.1	ATP-dependent Clp protease proteolytic subunit-related protein 1%2C chloroplastic-like
1624	gene-LOC11139464	5.3697653607	0.9729997351	0.7498706143	0.953823968	2.464347731695	protein_codin	XM_023020562.1	uncharacterized LOC111394649
1625	gene-LOC11139466	9.5298910357	3.7853417190666	0.7996290628	0.959261081	1.332036174154	protein_codin	XM_023020584.1	uncharacterized LOC111394661
1626	gene-LOC11139466	2.46109147383333	0.5	0.9626316258	0.976207875	2.299298280873	protein_codin	XM_023020585.1	DNA polymerase V-like%2C transcript variant X1
1627	gene-LOC11139466	189.333462943333	234.22154488503	-0.749208654	0.952437646	-0.30694437172	protein_codin	XM_023020588.1	MADS-box transcription factor ANR1-like
1628	gene-LOC11139469	3.449534735	0.5	0.9993502027	0.972252765	2.786401787753	protein_codin	XM_023020643.1	phytoene dehydrogenase%2C chloroplastic/chromoplastic
1629	gene-LOC11139473	1.06786185923333	3.732563242	-0.756203411	0.953180517	-1.80544167545	protein_codin	XR_002702701.1	PRA1 family protein H-like%2C transcript variant X5
1630	gene-LOC11139474	4.30417470906667	0.6531665342333	1.2603128001	0.985409870	2.720213859247	protein_codin	XM_023020719.1	ribosome biogenesis protein BRX1 homolog 2-like
1631	gene-LOC11139475	2.8702522444	0.6531665342333	0.7391955350	0.953412562	2.135654750142	protein_codin	XM_023020724.1	WD repeat-containing protein 44-like
1632	gene-LOC11139475	15.4585049393667	24.686123634866	-0.776430424	0.954942463	-0.67529951695	protein_codin	XM_023020734.1	uncharacterized protein YnbB-like%2C transcript variant X6
1633	gene-LOC11139479	3.8528331371	0.9729997351	0.6901414878	0.951647750	1.985408388120	protein_codin	XM_023020783.1	uncharacterized LOC111394790%2C transcript variant X2
1634	gene-LOC11139480	1.96905705183333	0.5	0.6815674662	0.950366313	1.977504912765	protein_codin	XM_023020800.1	ras-related protein RABD1-like
1635	gene-LOC11139484	8.0161583448	15.780972385266	-0.758960026	0.953433881	-0.97720319168	protein_codin	XM_023020896.1	uncharacterized LOC111394845%2C transcript variant X4
1636	gene-LOC11139484	954.9049501027	834.41346634223	1.2909497457	0.981175885	0.194594695200	protein_codin	XM_023020875.1	pheophytinase%2C chloroplastic-like%2C transcript variant X5
1637	gene-LOC11139485	690.1680708602	602.25970264426	0.8194739504	0.960211879	0.196562001093	protein_codin	XM_023020880.1	N-terminal acetyltransferase A complex catalytic subunit NAA10-like%2C transcript variant X3
1638	gene-LOC11139485	4.51153422576667	0.5	1.1920371770	0.982419761	3.173618130488	protein_codin	XM_023020884.1	chaperonin-like RbcX protein 2%2C chloroplastic%2C transcript variant X1
1639	gene-LOC11139486	0.6358831613	4.4325944412666	-1.042655721	0.983530761	-2.80131775892	protein_codin	XM_023020895.1	protein OBERON 4-like
1640	gene-LOC11139486	0.5	1.7863080976333	-0.789003132	0.956345087	-1.83698093409	protein_codin	XM_023020901.1	ATP synthase subunit O%2C mitochondrial-like%2C transcript variant X2
1641	gene-LOC11139488	2.5612481251	0.6531665342333	0.7162855073	0.953263197	1.971324243442	protein_codin	XM_023020933.1	uncharacterized LOC111394880%2C transcript variant X1
1642	gene-LOC11139489	165.659893832467	139.9900900685	0.9018170101	0.965168233	0.242899667108	protein_codin	XM_023020946.1	BTB/POZ domain-containing protein At1g21780-like
1643	gene-LOC11139500	0.5	3.4738397537666	-0.818365745	0.959493762	-2.79653120488	protein_codin	XM_023021034.1	peroxidase 52-like
1644	gene-LOC11139511	28.6283988551333	49.289734537333	-1.064587864	0.978761553	-0.78384022627	protein_codin	XM_023021120.1	G-type lectin S-receptor-like serine/threonine-protein kinase At4g27290
1645	gene-LOC11139511	3.62324761586667	10.878540547633	-0.799305642	0.957707095	-1.58612970998	protein_codin	XM_023021121.1	G-type lectin S-receptor-like serine/threonine-protein kinase At4g27290
1646	gene-LOC11139512	2.69426815593333	0.6531665342333	0.7439137701	0.953546246	2.044370667229	protein_codin	XM_023021132.1	EH domain-containing protein 1-like
1647	gene-LOC11139513	7.03456100313333	2.1061412985333	1.0135365034	0.970316328	1.739858165525	protein_codin	XM_023021150.1	cellulose synthase-like protein E6
1648	gene-LOC11139515	0.5	1.8662816210333	-0.769809929	0.954349700	-1.90016670463	protein_codin	XM_023021164.1	uncharacterized LOC111395157
1649	gene-LOC11139524	13.2815839705333	24.977028388233	-0.793970262	0.956992401	-0.91117463054	protein_codin	XM_023021235.1	uncharacterized protein At1g66480-like
1650	gene-LOC11139525	26.8379335016333	8.3182633248333	2.1658922491	0.999999999	1.689919328404	protein_codin	XM_023021237.1	auxin-responsive protein SAUR50-like
1651	gene-LOC11139525	364.960994434467	420.15665318083	-1.195479434	0.985555226	-0.20318504661	protein_codin	XM_023021239.1	uncharacterized protein At2g33490-like
1652	gene-LOC11139527	2.5483395424	0.5	0.7722236578	0.956042524	2.349557516016	protein_codin	XM_023021256.1	protein PECTIC ARABINOGALACTAN SYNTHESIS-RELATED-like
1653	gene-LOC11139527	2.4185190443	0.5	0.7545467141	0.954141492	2.274123898134	protein_codin	XM_023021262.1	sec-independent protein translocase protein TATC%2C chloroplastic-like
1654	gene-LOC11139532	8.08685144396667	1.2061397927333	0.9324893312	0.972099816	2.745180982651	protein_codin	XM_023021305.1	uncharacterized LOC111395325
1655	gene-LOC11139532	16.3745758166333	22.580727251533	-0.743879196	0.951747264	-0.46363441723	protein_codin	XM_023021311.1	uncharacterized LOC111395329
1656	gene-LOC11139538	3.04584470473333	0.5	0.8021320923	0.959452673	2.606842386532	protein_codin	XM_023021356.1	G2/mitotic-specific cyclin S13-7-like%2C transcript variant X2
1657	gene-LOC11139538	5.78465239223333	0.9729997351	1.0173502242	0.969867403	2.571718951051	protein_codin	XM_023021363.1	diacylglycerol kinase 5%2C transcript variant X2
1658	gene-LOC11139539	1.95614846913333	0.5	0.6824614660	0.950524364	1.968015873129	protein_codin	XM_023021370.1	putative late blight resistance protein homolog R1A-10
1659	gene-LOC11139540	34.9525692129667	14.287302442166	1.1757314406	0.982808914	1.290664956107	protein_codin	XM_023021374.1	G-type lectin S-receptor-like serine/threonine-protein kinase At5g24080
1660	gene-LOC11139541	1.22409144556667	5.4440667131	-0.780969526	0.955403992	-2.15297340776	protein_codin	XM_023021387.1	transcription factor SRM1-like
1661	gene-LOC11139541	6.93897906376667	2.5595323076666	0.7366432359	0.953369554	1.438843196968	protein_codin	XM_023021388.1	calumenin
1662	gene-LOC11139542	1.88279253426667	0.5	0.7311818734	0.953322721	1.912874037512	protein_codin	XR_002702799.1	ENHANCER OF AG-4 protein 2-like%2C transcript variant X2
1663	gene-LOC11139542	3.7119836358	1.3133083625333	0.6871358908	0.951242841	1.498984652082	protein_codin	XM_023021414.1	protein FAM133-like%2C transcript variant X2
1664	gene-LOC11139546	5.64739405173333	0.5	1.4664281172	0.965753703	3.497585300107	protein_codin	XM_023021469.1	probable protein phosphatase 2C 58

AQRNA_Control_vs_AQRNA_G7

1665	gene-LOC11139547	9729.2471787974	11256.158241829	-1.5648794252	0.9999999997	-0.2103144327500	protein_codin	XM_023021489.1	uncharacterized LOC111395479
1666	gene-LOC11139549	3.65838220303333	12.102799236	-1.1413001957	0.9785456842	-1.7260630534998	protein_codin	XM_023021513.1	uncharacterized LOC111395495
1667	gene-LOC11139549	3.06619112976667	0.5	0.9928071491	0.973238823	2.616447629603	protein_codin	XM_023021514.1	mannan endo-1%2C4-beta-mannosidase 2-like
1668	gene-LOC11139551	0.629428869966667	2.5331430691666	-0.7369637605	0.9506756748	-2.00881330394	protein_codin	XM_023021536.1	50S ribosomal protein L1
1669	gene-LOC11139551	14.3716718342667	7.9448458390333	0.7925153805	0.9585953978	0.855136766978	protein_codin	XM_023021538.1	ATP-dependent zinc metalloprotease FTSH 2%2C chloroplastic
1670	gene-LOC11139553	0.938432989266667	5.5611781342	-0.7336412968	0.9500868303	-2.567064914665	protein_codin	XM_023021558.1	flowering time control protein FPA
1671	gene-LOC11139554	11.1081666399333	4.4325944412666	0.7842109253	0.9576195153	1.325397450363	protein_codin	XM_023021567.1	50S ribosomal protein L13%2C chloroplastic-like%2C transcript variant X2
1672	gene-LOC11139554	7.32967298876667	1.4535857906666	0.9312183763	0.9717969529	2.334132612492	protein_codin	XM_023021577.1	trifunctional UDP-glucose 4%2C6-dehydratase/UDP-4-keto-6-deoxy-D-glucose 3%2C5-epimerase/UDP-4-keto-L-rhamnose-reductase RHM1-like
1673	gene-LOC11139555	4.61317882836667	0.5	1.6666684369	0.970958399	3.205761219088	protein_codin	XR_002702809.1	protein FLX-like 1%2C transcript variant X3
1674	gene-LOC11139559	22.7064881224333	47.616540128066	-1.1412304900	0.9785336882	-1.068358206324	protein_codin	XM_023021641.1	chlorophyll a-b binding protein 8%2C chloroplastic
1675	gene-LOC11139561	3.11979256253333	8.1847664092333	-0.8456600440	0.9560481543	-1.391491136859	protein_codin	XM_023021692.1	uncharacterized LOC111395613
1676	gene-LOC11139562	5.3919913652	0.6531665342333	1.1823697886	0.9825884459	3.045295406468	protein_codin	XM_023021709.1	proton pump-interactor 1-like
1677	gene-LOC11139563	2.8107884432	0.6531665342333	0.8657410647	0.9615507626	2.105452092216	protein_codin	XM_023021716.1	MAP kinase kinase MKK1/SSP32-like%2C transcript variant X1
1678	gene-LOC11139563	1.52664127353333	5.0110902377333	-0.7615152178	0.9536566028	-1.714763416510	protein_codin	XM_023021723.1	probable LRR receptor-like serine/threonine-protein kinase At1g14390
1679	gene-LOC11139563	5.87150883263333	1.605946517	0.8260685606	0.9603327878	1.870307440506	protein_codin	XR_002702827.1	uncharacterized LOC111395636%2C transcript variant X1
1680	gene-LOC11139564	2.3381168952	0.5	0.7562683526	0.9542771787	2.225347059856	protein_codin	XM_023021742.1	single-stranded DNA-binding protein WHY1%2C chloroplastic-like%2C transcript variant X2
1681	gene-LOC11139564	0.5	2.1861148219	-1.0350065102	0.9845091010	-2.128369178061	protein_codin	XM_023021732.1	trafficking protein particle complex subunit 3-like
1682	gene-LOC11139565	0.5	3.5726161952333	-1.3171151594	0.9711588873	-2.836980934081	protein_codin	XR_002702832.1	21.7 kDa class VI heat shock protein%2C transcript variant X5
1683	gene-LOC11139567	2.97001726753333	0.7331400576333	0.7015369207	0.9527305693	2.018310579386	protein_codin	XM_023021777.1	calcium/calmodulin-regulated receptor-like kinase 1%2C transcript variant X2
1684	gene-LOC11139567	31.6367502425	47.054125362566	-1.0238102806	0.9846517869	-0.572719801083	protein_codin	XM_023021783.1	(-)-germacrene D synthase-like
1685	gene-LOC11139568	2.03556706723333	9.6528529217333	-0.9868559836	0.9708072138	-2.245524641338	protein_codin	XR_002702849.1	protein TWIN LOV 1%2C transcript variant X7
1686	gene-LOC11139568	11.0694408919333	0.6531665342333	2.3887743444	1	4.082987669840	protein_codin	XM_023021792.1	protochlorophyllide reductase-like
1687	gene-LOC11139569	59.7339972994667	84.112019036566	-0.8583351303	0.9519256236	-0.493759698783	protein_codin	XM_023021818.1	cyclic nucleotide-gated ion channel 4-like%2C transcript variant X2
1688	gene-LOC11139570	5.17856918526667	1.1797505542	0.9768818427	0.9754036615	2.134071693800	protein_codin	XM_023021830.1	G-type lectin S-receptor-like serine/threonine-protein kinase At4g27290%2C transcript variant X1
1689	gene-LOC11139572	4.62608741103333	1.605946517	0.6805143869	0.9501815255	1.526368678865	protein_codin	XM_023021853.1	G-type lectin S-receptor-like serine/threonine-protein kinase At4g27290%2C transcript variant X2
1690	gene-LOC11139572	4.6667802611	0.9729997351	0.9614556993	0.9761930338	2.261916221163	protein_codin	XM_023021870.1	protein NRT1/ PTR FAMILY 4.6-like%2C transcript variant X2
1691	gene-LOC11139573	1.96905705183333	0.5	0.6815674662	0.9503663139	1.977504912765	protein_codin	XM_023021873.1	CCR4-NOT transcription complex subunit 9-like
1692	gene-LOC11139577	4.8770029083	13.038327023733	-0.8190776513	0.9595185374	-1.418692028279	protein_codin	XM_023021914.1	probable WRKY transcription factor 20
1693	gene-LOC11139577	2.51469290656667	7.1436207747666	-0.7569917992	0.9532557072	-1.506273266423	protein_codin	XR_002702866.1	zinc finger CCCH domain-containing protein 18-like%2C transcript variant X3
1694	gene-LOC11139578	16.7274723187333	4.4325944412666	1.0746311093	0.9676123309	1.915996182050	protein_codin	XM_023021936.1	probable glutathione S-transferase
1695	gene-LOC11139579	189.2766227604	156.99786035113	1.1110411045	0.9750257070	0.269751339739	protein_codin	XM_023021941.1	BTB/POZ domain-containing protein At1g55760-like%2C transcript variant X2
1696	gene-LOC11139581	40.9939119483667	29.1662667636	1.1008892376	0.9722616392	0.491108934036	protein_codin	XM_023021973.1	uncharacterized LOC111395811
1697	gene-LOC11139585	0.992034422	8.6372907178666	-1.5430233387	0.9999999903	-3.122116763534	protein_codin	XR_002702881.1	inosine-5'-monophosphate dehydrogenase 2-like%2C transcript variant X2
1698	gene-LOC11139588	8.66742159116667	3.5990054337666	0.9068243724	0.9660885189	1.268004599871	protein_codin	XM_023022086.1	U-box domain-containing protein 4-like
1699	gene-LOC11139589	2.66845099056667	0.5	0.8904698014	0.9635352514	2.416002514674	protein_codin	XM_023022103.1	prosaposin-like%2C transcript variant X2
1700	gene-LOC11139592	140.447383076467	156.59718692626	-0.7507734706	0.9526199721	-0.157028553326	protein_codin	XM_023022154.1	BTB/POZ domain-containing protein At5g47800-like
1701	gene-LOC11139596	1.85599181786667	8.0570006275333	-0.7968687620	0.9573807382	-2.118052517530	protein_codin	XM_023022205.1	uncharacterized LOC111395966
1702	gene-LOC11139598	43.0094756337667	69.5550008761	-1.3372309098	0.9737269390	-0.693499703376	protein_codin	XM_023022225.1	protein HEAT-STRESS-ASSOCIATED 32
1703	gene-LOC11139599	689.4982026203	861.40085393386	-0.7484915569	0.9523549064	-0.321137962834	protein_codin	XM_023022238.1	uncharacterized LOC111395991
1704	gene-LOC11139599	6.4284777962	0.5	1.5607012481	0.9610393113	3.684477161214	protein_codin	XM_023022235.1	receptor-like serine/threonine-protein kinase SD1-8%2C transcript variant X1
1705	gene-LOC11139600	60.5835581613333	94.7752948514	-2.1081834423	1	-0.645584727490	lncRNA	XR_002702903.1	uncharacterized LOC111396001
1706	gene-LOC11139600	0.629428869966667	3.0468379831	-0.7371525710	0.9507065584	-2.275197528892	protein_codin	XM_023022255.1	(3S%2C6E)-nerolidol synthase 1-like%2C transcript variant X2
1707	gene-LOC11139600	2.44818289113333	0.5	0.9691321958	0.9760356773	2.291711338371	protein_codin	XM_023022259.1	inositol-tetrakisphosphate 1-kinase 1-like
1708	gene-LOC11139605	2.46109147383333	0.5	0.9626316258	0.9762078754	2.299298280873	protein_codin	XM_023022320.1	receptor-like protein kinase HSL1
1709	gene-LOC11139605	8.1405081026	0.7331400576333	1.1732186522	0.9828962920	3.472958105957	protein_codin	XM_023022326.1	phosphatidylinositol 4-phosphate 5-kinase 9-like%2C transcript variant X2
1710	gene-LOC11139605	5.593792619	1.6126661369	0.6907671638	0.9517295238	1.794378974506	protein_codin	XM_023022329.1	E3 ubiquitin-protein ligase RZFP34-like%2C transcript variant X1
1711	gene-LOC11139607	123.387305454767	83.765613919066	1.7110456269	0.961168963	0.558763933145	lncRNA	XR_002702930.1	uncharacterized LOC111396070
1712	gene-LOC11139607	3.08653755483333	12.181966951533	-1.1124830701	0.974645283	-1.980685845365	protein_codin	XM_023022353.1	AT-rich interactive domain-containing protein 5-like
1713	gene-LOC11139609	87.6368257190333	123.96720593836	-1.0763803884	0.9763070550	-0.500349388397	protein_codin	XM_023022390.1	cytochrome P450 CYP72A219-like

1714	gene-LOC11139613	12.1188359056	4.3722296438	0.8630842232	0.961410584	1.470810041884	protein_codin	XM_023022450.1	cyclin-D1-1-like
1715	gene-LOC11139616	1.85599181786667	0.5	0.9346285653	0.972602995	1.892190350363	protein_codin	XM_023022505.1	protein-tyrosine-phosphatase MKP1-like%2C transcript variant X4
1716	gene-LOC11139617	0.5	2.4599500583333	-0.805478126	0.958483519	-2.29862902640	protein_codin	XM_023022508.1	paramyosin-like
1717	gene-LOC11139617	3.32715207923333	0.5	0.9823641090	0.974745431	2.734287811198	protein_codin	XM_023022519.1	polygalacturonase-1 non-catalytic subunit beta-like
1718	gene-LOC11139619	69.9326025793667	111.2964730672	-1.139145980	0.978178639	-0.67037077310	protein_codin	XM_023022540.1	intracellular ribonuclease LX-like%2C transcript variant X2
1719	gene-LOC11139620	2.08916849996667	0.5	0.7050067510	0.952935613	2.062928856341	protein_codin	XM_023022550.1	uncharacterized LOC111396200
1720	gene-LOC11139621	18.335803398	10.2653242771	0.6994072432	0.952583760	0.836884278009	protein_codin	XM_023022565.1	triose phosphate/phosphate translocator%2C chloroplastic-like
1721	gene-LOC11139621	0.5	3.899169016	-1.085286266	0.974985443	-2.96316669211	protein_codin	XM_023022569.1	ninja-family protein AFP3-like
1722	gene-LOC11139624	3.3622866664	9.1170100728333	-0.848256154	0.955297958	-1.43911803645	protein_codin	XM_023022615.1	uncharacterized LOC111396246%2C transcript variant X2
1723	gene-LOC11139625	40.8060028282	22.4767210205	0.9602536066	0.976177991	0.860349811910	protein_codin	XM_023022624.1	DNL-type zinc finger protein
1724	gene-LOC11139625	3.0532825471	0.6531665342333	0.9489887396	0.975282908	2.224838321284	protein_codin	XM_023022632.1	phosphoacetylglucosamine mutase-like%2C transcript variant X2
1725	gene-LOC11139626	0.5	2.8333675441	-0.853196542	0.953722240	-2.50251776008	protein_codin	XM_023022655.1	transmembrane protein 60-like
1726	gene-LOC11139632	1.55989628123333	6.8509826202	-1.162609205	0.982234027	-2.13486081892	protein_codin	XR_002702982.1	uncharacterized PKHD-type hydroxylase At1g22950-like%2C transcript variant X2
1727	gene-LOC11139635	0.5	5.2841196663	-1.248647688	0.980780454	-3.40166313880	protein_codin	XM_023022766.1	ankyrin repeat-containing protein At2g01680-like
1728	gene-LOC11139635	2.08916849996667	6.2920955497333	-0.775773717	0.954880837	-1.59061172288	protein_codin	XM_023022775.1	uncharacterized LOC111396358
1729	gene-LOC11139637	2.71102342006667	0.5	0.8481695866	0.960790892	2.438837576424	protein_codin	XM_023022804.1	uncharacterized LOC111396370%2C transcript variant X2
1730	gene-LOC11139637	2.69426815593333	0.5	0.8963939323	0.964311812	2.429893446933	lncRNA	XR_002702992.1	uncharacterized LOC111396377%2C transcript variant X1
1731	gene-LOC11139638	38.0006851253333	24.419874718866	1.2498300160	0.985731514	0.637969630661	protein_codin	XM_023022826.1	50S ribosomal protein L19-1%2C chloroplastic-like
1732	gene-LOC11139648	3.33646950103333	9.8655175528333	-0.843742537	0.956564718	-1.56407242174	lncRNA	XR_002703012.1	uncharacterized LOC111396480
1733	gene-LOC11139658	3.15343919836667	0.5	0.9662992048	0.976157527	2.656926116795	protein_codin	XM_023022992.1	dynein light chain 2%2C cytoplasmic
1734	gene-LOC11139659	5.3190270584	0.5	1.5036011957	0.969207407	3.411162376104	protein_codin	XM_023023047.1	RING-H2 finger protein ATL63-like
1735	gene-LOC11139661	0.5	2.8257812237	-1.119397939	0.975307321	-2.49864977434	protein_codin	XM_023023035.1	nuclear transcription factor Y subunit C-3-like
1736	gene-LOC11139662	0.5	4.4845062177333	-1.185478518	0.985046954	-3.16494914089	protein_codin	XM_023023036.1	peroxisomal adenine nucleotide carrier 1-like
1737	gene-LOC11139663	0.5	3.7853417190666	-1.078716400	0.975909185	-2.92042354437	protein_codin	XM_023023072.1	probable aldo-keto reductase 3%2C transcript variant X2
1738	gene-LOC11139665	16.2983567512667	5.8115094941666	1.1178780990	0.976930666	1.487741668812	protein_codin	XM_023023098.1	UDP-glucuronate 4-epimerase 6-like
1739	gene-LOC11139666	9.91185946176667	4.3261707866666	0.7770721199	0.956681891	1.196065111440	protein_codin	XM_023023111.1	UDP-glycosyltransferase 74F2-like
1740	gene-LOC11139668	2.26515258846667	0.5	0.7906429836	0.958390639	2.179608238443	protein_codin	XM_023023138.1	putative disease resistance protein RGA4%2C transcript variant X2
1741	gene-LOC11139668	2.08916849996667	0.5	0.7050067510	0.952935613	2.062928856341	lncRNA	XR_002703044.1	uncharacterized LOC111396683
1742	gene-LOC11139670	0.6358831613	3.3123419839666	-0.782562973	0.955576967	-2.38101802138	protein_codin	XM_023023168.1	probable sugar phosphate/phosphate translocator At3g17430%2C transcript variant X3
1743	gene-LOC11139672	244.791445141033	196.0897363081	1.3099706861	0.977133829	0.320039116032	protein_codin	XM_023023205.1	UDP-glycosyltransferase 74E1-like%2C transcript variant X1
1744	gene-LOC11139673	0.5	3.0452263673666	-0.959484033	0.951604986	-2.60654947507	protein_codin	XM_023023217.1	ABC transporter G family member 25
1745	gene-LOC11139675	2.76821601366667	0.5	0.7280483209	0.953313645	2.468956525953	lncRNA	XR_002703062.1	uncharacterized LOC111396755%2C transcript variant X4
1746	gene-LOC11139676	0.5	3.3206732195333	-0.770359075	0.954396269	-2.73147575724	protein_codin	XM_023023264.1	protein slowmo homolog%2C transcript variant X2
1747	gene-LOC11139677	5.32907251063333	1.0801683049	0.7632945174	0.954962903	2.302628342031	protein_codin	XM_023023276.1	casein kinase 1-like protein 11
1748	gene-LOC11139679	2.72393200276667	0.5	0.9684666577	0.976072516	2.445690689952	lncRNA	XR_002703066.1	uncharacterized LOC111396795
1749	gene-LOC11139682	1.10111686693333	9.2793745432	-1.258721830	0.978369846	-3.07505996946	protein_codin	XM_023023353.1	receptor protein-tyrosine kinase CEPR1-like
1750	gene-LOC11139683	43.3165126510333	65.241433935233	-0.773548103	0.954675115	-0.59087139289	protein_codin	XM_023023376.1	pentatricopeptide repeat-containing protein At4g20090
1751	gene-LOC11139684	13.0949625069333	5.4983350204333	0.7481490934	0.953729113	1.251945209161	protein_codin	XM_023023395.1	WEB family protein At5g55860-like%2C transcript variant X3
1752	gene-LOC11139685	4.06168060513333	0.5	1.2741666002	0.983987716	3.022076796131	protein_codin	XM_023023408.1	DNA-directed RNA polymerases I and III subunit RPAC1-like%2C transcript variant X6
1753	gene-LOC11139687	102.9956208612	77.607821092766	1.4460888078	0.960416274	0.408309043106	protein_codin	XM_023023458.1	ribulose bisphosphate carboxylase/oxygenase activase%2C chloroplastic
1754	gene-LOC11139687	62.4979427068333	38.9741100995	1.0781144827	0.967951154	0.681292618175	protein_codin	XM_023023461.1	phosphomannomutase/phosphoglucomutase%2C transcript variant X1
1755	gene-LOC11139687	0.5	2.5126676426666	-0.801150444	0.957946613	-2.32921985466	protein_codin	XM_023023465.1	GATA transcription factor 8-like
1756	gene-LOC11139688	6.48567038976667	1.8662816210333	0.7852288251	0.957746342	1.797089001380	protein_codin	XM_023023480.1	transcription factor ICE1%2C transcript variant X1
1757	gene-LOC11139693	8.11493981693333	3.1728094709666	0.7121957483	0.953189841	1.354819506121	protein_codin	XM_023023549.1	LRR receptor-like serine/threonine-protein kinase GSO2
1758	gene-LOC11139694	2.5612481251	0.5	0.8691339282	0.961743624	2.356847023146	protein_codin	XM_023023564.1	transmembrane protein 230-like%2C transcript variant X2
1759	gene-LOC11139695	3.963931269	0.5	0.8397074612	0.960564421	2.986931947707	protein_codin	XM_023023587.1	probable calcium-binding protein CML13
1760	gene-LOC11139697	2.7020976264	0.5	0.6901994521	0.951655310	2.434079799970	protein_codin	XM_023023615.1	twinkle homolog protein%2C chloroplastic/mitochondrial-like%2C transcript variant X1
1761	gene-LOC11139698	3.26064206383333	0.5	0.9928877293	0.973226604	2.705156078291	protein_codin	XM_023023637.1	uncharacterized LOC111396985
1762	gene-LOC11139699	3.6787286281	1.2333348391333	0.6876309796	0.951314600	1.576642724292	protein_codin	XM_023023643.1	extra-large guanine nucleotide-binding protein 1

AQRNA_Control_vs_AQRNA_G7

1763	gene-LOC11139699	7.27803865806667	2.4599500583333	0.7280218116	0.9533135817	1.564920687818	protein_codin	XR_002703105.1	uncharacterized LOC111396996%2C transcript variant X2
1764	gene-LOC11139701	0.9255244066	3.7589524805333	-0.7699038014	0.9543576448	-2.021987737019	protein_codin	XM_023023669.1	amino acid transporter AVT6A-like%2C transcript variant X1
1765	gene-LOC11139701	2.46109147383333	0.5	0.9626316258	0.9762078754	2.299298280873	protein_codin	XM_023023674.1	probable esterase D14L
1766	gene-LOC11139703	1.36395739586667	6.0837331148666	-0.8301685343	0.9590739571	-2.157158283863	protein_codin	XM_023023712.1	uncharacterized protein C9orf85 homolog%2C transcript variant X1
1767	gene-LOC11139703	15.0313564739333	6.6918413813	0.9711417876	0.9759136048	1.167500053917	protein_codin	XM_023023720.1	E3 ubiquitin-protein ligase At3g02290-like%2C transcript variant X5
1768	gene-LOC11139704	43.2444766694	60.092513279066	-0.9698299575	0.9588518677	-0.474669383052	protein_codin	XM_023023740.1	filament-like plant protein 3%2C transcript variant X2
1769	gene-LOC11139706	2.15854164583333	0.5	0.9576639473	0.9760782773	2.110056927711	protein_codin	XM_023023777.1	40S ribosomal protein S6-like
1770	gene-LOC11139708	0.6358831613	3.2067850299	-0.7801537564	0.9553171830	-2.334294033770	protein_codin	XM_023023818.1	probable inactive receptor kinase At1g27190
1771	gene-LOC11139708	0.5	2.2456129188	-0.7730286608	0.9546290333	-2.167109268675	protein_codin	XM_023023824.1	coronatine-insensitive protein homolog 1b-like%2C transcript variant X1
1772	gene-LOC11139709	3.64547362036667	0.9729997351	0.7362849821	0.9533637931	1.905594944496	protein_codin	XM_023023838.1	pyruvate dehydrogenase E1 component subunit beta-3%2C chloroplastic-like
1773	gene-LOC11139710	80.2842149836667	126.1491699355	-1.6974471548	0.9997486433	-0.651942446966	protein_codin	XM_023023850.1	uncharacterized LOC111397108
1774	gene-LOC11139710	5.6076847527	18.876225756433	-1.5683162726	0.9999999998	-1.751093178345	lncRNA	XR_002703149.1	uncharacterized LOC111397109
1775	gene-LOC11139711	0.5	2.9857282704666	-1.0424896930	0.9835565106	-2.578082872542	protein_codin	XM_023023862.1	3-oxoacyl-[acyl-carrier-protein] synthase I%2C chloroplastic-like
1776	gene-LOC11139714	5.11304272083333	1.0801683049	0.7438085770	0.9535426436	2.242925958781	protein_codin	XM_023023900.1	ubiquitin carboxyl-terminal hydrolase 17-like%2C transcript variant X1
1777	gene-LOC11139714	2.00974990186667	0.5	0.7043739485	0.9528997665	2.007015980117	protein_codin	XM_023023902.1	kinase-interacting protein 1
1778	gene-LOC11139715	4.37851419493333	0.5	0.9691713616	0.9760333161	3.130441388717	protein_codin	XM_023023918.1	receptor-like serine/threonine-protein kinase ALE2
1779	gene-LOC11139717	5.6086683037	1.6126661369	0.7053639695	0.9529528020	1.798210469979	protein_codin	XM_023023946.1	glutathione S-transferase F11-like
1780	gene-LOC11139718	4.3299918744	9.4912942591666	-0.7513164341	0.9526806385	-1.132240512937	protein_codin	XM_023023959.1	glutamate decarboxylase-like
1781	gene-LOC11139718	1.43046741126667	5.4388978162333	-0.9766608763	0.9637894934	-1.926827690565	protein_codin	XM_023023967.1	expansin-A8-like
1782	gene-LOC11139719	30.4059556259333	18.713861286033	0.8046127346	0.9596205994	0.700246667762	protein_codin	XM_023023969.1	epidermal growth factor receptor substrate 15-like 1%2C transcript variant X1
1783	gene-LOC11139723	0.629428869966667	2.6131165925666	-0.7568383522	0.9532419429	-2.053656239302	lncRNA	XR_002703202.1	uncharacterized LOC111397231
1784	gene-LOC11139724	9.97738592613333	2.1928344418	1.0649797934	0.9670948608	2.185864985975	protein_codin	XM_023024078.1	26S proteasome non-ATPase regulatory subunit 4 homolog
1785	gene-LOC11139725	49.6548792481667	18.846552393766	2.1984802723	0.9999999999	1.397634853639	protein_codin	XM_023024083.1	uncharacterized LOC111397253
1786	gene-LOC11139726	3.95806890053333	0.5	1.1181728914	0.9770120955	2.984796726481	protein_codin	XM_023024099.1	ATP synthase subunit delta'%2C mitochondrial-like
1787	gene-LOC11139727	31.3310008818333	53.423655226666	-1.2202177120	0.9849341081	-0.769887830072	protein_codin	XM_023024118.1	protein DCL%2C chloroplastic-like
1788	gene-LOC11139728	5.2654256257	1.7395043253	0.7434791130	0.9535313799	1.597873888611	protein_codin	XM_023024128.1	uncharacterized LOC111397283
1789	gene-LOC11139732	51.3477955602333	73.181824485966	-1.0669361545	0.9782293516	-0.511183042998	protein_codin	XM_023024173.1	protein NDR1-like
1790	gene-LOC11139732	19.0232723050667	3.946033681	1.6656282577	0.9714055212	2.269290258798	protein_codin	XM_023024176.1	2S sulfur-rich seed storage protein 1-like
1791	gene-LOC11139732	2.48143789886667	9.0627417655	-0.8137226790	0.9592698965	-1.868771227290	protein_codin	XM_023024182.1	early nodulin-like protein 3
1792	gene-LOC11139735	12.0574925249333	27.513577366966	-1.4732697493	0.9999392489	-1.190213818068	protein_codin	XM_023024249.1	auxilin-like protein 1
1793	gene-LOC11139740	0.5	3.2051734141666	-0.8581777332	0.9519824316	-2.680402415187	protein_codin	XM_023024311.1	uncharacterized LOC111397406%2C transcript variant X1
1794	gene-LOC11139741	2.3058454385	0.5	0.7515754292	0.9539302290	2.205295811956	protein_codin	XM_023024330.1	uncharacterized WD repeat-containing protein C2A9.03-like%2C transcript variant X1
1795	gene-LOC11139744	44.8054117275667	26.3150238947	0.9093294962	0.9665937569	0.767786290863	protein_codin	XR_002703249.1	probable disease resistance protein At5g63020%2C transcript variant X9
1796	gene-LOC11139745	10.7862539379667	4.3713629432333	0.7748869831	0.9563918403	1.303038832816	protein_codin	XM_023024395.1	mitochondrial phosphate carrier protein 3%2C mitochondrial-like
1797	gene-LOC11139746	49.6674837252667	78.9128591124	-1.3840692052	0.9874065202	-0.667958753132	protein_codin	XM_023024409.1	probable E3 ubiquitin-protein ligase RHC1A
1798	gene-LOC11139749	4.43719473986667	0.7331400576333	0.9835318401	0.9745890371	2.597487132211	protein_codin	XM_023024461.1	probable receptor-like serine/threonine-protein kinase At5g57670%2C transcript variant X2
1799	gene-LOC11139750	2.3649176116	0.5	0.8202289328	0.9602284416	2.241789924196	lncRNA	XR_002703258.1	uncharacterized LOC111397505
1800	gene-LOC11139751	3.87766675143333	0.5	0.9951734185	0.9728806702	2.955188822858	protein_codin	XM_023024476.1	vesicle-associated protein 4-1-like
1801	gene-LOC11139751	937.259202892567	1038.9219247682	-0.7987369535	0.9576307418	-0.148567248004	protein_codin	XM_023024482.1	zinc finger protein CONSTANS-LIKE 4-like%2C transcript variant X2
1802	gene-LOC11139751	2.14563306316667	0.5	0.7875947307	0.9580373793	2.101403373758	protein_codin	XM_023024483.1	universal stress protein PHOS34-like%2C transcript variant X1
1803	gene-LOC11139751	2.9968179839	0.5	0.9546740034	0.9758852584	2.583431462330	protein_codin	XM_023024493.1	G-type lectin S-receptor-like serine/threonine-protein kinase At4g27290%2C transcript variant X2
1804	gene-LOC11139753	3.488260483	0.5	0.8151360748	0.9600990724	2.802507776210	protein_codin	XM_023024511.1	uncharacterized LOC111397531%2C transcript variant X1
1805	gene-LOC11139753	2.60194097513333	0.5	0.7922052050	0.9585612544	2.379588234981	protein_codin	XM_023024520.1	protein HASTY 1
1806	gene-LOC11139756	97.8889932142	10.8242113476	1.8209981677	0.9995925531	3.176884736173	lncRNA	XR_002703271.1	uncharacterized LOC111397564
1807	gene-LOC11139756	26.4098014851667	15.2236360378	1.0478680330	0.9673711201	0.794760481615	protein_codin	XM_023024557.1	calcium-transporting ATPase 12%2C plasma membrane-type-like%2C transcript variant X2
1808	gene-LOC11139756	992.542925598333	160.12000430516	1.9752379629	1	2.631975937800	lncRNA	XR_002703272.1	uncharacterized LOC111397567
1809	gene-LOC11139757	33.7555825893333	21.008011378333	0.9080440840	0.9663327019	0.684186515959	protein_codin	XM_023024570.1	uncharacterized LOC111397577
1810	gene-LOC11139775	746.6208038211	450.23805896473	3.3951122133	0.9999999999	0.729687694361	protein_codin	XM_023024742.1	selenium-binding protein 1-like
1811	gene-LOC11139776	0.5	4.0515297423333	-1.0271430109	0.9847921159	-3.018466731450	protein_codin	XM_023024752.1	UDP-glucuronic acid decarboxylase 6-like%2C transcript variant X1

1812	gene-LOC1113977	0.5	3.6865043849	-1.033761570	0.984607952	-2.88225347297	protein_codin	XM_023024771.1	mitotic spindle checkpoint protein BUBR1
1813	gene-LOC11139781	3.8528331371	0.7331400576333	0.8467673572	0.960746056	2.393758966043	protein_codin	XM_023024795.1	laccase-15-like
1814	gene-LOC11139786	1.7062165229	6.4512367886333	-0.765207844	0.953966419	-1.91877503136	protein_codin	XM_023024856.1	uncharacterized LOC111397864
1815	gene-LOC11139793	343.516450406467	280.17727664516	0.8634084771	0.961426898	0.294039235947	protein_codin	XM_023024895.1	cyclin-dependent kinase C-1-like
1816	gene-LOC11139798	5.1056048785	0.5	1.2767409218	0.983619238	3.352081892252	protein_codin	XM_023024955.1	mannose-1-phosphate guanylyltransferase 1
1817	gene-LOC11139804	8.75427803156667	3.7853417190666	0.6872244976	0.951255663	1.209564659617	protein_codin	XM_023025002.1	protein argonaute 4-like
1818	gene-LOC1113980	0.9255244066	4.6443923718	-0.821113569	0.959530978	-2.32714692004	protein_codin	XM_023025048.1	1%2C4-alpha-glucan-branching enzyme 1%2C chloroplastic/amyloplastic-like
1819	gene-LOC1113981	3.3529692446	0.7331400576333	0.7771373156	0.956690552	2.193278510525	protein_codin	XM_023025060.1	putative tRNA pseudouridine synthase Pus10
1820	gene-LOC11139811	208.7268890946	86.451709537733	1.8496341622	0.999978652	1.271650068834	protein_codin	XM_023025067.1	calcium-transporting ATPase 12%2C plasma membrane-type-like
1821	gene-LOC11139811	1.06786185923333	3.6253946722666	-0.749539049	0.952475939	-1.76341303100	protein_codin	XM_023025072.1	AT-hook motif nuclear-localized protein 14-like
1822	gene-LOC11139818	0.5	2.5867273540666	-0.771349486	0.954481504	-2.37112799926	protein_codin	XM_023025119.1	ankyrin repeat-containing protein BDA1-like
1823	gene-LOC1113981	2.46754576516667	0.5	0.8241397523	0.960302186	2.303076842358	protein_codin	XM_023025130.1	uncharacterized LOC111398195
1824	gene-LOC1113982	12.9254327098	3.7852808263666	1.2947779781	0.980426459	1.771740337558	protein_codin	XM_023025140.1	TATA-binding protein-associated factor BTAF1
1825	gene-LOC11139826	18.7927584587333	8.9595413423	0.7077404609	0.953054974	1.068680060801	protein_codin	XM_023025189.1	receptor-like protein kinase FERONIA
1826	gene-LOC11139828	10.4236483859	4.8519489987666	0.7746713713	0.956363261	1.103224033582	protein_codin	XM_023025206.1	nucleolar protein 12
1827	gene-LOC11139839	29.4950513834333	17.67438816	0.8428895744	0.960639331	0.738812648833	protein_codin	XM_023025304.1	calcineurin B-like protein 10
1828	gene-LOC11139842	0.938432989266667	4.6444532645333	-0.784203211	0.955763507	-2.30718314026	protein_codin	XM_023025324.1	myosin-1-like
1829	gene-LOC11139844	0.5	2.1928344418	-0.754119384	0.952977800	-2.13279689317	protein_codin	XM_023025351.1	FAM10 family protein At4g22670%2C transcript variant X2
1830	gene-LOC11139845	29.1379165716667	13.994542502266	1.1289371773	0.979715043	1.058033400745	protein_codin	XM_023025352.1	uncharacterized LOC111398451
1831	gene-LOC11139845	0.9255244066	3.6253946722666	-0.785279114	0.955888304	-1.96979511976	protein_codin	XM_023025360.1	deSI-like protein At4g17486
1832	gene-LOC11139846	11.2292616391333	21.672455402866	-0.811010263	0.959055694	-0.94859954537	protein_codin	XM_023025378.1	MADS-box protein SOC1-like%2C transcript variant X4
1833	gene-LOC11139846	10.0051701935333	3.5454211488666	0.9811066987	0.974907150	1.496716792765	protein_codin	XM_023025382.1	PGR5-like protein 1B%2C chloroplastic%2C transcript variant X2
1834	gene-LOC11139848	2.69426815593333	0.5	0.8963939323	0.964311812	2.429893446933	protein_codin	XM_023025396.1	ACT domain-containing protein ACR8-like
1835	gene-LOC11139849	13.0842699059667	6.1848660872	0.7274834169	0.953312689	1.081019161288	protein_codin	XM_023025411.1	signal recognition particle 43 kDa protein%2C chloroplastic
1836	gene-LOC1113985	2.00974990186667	0.5	0.7043739485	0.952899766	2.007015980117	protein_codin	XM_023025442.1	ankyrin repeat-containing protein BDA1-like
1837	gene-LOC11139852	83.6055046944333	115.09966434473	-0.805063809	0.958437186	-0.46121378681	protein_codin	XM_023025473.1	cactin%2C transcript variant X1
1838	gene-LOC11139853	10.8957280110333	2.8852793205666	0.9640285935	0.976198955	1.916979698671	protein_codin	XM_023025482.1	uncharacterized LOC111398532%2C transcript variant X1
1839	gene-LOC11139854	24.1149850498667	12.3186870986	1.0000501111	0.972148718	0.969081411067	protein_codin	XM_023025498.1	RHOMBOID-like protein 2
1840	gene-LOC11139854	0.5	1.8662816210333	-0.769809929	0.954349700	-1.90016670463	protein_codin	XM_023025509.1	DEAD-box ATP-dependent RNA helicase 47A%2C transcript variant X2
1841	gene-LOC11139857	10.8390145681333	1.605946517	0.8699637039	0.961793729	2.754737847105	protein_codin	XM_023025530.1	BTB/POZ domain-containing protein At2g46260-like
1842	gene-LOC11139858	7.49047728696667	2.8257812237	0.8731877662	0.961998922	1.406407874596	protein_codin	XM_023025558.1	probable methyltransferase PMT21%2C transcript variant X3
1843	gene-LOC11139859	8.820788047	2.6922234154	0.8086089606	0.959841283	1.712109413926	protein_codin	XM_023025573.1	acyl-CoA-binding protein%2C transcript variant X2
1844	gene-LOC11139860	44.1695771512	26.709813093666	0.9215877921	0.969420089	0.725683140574	protein_codin	XM_023025586.1	jmjC domain-containing protein 7%2C transcript variant X2
1845	gene-LOC11139860	4.89636578233333	1.55316804	0.7763269181	0.956583229	1.656497412491	protein_codin	XM_023025599.1	F-box protein SKIP31-like%2C transcript variant X1
1846	gene-LOC11139861	2.0762599173	0.5	0.6996756982	0.952603165	2.053987059267	lncRNA	XR_002703365.1	uncharacterized LOC111398617
1847	gene-LOC11139861	2.5483395424	0.5	0.7722236578	0.956042524	2.349557516016	protein_codin	XM_023025614.1	NADH dehydrogenase [ubiquinone] 1 alpha subcomplex subunit 1
1848	gene-LOC11139862	0.5	2.2456129188	-0.773028660	0.954629033	-2.16710926867	protein_codin	XM_023025622.1	ribosomal RNA small subunit methyltransferase H
1849	gene-LOC11139864	5.12595130353333	1.4995837551	0.6879053704	0.951354489	1.773257670916	protein_codin	XM_023025645.1	helicase-like transcription factor CHR28%2C transcript variant X1
1850	gene-LOC11139864	181.6446488233	142.73521374633	1.0282996476	0.968753188	0.347777563789	protein_codin	XM_023025642.1	U-box domain-containing protein 34-like%2C transcript variant X2
1851	gene-LOC11139864	2.15854164583333	0.5	0.9576639473	0.976078277	2.110056927711	protein_codin	XM_023025655.1	uncharacterized LOC111398649
1852	gene-LOC11139867	51.4941547395333	25.485676190766	1.0715499692	0.967383190	1.014722045214	protein_codin	XM_023025698.1	pentatricopeptide repeat-containing protein At2g34400%2C transcript variant X1
1853	gene-LOC11139867	0.6358831613	2.7253931664666	-0.744393455	0.951819460	-2.09963075803	protein_codin	XM_023025711.1	probable serine/threonine-protein kinase SIS8
1854	gene-LOC11139869	38.0423615264333	59.214477030066	-1.040350981	0.983886118	-0.63834312970	protein_codin	XM_023025725.1	MADS-box protein AGL24-like
1855	gene-LOC1113987	2.5483395424	0.5	0.7722236578	0.956042524	2.349557516016	protein_codin	XM_023025745.1	probable cyclic nucleotide-gated ion channel 14
1856	gene-LOC11139872	1.36395739586667	5.2262331851	-0.817219589	0.959452833	-1.93797291503	protein_codin	XM_023025776.1	general transcription factor IIF subunit 1-like%2C transcript variant X1
1857	gene-LOC11139874	0.938432989266667	3.8652543497666	-0.799602850	0.957745403	-2.04223771734	protein_codin	XM_023025804.1	serine/threonine-protein kinase tricorner-like%2C transcript variant X1
1858	gene-LOC11139874	3.0532825471	0.5	1.1838123257	0.982546110	2.610361100988	protein_codin	XR_002703395.1	serine/threonine-protein kinase HT1-like%2C transcript variant X3
1859	gene-LOC11139876	0.5	2.8852793205666	-0.859212762	0.951607986	-2.52871099127	protein_codin	XM_023025856.1	auxin-induced protein PCNT115-like
1860	gene-LOC11139877	2.2586982971	0.5	0.7919045808	0.958528247	2.175491577350	protein_codin	XM_023025859.1	fatty-acid-binding protein 2-like%2C transcript variant X2

1861	gene-LOC11139877	2.5483395424	0.5	0.7722236578	0.956042524	2.349557516016	protein_codin	XM_023025862.1	ankyrin repeat-containing protein BDA1-like
1862	gene-LOC11139878	0.5	2.3000030115333	-0.746106397	0.952051421	-2.20163575017	protein_codin	XM_023025880.1	29 kDa ribonucleoprotein A%2C chloroplastic-like
1863	gene-LOC11139879	0.5	2.9389244981666	-0.767594118	0.954163783	-2.55528829620	protein_codin	XM_023025883.1	uncharacterized LOC111398790
1864	gene-LOC11139883	156.157787064133	191.73335950783	-1.364534762	0.980674110	-0.29609685849	protein_codin	XM_023025955.1	MADS-box transcription factor 7-like
1865	gene-LOC11139883	132.409535424133	184.7080059349	-1.097844997	0.974160577	-0.48023937842	protein_codin	XM_023025960.1	uncharacterized LOC111398839%2C transcript variant X4
1866	gene-LOC11139884	299.3741717309	418.38299443826	-1.927879367	0.999711738	-0.48287445371	lncRNA	XR_002703418.1	uncharacterized LOC111398840%2C transcript variant X3
1867	gene-LOC11139890	0.5	3.4918368639666	-1.082250652	0.975373068	-2.80398615889	protein_codin	XM_023026064.1	uncharacterized LOC111398902%2C transcript variant X5
1868	gene-LOC11139891	3.11979256253333	0.5	1.1572135605	0.983113460	2.641450106346	protein_codin	XM_023026076.1	protein argonaute 7
1869	gene-LOC11139891	9.77883943093333	4.4045326943333	0.7087711637	0.953091017	1.150674291552	protein_codin	XM_023026077.1	probable protein S-acyltransferase 22%2C transcript variant X1
1870	gene-LOC11139892	0.5	1.8126973361	-0.775838238	0.954886876	-1.85813806012	protein_codin	XM_023026086.1	uncharacterized LOC111398924
1871	gene-LOC11139893	3.0310565426	0.7067508191	0.7169716094	0.953270572	2.100547209807	lncRNA	XR_002703448.1	uncharacterized LOC111398933
1872	gene-LOC11139893	39.6913854557333	17.6471931136	1.6978047087	0.961279538	1.169387188607	protein_codin	XM_023026104.1	UDP-glycosyltransferase 89A2-like
1873	gene-LOC11139898	103.715416450867	76.777728473666	1.0355769153	0.968147522	0.433870572222	protein_codin	XM_023026186.1	uncharacterized LOC111398988
1874	gene-LOC11139900	3.70912050526667	0.6531665342333	0.8380098799	0.960528380	2.505554360481	lncRNA	XR_002703498.1	uncharacterized LOC111399008
1875	gene-LOC11139902	2.1520873545	0.5	0.9667103261	0.976141508	2.105736638929	protein_codin	XM_023026224.1	SUPPRESSOR OF GAMMA RESPONSE 1-like
1876	gene-LOC11139907	2.57415670776667	0.5	0.7724889366	0.956076955	2.364099883642	protein_codin	XM_023026296.1	probable NAD(P)H dehydrogenase subunit CRR3%2C chloroplastic
1877	gene-LOC11139908	211.751988825367	258.09139641996	-1.081601743	0.975468065	-0.28550652965	protein_codin	XM_023026312.1	uncharacterized LOC111399081%2C transcript variant X3
1878	gene-LOC11139908	3.488260483	0.5	0.8151360748	0.960099072	2.802507776210	protein_codin	XR_002703525.1	K(+) efflux antiporter 6-like%2C transcript variant X10
1879	gene-LOC11139909	150.084172872067	205.0176882085	-1.401927208	0.993052793	-0.44997654021	protein_codin	XM_023026321.1	molybdenum cofactor sulfurase-like
1880	gene-LOC11139910	4.61317882836667	1.605946517	0.7524240783	0.953986706	1.522337371652	protein_codin	XM_023026346.1	protein VAC14 homolog
1881	gene-LOC11139912	12.2334766134333	2.2396991068	1.5470114233	0.963604981	2.449457628463	lncRNA	XR_002703550.1	uncharacterized LOC111399121%2C transcript variant X17
1882	gene-LOC11139912	6.44980777223333	1.0801683049	1.1235145951	0.978420203	2.578000042213	protein_codin	XM_023026366.1	tobamovirus multiplication protein 3-like
1883	gene-LOC11139913	0.5	3.5793358151	-1.050334964	0.982048449	-2.83969190448	protein_codin	XM_023026374.1	protein BASIC PENTACYSTEINE2-like%2C transcript variant X2
1884	gene-LOC11139916	2.18534236223333	0.5	0.9277477865	0.970947782	2.127859314327	protein_codin	XM_023026425.1	E3 ubiquitin ligase BIG BROTHER-related
1885	gene-LOC11139917	43.2970945511333	9.7311539366666	2.3844906038	0.999999999	2.153587418760	protein_codin	XM_023026443.1	protease 2%2C transcript variant X2
1886	gene-LOC11139920	2.94321655113333	0.5	0.8336306563	0.960449633	2.557393694419	protein_codin	XM_023026477.1	ankyrin repeat-containing protein BDA1-like
1887	gene-LOC11139921	2.8107884432	13.674770194066	-1.386262458	0.988163692	-2.28246981154	lncRNA	XR_002703600.1	uncharacterized LOC111399210%2C transcript variant X2
1888	gene-LOC11139925	2.08916849996667	0.5	0.7050067510	0.952935613	2.062928856341	protein_codin	XM_023026515.1	probable calcium-binding protein CML44
1889	gene-LOC11139926	2.69426815593333	0.5	0.8963939323	0.964311812	2.429893446933	protein_codin	XM_023026556.1	uncharacterized LOC111399264
1890	gene-LOC11139931	4.28382828403333	12.9287410304	-1.146559981	0.979462637	-1.59360923946	protein_codin	XM_023026558.1	uncharacterized LOC111399310
1891	gene-LOC11139931	0.5	4.171331744	-0.859717154	0.951424471	-3.06050805363	protein_codin	XM_023026559.1	F-box/LRR-repeat protein 3
1892	gene-LOC11139931	2.64066672316667	0.5	0.7971634153	0.959049456	2.400902231389	protein_codin	XM_023026560.1	probable WRKY transcription factor 33
1893	gene-LOC11139931	0.992034422	3.5726161952333	-0.737394785	0.950746249	-1.84851884828	protein_codin	XM_023026565.1	mitoferrin-like
1894	gene-LOC11139932	0.5	2.4863392968666	-0.852188209	0.954055966	-2.31402318637	protein_codin	XM_023026578.1	putative serine/threonine-protein kinase
1895	gene-LOC11139932	1.96905705183333	0.5	0.6815674662	0.950366313	1.977504912765	protein_codin	XM_023026585.1	auxin transporter-like protein 2
1896	gene-LOC11139934	2.73684058543333	0.5	0.8471751190	0.960758978	2.452511403536	protein_codin	XM_023026598.1	probable 2-oxoglutarate-dependent dioxygenase At5g05600
1897	gene-LOC11139934	0.5	4.8519489987666	-1.385744279	0.987989125	-3.27856438565	lncRNA	XR_002703642.1	uncharacterized LOC111399345%2C transcript variant X1
1898	gene-LOC11139939	63.5437788563333	133.41810776176	-1.385569473	0.987929007	-1.07013169245	protein_codin	XM_023026669.1	signal peptidase complex catalytic subunit SEC11A-like
1899	gene-LOC11139939	2.44818289113333	0.5	0.9691321958	0.976035677	2.291711338371	protein_codin	XM_023026674.1	probable chlorophyll(ide) b reductase NYC1%2C chloroplastic%2C transcript variant X2
1900	gene-LOC11139940	4.98869296306667	1.0801683049	0.8581609627	0.961174127	2.207405757754	protein_codin	XM_023026706.1	UPF0481 protein At3g47200-like
1901	gene-LOC11139941	36.3504287653667	58.781773711666	-1.033839461	0.984603094	-0.69339651096	protein_codin	XM_023026711.1	putative ABC transporter B family member 8%2C transcript variant X2
1902	gene-LOC11139949	5.5328408665	1.4928641352333	0.7601012216	0.954630622	1.889937557579	protein_codin	XM_023026848.1	rRNA biogenesis protein RRP5
1903	gene-LOC11139950	16.2040624685	6.6926471891666	0.8735186434	0.962021248	1.275706683860	protein_codin	XM_023026862.1	tetraspanin-2
1904	gene-LOC11139951	2.66845099056667	0.6531665342333	0.7381393366	0.953394432	2.030479734970	protein_codin	XM_023026870.1	uncharacterized LOC111399511%2C transcript variant X4
1905	gene-LOC11139953	10.2389387985	2.4599500583333	0.8484232776	0.960799067	2.057365265314	protein_codin	XM_023026897.1	DNA-directed RNA polymerase I subunit 1-like
1906	gene-LOC11139954	5.43307584336667	1.0801683049	0.8501566737	0.960856774	2.330513065147	protein_codin	XM_023026931.1	26S proteasome non-ATPase regulatory subunit 11 homolog%2C transcript variant X1
1907	gene-LOC11139955	2.7020976264	0.5	0.6901994521	0.951655310	2.434079799970	protein_codin	XM_023026939.1	nucleolar protein 10-like%2C transcript variant X2
1908	gene-LOC11139955	0.5	1.8662816210333	-0.769809929	0.954349700	-1.90016670463	protein_codin	XM_023026952.1	transcription factor TGA2.2-like%2C transcript variant X2
1909	gene-LOC11139955	0.938432989266667	3.5726161952333	-0.775580881	0.954862810	-1.92865529883	protein_codin	XM_023026956.1	microtubule-associated protein 70-1-like

1910	gene-LOC11139956	4.03487988876667	9.2242395353	-0.836361433	0.958195315	-1.19290424975	protein_codin	XM_023026961.1	kinesin-like protein KIN-7O%2C transcript variant X2
1911	gene-LOC11139957	2.8107884432	7.0652588670666	-0.754897289	0.953053044	-1.32976754913	lncRNA	XR_002703757.1	uncharacterized LOC111399573%2C transcript variant X5
1912	gene-LOC11139961	1.96905705183333	0.5	0.6815674662	0.950366313	1.977504912765	protein_codin	XR_002703769.1	uncharacterized LOC111399618%2C transcript variant X7
1913	gene-LOC11139965	26.1373071322333	8.5861847492666	1.1389463482	0.981613358	1.606021390418	protein_codin	XM_023027090.1	uncharacterized LOC111399654
1914	gene-LOC11139965	19.2531619318333	31.511583717166	-0.827502446	0.959314583	-0.71078686555	protein_codin	XM_023027092.1	glucan endo-1%2C3-beta-glucosidase%2C basic isoform-like
1915	gene-LOC11139966	2.57415670776667	0.5	0.7724889366	0.956076955	2.364099883642	protein_codin	XM_023027100.1	folylpolyglutamate synthase%2C transcript variant X2
1916	gene-LOC11139969	76.6223291423333	147.79938692463	-2.591989978	0.999999999	-0.94780349901	protein_codin	XM_023027156.1	myb family transcription factor PHL5-like%2C transcript variant X2
1917	gene-LOC11139973	4.6871266861	1.0801683049	0.8200319530	0.960224111	2.117447667861	protein_codin	XM_023027202.1	uncharacterized LOC111399731
1918	gene-LOC11139973	54.0500934814	31.749649100766	1.1519913785	0.982928771	0.767556465938	protein_codin	XM_023027206.1	thaumatin-like protein
1919	gene-LOC11139974	0.938432989266667	5.1734547081	-0.839097691	0.957662896	-2.46280236400	protein_codin	XM_023027216.1	activator of 90 kDa heat shock protein ATPase homolog
1920	gene-LOC11139975	4.587361663	0.9729997351	0.8362193191	0.960494835	2.237153335191	protein_codin	XM_023027223.1	uncharacterized LOC111399756%2C transcript variant X1
1921	gene-LOC11139978	109.229055800433	51.213629482566	3.6797779105	0.999999999	1.092756964852	protein_codin	XM_023027270.1	serine carboxypeptidase-like 18%2C transcript variant X4
1922	gene-LOC11139981	2.49434648156667	7.1452323904333	-0.852685152	0.953892440	-1.51831905490	protein_codin	XM_023027309.1	F-actin-monooxygenase MICAL3-like
1923	gene-LOC11139991	55.4924536276667	73.020448501566	-0.735593691	0.950434103	-0.39600893611	protein_codin	XM_023027400.1	serine/threonine-protein kinase CDG1-like
1924	gene-LOC11139992	45.5309269372333	31.9361071715	0.6855402047	0.951013408	0.511658363425	protein_codin	XM_023027412.1	protein TSS-like
1925	gene-LOC11139994	2.87084416726667	0.6531665342333	0.7208853927	0.953298800	2.135952241815	protein_codin	XM_023027422.1	glycine-rich cell wall structural protein-like
1926	gene-LOC11140010	3.39554167413333	11.222345563466	-0.983000812	0.968243084	-1.72466059869	protein_codin	XM_023027550.1	uncharacterized mitochondrial protein AtMg00810-like
1927	gene-LOC11140017	41.3694260831	32.605386043266	0.7533245796	0.954047231	0.343454642097	protein_codin	XM_023027600.1	protein LONGIFOLIA 1-like
1928	gene-LOC11140019	4.65327975553333	0.5	1.2143581158	0.983339958	3.218247924638	protein_codin	XM_023027626.1	protein DA1-related 1-like
1929	gene-LOC11140026	10.0665135742	3.3599515641333	0.7613136579	0.954751724	1.583051767468	protein_codin	XM_023027688.1	uncharacterized LOC111400269
1930	gene-LOC11140030	113.298229473833	59.555424170666	1.4638691653	0.965288655	0.927820499628	protein_codin	XM_023027723.1	protein FAR1-RELATED SEQUENCE 5-like
1931	gene-LOC11140032	1.85599181786667	0.5	0.9346285653	0.972602995	1.892190350363	protein_codin	XM_023027744.1	uncharacterized LOC111400321%2C transcript variant X2
1932	gene-LOC11140037	1.85599181786667	0.5	0.9346285653	0.972602995	1.892190350363	protein_codin	XM_023027792.1	late embryogenesis abundant protein At3g53040-like
1933	gene-LOC11140042	5.85702477606667	0.5	1.3547025088	0.961885646	3.550167997270	protein_codin	XM_023027852.1	arogenate dehydratase/prephenate dehydratase 6%2C chloroplastic-like
1934	gene-LOC11140045	0.5	2.3000030115333	-0.746106397	0.952051421	-2.20163575017	protein_codin	XM_023027866.1	uncharacterized LOC111400450
1935	gene-LOC11140054	29.5040809879333	16.0734278617	0.6807987290	0.950231273	0.876236886263	protein_codin	XM_023027952.1	caffeoylshikimate esterase-like
1936	gene-LOC11140055	0.5	3.6517839107666	-1.109893827	0.974459603	-2.86860139837	protein_codin	XM_023027965.1	zinc finger MYM-type protein 1-like
1937	gene-LOC11140069	12.0113289345333	6.4775651344333	0.7043073658	0.952896005	0.890872257335	protein_codin	XM_023028081.1	WD repeat-containing protein 75-like
1938	gene-LOC11140072	8.64805871713333	2.5331430691666	1.0935162597	0.970496695	1.771447757274	protein_codin	XM_023028113.1	dr1-associated corepressor-like
1939	gene-LOC11140075	0.5	3.4654476254666	-1.123665538	0.975826824	-2.79304171465	protein_codin	XM_023028139.1	probable E3 ubiquitin-protein ligase RHC1A
1940	gene-LOC11140076	13.1217632233333	30.446405375	-1.440344156	0.991106497	-1.21431031417	protein_codin	XM_023028153.1	F-box/kelch-repeat protein At3g27150%2C transcript variant X3
1941	gene-LOC11140078	5.1388598862	1.55316804	0.7590124207	0.954522925	1.726234390916	protein_codin	XM_023028181.1	uncharacterized protein At2g33490-like
1942	gene-LOC11140078	2.1317409295	6.2657063112	-0.782639039	0.955585557	-1.55544503068	protein_codin	XM_023028187.1	uncharacterized monothiol glutaredoxin ycf64-like%2C transcript variant X2
1943	gene-LOC11140079	2.85088937036667	0.6531665342333	0.7395299112	0.953420635	2.125889276435	lncRNA	XR_002703853.1	uncharacterized LOC111400790%2C transcript variant X1
1944	gene-LOC11140079	26.4278767026	17.541697052266	0.6878149981	0.951341342	0.591272192565	protein_codin	XM_023028193.1	glutaredoxin
1945	gene-LOC11140080	84.8316736938667	27.6726577132	1.8481826163	0.999974507	1.616141817325	lncRNA	XR_002703859.1	uncharacterized LOC111400804%2C transcript variant X2
1946	gene-LOC11140082	7.34356512246667	2.5059480228	0.9501138912	0.975419898	1.551124135264	protein_codin	XM_023028240.1	protein arginine methyltransferase NDUFAF7 homolog%2C mitochondrial-like%2C transcript variant X1
1947	gene-LOC11140084	13.7171538293	3.9715562189666	1.0339558301	0.968272278	1.788204837450	protein_codin	XM_023028273.1	RNA exonuclease 4-like
1948	gene-LOC11140084	0.5	2.1928344418	-0.754119384	0.952977800	-2.13279689317	protein_codin	XM_023028281.1	uncharacterized LOC111400848
1949	gene-LOC11140086	62.1253792251667	38.448119623133	1.0218405957	0.969380789	0.692269710961	protein_codin	XM_023028305.1	protein ALTERED XYLOGLUCAN 4-like
1950	gene-LOC11140087	3.68975763126667	0.9398908767333	0.6849646090	0.950924666	1.972960881475	protein_codin	XM_023028315.1	probable WRKY transcription factor 72
1951	gene-LOC11140087	2.03556706723333	0.5	0.7088504665	0.953093597	2.025430755730	protein_codin	XM_023028324.1	pentatricopeptide repeat-containing protein At5g15300
1952	gene-LOC11140088	5.1572392092	0.7067508191	0.8987386075	0.964663824	2.867325408681	protein_codin	XM_023028327.1	SPX domain-containing protein 4-like
1953	gene-LOC11140093	5.75459691723333	0.5	1.2641287793	0.985120883	3.524714877814	protein_codin	XM_023028415.1	E3 ubiquitin-protein ligase At3g02290-like%2C transcript variant X2
1954	gene-LOC11140096	12.8514848520333	3.4450330916666	1.1861669649	0.982491447	1.899345310993	protein_codin	XM_023028457.1	serine/threonine-protein kinase Nek6-like%2C transcript variant X1
1955	gene-LOC11140096	34.1211940203333	15.8615690384	0.9124402541	0.967262969	1.105132641917	protein_codin	XM_023028470.1	BTB/POZ domain-containing protein At5g66560
1956	gene-LOC11140097	69.4063133103667	94.1926186441	-0.780668254	0.955371859	-0.44054710957	protein_codin	XM_023028473.1	uncharacterized LOC111400972
1957	gene-LOC11140099	9.04391927686667	2.8333675441	0.9210730852	0.969293360	1.674430355027	protein_codin	XM_023028500.1	LOB domain-containing protein 37-like
1958	gene-LOC11140100	0.9255244066	6.5055659886666	-1.247707344	0.980986261	-2.81333163746	protein_codin	XM_023028517.1	multiple RNA-binding domain-containing protein 1-like

1959	gene-LOC11140101	28.7875401568667	76.253410505733	-1.597357747	0.999999999	-1.40535734641	protein_codin	XM_023028540.1	uncharacterized LOC111401012
1960	gene-LOC11140107	0.5	2.5127285353666	-0.801158618	0.957947684	-2.32925481692	protein_codin	XM_023028623.1	enoyl-CoA delta isomerase 1%2C peroxisomal
1961	gene-LOC11140108	0.5	3.2595635069	-1.004876782	0.980334337	-2.70467878390	protein_codin	XM_023028631.1	nuclear intron maturase 2%2C mitochondrial
1962	gene-LOC11140110	1.85599181786667	0.5	0.9346285653	0.972602995	1.892190350363	protein_codin	XM_023028658.1	ultraviolet-B receptor UVR8-like%2C transcript variant X1
1963	gene-LOC11140110	6.6580633174	0.9729997351	1.0864466465	0.969119778	2.774591273059	protein_codin	XM_023028663.1	NAC domain-containing protein 2-like
1964	gene-LOC11140111	48.7172295151667	27.807766244533	1.2430404094	0.985592055	0.808944231559	protein_codin	XM_023028695.1	probable polygalacturonase
1965	gene-LOC11140113	33.6000001518	10.665070108666	1.5810553509	0.962590423	1.655567790009	protein_codin	XR_002703932.1	pentatricopeptide repeat-containing protein At1g03100%2C mitochondrial%2C transcript variant X2
1966	gene-LOC11140113	147.027884446	199.67826623956	-1.507113153	0.999998523	-0.44158751868	protein_codin	XM_023028719.1	UDP-glucuronate:xylan alpha-glucuronosyltransferase 1%2C transcript variant X3
1967	gene-LOC11140113	2.91641583476667	0.5	0.7549277249	0.954171078	2.544196439823	protein_codin	XM_023028735.1	autophagy-related protein 13b%2C transcript variant X3
1968	gene-LOC11140114	0.5	2.3000030115333	-0.746106397	0.952051421	-2.20163575017	protein_codin	XM_023028742.1	probable mediator of RNA polymerase II transcription subunit 26c%2C transcript variant X1
1969	gene-LOC11140115	181.786824918867	14.794155950833	4.6406225734	0.999999999	3.619148349265	protein_codin	XM_023028758.1	2-hydroxy-6-oxononadienedioate/2-hydroxy-6-oxononatrienedioate hydrolase-like%2C transcript variant X3
1970	gene-LOC11140118	9.5330905684	0.9729997351	1.2785842445	0.983338661	3.292432685340	protein_codin	XM_023028814.1	G-type lectin S-receptor-like serine/threonine-protein kinase LECRK3
1971	gene-LOC11140120	5.91767242303333	1.0801683049	1.0459007349	0.967472247	2.453773715936	protein_codin	XM_023028828.1	nicastrin%2C transcript variant X4
1972	gene-LOC11140120	0.5	2.9857282704666	-1.042489693	0.983556510	-2.57808287254	protein_codin	XM_023028832.1	putative pentatricopeptide repeat-containing protein At1g09680
1973	gene-LOC11140121	0.5	2.3000030115333	-0.746106397	0.952051421	-2.20163575017	protein_codin	XM_023028842.1	DNA repair protein RAD4%2C transcript variant X1
1974	gene-LOC11140122	6.59573638573333	1.339697601	1.0361967106	0.968099290	2.299626349535	protein_codin	XM_023028860.1	butyrate--CoA ligase AAE11%2C peroxisomal-like
1975	gene-LOC11140124	0.5	4.2650610740333	-1.370789467	0.982763920	-3.09256640052	protein_codin	XM_023028895.1	3-hydroxy-3-methylglutaryl-coenzyme A reductase-like
1976	gene-LOC11140125	22.91247246	8.3462641790666	0.9454698260	0.974761746	1.456930655538	protein_codin	XM_023028916.1	lysine histidine transporter-like 8
1977	gene-LOC11140127	0.5	2.4599500583333	-0.805478126	0.958483519	-2.29862902640	protein_codin	XM_023028952.1	thaumatin-like protein 1
1978	gene-LOC11140129	4.2366811426	0.5	1.1129209246	0.975554793	3.082934553931	protein_codin	XM_023028975.1	receptor-like protein 2
1979	gene-LOC11140131	1.85599181786667	5.2790116621666	-0.751608759	0.952711581	-1.50807750278	protein_codin	XM_023029000.1	50S ribosomal protein L31%2C chloroplastic-like
1980	gene-LOC11140131	24.9315397837333	37.616354495566	-0.742949638	0.951617566	-0.59338805297	protein_codin	XM_023029023.1	pentatricopeptide repeat-containing protein At1g06143-like%2C transcript variant X3
1981	gene-LOC11140132	5.87052528163333	1.1797505542	1.1868921416	0.982475631	2.315007749080	protein_codin	XM_023029021.1	exocyst complex component SEC3A-like
1982	gene-LOC11140134	2.7537319571	10.103826507266	-0.853668860	0.953561983	-1.87544172964	protein_codin	XR_002703991.1	bZIP transcription factor 46-like%2C transcript variant X2
1983	gene-LOC11140136	3.963931269	0.5	0.8397074612	0.960564421	2.986931947707	protein_codin	XM_023029064.1	probable E3 ubiquitin-protein ligase RNF144A-A
1984	gene-LOC11140136	0.5	3.2051734141666	-0.858177733	0.951982431	-2.68040241518	protein_codin	XM_023029070.1	receptor protein-tyrosine kinase CEPR1-like
1985	gene-LOC11140137	57.5380727880333	32.359019010133	0.8042719108	0.959600294	0.830349027500	protein_codin	XM_023029081.1	serine carboxypeptidase-like 11%2C transcript variant X2
1986	gene-LOC11140138	0.5	2.4599500583333	-0.805478126	0.958483519	-2.29862902640	protein_codin	XM_023029089.1	casein kinase II subunit alpha-2-like%2C transcript variant X2
1987	gene-LOC11140138	0.5	2.4055599655666	-0.761230258	0.953631963	-2.26637276323	protein_codin	XM_023029100.1	blue copper protein-like
1988	gene-LOC11140139	0.798567038966667	3.6857594697333	-0.792153565	0.956751752	-2.20647649394	protein_codin	XM_023029102.1	queuine tRNA-ribosyltransferase catalytic subunit 1-like
1989	gene-LOC11140139	4.94064979323333	0.5	1.1610081240	0.983153982	3.304700797240	protein_codin	XM_023029103.1	thylakoid lumenal 29 kDa protein%2C chloroplastic
1990	gene-LOC11140141	0.5	2.4599500583333	-0.805478126	0.958483519	-2.29862902640	protein_codin	XM_023029134.1	auxin-induced protein PCNT115-like
1991	gene-LOC11140141	3.7784936512	9.5168167971	-0.824569018	0.959471953	-1.33266790013	protein_codin	XM_023029135.1	uncharacterized LOC111401413%2C transcript variant X1
1992	gene-LOC11140143	2.0762599173	0.5	0.6996756982	0.952603165	2.053987059267	protein_codin	XM_023029164.1	transcription factor MYBS1-like
1993	gene-LOC11140143	2.84443507903333	0.5	0.8095492150	0.959887199	2.508142153549	protein_codin	XM_023029167.1	zinc finger BED domain-containing protein DAYSLEEPER
1994	gene-LOC11140145	2.44818289113333	0.5	0.9691321958	0.976035677	2.291711338371	protein_codin	XM_023029195.1	biotin carboxyl carrier protein of acetyl-CoA carboxylase-like%2C transcript variant X2
1995	gene-LOC11140146	2.43886546933333	0.5	0.6809304862	0.950254362	2.286210179586	protein_codin	XM_023029204.1	uncharacterized LOC111401465
1996	gene-LOC11140148	10.8195089456667	2.9932536982	1.1356594994	0.981072927	1.853848555329	protein_codin	XM_023029221.1	auxin-induced protein 22D-like
1997	gene-LOC11140150	0.9255244066	7.1716216289666	-1.433365248	0.998592406	-2.95395643398	protein_codin	XR_002704026.1	uncharacterized LOC111401501%2C transcript variant X4
1998	gene-LOC11140150	7.29094724073333	2.4523637379	0.8278569951	0.960360505	1.571933284465	protein_codin	XM_023029271.1	phospholipid:diacylglycerol acyltransferase 1
1999	gene-LOC11140150	4.96287579773333	1.0801683049	0.9379414574	0.973335852	2.199920228416	protein_codin	XM_023029274.1	polyadenylate-binding protein-interacting protein 3-like%2C transcript variant X2
2000	gene-LOC11140151	8.97740926146667	17.4345284825	-0.986037990	0.970282513	-0.95757627576	protein_codin	XM_023029285.1	protein LNK3-like%2C transcript variant X1
2001	gene-LOC11140151	5.84372456526667	11.8900737122	-0.981293703	0.967064942	-1.02479757335	protein_codin	XM_023029293.1	histone H1-like
2002	gene-LOC11140152	1.8291911015	0.5	0.7379989413	0.953392084	1.871205805969	protein_codin	XM_023029303.1	uncharacterized LOC111401528
2003	gene-LOC11140153	5.30325534526667	13.461238862366	-0.735787635	0.950468282	-1.34386107064	protein_codin	XM_023029324.1	uncharacterized LOC111401535%2C transcript variant X2
2004	gene-LOC11140154	0.5	2.3000030115333	-0.746106397	0.952051421	-2.20163575017	protein_codin	XM_023029327.1	basic form of pathogenesis-related protein 1-like
2005	gene-LOC11140155	96.5919766793333	187.76490473493	-5.519120129	0.999999999	-0.95895217002	protein_codin	XM_023029338.1	beta-galactosidase-like%2C transcript variant X2
2006	gene-LOC11140156	2.74786958863333	0.5	0.7977912973	0.959106193	2.458313536799	lncRNA	XR_002704055.1	uncharacterized LOC111401563
2007	gene-LOC11140162	3.7784936512	9.6512413059666	-0.845610367	0.956062042	-1.35290330955	protein_codin	XM_023029446.1	putative pectinesterase/pectinesterase inhibitor 26

AQRNA_Control_vs_AQRNA_G7

2008	gene-LOC11140169	3.92220628296667	0.5	0.9656238773	0.976179378	2.971665414058	protein_codin	XM_023029551.1	calmodulin-binding protein 60 A-like%2C transcript variant X2
2009	gene-LOC11140170	0.5	2.8852793205666	-0.859212762	0.951607986	-2.52871099127	protein_codin	XM_023029559.1	mitogen-activated protein kinase kinase 3%2C transcript variant X2
2010	gene-LOC11140171	2.03556706723333	9.3577364509	-1.304233280	0.970702595	-2.20072884181	protein_codin	XM_023029572.1	vignain-like
2011	gene-LOC11140172	3.43623452416667	0.9398908767333	0.7992232046	0.959229098	1.870263332500	protein_codin	XM_023029583.1	uncharacterized LOC111401720%2C transcript variant X1
2012	gene-LOC11140172	5.50146543826667	2.0057532413	0.7103166179	0.953141581	1.455671835237	protein_codin	XM_023029596.1	probable anion transporter 6%2C chloroplastic%2C transcript variant X3
2013	gene-LOC11140173	5.85116240763333	1.9265855257666	0.7733922258	0.956194793	1.602677030716	protein_codin	XR_002704094.1	hemK methyltransferase family member 2-like%2C transcript variant X6
2014	gene-LOC11140173	3.11979256253333	0.5	1.1572135605	0.983113460	2.641450106346	protein_codin	XM_023029614.1	glycerophosphodiester phosphodiesterase GDPD6-like%2C transcript variant X2
2015	gene-LOC11140179	24.1857333749667	11.648541526266	0.8913773017	0.963643154	1.054006953436	protein_codin	XM_023029701.1	pre-mRNA-processing-splicing factor 8A-like
2016	gene-LOC11140180	15.6965118539667	8.0767311389	0.8139104188	0.960058755	0.958600572490	protein_codin	XM_023029721.1	F-box/LRR-repeat protein At5g63520-like
2017	gene-LOC11140181	2.96813768803333	0.5	0.7266124400	0.953311660	2.569558018250	protein_codin	XM_023029732.1	F-box/LRR-repeat protein 14
2018	gene-LOC11140182	31.8502276483333	18.476358139433	0.9360295803	0.972915173	0.785623267848	protein_codin	XM_023029741.1	protein TSS%2C transcript variant X1
2019	gene-LOC11140182	2.08916849996667	0.5	0.7050067510	0.952935613	2.062928856341	protein_codin	XM_023029748.1	ELMO domain-containing protein C
2020	gene-LOC11140182	5.8118447367	0.9398908767333	0.8643034769	0.961473536	2.628430990160	lncRNA	XR_002704117.1	uncharacterized LOC111401826
2021	gene-LOC11140182	146.269959351833	73.632676286133	1.6324585864	0.981088412	0.990215457500	lncRNA	XR_002704119.1	uncharacterized LOC111401827%2C transcript variant X2
2022	gene-LOC11140183	3.72489221846667	0.5	1.2200150692	0.983824698	2.897198681109	protein_codin	XM_023029760.1	photosystem I reaction center subunit psaK%2C chloroplastic-like
2023	gene-LOC11140183	6.25249370766667	1.1329467819	0.9682858026	0.976079663	2.464351605726	protein_codin	XM_023029768.1	ADP-ribosylation factor GTPase-activating protein AGD12-like
2024	gene-LOC11140184	26.3306869927	11.6237639035	0.8866937031	0.963115484	1.179667858134	protein_codin	XM_023029771.1	protein FAR-RED IMPAIRED RESPONSE 1-like
2025	gene-LOC11140185	10.9063653861333	23.142959338933	-0.993677172	0.974949907	-1.08540296200	protein_codin	XM_023029796.1	photosystem I reaction center subunit III%2C chloroplastic
2026	gene-LOC11140186	4.909274365	1.8126973361	0.8006088656	0.959338738	1.437371736838	protein_codin	XM_023029813.1	oxygen-dependent coproporphyrinogen-III oxidase%2C chloroplastic-like%2C transcript variant X2
2027	gene-LOC11140190	22.1388817838333	10.876184016766	1.1626256692	0.983144706	1.025409888669	protein_codin	XM_023029877.1	patellin-3-like
2028	gene-LOC11140191	56.0704484617	100.9097389925	-2.255857599	0.999999999	-0.84775290452	protein_codin	XM_023029895.1	putative leucine-rich repeat receptor-like serine/threonine-protein kinase At2g24130%2C transcript variant X1
2029	gene-LOC11140194	22.2302254136	13.810745425966	0.7055298969	0.952959874	0.686731387088	protein_codin	XM_023029935.1	dehydrin DHN1-like
2030	gene-LOC11140195	229.0704037783	204.69815947113	0.7579444307	0.954426416	0.162292941903	protein_codin	XM_023029949.1	probable methyltransferase PMT27
2031	gene-LOC11140195	2.15854164583333	0.5	0.9576639473	0.976078277	2.110056927711	protein_codin	XM_023029950.1	aldehyde dehydrogenase family 3 member F1-like
2032	gene-LOC11140196	3.7562676467	0.7067508191	0.7069954225	0.953022737	2.410026308322	protein_codin	XM_023029953.1	aldehyde dehydrogenase family 3 member F1-like
2033	gene-LOC11140198	2.5612481251	0.5	0.8691339282	0.961743624	2.356847023146	protein_codin	XM_023029982.1	probable alpha-mannosidase At5g66150
2034	gene-LOC11140199	0.5	2.5127285353666	-0.801158618	0.957947684	-2.32925481692	protein_codin	XM_023030000.1	ADP-ribosylation factor 2-like%2C transcript variant X2
2035	gene-LOC11140201	68.783138437	51.319992244466	1.0497776716	0.967287795	0.422533992249	protein_codin	XM_023030028.1	ferredoxin--NADP reductase%2C leaf-type isozyme%2C chloroplastic-like
2036	gene-LOC11140202	51.9474797017667	74.566653350833	-0.840431294	0.957367403	-0.52147683629	protein_codin	XM_023030054.1	uncharacterized LOC111402024
2037	gene-LOC11140202	2.7020976264	0.5	0.6901994521	0.951655310	2.434079799970	protein_codin	XM_023030058.1	ubiquitin-fold modifier 1
2038	gene-LOC11140203	2.08916849996667	0.5	0.7050067510	0.952935613	2.062928856341	protein_codin	XM_023030075.1	ethylene-responsive transcription factor ERF014-like
2039	gene-LOC11140204	2.74427842776667	7.9703683769666	-0.785368143	0.955898971	-1.53821954344	protein_codin	XM_023030089.1	uncharacterized protein At4g08330%2C chloroplastic-like%2C transcript variant X2
2040	gene-LOC11140204	0.5	2.4599500583333	-0.805478126	0.958483519	-2.29862902640	protein_codin	XM_023030090.1	filament-like plant protein
2041	gene-LOC11140207	20.9438070363333	4.5321766906	1.4724528695	0.966681654	2.208247698322	protein_codin	XM_023030120.1	glucan endo-1%2C3-beta-glucosidase 13-like
2042	gene-LOC11140213	0.5	2.9329497934333	-1.062324105	0.979286965	-2.55235237508	protein_codin	XM_023030226.1	60S ribosomal protein L26-1-like
2043	gene-LOC11140220	1.08820828426667	4.1051140272333	-0.736262403	0.950552191	-1.91546757967	protein_codin	XM_023030364.1	adagio protein 1-like%2C transcript variant X1
2044	gene-LOC11140220	2.44531976066667	0.5	0.6834549292	0.950682551	2.290023130602	protein_codin	XM_023030366.1	1-deoxy-D-xylulose 5-phosphate reductoisomerase%2C chloroplastic-like
2045	gene-LOC11140220	2.5612481251	0.5	0.8691339282	0.961743624	2.356847023146	protein_codin	XM_023030371.1	pantothenate kinase 2-like%2C transcript variant X2
2046	gene-LOC11140221	0.5	4.9139254119666	-1.005574459	0.980611402	-3.29687595932	protein_codin	XM_023030376.1	uncharacterized LOC111402214
2047	gene-LOC11140221	2.9968179839	0.5	0.9546740034	0.975885258	2.583431462330	protein_codin	XM_023030379.1	uncharacterized LOC111402217
2048	gene-LOC11140222	0.5	2.8257812237	-1.119397939	0.975307321	-2.49864977434	protein_codin	XM_023030385.1	histone deacetylase 6-like
2049	gene-LOC11140223	10.9341496535	4.1051140272333	0.8123689575	0.960004904	1.413346825431	protein_codin	XM_023030398.1	RNA-binding protein 25%2C transcript variant X1
2050	gene-LOC11140226	0.5	4.4333393564333	-1.092915347	0.974338586	-3.14839379992	protein_codin	XM_023030432.1	putative clathrin assembly protein At5g35200%2C transcript variant X2
2051	gene-LOC11140226	0.938432989266667	8.6389023335666	-1.380886164	0.986295043	-3.20252237870	protein_codin	XM_023030441.1	pheophytinase%2C chloroplastic
2052	gene-LOC11140226	334.819972899733	569.56692787163	-3.278506145	0.999999999	-0.76647978703	protein_codin	XM_023030444.1	arogenate dehydrogenase 2%2C chloroplastic-like
2053	gene-LOC11140227	1.85599181786667	0.5	0.9346285653	0.972602995	1.892190350363	protein_codin	XM_023030449.1	cytochrome P450 71A1-like
2054	gene-LOC11140230	4.6167699892	0.9729997351	0.8057895342	0.959689914	2.246372540729	protein_codin	XM_023030525.1	phospholipase SGR2-like
2055	gene-LOC11140231	0.5	5.1241726195	-1.274715272	0.974370993	-3.35731907698	protein_codin	XM_023030536.1	uncharacterized LOC111402313
2056	gene-LOC11140231	0.662683877666667	3.5249457223333	-0.793921214	0.956985889	-2.41120831698	protein_codin	XM_023030541.1	uncharacterized LOC111402319

2057	gene-LOC11140240	13.6377352312333	29.005635694366	-1.3166834491	0.9711283764	-1.0887291569	protein_codin	XM_023030687.1	serine acetyltransferase 1%2C chloroplastic-like%2C transcript variant X1
2058	gene-LOC11140243	5.91023458066667	1.0801683049	1.0505932135	0.9672520470	2.451959271553	protein_codin	XM_023030745.1	glycolipid transfer protein 1-like%2C transcript variant X3
2059	gene-LOC11140243	3.00066466536667	0.5	0.7984631085	0.9591672297	2.585282101793	protein_codin	XM_023030727.1	polyadenylation and cleavage factor homolog 4-like
2060	gene-LOC11140243	4.09207248236667	0.7067508191	0.7418938169	0.9534783544	2.533558144381	protein_codin	XM_023030732.1	probable xyloglucan endotransglucosylase/hydrolase protein 8
2061	gene-LOC11140244	2.74786958863333	0.5	0.7977912973	0.9591061931	2.458313536799	protein_codin	XM_023030741.1	probable UDP-3-O-acylglucosamine N-acyltransferase 2%2C mitochondrial%2C transcript variant X1
2062	gene-LOC11140245	4.9558295835	0.6531665342333	1.1019682218	0.9725401231	2.923603798559	protein_codin	XR_002704263.1	uncharacterized LOC111402457%2C transcript variant X6
2063	gene-LOC11140245	3.11979256253333	0.5	1.1572135605	0.9831134604	2.641450106346	protein_codin	XR_002704266.1	uncharacterized LOC111402458%2C transcript variant X5
2064	gene-LOC11140246	2.75718701046667	0.5	1.1062460770	0.9736904163	2.463197123685	protein_codin	XM_023030803.1	uncharacterized ATP-dependent helicase YprA%2C transcript variant X4
2065	gene-LOC11140247	2.0762599173	0.5	0.6996756982	0.9526031653	2.053987059267	protein_codin	XM_023030822.1	uncharacterized protein At1g03900-like%2C transcript variant X1
2066	gene-LOC11140249	76.6123712127	58.091807149166	0.7167256928	0.9532683496	0.399242663835	protein_codin	XM_023030845.1	ABC transporter G family member 26%2C transcript variant X1
2067	gene-LOC11140249	2.5612481251	0.5	0.8691339282	0.9617436241	2.356847023146	protein_codin	XM_023030852.1	peptidyl-prolyl cis-trans isomerase FKBP16-1%2C chloroplastic%2C transcript variant X1
2068	gene-LOC11140251	0.6358831613	2.1861148219	-0.7722623857	0.9545614391	-1.7815355673427	protein_codin	XM_023030888.1	NAC transcription factor 56-like%2C transcript variant X1
2069	gene-LOC11140252	121.6026861815	77.821230639066	2.1088424207	1	0.643939398104	protein_codin	XM_023030905.1	putative late blight resistance protein homolog R1C-3%2C transcript variant X2
2070	gene-LOC11140253	121.589347033033	85.742971363766	1.0436530995	0.9675989864	0.503926514845	protein_codin	XM_023030910.1	squamosa promoter-binding protein 2-like
2071	gene-LOC11140254	10.4236483859	0.9729997351	1.7333184991	0.9679201797	3.421277101896	protein_codin	XM_023030926.1	transcription factor TCP4-like
2072	gene-LOC11140254	4.282844733	0.9729997351	0.6845445769	0.9508570212	2.138058058462	protein_codin	XM_023030928.1	malate dehydrogenase%2C mitochondrial-like
2073	gene-LOC11140259	2.66845099056667	0.5	0.8904698014	0.9635352514	2.416002514674	protein_codin	XM_023030979.1	60S ribosomal protein L23-like
2074	gene-LOC11140267	33.1117571853667	17.540769458966	1.2029005722	0.9826277742	0.916631538457	protein_codin	XM_023031057.1	ATP-dependent Clp protease ATP-binding subunit ClpA homolog CD4B%2C chloroplastic
2075	gene-LOC11140284	0.992034422	4.4250081208666	-0.8296200802	0.9591243059	-2.1572180170	protein_codin	XM_023031145.1	DNA (cytosine-5)-methyltransferase CMT2-like
2076	gene-LOC11140287	267.188084020967	150.2317469942	2.1072415658	1	0.830665952747	protein_codin	XM_023031181.1	MADS-box protein AGL71-like%2C transcript variant X3
2077	gene-LOC11140288	22.5570558706667	34.177417894066	-0.7391132137	0.9510302125	-0.5994646248	protein_codin	XM_023031197.1	ethylene-responsive transcription factor ERF061
2078	gene-LOC11140291	0.992034422	5.0738724587666	-0.7414394361	0.9513918515	-2.3546251692	protein_codin	XM_023031234.1	rhodanese-like domain-containing protein 14%2C chloroplastic%2C transcript variant X1
2079	gene-LOC11140292	47.0639672762333	110.0276766586	-3.2764639605	0.9999999999	-1.2251716254	protein_codin	XM_023031245.1	uncharacterized LOC111402921
2080	gene-LOC11140292	118.9186291555	61.585377414266	1.2600210857	0.9854262130	0.949314988713	protein_codin	XM_023031249.1	putative aminoacrylate hydrolase RutD%2C transcript variant X2
2081	gene-LOC11140292	592.198079253433	644.02046753413	-0.9586372512	0.9510434064	-0.1210267279	lncRNA	XR_002704373.1	uncharacterized LOC111402927%2C transcript variant X2
2082	gene-LOC11140294	2.5483395424	0.5	0.7722236578	0.9560425242	2.349557516016	protein_codin	XM_023031271.1	probable pectinesterase/pectinesterase inhibitor 34
2083	gene-LOC11140294	0.629428869966667	4.559371737	-1.1121050967	0.9746159144	-2.8567197832	protein_codin	XM_023031382.1	protein STRUBBELIG-RECEPTOR FAMILY 6%2C transcript variant X2
2084	gene-LOC11140297	76.4446820573667	104.6397021328	-1.0297163199	0.9847928952	-0.4529422913	protein_codin	XM_023031308.1	licodione synthase-like
2085	gene-LOC11140299	2.44818289113333	0.7331400576333	0.6796074784	0.9500228736	1.739550598950	protein_codin	XM_023031337.1	putative glycerol-3-phosphate transporter 1
2086	gene-LOC11140303	1.12146329196667	4.4793982135666	-0.8228593422	0.9595127272	-1.9979225273	protein_codin	XM_023031376.1	U6 snRNA phosphodiesterase
2087	gene-LOC11140303	5.0422943958	1.339697601	0.7925431887	0.9585984633	1.912172963158	protein_codin	XM_023031377.1	transcription factor bHLH51-like
2088	gene-LOC11140303	7.37134938983333	1.0801683049	1.2026515197	0.9826181318	2.770672620018	protein_codin	XM_023031385.1	uncharacterized LOC111403038%2C transcript variant X2
2089	gene-LOC11140305	1.4175588286	6.1321485029	-1.0778464165	0.9760545544	-2.1129840271	protein_codin	XM_023031400.1	cryptochrome-1
2090	gene-LOC11140305	2.66845099056667	0.5	0.8904698014	0.9635352514	2.416002514674	protein_codin	XM_023031411.1	uncharacterized LOC111403057
2091	gene-LOC11140306	0.5	2.0865325725666	-0.7450041296	0.9519056062	-2.0611074424	protein_codin	XM_023031415.1	receptor-like kinase TMK4
2092	gene-LOC11140312	131.2333257913	92.5842233969	1.2289162361	0.9846342317	0.503295848474	protein_codin	XM_023031513.1	protein NUCLEAR FUSION DEFECTIVE 4-like
2093	gene-LOC11140312	1.4175588286	5.5188104469666	-0.8267180637	0.9593583116	-1.9609487263	protein_codin	XM_023031517.1	protein SHOOT GRAVITROPISM 6
2094	gene-LOC11140319	38.375215709	18.316593770766	1.2360734066	0.9852045553	1.067023621101	protein_codin	XM_023031597.1	laccase-4-like
2095	gene-LOC11140321	0.5	2.9329497934333	-1.0623241055	0.9792869654	-2.5523523750	protein_codin	XM_023031624.1	uncharacterized protein At4g22758-like
2096	gene-LOC11140325	1.4175588286	9.9438185677666	-1.6040564166	0.9999999996	-2.8103913654	protein_codin	XM_023031685.1	wall-associated receptor kinase 2-like
2097	gene-LOC11140326	4.71392740246667	0.7331400576333	1.0070939703	0.9711487741	2.684768800900	lncRNA	XR_002704427.1	uncharacterized LOC111403269
2098	gene-LOC11140327	4.38359330713333	1.1329467819	0.7549314689	0.9541713699	1.952033862036	protein_codin	XR_002704432.1	putative disease resistance RPP13-like protein 3%2C transcript variant X4
2099	gene-LOC11140329	2.74329487676667	0.5	0.7654050307	0.9551999362	2.455909705811	protein_codin	XM_023031739.1	cyclic pyranopterin monophosphate synthase%2C mitochondrial-like%2C transcript variant X1
2100	gene-LOC11140329	3.29936781186667	0.5	0.9977881427	0.9724866625	2.722189617786	protein_codin	XM_023031779.1	ubiquitin-conjugating enzyme E2 34-like
2101	gene-LOC11140331	4.2505732763	0.7331400576333	1.0432316472	0.9676238039	2.535496691734	protein_codin	XM_023031767.1	pre-mRNA-splicing factor ATP-dependent RNA helicase DEAH10%2C transcript variant X1
2102	gene-LOC11140331	4.90282007366667	1.0801683049	0.9281385802	0.9710448377	2.182355696399	protein_codin	XM_023031771.1	ribonuclease H2 subunit B
2103	gene-LOC11140334	1.85599181786667	0.5	0.9346285653	0.9726029951	1.892190350363	protein_codin	XM_023031814.1	probable alpha%2Calpha-trehalose-phosphate synthase [UDP-forming] 9%2C transcript variant X3
2104	gene-LOC11140336	4.30417470906667	1.605946517	0.6861473784	0.9511003792	1.422312791515	protein_codin	XM_023031847.1	proteasome subunit beta type-5-like
2105	gene-LOC11140339	10.6855053638333	3.0468379831	0.8889211391	0.9633557380	1.810270449651	protein_codin	XM_023031885.1	F-box/kelch-repeat protein At1g23390

2106	gene-LOC11140339	162.6396174134	205.836803981	-1.238000730	0.982840107	-0.33982223513	protein_codin	XM_023031891.1	galactinol synthase 1-like
2107	gene-LOC11140340	4.88345719963333	0.5	1.2662222824	0.984930732	3.287902852471	protein_codin	XM_023031902.1	F-box/kelch-repeat protein At1g67480%2C transcript variant X2
2108	gene-LOC11140341	0.5	3.7317574341666	-1.044192130	0.983262146	-2.89985521344	protein_codin	XM_023031913.1	probable 2-oxoglutarate-dependent dioxygenase ANS%2C transcript variant X2
2109	gene-LOC11140341	10.1553371166667	3.4202554688666	0.8418103318	0.960612454	1.570062138794	protein_codin	XM_023031919.1	disease resistance protein RPM1-like
2110	gene-LOC11140341	18.0684756797333	8.7708485261333	0.9891690977	0.973784662	1.042686474013	protein_codin	XM_023031916.1	probable nucleolar protein 5-2
2111	gene-LOC11140343	13.5434409484	6.2912288491333	0.9186807458	0.968711094	1.106180579871	protein_codin	XM_023031946.1	protein ETHYLENE INSENSITIVE 3-like
2112	gene-LOC11140343	3.35883161303333	0.5	0.7748239116	0.956383475	2.747959471329	protein_codin	XM_023031947.1	myb-like protein X%2C transcript variant X1
2113	gene-LOC11140344	7.0613617195	3.0452263673666	0.6927678535	0.951957558	1.213396945892	protein_codin	XM_023032066.1	proteasome subunit alpha type-5-like%2C transcript variant X2
2114	gene-LOC11140345	12.7559029126667	29.296601340433	-1.246826583	0.981177891	-1.19956828708	protein_codin	XM_023031962.1	uncharacterized LOC111403451%2C transcript variant X3
2115	gene-LOC11140346	3.4223423905	0.8599173533333	0.8589872776	0.961212384	1.992714188270	lncRNA	XR_002704478.1	uncharacterized LOC111403460
2116	gene-LOC11140350	10.2541185887667	3.3123419839666	0.9069640675	0.966116406	1.630279950476	protein_codin	XM_023032030.1	splicing factor U2af large subunit B%2C transcript variant X1
2117	gene-LOC11140353	107.303354434433	82.087781543433	0.8978694165	0.964527088	0.386455773833	protein_codin	XM_023032075.1	uncharacterized LOC111403531
2118	gene-LOC11140355	12.2570225712	4.3194511668	0.9322092378	0.972034392	1.504688648587	protein_codin	XM_023032101.1	protein disulfide isomerase-like 1-4
2119	gene-LOC11140355	4.8685815149	1.1329467819	0.7676825342	0.955469578	2.103421402461	protein_codin	XM_023032104.1	probable galacturonosyltransferase-like 7
2120	gene-LOC11140357	0.5	3.2595635069	-1.004876782	0.980334337	-2.70467878390	protein_codin	XM_023032133.1	wee1-like protein kinase
2121	gene-LOC11140362	4.85567293223333	1.0801683049	0.8455539240	0.960711339	2.168415126611	protein_codin	XM_023032192.1	chaperone protein dnaJ 20%2C chloroplastic-like
2122	gene-LOC11140362	3.01357324803333	0.7067508191	0.7583141344	0.954459621	2.092201576218	protein_codin	XM_023032194.1	E3 ubiquitin-protein ligase RNF4-like%2C transcript variant X1
2123	gene-LOC11140363	6.42300705586667	2.4523637379	0.7559195591	0.954248544	1.389075903914	protein_codin	XM_023032215.1	26S proteasome regulatory subunit 4 homolog A-like
2124	gene-LOC11140366	3.34292379236667	0.5	1.1230474234	0.978300265	2.741110466643	protein_codin	XM_023032246.1	asparagine synthetase [glutamine-hydrolyzing]
2125	gene-LOC11140368	0.5	2.3000030115333	-0.746106397	0.952051421	-2.20163575017	protein_codin	XM_023032278.1	60S ribosomal protein L10-like
2126	gene-LOC11140369	91.1545563951333	117.40886147263	-0.811057221	0.959059498	-0.36515462360	protein_codin	XM_023032288.1	transcription repressor OFP2-like
2127	gene-LOC11140370	8.58253225273333	3.2595026142	0.7052939630	0.952949820	1.396751541452	protein_codin	XM_023032307.1	uncharacterized LOC111403709%2C transcript variant X1
2128	gene-LOC11140371	0.629428869966667	4.3458404053	-1.290897393	0.971515320	-2.78751993735	protein_codin	XM_023032314.1	tRNA (adenine(37)-N6)-methyltransferase%2C transcript variant X1
2129	gene-LOC11140371	7.3832744215	1.1329467819	1.1741702044	0.982863360	2.704180686801	protein_codin	XM_023032319.1	protein SUPPRESSOR OF MAX2 1%2C transcript variant X1
2130	gene-LOC11140372	0.5	2.4599500583333	-0.805478126	0.958483519	-2.29862902640	protein_codin	XM_023032331.1	protein FLX-like 4%2C transcript variant X1
2131	gene-LOC11140375	15.6161097048333	8.7444592876	0.8506473022	0.960874194	0.836594009961	protein_codin	XM_023032369.1	uncharacterized LOC111403752%2C transcript variant X1
2132	gene-LOC11140376	21.7462759827	39.3540958336	-0.790786385	0.956572974	-0.85574543134	protein_codin	XM_023032381.1	NADP-dependent glyceraldehyde-3-phosphate dehydrogenase
2133	gene-LOC11140376	8.60736586706667	4.3722296438	0.6990999994	0.952557967	0.977202615662	protein_codin	XR_002704530.1	formin-like protein 13%2C transcript variant X2
2134	gene-LOC11140378	60.5664664949333	97.361900420033	-2.213383301	1	-0.68483808283	lncRNA	XR_002704534.1	uncharacterized LOC111403785
2135	gene-LOC11140378	245.6661989487	322.6575454721	-1.560475126	0.999999999	-0.39330439197	protein_codin	XM_023032411.1	cellulose synthase A catalytic subunit 7 [UDP-forming]%2C transcript variant X2
2136	gene-LOC11140379	98.6436195652667	17.7544225761	4.0691780110	0.999999999	2.474047297422	lncRNA	XR_002704537.1	uncharacterized LOC111403790
2137	gene-LOC11140380	4.86956506596667	1.1329467819	0.8476614500	0.960774559	2.103712826345	protein_codin	XM_023032425.1	zinc finger protein ZAT10-like
2138	gene-LOC11140383	0.5	3.7853417190666	-1.078716400	0.975909185	-2.92042354437	protein_codin	XM_023032462.1	probable mediator of RNA polymerase II transcription subunit 26b%2C transcript variant X1
2139	gene-LOC11140386	7.10654175886667	1.2333348391333	0.9814742901	0.974860048	2.526583143590	protein_codin	XM_023032502.1	cytokinin dehydrogenase 1-like
2140	gene-LOC11140388	2.51469290656667	0.5	0.9399167264	0.973746235	2.330382229365	protein_codin	XR_002704584.1	N-alpha-acetyltransferase 50-like%2C transcript variant X3
2141	gene-LOC11140391	293.490168872967	366.5364439401	-1.251244583	0.980193460	-0.32064447183	protein_codin	XM_023032580.1	subtilisin-like protease SBT1.3
2142	gene-LOC11140392	19.9031375215667	34.2870038874	-0.960269454	0.952128980	-0.78466596779	protein_codin	XM_023032593.1	superoxide dismutase [Fe]%2C chloroplastic-like
2143	gene-LOC11140393	4.22475611093333	11.1975679407	-0.764626520	0.953917712	-1.40624545549	protein_codin	XM_023032604.1	bifunctional epoxide hydrolase 2%2C transcript variant X1
2144	gene-LOC11140395	3.6712907857	12.395376497833	-1.008986989	0.981812483	-1.75544279941	protein_codin	XM_023032653.1	fatty acid desaturase 4%2C chloroplastic-like
2145	gene-LOC11140397	0.5	3.3206732195333	-0.770359075	0.954396269	-2.73147575724	protein_codin	XM_023032666.1	calcium-dependent protein kinase 2-like%2C transcript variant X1
2146	gene-LOC11140400	2.48143789886667	0.6531665342333	0.8146658233	0.960083557	1.925653570107	lncRNA	XR_002704621.1	uncharacterized LOC111404001
2147	gene-LOC11140402	4.91572865633333	1.7863080976333	0.7995038036	0.959251199	1.460424348384	protein_codin	XM_023032734.1	probable LRR receptor-like serine/threonine-protein kinase At4g36180
2148	gene-LOC11140402	0.5	3.8916435882333	-1.174587827	0.983963765	-2.96037958857	protein_codin	XM_023032732.1	uncharacterized LOC111404021
2149	gene-LOC11140405	5.56797545366667	10.130960660966	-0.820308920	0.959526031	-0.86354622691	protein_codin	XM_023032768.1	probable small nuclear ribonucleoprotein G%2C transcript variant X2
2150	gene-LOC11140407	2.46109147383333	0.5	0.9626316258	0.976207875	2.299298280873	protein_codin	XM_023032808.1	phosphatidylinositol:ceramide inositolphosphotransferase 1-like%2C transcript variant X2
2151	gene-LOC11140407	2.51469290656667	0.5	0.9399167264	0.973746235	2.330382229365	protein_codin	XM_023032809.1	uncharacterized LOC111404079
2152	gene-LOC11140408	0.5	2.4055599655666	-0.761230258	0.953631963	-2.26637276323	protein_codin	XR_002704645.1	protein CIA1-like%2C transcript variant X4
2153	gene-LOC11140409	3.9044674678	0.5	1.0594868120	0.967046082	2.965125791912	protein_codin	XM_023032825.1	MLO-like protein 3%2C transcript variant X1
2154	gene-LOC11140411	0.5	2.1928344418	-0.754119384	0.952977800	-2.13279689317	protein_codin	XM_023032856.1	serine/threonine-protein kinase HT1-like

2155	gene-LOC11140411	10.1340071406	5.0390910919666	0.7401886053	0.953436605	1.007969309176	protein_codin	XM_023032858.1	AT-hook motif nuclear-localized protein 7-like
2156	gene-LOC11140411	3.2457663791	0.5	0.8667081315	0.961603331	2.698559162595	protein_codin	XM_023032860.1	uncharacterized FCP1 homology domain-containing protein C1271.03c-like
2157	gene-LOC11140412	10.4052690629333	3.2527829943333	0.9557062739	0.975955215	1.677567788281	protein_codin	XM_023032869.1	UDP-glycosyltransferase 86A1-like
2158	gene-LOC11140414	0.5	2.5126676426666	-0.801150444	0.957946613	-2.32921985466	protein_codin	XM_023032897.1	zinc finger protein 593-like
2159	gene-LOC11140414	0.5	4.9055941763666	-1.391398819	0.989888105	-3.29442788942	protein_codin	XR_002704660.1	nuclear nucleic acid-binding protein C1D-like%2C transcript variant X2
2160	gene-LOC11140415	27.6468015134	38.3640561677	-0.857251067	0.952314964	-0.47264267890	protein_codin	XM_023032914.1	ubiquitin carboxyl-terminal hydrolase 22
2161	gene-LOC11140415	1.43046741126667	7.0380638207	-1.160909070	0.981955583	-2.29869196567	protein_codin	XM_023032918.1	uncharacterized LOC111404155%2C transcript variant X1
2162	gene-LOC11140415	2.39171832793333	0.5	0.8150955481	0.960097733	2.258047493707	protein_codin	XM_023032921.1	histone acetyltransferase type B catalytic subunit
2163	gene-LOC11140416	8.56864011903333	3.3327565177333	0.8335641337	0.960448509	1.362350338578	protein_codin	XM_023032924.1	PAN domain-containing protein At5g03700
2164	gene-LOC11140419	0.5	2.1061412985333	-0.859223837	0.951603966	-2.07460222831	protein_codin	XM_023032965.1	dicer-like protein 4%2C transcript variant X1
2165	gene-LOC11140421	159.869715474	189.7607151249	-1.191905647	0.985425354	-0.24728468057	protein_codin	XM_023032995.1	beta-glucosidase
2166	gene-LOC11140422	4.18218368143333	1.0801683049	0.6932614629	0.952013296	1.953000305064	protein_codin	XM_023033017.1	splicing factor-like protein 1
2167	gene-LOC11140423	47.6829590659	70.641429146	-0.798766619	0.957634747	-0.56704076238	protein_codin	XM_023033022.1	methyl-CpG-binding domain-containing protein 13-like%2C transcript variant X4
2168	gene-LOC11140423	1.95614846913333	0.5	0.6824614660	0.950524364	1.968015873129	protein_codin	XM_023033029.1	uncharacterized LOC111404236
2169	gene-LOC11140426	3.6519279117	0.7067508191	0.9838878917	0.974540231	2.369384731897	lncRNA	XR_002704672.1	uncharacterized LOC111404261
2170	gene-LOC11140427	3.449534735	11.195150517066	-0.851401822	0.954312990	-1.69840023226	protein_codin	XM_023033090.1	beta-amylase 8
2171	gene-LOC11140427	2.85734366173333	0.7331400576333	0.7033091551	0.952839848	1.962513824770	protein_codin	XM_023033094.1	uncharacterized LOC111404272%2C transcript variant X4
2172	gene-LOC11140427	2.69426815593333	0.5	0.8963939323	0.964311812	2.429893446933	protein_codin	XM_023033100.1	rop guanine nucleotide exchange factor 14-like%2C transcript variant X1
2173	gene-LOC11140428	4.29126612636667	13.3285477547	-1.074488881	0.976654313	-1.63504431765	protein_codin	XM_023033114.1	carbamoyl-phosphate synthase large chain%2C chloroplastic-like
2174	gene-LOC11140437	3.6519279117	1.2061397927333	0.6911340107	0.951774574	1.598261160465	protein_codin	XM_023033189.1	protein CHUP1%2C chloroplastic
2175	gene-LOC11140437	3.7779017283	0.5	0.9663630597	0.976155042	2.917585174479	protein_codin	XM_023033190.1	E3 ubiquitin-protein ligase UPL7
2176	gene-LOC11140440	1.2305457369	4.1587592048333	-0.735306945	0.950383672	-1.75685487346	protein_codin	XM_023033213.1	putative clathrin assembly protein At1g25240
2177	gene-LOC11140440	2.13819522083333	7.9457125396	-0.823353976	0.959501002	-1.89378302090	protein_codin	XM_023033214.1	uncharacterized LOC111404401
2178	gene-LOC11140443	2.0430049096	0.5	0.7450638976	0.953588108	2.030692671054	protein_codin	XM_023033247.1	guanosine nucleotide diphosphate dissociation inhibitor At5g09550-like
2179	gene-LOC11140446	8.4517282036	2.9389244981666	0.7263675983	0.953311372	1.523958076648	protein_codin	XM_023033265.1	uncharacterized LOC111404463
2180	gene-LOC11140447	4.29126612636667	1.1329467819	0.7202756878	0.953296132	1.921323278976	protein_codin	XM_023033341.1	oligouridylate-binding protein 1B-like%2C transcript variant X2
2181	gene-LOC11140450	113.1402653769	134.23240843003	-0.967352473	0.957067891	-0.24662057095	protein_codin	XM_023033298.1	uncharacterized LOC111404502
2182	gene-LOC11140450	0.792112747633333	4.2650610740333	-1.079968518	0.975705255	-2.42878870037	protein_codin	XM_023033299.1	uncharacterized LOC111404505
2183	gene-LOC11140468	67.3416906074333	47.341867777166	0.7164611827	0.953265858	0.508383314561	protein_codin	XM_023033452.1	uncharacterized LOC111404680
2184	gene-LOC11140470	49.0711095682667	65.771270772766	-1.199365533	0.985624039	-0.42258365180	protein_codin	XM_023033472.1	uncharacterized LOC111404705
2185	gene-LOC11140471	0.992034422	5.6040137598666	-0.763347724	0.953811433	-2.49799841231	protein_codin	XM_023033484.1	probable zinc metalloprotease EGY2%2C chloroplastic
2186	gene-LOC11140494	2.08916849996667	0.5	0.7050067510	0.952935613	2.062928856341	protein_codin	XM_023033665.1	uncharacterized LOC111404940
2187	gene-LOC11140497	41.6539006936333	16.9043871814	1.3619094979	0.958551219	1.301053887048	protein_codin	XM_023033692.1	auxin-responsive protein SAUR50-like
2188	gene-LOC11140503	5.7975609749	18.045510007566	-0.840846411	0.957274589	-1.63812192431	protein_codin	XM_023033729.1	uncharacterized LOC111405037
2189	gene-LOC11140503	6.98292667243333	1.7131150868	0.7751812133	0.956430924	2.027209748389	protein_codin	XM_023033732.1	transcription factor PRE6-like
2190	gene-LOC11140504	2.18534236223333	0.5	0.9277477865	0.970947782	2.127859314327	protein_codin	XM_023033731.1	auxin-responsive protein IAA7-like
2191	gene-LOC11140509	6.239585125	1.6867258483	0.8700969090	0.961801971	1.887224601844	protein_codin	XM_023033813.1	PRA1 family protein F2-like
2192	gene-LOC11140512	6.6719554511	2.1861148219	0.8766106931	0.962238615	1.609740477823	protein_codin	XR_002704731.1	uncharacterized LOC111405123%2C transcript variant X3
2193	gene-LOC11140514	1.96905705183333	0.5	0.6815674662	0.950366313	1.977504912765	protein_codin	XM_023033904.1	N-terminal acetyltransferase A complex auxiliary subunit NAA15-like%2C transcript variant X1
2194	gene-LOC11140515	2.5612481251	0.5	0.8691339282	0.961743624	2.356847023146	protein_codin	XM_023033910.1	L10-interacting MYB domain-containing protein-like
2195	gene-LOC11140517	0.5	2.5059480228	-1.134616510	0.977430730	-2.32535649126	protein_codin	XM_023033943.1	26S proteasome regulatory subunit 8 homolog A
2196	gene-LOC11140517	0.938432989266667	7.8461302903	-1.141288395	0.978543652	-3.06365565729	protein_codin	XM_023033949.1	phosphatidylinositol 4-kinase gamma 7-like
2197	gene-LOC11140517	40.7407252434667	24.260672587233	1.0120985720	0.970495242	0.747852115218	protein_codin	XM_023033948.1	methyl-CpG-binding domain-containing protein 11-like%2C transcript variant X2
2198	gene-LOC11140520	0.992034422	5.4133143856333	-0.850929873	0.954466224	-2.44805009117	protein_codin	XM_023033983.1	F-box/LRR-repeat protein 14-like%2C transcript variant X3
2199	gene-LOC11140523	9.46239746926667	2.6454196431	1.0464330937	0.967445074	1.838709167413	protein_codin	XM_023034041.1	beta-glucosidase 44-like
2200	gene-LOC11140524	2.33910044623333	7.5714283532666	-0.991167699	0.973492570	-1.69461167534	protein_codin	XM_023034053.1	histone deacetylase 5
2201	gene-LOC11140527	5.68808690183333	1.8730012409	0.7112268166	0.953167454	1.602591651033	protein_codin	XM_023034078.1	two-component response regulator ARR15-like
2202	gene-LOC11140528	10.1381902243333	3.3591457562333	0.8301799150	0.960395579	1.593633836964	protein_codin	XM_023034095.1	uncharacterized LOC111405284%2C transcript variant X1
2203	gene-LOC11140528	2.2586982971	0.5	0.7919045808	0.958528247	2.175491577350	protein_codin	XM_023034102.1	E3 ubiquitin-protein ligase SIS3-like

AQRNA_Control_vs_AQRNA_G7

2204	gene-LOC11140529	0.9255244066	6.4248475501333	-1.1121211457	0.9746171594	-2.795319281810	protein_codin	XM_023034109.1	uncharacterized LOC111405293
2205	gene-LOC11140531	18.8879810666333	8.8260444267333	0.7067481774	0.9530120814	1.097629586130	protein_codin	XM_023034140.1	GDSL esterase/lipase At3g48460-like
2206	gene-LOC11140532	2.73496100596667	0.5	0.7931074243	0.9586591417	2.451520263767	protein_codin	XM_023034149.1	60S ribosomal protein L17-2-like
2207	gene-LOC11140533	2.49434648156667	0.7067508191	0.6794960032	0.9500016247	1.819388323964	protein_codin	XM_023034162.1	putative 3%2C4-dihydroxy-2-butanone kinase
2208	gene-LOC11140535	134.873024565767	86.2186007867	1.2284793843	0.9845949086	0.645530774462	protein_codin	XM_023034179.1	probable metal-nicotianamine transporter YSL8%2C transcript variant X1
2209	gene-LOC11140535	6.2673693924	1.8730012409	0.7772519706	0.9567057967	1.742508171220	protein_codin	XR_002704763.1	DNA-binding protein RHL1%2C transcript variant X10
2210	gene-LOC11140535	0.5	2.4055599655666	-0.7612302589	0.9536319638	-2.266372763231	protein_codin	XM_023034194.1	uncharacterized LOC111405359
2211	gene-LOC11140538	1.22161994323333	4.8519489987666	-0.7727636066	0.9546056008	-1.989768865881	protein_codin	XM_023034230.1	putative lysine-specific demethylase JMJ16%2C transcript variant X2
2212	gene-LOC11140540	9.35814525683333	0.8599173533333	1.2132546375	0.9832530594	3.443952708185	protein_codin	XM_023034267.1	protein TRIGALACTOSYLDIACYLGLYCEROL 3%2C chloroplastic-like%2C transcript variant X3
2213	gene-LOC11140540	2445.85277531957	3088.6899663085	-4.8482764477	0.9999999999	-0.336657499239	protein_codin	XM_023034268.1	eukaryotic initiation factor 4A-3
2214	gene-LOC11140542	5.0422943958	1.1329467819	0.8273944974	0.9603535781	2.154000258025	protein_codin	XM_023034288.1	V-type proton ATPase subunit H-like%2C transcript variant X2
2215	gene-LOC11140542	2.66845099056667	8.7428476719	-0.8120338136	0.9591390067	-1.712100748230	protein_codin	XM_023034289.1	brefeldin A-inhibited guanine nucleotide-exchange protein 5%2C transcript variant X1
2216	gene-LOC11140544	18.7681737240667	0.5	3.1687510037	0.9999999999	5.230216367463	protein_codin	XM_023034327.1	BTB/POZ domain-containing protein At5g03250-like
2217	gene-LOC11140545	7.6738992178	14.344049065833	-0.7410756452	0.9513364536	-0.902420605170	protein_codin	XM_023034355.1	caffeoylshikimate esterase-like
2218	gene-LOC11140546	4.67323455243333	0.8863065918666	1.1493195609	0.9827632614	2.398543700323	protein_codin	XM_023034357.1	vacuolar protein sorting-associated protein 53 A%2C transcript variant X1
2219	gene-LOC11140546	2.21859736993333	0.5	0.7780618088	0.9568138950	2.149647871633	protein_codin	XM_023034360.1	probable serine/threonine-protein kinase PBL7
2220	gene-LOC11140546	6.42202350486667	17.4871242814	-0.8623277851	0.9504625089	-1.445193210273	protein_codin	XM_023034361.1	two-component response regulator-like APRR3%2C transcript variant X1
2221	gene-LOC11140548	3.1941320484	0.5	0.8612726627	0.9613200286	2.675423956341	protein_codin	XM_023034383.1	uncharacterized LOC111405483
2222	gene-LOC11140548	1.24098281723333	7.1980108675	-1.2192592032	0.9850054615	-2.536115140520	protein_codin	XM_023034391.1	geranylgeranyl transferase type-1 subunit beta%2C transcript variant X2
2223	gene-LOC11140549	7.9496483294	1.8126973361	1.2238378027	0.9841739801	2.132752980814	protein_codin	XM_023034404.1	clathrin heavy chain 1
2224	gene-LOC11140554	2.69426815593333	0.5	0.8963939323	0.9643118126	2.429893446933	protein_codin	XM_023034491.1	ethylene-responsive transcription factor 2-like
2225	gene-LOC11140557	61.7443391242333	93.809348785833	-0.9863796901	0.9705053685	-0.603424832125	protein_codin	XM_023034526.1	pentatricopeptide repeat-containing protein At3g29230-like
2226	gene-LOC11140558	63.7453864564	41.664843684566	0.7451141814	0.9535902955	0.613490363847	protein_codin	XM_023034529.1	WD repeat-containing protein RUP2-like
2227	gene-LOC11140559	0.5	2.3000030115333	-0.7461063970	0.9520514212	-2.201635750178	protein_codin	XM_023034558.1	E3 ubiquitin ligase BIG BROTHER-like
2228	gene-LOC11140560	14.8618266768	5.1462596617333	1.1701828329	0.9829944410	1.530015292144	lncRNA	XR_002704839.1	uncharacterized LOC111405600
2229	gene-LOC11140561	1.96260276046667	0.5	0.7250116886	0.9533097872	1.972768194454	protein_codin	XM_023034575.1	UDP-glycosyltransferase 88B1-like
2230	gene-LOC11140563	4.29126612636667	17.757645807566	-1.2722996760	0.9749357682	-2.048965052418	protein_codin	XM_023034595.1	uncharacterized LOC111405631%2C transcript variant X1
2231	gene-LOC11140564	0.5	4.3534267257333	-1.0725158066	0.9770419690	-3.122151440967	protein_codin	XM_023034616.1	golgin subfamily A member 4%2C transcript variant X3
2232	gene-LOC11140567	8.1127238352	2.0601433340666	0.8047778535	0.9596302851	1.977441662409	protein_codin	XM_023034674.1	pentatricopeptide repeat-containing protein At3g18110%2C chloroplastic
2233	gene-LOC11140567	3.7585388543	10.503572338833	-0.9667524425	0.9566393036	-1.482636259041	protein_codin	XM_023034678.1	pentatricopeptide repeat-containing protein At5g42310%2C chloroplastic%2C transcript variant X3
2234	gene-LOC11140568	59.0617081826667	83.984101883366	-0.7375466795	0.9507711825	-0.507893170134	protein_codin	XM_023034680.1	late embryogenesis abundant protein At5g17165-like%2C transcript variant X1
2235	gene-LOC11140568	4.42428615716667	0.8599173533333	0.9379616990	0.9733400333	2.363174786106	protein_codin	XM_023034683.1	cellulose synthase-like protein D3
2236	gene-LOC11140568	0.6358831613	3.8472572395666	-0.7641913757	0.9538814154	-2.596996685210	protein_codin	XM_023034685.1	cellulose synthase-like protein D4
2237	gene-LOC11140572	0.5	3.3650595682333	-1.0240141174	0.9846634892	-2.750632043627	protein_codin	XM_023034741.1	uncharacterized LOC111405725
2238	gene-LOC11140573	63.5493048225333	45.645324682766	0.7275826718	0.9533128075	0.477409246672	protein_codin	XM_023034758.1	calvin cycle protein CP12-1%2C chloroplastic-like
2239	gene-LOC11140573	140.745821055	204.0577481542	-1.5822984669	0.9999999999	-0.535885404083	protein_codin	XM_023034765.1	uncharacterized LOC111405739
2240	gene-LOC11140574	1.06786185923333	6.025785741	-1.0683858679	0.9779045673	-2.496424346752	protein_codin	XM_023034768.1	LEC14B protein
2241	gene-LOC11140575	8.41945674693333	3.4127300411333	0.7130481763	0.9532096798	1.302800851785	protein_codin	XM_023034780.1	protease Do-like 2%2C chloroplastic
2242	gene-LOC11140577	0.6358831613	4.6392843676666	-1.2256645942	0.9844576660	-2.867068669020	protein_codin	XM_023034808.1	BTB/POZ and MATH domain-containing protein 2-like
2243	gene-LOC11140578	1.95614846913333	0.5	0.6824614660	0.9505243641	1.968015873129	protein_codin	XM_023034826.1	probable inactive histone-lysine N-methyltransferase SUVR1%2C transcript variant X1
2244	gene-LOC11140579	4.72683598513333	0.6531665342333	1.1575627632	0.9831206684	2.855352026278	protein_codin	XR_002704887.1	casein kinase 1-like protein 9%2C transcript variant X3
2245	gene-LOC11140579	120.065407532867	229.26440428846	-2.1799953893	0.9999999999	-0.933191827752	protein_codin	XM_023034834.1	carboxylesterase 1-like
2246	gene-LOC11140580	1.6507355107	6.6655130355	-0.8628522217	0.9502677840	-2.013606939091	protein_codin	XM_023034849.1	transcriptional corepressor LEUNIG_HOMOLOG-like%2C transcript variant X2
2247	gene-LOC11140581	21.4930892778333	32.6334477902	-0.8118004184	0.9591199366	-0.602478557813	protein_codin	XM_023034875.1	uncharacterized LOC111405819
2248	gene-LOC11140582	7.04845313683333	1.8270032764333	0.8587513464	0.9612014413	1.947827455215	protein_codin	XM_023034886.1	alcohol dehydrogenase class-3
2249	gene-LOC11140584	5.593792619	1.8730012409	0.6949144570	0.9521833149	1.578474913777	protein_codin	XM_023034907.1	E3 ubiquitin-protein ligase BRE1-like 2%2C transcript variant X3
2250	gene-LOC11140584	5.6086683037	12.075543296933	-0.7374569245	0.9507564458	-1.106357929791	protein_codin	XM_023034919.1	elongation factor P%2C transcript variant X1
2251	gene-LOC11140585	0.5	3.3206732195333	-0.7703590752	0.9543962690	-2.731475757241	protein_codin	XM_023034922.1	probable E3 ubiquitin-protein ligase RHC1A%2C transcript variant X1
2252	gene-LOC11140586	4.908290814	1.339697601	0.6998912519	0.9526178262	1.873313340899	protein_codin	XM_023034934.1	serine/threonine-protein phosphatase BSL3-like

2253	gene-LOC11140586	38.0866132406667	25.807121007333	0.6888170026	0.9514759427	0.561514799330	protein_codin	XM_023034948.1	abscisic-aldehyde oxidase-like%2C transcript variant X2
2254	gene-LOC11140589	0.5	2.3000030115333	-0.7461063970	0.9520514212	-2.201635750178	protein_codin	XM_023034995.1	uncharacterized protein C20orf24 homolog
2255	gene-LOC11140590	10.9728754015333	5.0118960455666	0.7898373228	0.9583003265	1.130513231830	protein_codin	XM_023034998.1	uncharacterized LOC111405901
2256	gene-LOC11140590	4.66032596973333	1.1329467819	0.8297441156	0.9603890181	2.040350773921	protein_codin	XM_023035007.1	uncharacterized LOC111405906%2C transcript variant X4
2257	gene-LOC11140591	2.87670653573333	0.5	0.8103728623	0.9599240265	2.524418053528	protein_codin	XM_023035013.1	tRNA-splicing endonuclease subunit Sen2-1-like%2C transcript variant X1
2258	gene-LOC11140599	11.6725734458	4.4522031672	0.7565522967	0.9543023124	1.390531332552	protein_codin	XM_023035154.1	probable low-specificity L-threonine aldolase 1%2C transcript variant X1
2259	gene-LOC11140603	2.4407450488	0.5	0.7250500176	0.9533098318	2.287321604401	protein_codin	XM_023035220.1	probable methionine--tRNA ligase
2260	gene-LOC11140607	301.161686431367	366.7569697691	-1.1439549878	0.9790059201	-0.284286139739	protein_codin	XM_023035272.1	uncharacterized LOC111406074
2261	gene-LOC11140608	1.95614846913333	0.5	0.6824614660	0.9505243641	1.968015873129	protein_codin	XM_023035282.1	dihydrodipicolinate reductase-like protein CRR1%2C chloroplastic%2C transcript variant X2
2262	gene-LOC11140609	1.96260276046667	10.155799176466	-1.0010685209	0.9787332971	-2.371463672568	protein_codin	XM_023035291.1	protein ROOT PRIMORDIUM DEFECTIVE 1
2263	gene-LOC11140610	4.8685815149	1.1329467819	0.7676825342	0.9554695787	2.103421402461	protein_codin	XM_023035303.1	60S ribosomal protein L36-2-like
2264	gene-LOC11140610	1.24098281723333	8.8788229037666	-1.2279461000	0.9842168347	-2.838885286272	protein_codin	XM_023035316.1	WD repeat-containing protein YMR102C-like%2C transcript variant X1
2265	gene-LOC11140614	5.555066871	12.878440869666	-0.8157889997	0.9593918742	-1.213081760175	protein_codin	XM_023035372.1	nascent polypeptide-associated complex subunit alpha-like protein 2
2266	gene-LOC11140616	8.5140551353	1.3133083625333	1.2497192144	0.9857318626	2.696640734444	protein_codin	XM_023035401.1	nodulin-related protein 1
2267	gene-LOC11140617	22.9347859871	10.585157477966	0.8876493447	0.9632182091	1.115494712335	protein_codin	XR_002704947.1	uncharacterized LOC111406176%2C transcript variant X6
2268	gene-LOC11140618	4.43719473986667	0.5	1.2207739825	0.9838932245	3.149647871633	protein_codin	XM_023035429.1	transcription factor BIM2-like%2C transcript variant X1
2269	gene-LOC11140619	0.6358831613	2.5859215462	-0.8408730076	0.9572686290	-2.023844895432	protein_codin	XM_023035459.1	proline-rich receptor-like protein kinase PERK10
2270	gene-LOC11140623	16.2544643685	4.0591769555	1.4299840532	0.9539643195	2.001576876573	protein_codin	XM_023035525.1	J protein JJJ2-like
2271	gene-LOC11140625	3.10688397983333	0.5	1.1604430177	0.9831506234	2.635468367998	protein_codin	XM_023035562.1	solute carrier family 25 member 44-like
2272	gene-LOC11140629	1.85599181786667	0.5	0.9346285653	0.9726029951	1.892190350363	protein_codin	XM_023035641.1	F-box protein SKIP14-like
2273	gene-LOC11140629	2.5483395424	0.5	0.7722236578	0.9560425242	2.349557516016	protein_codin	XR_002704987.1	actin-related protein 3-like%2C transcript variant X1
2274	gene-LOC11140630	1.32138496636667	5.7638390213	-0.7774591169	0.9550434472	-2.124979206813	protein_codin	XM_023035653.1	proteasome subunit beta type-7-A%2C transcript variant X1
2275	gene-LOC11140631	3.963931269	0.5	0.8397074612	0.9605644217	2.986931947707	protein_codin	XM_023035701.1	uncharacterized LOC111406317
2276	gene-LOC11140633	11.1433887496667	2.5127285353666	1.2183818977	0.9836788188	2.148861306768	protein_codin	XM_023035718.1	ubiquitin-like-specific protease 1D
2277	gene-LOC11140634	595.5625106672	949.8347367749	-1.2397725926	0.9825370149	-0.673423576180	protein_codin	XM_023035734.1	type 2 DNA topoisomerase 6 subunit B-like
2278	gene-LOC11140634	0.5	2.9389244981666	-0.7675941189	0.9541637835	-2.555288296206	protein_codin	XM_023035740.1	RNA-binding protein 48%2C transcript variant X1
2279	gene-LOC11140636	2.51469290656667	0.5	0.9399167264	0.9737462359	2.330382229365	protein_codin	XM_023035799.1	sucrose synthase 2-like
2280	gene-LOC11140637	4.43719473986667	1.3133083625333	0.6995416153	0.9525940580	1.756442173326	lncRNA	XR_002704994.1	uncharacterized LOC111406372
2281	gene-LOC11140637	4.76654528423333	0.7331400576333	1.0053046136	0.9713928379	2.700783263273	protein_codin	XM_023035808.1	2-alkenal reductase (NADP(+)-dependent)-like
2282	gene-LOC11140638	4.95642150636667	1.8662816210333	0.7118507157	0.9531818501	1.409132178425	protein_codin	XM_023035813.1	putative serine/threonine-protein kinase-like protein CCR3
2283	gene-LOC11140638	0.6358831613	4.3722296438	-1.3442276106	0.9751189741	-2.781535567342	protein_codin	XM_023035818.1	hydroxyproline O-galactosyltransferase GALT6-like
2284	gene-LOC11140639	0.5	5.2790116621666	-1.4468698822	0.9994364456	-3.400267853152	protein_codin	XM_023035820.1	thioredoxin H2-like
2285	gene-LOC11140639	5.46821043056667	1.7131150868	0.7869495085	0.9579593668	1.674446687864	protein_codin	XM_023035822.1	probable AMP deaminase
2286	gene-LOC11140643	15.2922299008333	4.6384785598	1.2368897891	0.9852605589	1.721075216584	protein_codin	XM_023035862.1	uncharacterized LOC111406435
2287	gene-LOC11140644	8.54282295366667	0.6531665342333	1.4541642939	0.9629966861	3.709190103757	protein_codin	XM_023035866.1	GDSL esterase/lipase APG-like
2288	gene-LOC11140662	3.11333827116667	0.5	1.2478099587	0.9857157605	2.638462337366	protein_codin	XM_023036010.1	protein EXPORTIN 1A-like
2289	gene-LOC11140663	9.71651249926667	2.2125040604	1.1265964388	0.9791773085	2.134758484359	protein_codin	XM_023036020.1	uncharacterized LOC111406631
2290	gene-LOC11140668	0.5	3.3387312224333	-0.8146153884	0.9593222924	-2.739299956931	protein_codin	XM_023036068.1	chromatin modification-related protein eaf-1-like
2291	gene-LOC11140673	0.5	2.3000030115333	-0.7461063970	0.9520514212	-2.201635750178	protein_codin	XM_023036112.1	uncharacterized LOC111406734
2292	gene-LOC11140673	3.92122273196667	0.9398908767333	0.8313903782	0.9604138808	2.060738419897	protein_codin	XM_023036116.1	uncharacterized LOC111406737
2293	gene-LOC11140678	3.38263309146667	8.6644248715	-0.7845063769	0.9557982890	-1.356957290225	protein_codin	XM_023036160.1	uncharacterized LOC111406784
2294	gene-LOC11140679	33.7340037336333	22.978983252766	0.7159801469	0.9532585818	0.553888590642	protein_codin	XM_023036168.1	pentatricopeptide repeat-containing protein At2g13600-like
2295	gene-LOC11140680	35.4601750534	56.754116106233	-1.0010065528	0.9787061473	-0.678525369719	protein_codin	XM_023036180.1	uncharacterized LOC111406808
2296	gene-LOC11140680	84.5984740825333	133.39834766433	-1.2734403880	0.9746695418	-0.657037250173	protein_codin	XM_023036181.1	uncharacterized LOC111406809
2297	gene-LOC11140681	4.56903092493333	0.7067508191	0.7665407200	0.9553333436	2.692614652082	protein_codin	XM_023036183.1	nucleolar complex protein 2 homolog
2298	gene-LOC11140684	2.43886546933333	0.5	0.6809304862	0.9502543622	2.286210179586	protein_codin	XM_023036217.1	uncharacterized LOC111406849
2299	gene-LOC11140685	24.1666193805667	9.1186216885666	1.2405621207	0.9854833807	1.406127993930	protein_codin	XM_023036218.1	midasin
2300	gene-LOC11140686	1.96905705183333	0.5	0.6815674662	0.9503663139	1.977504912765	protein_codin	XM_023036249.1	D-3-phosphoglycerate dehydrogenase 1%2C chloroplastic-like
2301	gene-LOC11140687	15.5870377809	7.4371256298333	0.7942908867	0.9587762261	1.067529731071	protein_codin	XM_023036240.1	alpha-glucan water dikinase%2C chloroplastic

AQRNA_Control_vs_AQRNA_G7

2302	gene-LOC11140688	196.666461925	169.47457841726	0.7765296435	0.956610007	0.214682069857	protein_codin	XM_023036245.1	2-methylene-furan-3-one reductase-like
2303	gene-LOC11140699	2.7537319571	0.5	0.6975232012	0.952426558	2.461388137184	protein_codin	XM_023036340.1	uncharacterized LOC111406996
2304	gene-LOC11140703	6.72654043483333	1.5327535062	0.8020946209	0.959450111	2.133738990071	protein_codin	XM_023036373.1	E3 SUMO-protein ligase SIZ1-like
2305	gene-LOC11140703	2.25224400576667	0.5	0.7304862915	0.953319521	2.171363135961	protein_codin	XM_023036376.1	protein 108-like
2306	gene-LOC11140713	9.61576392513333	22.6079222979	-1.521078795	0.999999761	-1.23335503008	protein_codin	XM_023036451.1	peroxisome biogenesis protein 5
2307	gene-LOC11140714	1.12146329196667	4.4250081208666	-0.805656361	0.958503508	-1.98029770434	protein_codin	XM_023036467.1	RNA polymerase II C-terminal domain phosphatase-like 1
2308	gene-LOC11140714	36.6393905652	63.393923925666	-1.270433948	0.975391893	-0.79094906315	protein_codin	XM_023036471.1	uncharacterized LOC111407145
2309	gene-LOC11140714	62.0381634532667	42.493886925033	0.7847678651	0.957689800	0.545900664869	protein_codin	XM_023036473.1	CBS domain-containing protein CBSX5-like
2310	gene-LOC11140718	3.34292379236667	0.5	1.1230474234	0.978300265	2.741110466643	protein_codin	XM_023036522.1	serine/threonine-protein kinase D6PK-like%2C transcript variant X2
2311	gene-LOC11140721	3.1792563637	0.5	0.8447531289	0.960688583	2.668689354861	protein_codin	XM_023036567.1	U-box domain-containing protein 17-like
2312	gene-LOC11140722	18.9848506626	8.8771503953333	1.0721753904	0.967424911	1.096680106074	protein_codin	XM_023036591.1	GDSL esterase/lipase At1g29670-like
2313	gene-LOC11140723	41.1753667771667	19.672676866266	1.1247793077	0.978740023	1.065588222217	protein_codin	XM_023036597.1	glucan endo-1%2C3-beta-glucosidase 3-like
2314	gene-LOC11140725	4.37068472446667	8.9843189651	-0.818253847	0.959489746	-1.03954983349	protein_codin	XM_023036637.1	transcription factor bHLH30-like
2315	gene-LOC11140725	2.15854164583333	0.6531665342333	0.7183454324	0.953283070	1.724534148007	protein_codin	XR_002705056.1	NEP1-interacting protein 1-like%2C transcript variant X10
2316	gene-LOC11140728	2.54794791426667	0.5	0.8179957286	0.960177446	2.349335785994	lncRNA	XR_002705074.1	uncharacterized LOC111407281
2317	gene-LOC11140728	1.06786185923333	5.1718430924	-1.028810005	0.984815999	-2.27595347699	protein_codin	XM_023036688.1	potassium transporter 10-like%2C transcript variant X2
2318	gene-LOC11140729	401.9036534906	239.10576734823	3.0108780444	1	0.749200763242	protein_codin	XM_023036703.1	uncharacterized protein C12B10.15c
2319	gene-LOC11140729	59.4281927129	78.915337428733	-0.733675209	0.950093220	-0.40915821210	lncRNA	XR_002705079.1	uncharacterized LOC111407296%2C transcript variant X2
2320	gene-LOC11140729	2.0430049096	10.239740846433	-1.007783993	0.981399776	-2.32541462703	protein_codin	XM_023036707.1	uncharacterized LOC111407298
2321	gene-LOC11140730	2.66845099056667	0.7067508191	0.6897099277	0.951591577	1.916728959470	protein_codin	XM_023036713.1	COP9 signalosome complex subunit 2-like
2322	gene-LOC11140730	10.4236483859	4.5321157979	0.8574620850	0.961143871	1.201603696007	protein_codin	XM_023036718.1	protein G1-like1
2323	gene-LOC11140730	0.5	3.6848927691333	-1.071787357	0.977187220	-2.88162263731	protein_codin	XM_023036725.1	CBL-interacting serine/threonine-protein kinase 3-like
2324	gene-LOC11140732	4.64037117286667	1.0930574109666	0.8079210187	0.959807910	2.085871029820	protein_codin	XR_002705087.1	glucomannan 4-beta-mannosyltransferase 1-like%2C transcript variant X4
2325	gene-LOC11140733	21.7559298067667	8.5911709679666	1.0415639669	0.967726737	1.340481988456	protein_codin	XM_023036748.1	ADP%2CATP carrier protein 1%2C mitochondrial-like%2C transcript variant X1
2326	gene-LOC11140733	2.3381168952	0.5	0.7562683526	0.954277178	2.225347059856	protein_codin	XM_023036752.1	histone acetyltransferase HAC1-like%2C transcript variant X1
2327	gene-LOC11140734	0.938432989266667	4.6128951291666	-0.851197486	0.954379428	-2.29734685890	protein_codin	XR_002705092.1	myosin-11-like%2C transcript variant X3
2328	gene-LOC11140738	5.1682682124	1.0801683049	0.8112566708	0.959959676	2.258424820668	protein_codin	XM_023036805.1	uncharacterized protein sll0103-like
2329	gene-LOC11140738	5.54802065676667	1.55316804	0.7508003393	0.953878998	1.836759233311	protein_codin	XM_023036810.1	histone H2B.3-like
2330	gene-LOC11140741	0.5	2.3000030115333	-0.746106397	0.952051421	-2.20163575017	protein_codin	XM_023036868.1	protein YIPF1 homolog%2C transcript variant X2
2331	gene-LOC11140742	5.90279673833333	0.9729997351	0.9457148623	0.974803486	2.600887346371	protein_codin	XR_002705107.1	pyridine nucleotide-disulfide oxidoreductase domain-containing protein 2-like%2C transcript variant X3
2332	gene-LOC11140743	211.343093222733	113.57440495966	2.2417892078	0.999999999	0.895949218455	protein_codin	XM_023036897.1	uncharacterized LOC111407433
2333	gene-LOC11140743	55.7169437484333	37.1903412109	0.8494060492	0.960830868	0.583188139367	protein_codin	XM_023036899.1	homeobox protein knotted-1-like 3%2C transcript variant X1
2334	gene-LOC11140745	2.37235545393333	7.2524009602333	-0.802741647	0.958150015	-1.61213850146	protein_codin	XM_023036932.1	diacylglycerol O-acyltransferase 3%2C cytosolic
2335	gene-LOC11140745	2.7507327191	0.8863065918666	0.7143026877	0.953233424	1.633938215454	protein_codin	XM_023036942.1	uncharacterized LOC111407459%2C transcript variant X2
2336	gene-LOC11140746	15.071065773	8.2103498398666	0.7041340452	0.952886223	0.876265841775	protein_codin	XM_023036952.1	probable protein phosphatase 2C 50%2C transcript variant X4
2337	gene-LOC11140747	6.11947367686667	1.6867258483	0.8383699696	0.960535367	1.859182070584	protein_codin	XM_023036973.1	IQ domain-containing protein IQM1-like
2338	gene-LOC11140747	3.05973683843333	0.5	1.1098043959	0.974679482	2.613407575070	protein_codin	XR_002705115.1	protein CLP1 homolog%2C transcript variant X2
2339	gene-LOC11140750	5.82337814023333	2.1861148219	0.7564769725	0.954295639	1.413487124950	protein_codin	XM_023037026.1	asparagine--tRNA ligase%2C chloroplastic/mitochondrial-like
2340	gene-LOC11140752	13.5156566810333	2.2456129188	1.3353067882	0.969823147	2.589450435565	protein_codin	XM_023037063.1	thaumatin-like protein
2341	gene-LOC11140753	5.80401526623333	1.6859200404333	0.8093184663	0.959875898	1.783515200138	protein_codin	XM_023037095.1	uncharacterized LOC111407539%2C transcript variant X4
2342	gene-LOC11140756	0.5	4.5321766906	-1.311437767	0.970826132	-3.18020410689	protein_codin	XM_023037139.1	GTP-binding nuclear protein Ran-A1
2343	gene-LOC11140760	10.7261982138667	4.1051749199666	0.9045782028	0.965661498	1.385623217759	protein_codin	XM_023037204.1	aluminum-activated malate transporter 9-like
2344	gene-LOC11140761	1.24098281723333	5.2782058543	-0.782008283	0.955515455	-2.08856447812	protein_codin	XM_023037213.1	metal tolerance protein 1-like%2C transcript variant X3
2345	gene-LOC11140765	15.8379532781667	1.9325602304666	1.3724164887	0.953760630	3.034800626302	protein_codin	XM_023037265.1	luminal-binding protein 5
2346	gene-LOC11140765	5.81046955756667	2.1861148219	0.8414784479	0.960604229	1.410285577822	protein_codin	XM_023037272.1	probable serine/threonine-protein kinase WNK10%2C transcript variant X1
2347	gene-LOC11140766	5.4252463729	1.3133083625333	0.7809811925	0.957199969	2.046482956833	protein_codin	XM_023037284.1	GTPase-activating protein gyp7-like%2C transcript variant X2
2348	gene-LOC11140766	2.1649959372	0.5	0.7870403322	0.957970697	2.114364317613	lncRNA	XR_002705149.1	uncharacterized LOC111407666
2349	gene-LOC11140767	29.9199838671667	20.715251438366	0.7277324193	0.953312985	0.530416065703	protein_codin	XM_023037306.1	uncharacterized LOC111407678
2350	gene-LOC11140768	1.95614846913333	0.5	0.6824614660	0.950524364	1.968015873129	protein_codin	XM_023037324.1	uncharacterized LOC111407689%2C transcript variant X2

2351	gene-LOC11140769	0.629428869966667	3.0656409012	-0.823559117	0.959496122	-2.28407345722	protein_codin	XM_023037333.1	DUF724 domain-containing protein 6-like
2352	gene-LOC11140771	142.899196066167	95.980051215766	1.1619021802	0.983150510	0.574191312025	lncRNA	XR_002705161.1	uncharacterized LOC111407711
2353	gene-LOC11140771	8.1727795593	1.2333348391333	1.1574749822	0.983118854	2.728262290442	protein_codin	XM_023037357.1	calcium-transporting ATPase 4%2C endoplasmic reticulum-type-like%2C transcript variant X1
2354	gene-LOC11140773	2.86379795306667	0.5	0.9235096838	0.969896025	2.517929711041	protein_codin	XM_023037388.1	nudix hydrolase 16%2C mitochondrial-like%2C transcript variant X2
2355	gene-LOC11140774	1.53309556486667	4.9055332836666	-0.753120994	0.952872895	-1.67796235168	lncRNA	XR_002705169.1	uncharacterized LOC111407745%2C transcript variant X2
2356	gene-LOC11140774	0.5	2.5127285353666	-0.801158618	0.957947684	-2.32925481692	protein_codin	XR_002705171.1	protein ELF4-LIKE 3-like%2C transcript variant X3
2357	gene-LOC11140775	4.30417470906667	9.9702686989666	-0.831670081	0.958896635	-1.21189574685	protein_codin	XM_023037416.1	probable pyridoxal 5'-phosphate synthase subunit PDX1
2358	gene-LOC11140775	5.58088403633333	1.0801683049	1.0075015710	0.971092889	2.369237547937	protein_codin	XM_023037417.1	cysteine-rich repeat secretory protein 3-like
2359	gene-LOC11140778	29.5693585726667	11.835561834	0.8439945781	0.960667142	1.320974756010	protein_codin	XM_023037456.1	zinc protease PQQL-like%2C transcript variant X5
2360	gene-LOC11140780	3.15343919836667	0.5	0.9662992048	0.976157527	2.656926116795	protein_codin	XM_023037486.1	uncharacterized LOC111407807%2C transcript variant X2
2361	gene-LOC11140781	2.51469290656667	0.5	0.9399167264	0.973746235	2.330382229365	protein_codin	XR_002705206.1	transcription initiation factor TFIID subunit 15b-like%2C transcript variant X7
2362	gene-LOC11140782	9.6812903895	18.0750006921	-0.797090609	0.957410198	-0.90072444459	protein_codin	XM_023037509.1	mitochondrial-processing peptidase subunit alpha-like%2C transcript variant X2
2363	gene-LOC11140783	5.0801241154	0.5	0.9592102058	0.976139759	3.344863744730	protein_codin	XM_023037523.1	receptor-like protein kinase 5
2364	gene-LOC11140784	1.68039935753333	5.9194838718	-0.747689662	0.952254903	-1.81666725135	protein_codin	XM_023037529.1	hexokinase-1-like
2365	gene-LOC11140788	7.5925135177	1.8458061945333	0.8775718563	0.962309227	2.040326490740	protein_codin	XM_023037577.1	golgin candidate 1-like%2C transcript variant X1
2366	gene-LOC11140788	3.22738705613333	8.7172033485333	-0.743600562	0.951708279	-1.43349875543	protein_codin	XM_023037585.1	dnaJ homolog subfamily C GRV2%2C transcript variant X1
2367	gene-LOC11140788	4.6445542566	0.9729997351	0.7564162134	0.954290259	2.255028828747	protein_codin	XM_023037587.1	uncharacterized LOC111407889
2368	gene-LOC11140789	0.5	2.4055599655666	-0.761230258	0.953631963	-2.26637276323	protein_codin	XM_023037589.1	ras-related protein Rab7
2369	gene-LOC11140790	40.6878032562	24.794842927666	0.7886471386	0.958160155	0.714556304997	protein_codin	XM_023037615.1	aldehyde oxidase GLOX1-like
2370	gene-LOC11140791	25.0558895415	13.1158831236	1.2775939280	0.983492581	0.933834806695	protein_codin	XM_023037633.1	probable 2-oxoglutarate-dependent dioxygenase ANS
2371	gene-LOC11140793	5.23674532986667	0.5	1.2258296030	0.984356797	3.388670446247	protein_codin	XM_023037658.1	EIN3-binding F-box protein 1-like%2C transcript variant X2
2372	gene-LOC11140793	4.82887221586667	1.0930574109666	0.8911754682	0.963619111	2.143317108583	protein_codin	XM_023037659.1	uncharacterized LOC111407934%2C transcript variant X1
2373	gene-LOC11140794	57.8289464640667	100.10436310346	-1.542148876	0.999999988	-0.79164113194	protein_codin	XM_023037671.1	NADH-cytochrome b5 reductase-like protein
2374	gene-LOC11140795	0.938432989266667	7.6233401297333	-1.172678959	0.983723565	-3.02209761086	protein_codin	XM_023037683.1	probable sugar phosphate/phosphate translocator At5g25400
2375	gene-LOC11140796	0.5	2.5127285353666	-0.801158618	0.957947684	-2.32925481692	protein_codin	XM_023037686.1	uncharacterized protein At2g34160-like
2376	gene-LOC11140798	3.17339399523333	0.5	0.9806593731	0.974964276	2.666026651316	protein_codin	XM_023037725.1	uncharacterized LOC111407987
2377	gene-LOC11140798	27.119832799	43.615127868333	-1.059537565	0.979942360	-0.68548033539	protein_codin	XM_023037728.1	LEAF RUST 10 DISEASE-RESISTANCE LOCUS RECEPTOR-LIKE PROTEIN KINASE-like 2.1%2C transcript variant X2
2378	gene-LOC11140799	33.6748763346333	7.5986233996666	2.0517649775	1	2.147862665334	lncRNA	XR_002705265.1	uncharacterized LOC111407993%2C transcript variant X3
2379	gene-LOC11140800	3.0393904134	0.5	0.8781851097	0.962355789	2.603782002698	protein_codin	XM_023037746.1	transcription repressor OFP1-like
2380	gene-LOC11140800	2.5483395424	0.5	0.7722236578	0.956042524	2.349557516016	protein_codin	XM_023037747.1	U-box domain-containing protein 16-like
2381	gene-LOC11140802	7.8188442803	1.605946517	1.0136158405	0.970306384	2.283531528089	protein_codin	XM_023037771.1	probable L-type lectin-domain containing receptor kinase VI.1
2382	gene-LOC11140802	3.7655850685	0.5	1.1966649729	0.982451593	2.912874037499	protein_codin	XM_023037776.1	ammonium transporter 2
2383	gene-LOC11140803	1.95614846913333	0.5	0.6824614660	0.950524364	1.968015873129	protein_codin	XM_023037777.1	receptor-like protein 12
2384	gene-LOC11140803	2.7537319571	0.5	0.6975232012	0.952426558	2.461388137184	protein_codin	XM_023037779.1	abscisic acid receptor PYL4-like
2385	gene-LOC11140804	1.96260276046667	0.5	0.7250116886	0.953309787	1.972768194454	protein_codin	XM_023037801.1	uncharacterized LOC111408043
2386	gene-LOC11140804	20.1078894284667	9.4896826434333	0.9461519520	0.974873055	1.083329914750	protein_codin	XM_023037812.1	heavy metal-associated isoprenylated plant protein 6-like
2387	gene-LOC11140805	12.6551543385	0.5	2.6627132518	1	4.661653197123	protein_codin	XM_023037823.1	tonoplast dicarboxylate transporter
2388	gene-LOC11140806	0.5	1.7863080976333	-0.789003132	0.956345087	-1.83698093409	protein_codin	XR_002705277.1	sucrose nonfermenting 4-like protein%2C transcript variant X5
2389	gene-LOC11140807	0.5	5.2781449615666	-1.555680824	0.999999998	-3.40003097409	lncRNA	XR_002705279.1	uncharacterized LOC111408079
2390	gene-LOC11140808	2.1317409295	0.5	0.7780517328	0.956812545	2.092032118005	protein_codin	XM_023037869.1	probable protein phosphatase 2C 5%2C transcript variant X3
2391	gene-LOC11140809	8.66195085083333	22.0994571735	-1.117133959	0.975065253	-1.35124704181	protein_codin	XM_023037876.1	protein DEHYDRATION-INDUCED 19 homolog 3-like%2C transcript variant X2
2392	gene-LOC11140812	1.96905705183333	7.7033745458333	-0.859457332	0.951519125	-1.96798565954	protein_codin	XM_023037918.1	serine/threonine-protein kinase SAPK2-like
2393	gene-LOC11140812	2.87084416726667	8.9316013808	-0.851666221	0.954226811	-1.63744384330	protein_codin	XM_023037926.1	heat shock factor protein HSF8-like
2394	gene-LOC11140815	3.15343919836667	0.6531665342333	0.8191240094	0.960204235	2.271403337091	protein_codin	XM_023037957.1	uncharacterized LOC111408154%2C transcript variant X1
2395	gene-LOC11140815	47.7407988082667	35.2184417431	0.9389639635	0.973547674	0.438891628228	protein_codin	XM_023037958.1	uncharacterized LOC111408156
2396	gene-LOC11140817	3.06619112976667	0.5	0.9928071491	0.973238823	2.616447629603	protein_codin	XM_023037985.1	mitochondrial carnitine/acylcarnitine carrier-like protein%2C transcript variant X2
2397	gene-LOC11140828	8.8485723144	1.1329467819	1.1723062122	0.982926988	2.965364605477	protein_codin	XM_023038065.1	probable 1-deoxy-D-xylulose-5-phosphate synthase 2%2C chloroplastic
2398	gene-LOC11140832	1.85599181786667	0.5	0.9346285653	0.972602995	1.892190350363	protein_codin	XM_023038094.1	alpha-glucosidase-like
2399	gene-LOC11140834	3.32715207923333	0.6531665342333	0.8387820735	0.960544290	2.348765031494	protein_codin	XM_023038110.1	SAC3 family protein A-like

2400	gene-LOC11140841	12.8569555923333	2.8325008435666	1.0789879883	0.9680452088	2.182400775502	protein_codin	XM_023038175.1	protein transport protein SEC16B homolog
2401	gene-LOC11140841	2.96813768803333	0.5	0.7266124400	0.9533116603	2.569558018250	protein_codin	XM_023038180.1	uncharacterized LOC111408418
2402	gene-LOC11140842	1.95614846913333	0.5	0.6824614660	0.9505243641	1.968015873129	protein_codin	XM_023038184.1	serine/threonine-protein kinase CTR1-like
2403	gene-LOC11140843	2.5612481251	0.6531665342333	0.7162855073	0.9532631976	1.971324243442	protein_codin	XM_023038200.1	uncharacterized mitochondrial protein AtMg00820-like
2404	gene-LOC11140850	8.60736586706667	1.4928641352333	1.3554651984	0.9615382058	2.527488921181	protein_codin	XM_023038255.1	uncharacterized LOC111408507
2405	gene-LOC11140852	12.1294732806667	5.4660928626333	0.7587601729	0.9544998211	1.149935028236	protein_codin	XM_023038276.1	leucine-rich repeat extensin-like protein 4
2406	gene-LOC11140860	487.944183613833	565.77959497793	-1.2261756451	0.9844063743	-0.213524020407	protein_codin	XM_023038350.1	pumilio homolog 12-like
2407	gene-LOC11140862	66.3959395950667	89.439292994733	-0.7634623600	0.9538209123	-0.429813766824	protein_codin	XM_023038363.1	putative ATP synthase protein YMF19
2408	gene-LOC11140863	1.85599181786667	0.5	0.9346285653	0.9726029951	1.892190350363	protein_codin	XM_023038376.1	ethylene-responsive transcription factor ERF069-like
2409	gene-LOC11140867	86.4625119539	66.7513971735	0.7855145118	0.9577814961	0.373276716603	protein_codin	XM_023038405.1	ABC transporter F family member 5
2410	gene-LOC11140867	3.93413131463333	0.6531665342333	0.9463095997	0.9748959234	2.590522333430	protein_codin	XM_023038407.1	programmed cell death protein 2-like%2C transcript variant X2
2411	gene-LOC11140871	646.928330259767	797.11236797386	-1.8791491875	0.9891669691	-0.301177220960	lncRNA	XR_002705343.1	uncharacterized LOC111408713
2412	gene-LOC11140873	7.5130949196	1.7131150868	0.9235810338	0.9699136609	2.132785254526	protein_codin	XM_023038490.1	transcriptional corepressor SEUSS-like%2C transcript variant X2
2413	gene-LOC11140875	12.0574925249333	6.0785642180333	0.9573147913	0.9760644866	0.988127417203	protein_codin	XM_023038514.1	trihelix transcription factor GT-2-like
2414	gene-LOC11140876	69.1159373937333	101.30793410106	-1.2549596393	0.9793086578	-0.551656841562	protein_codin	XM_023038525.1	abscisic stress-ripening protein 2-like%2C transcript variant X1
2415	gene-LOC11140876	8.2909239054	2.9396694133333	0.7610309070	0.9547233709	1.495878955598	protein_codin	XM_023038528.1	cellulose synthase A catalytic subunit 2 [UDP-forming]-like
2416	gene-LOC11140878	0.629428869966667	3.3123419839666	-0.7876402761	0.9561741483	-2.395736374855	protein_codin	XM_023038556.1	cyclic nucleotide-gated ion channel 1-like
2417	gene-LOC11140879	2.60194097513333	0.5	0.7922052050	0.9585612544	2.379588234981	protein_codin	XR_002705364.1	bZIP transcription factor 46-like%2C transcript variant X9
2418	gene-LOC11140880	15.3071055855333	8.9044063344	0.7065060474	0.9530016656	0.781610176261	protein_codin	XM_023038573.1	polyadenylate-binding protein 2-like
2419	gene-LOC11140883	171.760075869633	295.98955375183	-1.9048254957	0.9979594805	-0.785151526683	protein_codin	XM_023038611.1	exocyst complex component SEC3A-like
2420	gene-LOC11140883	5.32548134976667	12.2371019595	-0.9934788570	0.9748365753	-1.200278097547	protein_codin	XM_023038635.1	probable galacturonosyltransferase-like 1
2421	gene-LOC11140884	35.9493463449667	23.8354042177	0.9142606651	0.9676717445	0.592859446996	protein_codin	XM_023038647.1	cyclin-D-binding Myb-like transcription factor 1%2C transcript variant X1
2422	gene-LOC11140888	6.88537763103333	17.2745814357	-1.2179500804	0.9850960000	-1.327043067661	protein_codin	XM_023038715.1	acetyl-CoA carboxylase 1-like%2C transcript variant X1
2423	gene-LOC11140889	0.6358831613	3.1991987094666	-1.0350171018	0.9845080910	-2.330876993568	protein_codin	XM_023038735.1	60S ribosomal protein L30-like
2424	gene-LOC11140890	3.9193431525	0.9729997351	0.7208926784	0.9532988322	2.010100574232	protein_codin	XM_023038753.1	V-type proton ATPase 16 kDa proteolipid subunit
2425	gene-LOC11140890	1.55989628123333	4.7992314144666	-0.7992754261	0.9577032068	-1.621353273826	protein_codin	XM_023038757.1	peptidyl-prolyl cis-trans isomerase Pin1-like
2426	gene-LOC11140890	3.96738632233333	0.5	1.1372114645	0.9813322349	2.988188886058	protein_codin	XM_023038754.1	kinesin-like protein KIN-7G
2427	gene-LOC11140891	2.08916849996667	0.5	0.7050067510	0.9529356131	2.062928856341	protein_codin	XM_023038760.1	protein SHI RELATED SEQUENCE 1-like
2428	gene-LOC11140893	3.02648183073333	0.6531665342333	0.8465419593	0.9607395859	2.212118910253	protein_codin	XM_023038785.1	uncharacterized LOC111408937%2C transcript variant X1
2429	gene-LOC11140895	3.9044674678	0.5	1.0594868120	0.9670460820	2.965125791912	protein_codin	XM_023038805.1	isoflavone reductase-like protein
2430	gene-LOC11140895	8.62125800073333	3.2850860448666	0.8394070382	0.9605578719	1.391967241578	protein_codin	XM_023038806.1	isoflavone reductase-like protein
2431	gene-LOC11140895	5.61512259503333	1.819416956	0.7059017862	0.9529757580	1.625841317872	protein_codin	XM_023038810.1	cyclin-T1-3-like%2C transcript variant X2
2432	gene-LOC11140898	5.96195643396667	0.5	1.1638439691	0.9831348653	3.575785833106	protein_codin	XM_023038863.1	mannose-6-phosphate isomerase 1-like
2433	gene-LOC11140902	3.8320950839	10.450732969133	-0.7728557775	0.9546137430	-1.447398866717	protein_codin	XM_023038916.1	probable isoaspartyl peptidase/L-asparaginase 2
2434	gene-LOC11140902	2.51469290656667	0.5	0.9399167264	0.9737462359	2.330382229365	protein_codin	XM_023038920.1	F-box protein At1g47056-like
2435	gene-LOC11140903	0.5	2.4055599655666	-0.7612302589	0.9536319638	-2.266372763231	protein_codin	XM_023038930.1	uncharacterized LOC111409031
2436	gene-LOC11140904	5.251533492	2.1861148219	0.7155393456	0.9532519384	1.264369585337	protein_codin	XM_023038942.1	nucleolar GTP-binding protein 1
2437	gene-LOC11140905	69.9955537305667	52.5468510345	0.7494766445	0.9538021395	0.413658971192	lncRNA	XR_002705415.1	uncharacterized LOC111409054
2438	gene-LOC11140906	2.03556706723333	0.5	0.7088504665	0.9530935972	2.025430755730	protein_codin	XM_023038957.1	uncharacterized LOC111409060%2C transcript variant X2
2439	gene-LOC11140907	56.5673617011333	76.8642093527	-0.7746701076	0.9547780796	-0.442342100258	protein_codin	XM_023039003.1	serine hydroxymethyltransferase%2C mitochondrial%2C transcript variant X4
2440	gene-LOC11140908	6.69875616746667	2.3000030115333	0.7546593345	0.9541502280	1.542257489030	protein_codin	XM_023038990.1	alpha-amylase
2441	gene-LOC11140909	8.24248910743333	0.5	1.6552513046	0.9757989788	4.043080075354	protein_codin	XM_023039018.1	oxysterol-binding protein-related protein 1C-like%2C transcript variant X1
2442	gene-LOC11140913	2.44818289113333	0.5	0.9691321958	0.9760356773	2.291711338371	protein_codin	XM_023039090.1	amino acid permease 3-like
2443	gene-LOC11140914	0.5	2.8852793205666	-0.8592127622	0.9516079860	-2.528710991276	lncRNA	XR_002705435.1	uncharacterized LOC111409140%2C transcript variant X1
2444	gene-LOC11140914	1.8892468256	0.5	0.7326001315	0.9533302059	1.917811198712	protein_codin	XM_023039109.1	probable gamma-secretase subunit PEN-2
2445	gene-LOC11140914	40.5528713492667	22.982206484233	1.2474129004	0.9857102383	0.819286752759	protein_codin	XM_023039113.1	photosystem I reaction center subunit N%2C chloroplastic-like
2446	gene-LOC11140917	9.974273916	1.8458061945333	0.9449285624	0.9746697753	2.433960741971	protein_codin	XM_023039149.1	AAA-ATPase ASD%2C mitochondrial-like
2447	gene-LOC11140917	16.8185670688	6.2912288491333	1.0344239347	0.9682364597	1.418641046572	protein_codin	XM_023039151.1	AAA-ATPase ASD%2C mitochondrial-like
2448	gene-LOC11140917	0.5	2.8325008435666	-0.8411716718	0.9572015853	-2.502076385704	protein_codin	XM_023039156.1	uncharacterized LOC111409175%2C transcript variant X2

2449	gene-LOC11140919	1.96905705183333	8.1064045015666	-0.859594752(	0.951469095:	-2.04155725347'	protein_codin	XM_023039180.1	UPF0496 protein 1-like
2450	gene-LOC11140919	6.58282780306667	1.8926708595333	0.8546557659	0.961025702	1.798283915132	lncRNA	XR_002705458.1	uncharacterized LOC111409199
2451	gene-LOC11140920	46.3750170588	102.76870745	-1.867269018	0.980479991	-1.14798132054	protein_codin	XM_023039201.1	sigma intracellular receptor 2-like
2452	gene-LOC11140920	2.66845099056667	0.5	0.8904698014	0.963535251	2.416002514674	protein_codin	XR_002705462.1	nudix hydrolase 20%2C chloroplastic-like%2C transcript variant X2
2453	gene-LOC11140920	0.5	3.732563242	-1.267130059	0.976216522	-2.90016670460	protein_codin	XM_023039204.1	probable polygalacturonase
2454	gene-LOC11140921	3.1792563637	12.2099678058	-1.061946979	0.979376116	-1.94129813638	protein_codin	XM_023039213.1	glucose-6-phosphate/phosphate translocator 2%2C chloroplastic-like
2455	gene-LOC11140921	6.48853352026667	2.1393110495666	0.7030484803	0.952825255	1.600746188773	protein_codin	XM_023039227.1	V-type proton ATPase subunit d2
2456	gene-LOC11140922	1.36395739586667	7.9448458390333	-1.448844448	0.999513742	-2.54222064443	protein_codin	XM_023039234.1	alpha-mannosidase 2%2C transcript variant X2
2457	gene-LOC11140923	2.69426815593333	0.5	0.8963939323	0.964311812	2.429893446933	protein_codin	XM_023039243.1	60S ribosomal protein L7a-1-like
2458	gene-LOC11140924	4.3571842189	0.7331400576333	0.9511410606	0.975545848	2.571235371324	protein_codin	XM_023039265.1	uncharacterized LOC111409249
2459	gene-LOC11140926	2.88316082706667	0.5	0.7432816620	0.953524644	2.527651314786	protein_codin	XM_023039279.1	wall-associated receptor kinase 2-like
2460	gene-LOC11140930	8.0901062026	2.1597255834	0.9953991274	0.972846624	1.905310627756	protein_codin	XR_002705486.1	SAGA-associated factor 29-like%2C transcript variant X7
2461	gene-LOC11140931	5.1850234766	0.9729997351	0.9981648847	0.972430555	2.413839203902	protein_codin	XM_023039344.1	pescadillo homolog
2462	gene-LOC11140933	3.64547362036667	0.7331400576333	0.9336859547	0.972380530	2.313945522418	protein_codin	XM_023039377.1	photosystem I assembly factor PSA3%2C chloroplastic
2463	gene-LOC11140934	26.2628893206667	7.5179049611	1.5457509048	0.963901223	1.804623061016	protein_codin	XR_002705491.1	protein NLP5-like%2C transcript variant X4
2464	gene-LOC11140934	0.5	2.4599500583333	-0.805478126	0.958483519	-2.29862902640	protein_codin	XM_023039407.1	beta-amylase-like
2465	gene-LOC11140935	2.25224400576667	0.5	0.7304862915	0.953319521	2.171363135961	protein_codin	XM_023039413.1	psbP domain-containing protein 3%2C chloroplastic
2466	gene-LOC11140938	0.5	2.4599500583333	-0.805478126	0.958483519	-2.29862902640	protein_codin	XM_023039437.1	pyridoxal kinase
2467	gene-LOC11140938	0.938432989266667	5.5987839703333	-1.051357719	0.981829447	-2.57678787933	protein_codin	XM_023039442.1	protein COFACTOR ASSEMBLY OF COMPLEX C SUBUNIT B CCB1%2C chloroplastic
2468	gene-LOC11140939	0.5	3.7853417190666	-1.078716400	0.975909185	-2.92042354437	protein_codin	XM_023039443.1	uncharacterized LOC111409390
2469	gene-LOC11140939	0.629428869966667	2.1861148219	-0.782603758	0.955581572	-1.79625392081	protein_codin	XM_023039447.1	subtilisin-like protease SBT1.9
2470	gene-LOC11140940	14.7749702363667	24.5813724887	-1.000622544	0.978538109	-0.73441024596	protein_codin	XM_023039460.1	uncharacterized LOC111409407
2471	gene-LOC11140948	0.938432989266667	5.9985906946333	-1.160203740	0.981838266	-2.67629795936	protein_codin	XM_023039531.1	ACT domain-containing protein ACR1-like
2472	gene-LOC11140951	164.555969060933	80.423689051233	1.8764070474	0.999999307	1.032885939872	protein_codin	XM_023039557.1	heptahelical transmembrane protein 4-like%2C transcript variant X1
2473	gene-LOC11140957	3.02361870026667	0.5	0.9494124709	0.975334379	2.596276216966	protein_codin	XM_023039610.1	salicylate carboxymethyltransferase-like
2474	gene-LOC11140960	3.6712907857	0.5	1.0300646715	0.968597662	2.876287388061	protein_codin	XM_023039633.1	uncharacterized LOC111409605%2C transcript variant X1
2475	gene-LOC11140964	3.05628178506667	0.5	0.7379978069	0.953392065	2.611777564046	protein_codin	XM_023039670.1	protein DMP7-like
2476	gene-LOC11140968	26.7736394679	44.546626616766	-0.990309472	0.972970081	-0.73450292019	protein_codin	XM_023039701.1	LOB domain-containing protein 37-like
2477	gene-LOC11140971	3.10688397983333	7.1452323904333	-0.744364701	0.951815415	-1.20151256607	protein_codin	XM_023039722.1	zinc finger CCCH domain-containing protein 7-like
2478	gene-LOC11140974	0.629428869966667	5.9450673024333	-1.485982536	0.999983033	-3.23957788515	protein_codin	XM_023039753.1	protein ABIL1-like
2479	gene-LOC11140977	0.5	3.0452263673666	-0.959484033	0.951604986	-2.60654947507	protein_codin	XR_002705528.1	probable translation initiation factor eIF-2B subunit delta%2C transcript variant X2
2480	gene-LOC11140978	6.1730751096	1.1329467819	1.1131265593	0.975612993	2.445909250364	protein_codin	XM_023039816.1	mRNA turnover protein 4 homolog
2481	gene-LOC11140978	4.29126612636667	1.2061397927333	0.7520517603	0.953961878	1.831006247132	protein_codin	XM_023039887.1	protein FLX-like 2%2C transcript variant X10
2482	gene-LOC11140980	0.5	3.9452278731333	-1.344785951	0.975241642	-2.98010863153	protein_codin	XM_023039852.1	GTPase Der%2C transcript variant X2
2483	gene-LOC11140980	4.31708329173333	0.9729997351	0.9036037177	0.965480283	2.149545610389	protein_codin	XM_023039857.1	2-C-methyl-D-erythritol 4-phosphate cytidylyltransferase%2C chloroplastic-like%2C transcript variant X1
2484	gene-LOC11140980	8.62027444973333	2.1861148219	1.0925538818	0.970291422	1.979364624079	protein_codin	XM_023039861.1	auxin response factor 1%2C transcript variant X1
2485	gene-LOC11140981	6.17952940093333	17.0897958734	-0.980003096	0.966166592	-1.46756628537	protein_codin	XM_023039888.1	ras-related protein RABD1-like
2486	gene-LOC11140982	2.57415670776667	0.5	0.7724889366	0.956076955	2.364099883642	protein_codin	XM_023039896.1	leucine-rich repeat receptor-like serine/threonine-protein kinase BAM3
2487	gene-LOC11140982	2.7507327191	0.5	1.1130930723	0.975603615	2.459815963877	protein_codin	XM_023039903.1	nifU-like protein 2%2C chloroplastic
2488	gene-LOC11140983	20.9008982045667	13.1150773157	0.6876532437	0.951317833	0.672338630091	protein_codin	XM_023039915.1	serine/threonine-protein kinase SRK2A-like
2489	gene-LOC11140983	3.40297951646667	0.6531665342333	0.9938152927	0.973085321	2.381275687619	protein_codin	XM_023039918.1	E3 ubiquitin-protein ligase RNF170-like%2C transcript variant X3
2490	gene-LOC11140984	2.66845099056667	0.5	0.8904698014	0.963535251	2.416002514674	protein_codin	XM_023039932.1	shaggy-related protein kinase NtK-1-like
2491	gene-LOC11140987	1.51771547986667	7.0380638207	-0.972823358	0.961020303	-2.21327723665	protein_codin	XM_023039967.1	T-complex protein 1 subunit alpha-like%2C transcript variant X1
2492	gene-LOC11140988	6.239585125	0.9729997351	1.1576336591	0.983122133	2.680938788986	protein_codin	XR_002705567.1	transcription factor UNE10-like%2C transcript variant X2
2493	gene-LOC11140988	1.85599181786667	0.5	0.9346285653	0.972602995	1.892190350363	protein_codin	XM_023039983.1	scarecrow-like protein 6%2C transcript variant X1
2494	gene-LOC11140989	4.50956712373333	0.5	1.1050198764	0.973355171	3.172988954896	protein_codin	XM_023039989.1	nicotianamine synthase-like
2495	gene-LOC11140993	0.5	2.1928344418	-0.754119384	0.952977800	-2.13279689317	protein_codin	XM_023040056.1	uncharacterized LOC111409932
2496	gene-LOC11140993	5.27187991703333	11.304736510433	-0.740762598	0.951288920	-1.10053795220	protein_codin	XM_023040065.1	uncharacterized LOC111409939
2497	gene-LOC11140996	11.0565323092333	3.7936729546333	0.8521104842	0.960926479	1.543231762869	protein_codin	XM_023040096.1	eukaryotic translation initiation factor 1A-like

2498	gene-LOC11140997	21.5412199702333	9.2258511510333	1.0493275255	0.967307404	1.223346036701	protein_codin	XM_023040114.1	protein COBRA-like
2499	gene-LOC11140997	2.46109147383333	0.7067508191	0.7058878411	0.952975161	1.800024725669	protein_codin	XM_023040117.1	dihydrolipoyl dehydrogenase 2%2C chloroplastic-like
2500	gene-LOC11140997	3.11333827116667	0.8863065918666	0.8197714542	0.960218394	1.812584588943	protein_codin	XM_023040119.1	uncharacterized LOC111409976
2501	gene-LOC11140998	104.726638701733	150.62677976403	-1.473613487	0.999941279	-0.52434982908	lncRNA	XR_002705591.1	uncharacterized LOC111409981
2502	gene-LOC11140998	0.6358831613	2.9396694133333	-0.807936230	0.958758582	-2.20882031217	protein_codin	XM_023040142.1	putative methylesterase 11%2C chloroplastic%2C transcript variant X2
2503	gene-LOC11140999	0.992034422	3.9452278731333	-0.794538278	0.957068036	-1.99164654573	protein_codin	XM_023040144.1	uncharacterized LOC111409995%2C transcript variant X3
2504	gene-LOC11141001	4.06168060513333	13.569213240033	-1.311734412	0.970836719	-1.74018837267	protein_codin	XM_023040166.1	F-box/WD-40 repeat-containing protein At3g52030
2505	gene-LOC11141004	2.0762599173	0.5	0.6996756982	0.952603165	2.053987059267	protein_codin	XM_023040207.1	cytochrome c1-2%2C heme protein%2C mitochondrial%2C transcript variant X2
2506	gene-LOC11141004	2.03556706723333	0.5	0.7088504665	0.953093597	2.025430755730	protein_codin	XM_023040206.1	long chain acyl-CoA synthetase 6%2C peroxisomal-like
2507	gene-LOC11141004	268.628050540233	326.3505276265	-1.068531066	0.977873830	-0.28081241078	lncRNA	XR_002705605.1	uncharacterized LOC111410049
2508	gene-LOC11141005	2.69426815593333	0.5	0.8963939323	0.964311812	2.429893446933	lncRNA	XR_002705607.1	uncharacterized LOC111410057%2C transcript variant X2
2509	gene-LOC11141008	5.08298724586667	1.1329467819	0.7772862194	0.956710353	2.165596516015	protein_codin	XM_023040260.1	purple acid phosphatase 2-like
2510	gene-LOC11141008	1.95614846913333	0.5	0.6824614660	0.950524364	1.968015873129	protein_codin	XM_023040267.1	kinesin-like protein KIN-7D%2C mitochondrial%2C transcript variant X2
2511	gene-LOC11141009	2.7636413018	6.95815119	-0.790196750	0.956497157	-1.33213364908	protein_codin	XM_023040296.1	protein XAP5 CIRCADIAN TIMEKEEPER
2512	gene-LOC11141009	2.08916849996667	8.3182633248333	-1.090249058	0.974515089	-1.99335349964	protein_codin	XM_023040691.1	protein MODIFIER OF SNC1 1-like%2C transcript variant X6
2513	gene-LOC11141010	0.5	2.1861148219	-1.035006510	0.984509101	-2.12836917806	protein_codin	XM_023040300.1	putative ER lumen protein-retaining receptor C28H8.4
2514	gene-LOC11141011	2.08916849996667	0.5	0.7050067510	0.952935613	2.062928856341	protein_codin	XM_023040316.1	eukaryotic translation initiation factor 4B3-like
2515	gene-LOC11141011	5.56797545366667	2.0865325725666	0.7470474611	0.953675082	1.416045408615	protein_codin	XM_023040324.1	L-ascorbate peroxidase 3-like
2516	gene-LOC11141012	5.7624263877	16.8475187723	-1.009307207	0.981906778	-1.54778781231	protein_codin	XM_023040325.1	heavy metal-associated isoprenylated plant protein 26-like
2517	gene-LOC11141016	3.62324761586667	0.7067508191	0.8566390720	0.961108510	2.358009848979	lncRNA	XR_002705617.1	uncharacterized LOC111410166
2518	gene-LOC11141017	2.46109147383333	0.5	0.9626316258	0.976207875	2.299298280873	protein_codin	XM_023040411.1	squalene monooxygenase-like
2519	gene-LOC11141018	75.9194315653	118.74921351	-1.150043083	0.980078365	-0.64537686277	lncRNA	XR_002705619.1	uncharacterized LOC111410180
2520	gene-LOC11141019	0.5	2.1861148219	-1.035006510	0.984509101	-2.12836917806	protein_codin	XM_023040423.1	protein RMD5 homolog%2C transcript variant X1
2521	gene-LOC11141019	18.1352898007333	8.7691760177	0.7125127050	0.953197202	1.048286606160	protein_codin	XM_023040436.1	uncharacterized LOC111410194%2C transcript variant X2
2522	gene-LOC11141020	0.5	3.0393125553666	-1.002634183	0.979422332	-2.60374504566	protein_codin	XM_023040451.1	probable ubiquitin-like-specific protease 2A%2C transcript variant X2
2523	gene-LOC11141021	0.5	2.4599500583333	-0.805478126	0.958483519	-2.29862902640	lncRNA	XR_002705629.1	uncharacterized LOC111410213%2C transcript variant X3
2524	gene-LOC11141024	2.57415670776667	0.5	0.7724889366	0.956076955	2.364099883642	protein_codin	XM_023040503.1	xanthoxin dehydrogenase
2525	gene-LOC11141024	284.329144026867	355.35472884296	-0.772960791	0.954623028	-0.32169791362	protein_codin	XM_023040511.1	protein MON2 homolog%2C transcript variant X2
2526	gene-LOC11141026	4.3299918744	1.1329467819	0.7224626006	0.953304950	1.934284222821	protein_codin	XM_023040537.1	luc7-like protein 3%2C transcript variant X2
2527	gene-LOC11141029	3.18571065503333	0.5	0.7808221812	0.957178876	2.671615238770	lncRNA	XR_002705648.1	uncharacterized LOC111410294
2528	gene-LOC11141029	74.2164308633667	100.15546907203	-0.799232981	0.957697746	-0.43243067557	lncRNA	XR_002705650.1	uncharacterized LOC111410296
2529	gene-LOC11141031	3.0504194166	0.7331400576333	0.7021933227	0.952775943	2.056846879860	protein_codin	XM_023040584.1	transcription factor HBI1-like
2530	gene-LOC11141032	0.9255244066	3.899169016	-0.734934981	0.950318438	-2.07482375172	protein_codin	XM_023040594.1	protein kish-like%2C transcript variant X1
2531	gene-LOC11141034	15.0881574394	7.0380638207	0.7892841547	0.958235002	1.100166133675	protein_codin	XM_023040617.1	ascorbate transporter%2C chloroplastic%2C transcript variant X1
2532	gene-LOC11141036	3.10688397983333	0.7331400576333	0.8467665488	0.960746033	2.083307628576	protein_codin	XM_023040658.1	probable esterase PIR7A
2533	gene-LOC11141037	1.85599181786667	5.4916154005666	-0.737357687	0.950740165	-1.56504024104	protein_codin	XM_023040680.1	nascent polypeptide-associated complex subunit beta-like
2534	gene-LOC11141038	213.6218567685	182.45311826403	0.8316127346	0.960417260	0.227533456345	protein_codin	XM_023040701.1	non-specific lipid-transfer protein A-like
2535	gene-LOC11141039	138.8348689135	197.2260851798	-1.518668414	0.999999670	-0.50648042374	protein_codin	XM_023040703.1	RING-H2 finger protein ATL22-like
2536	gene-LOC11141041	0.5	2.4599500583333	-0.805478126	0.958483519	-2.29862902640	lncRNA	XR_002705678.1	uncharacterized LOC111410410
2537	gene-LOC11141043	11.5802462650667	4.0047868627333	0.7685421789	0.955575714	1.531868565294	protein_codin	XM_023040750.1	uncharacterized LOC111410430%2C transcript variant X2
2538	gene-LOC11141043	54.7165365179667	43.827792499466	0.6971712819	0.952397448	0.320130896876	protein_codin	XM_023040754.1	tRNA (guanine-N(7)-)-methyltransferase
2539	gene-LOC11141048	3.97482416473333	1.339697601	0.6877186842	0.951327340	1.568983651296	protein_codin	XM_023040841.1	protein OBERON 4-like
2540	gene-LOC11141051	2.03556706723333	0.5	0.7088504665	0.953093597	2.025430755730	protein_codin	XM_023040880.1	3-hydroxy-3-methylglutaryl-coenzyme A reductase-like
2541	gene-LOC11141052	2.08916849996667	0.5	0.7050067510	0.952935613	2.062928856341	protein_codin	XM_023040910.1	rac-like GTP-binding protein ARAC3
2542	gene-LOC11141053	5.87150883263333	2.1861148219	0.7114887940	0.953173490	1.425362109880	protein_codin	XM_023040937.1	SUMO-activating enzyme subunit 2-like
2543	gene-LOC11141055	1.96905705183333	0.5	0.6815674662	0.950366313	1.977504912765	protein_codin	XM_023040964.1	eukaryotic translation initiation factor 4B3-like
2544	gene-LOC11141056	2.5612481251	7.7305695922	-0.810582994	0.959021159	-1.59372769340	protein_codin	XM_023040978.1	protein BASIC PENTACYSTEINE4-like%2C transcript variant X4
2545	gene-LOC11141056	0.5	2.1928344418	-0.754119384	0.952977800	-2.13279689317	protein_codin	XM_023040980.1	uncharacterized LOC111410567
2546	gene-LOC11141058	2.0762599173	0.5	0.6996756982	0.952603165	2.053987059267	protein_codin	XM_023041006.1	protein SMG9-like

2547	gene-LOC11141060	1.95614846913333	5.8115094941666	-0.781792669	0.955492233	-1.57089706793	protein_codin	XM_023041079.1	plant UBX domain-containing protein 10-like%2C transcript variant X1
2548	gene-LOC11141062	0.5	2.1129218111	-0.740690064	0.951277924	-2.07923938117	protein_codin	XM_023041055.1	PI-PLC X domain-containing protein At5g67130-like
2549	gene-LOC11141063	2.0762599173	0.5	0.6996756982	0.952603165	2.053987059267	protein_codin	XM_023041076.1	RNA pseudouridine synthase 4%2C mitochondrial
2550	gene-LOC11141064	4.97292124993333	0.5	1.1684173413	0.983043184	3.314093585126	protein_codin	XR_002705724.1	pathogenesis-related homeodomain protein-like%2C transcript variant X3
2551	gene-LOC11141065	0.938432989266667	4.3261707866666	-0.797493958	0.957463917	-2.20476498525	protein_codin	XM_023041108.1	cysteine-rich and transmembrane domain-containing protein WIH2-like
2552	gene-LOC11141066	3.29936781186667	0.5	0.9977881427	0.972486662	2.722189617786	protein_codin	XM_023041115.1	mediator of RNA polymerase II transcription subunit 32-like%2C transcript variant X1
2553	gene-LOC11141067	5.53472044596667	1.55316804	0.9561729173	0.975987003	1.833296523644	protein_codin	XM_023041142.1	uncharacterized LOC111410678%2C transcript variant X1
2554	gene-LOC11141068	4.96287579773333	0.5	1.6625279099	0.972751841	3.311176349812	protein_codin	XM_023041147.1	L-type lectin-domain containing receptor kinase IX.1-like
2555	gene-LOC11141069	6.34162135573333	1.605946517	0.7795341888	0.957009074	1.981427892428	protein_codin	XM_023041161.1	testis-expressed protein 2-like
2556	gene-LOC11141069	30.4630606969333	57.190726678566	-1.079972040	0.975704746	-0.90872033672	protein_codin	XM_023041165.1	metacaspase-1-like%2C transcript variant X1
2557	gene-LOC11141071	36.5340995758667	57.001349839933	-1.150100370	0.980088595	-0.64175243298	protein_codin	XM_023041188.1	aminotransferase ALD1%2C chloroplastic
2558	gene-LOC11141072	3.52954525593333	0.5	0.9167642461	0.968253721	2.819482319680	protein_codin	XM_023041207.1	uncharacterized protein At1g10890-like%2C transcript variant X2
2559	gene-LOC11141073	1.95614846913333	0.5	0.6824614660	0.950524364	1.968015873129	protein_codin	XR_002705745.1	tRNA pseudouridine synthase A%2C transcript variant X2
2560	gene-LOC11141073	48.9083381680333	65.527382055933	-0.832142073	0.958838363	-0.42201744956	protein_codin	XR_002705747.1	metallothiol transferase FosB-like%2C transcript variant X2
2561	gene-LOC11141073	25.8657411043667	10.397954492133	1.1222706826	0.978101724	1.314742781963	protein_codin	XM_023041235.1	protein WHAT'S THIS FACTOR 1 homolog%2C transcript variant X5
2562	gene-LOC11141077	1.43046741126667	6.2912897418333	-1.011366408	0.982517021	-2.13686917546	protein_codin	XR_002705762.1	NAD-dependent malic enzyme 59 kDa isoform%2C mitochondrial%2C transcript variant X3
2563	gene-LOC11141079	3.15343919836667	0.5	0.9662992048	0.976157527	2.656926116795	protein_codin	XM_023041328.1	diphthamide biosynthesis protein 3-like
2564	gene-LOC11141080	6.0594179528	1.2861133161333	0.9164235551	0.968173577	2.236161459534	protein_codin	XM_023041339.1	polyadenylate-binding protein RBP47-like%2C transcript variant X2
2565	gene-LOC11141083	79.9471777172667	121.37620597413	-1.446812691	0.999434019	-0.60236662017	protein_codin	XM_023041398.1	type IV inositol polyphosphate 5-phosphatase 9-like
2566	gene-LOC11141089	1.75981795563333	8.6372907178666	-1.282681629	0.972734405	-2.29515265233	protein_codin	XM_023041491.1	eukaryotic translation initiation factor 4G-like%2C transcript variant X2
2567	gene-LOC11141091	2.97001726753333	0.7331400576333	0.7015369207	0.952730569	2.018310579386	protein_codin	XM_023041509.1	abscisic acid receptor PYL4-like
2568	gene-LOC11141100	0.5	2.5127285353666	-0.801158618	0.957947684	-2.32925481692	protein_codin	XM_023041584.1	transcription factor SCREAM2-like
2569	gene-LOC11141104	2.25224400576667	0.5	0.7304862915	0.953319521	2.171363135961	protein_codin	XM_023041615.1	uncharacterized LOC111411041
2570	gene-LOC11141107	0.5	3.9536200014333	-0.962842806	0.953886634	-2.98317421441	protein_codin	XM_023041650.1	peroxiredoxin-2
2571	gene-LOC11141115	3.71843792713333	0.5	1.3818228423	0.950048892	2.894696689057	protein_codin	XM_023041714.1	acyl-protein thioesterase 2-like
2572	gene-LOC11141116	0.5	2.8852793205666	-0.859212762	0.951607986	-2.52871099127	protein_codin	XM_023041723.1	F-box/LRR-repeat protein At4g29420
2573	gene-LOC11141124	2.7020976264	15.7834507016	-1.229392557	0.984052634	-2.54626094804	protein_codin	XM_023041801.1	subtilisin-like protease SBT6.1
2574	gene-LOC11141128	3.0532825471	0.5	1.1838123257	0.982546110	2.610361100988	protein_codin	XM_023041832.1	palmitoyl-acyl carrier protein thioesterase%2C chloroplastic-like
2575	gene-LOC11141131	42.6158310557	63.2912106442	-0.739291944	0.951059987	-0.57061569743	protein_codin	XM_023041855.1	MADS-box protein SVP-like
2576	gene-LOC11141139	3.45697257733333	0.5	0.8184713857	0.960190035	2.789509159370	protein_codin	XM_023041931.1	probable receptor-like serine/threonine-protein kinase At4g34500
2577	gene-LOC11141139	51.6031982468333	38.3911903214	0.7059021243	0.952975772	0.426685191586	protein_codin	XM_023041934.1	uncharacterized LOC111411399
2578	gene-LOC11141140	2.7537319571	0.5	0.6975232012	0.952426558	2.461388137184	protein_codin	XM_023041941.1	lysine histidine transporter-like 8
2579	gene-LOC11141147	12.4714835280333	27.7246303824	-0.966786634	0.956663679	-1.15253513771	protein_codin	XM_023041999.1	K(+) efflux antiporter 3%2C chloroplastic-like
2580	gene-LOC11141149	6.9121783474	2.7993919852	0.6936338356	0.952055485	1.304026927665	protein_codin	XM_023042018.1	uncharacterized LOC111411494
2581	gene-LOC11141155	44.5225288792	61.663739321933	-0.820561924	0.959527583	-0.46988683782	protein_codin	XM_023042072.1	uncharacterized WD repeat-containing protein C3H5.08c-like
2582	gene-LOC11141163	208.233064936067	152.78223273726	1.7711912973	0.989090630	0.446722389580	protein_codin	XM_023042150.1	calcium-transporting ATPase 5%2C plasma membrane-type-like
2583	gene-LOC11141164	6.8188676156	2.5126676426666	0.7592762542	0.954548931	1.440312321383	protein_codin	XM_023042167.1	PHD finger protein Alfin1-like
2584	gene-LOC11141165	71.9391366602667	40.2327552123	1.1553753582	0.983071769	0.838406306102	protein_codin	XM_023042189.1	protein CLT1%2C chloroplastic-like%2C transcript variant X2
2585	gene-LOC11141167	2.44818289113333	8.8516278574	-1.123934221	0.975861641	-1.85423145985	protein_codin	XM_023042228.1	60S ribosomal protein L31
2586	gene-LOC11141170	0.6358831613	3.1991987094666	-1.035017101	0.984508091	-2.33087699356	protein_codin	XM_023042266.1	delta(8)-fatty-acid desaturase-like
2587	gene-LOC11141170	1311.55608832353	1761.1204624114	-3.081325791	0.999999999	-0.42521408897	protein_codin	XM_023042270.1	ubiquitin-conjugating enzyme E2 28-like%2C transcript variant X2
2588	gene-LOC11141171	12.2260387710333	5.7059525401	0.7646756805	0.955114938	1.099417394971	protein_codin	XM_023042274.1	heavy metal-associated isoprenylated plant protein 39-like
2589	gene-LOC11141172	4.909274365	0.5	1.7254191230	0.964577484	3.295509796967	protein_codin	XM_023042292.1	probable glycerol-3-phosphate acyltransferase 8
2590	gene-LOC11141173	0.9255244066	4.9115688811	-0.753876054	0.952954229	-2.40784099157	protein_codin	XM_023042316.1	endoplasmic reticulum-Golgi intermediate compartment protein 3-like
2591	gene-LOC11141173	390.7988408196	469.8757460197	-1.098882732	0.974144366	-0.26585311281	protein_codin	XM_023042318.1	putative clathrin assembly protein At1g03050
2592	gene-LOC11141174	5.02938581313333	1.0801683049	0.8843557652	0.962885878	2.219126107969	protein_codin	XM_023042324.1	BRASSINOSTEROID INSENSITIVE 1-associated receptor kinase 1-like
2593	gene-LOC11141175	6.6557921098	2.2133098683	0.6853109445	0.950980607	1.588404926563	protein_codin	XR_002705890.1	uncharacterized LOC111411755%2C transcript variant X3
2594	gene-LOC11141177	2.03556706723333	0.5	0.7088504665	0.953093597	2.025430755730	protein_codin	XM_023042372.1	glucan endo-1%2C3-beta-glucosidase 3-like%2C transcript variant X2
2595	gene-LOC11141178	5.93801884806667	1.8458061945333	0.7095479802	0.953116356	1.685730591412	protein_codin	XM_023042384.1	serine hydroxymethyltransferase 7-like

2596	gene-LOC11141178	8.4656203373	3.0656409012	0.7755462809	0.956479554	1.465427074227	protein_codin	XR_002705895.1	uncharacterized LOC111411789%2C transcript variant X2
2597	gene-LOC11141179	22.1743527732333	45.2191896127	-1.110269142	0.974482917	-1.02804314168	lncRNA	XR_002705896.1	uncharacterized LOC111411790
2598	gene-LOC11141182	20.2198835888	36.687334063466	-0.793649930	0.956949921	-0.85950738164	lncRNA	XR_002705908.1	uncharacterized LOC111411822%2C transcript variant X5
2599	gene-LOC11141183	4.02744204643333	0.5	1.2336071090	0.985023200	3.009863829173	protein_codin	XM_023042471.1	transcription factor UNE12-like%2C transcript variant X2
2600	gene-LOC11141185	3.0393904134	11.810905996666	-1.147987877	0.979715142	-1.95826572811	protein_codin	XM_023042487.1	transcription factor bHLH3-like%2C transcript variant X1
2601	gene-LOC11141188	0.798567038966667	3.4127300411333	-0.835574781	0.958330863	-2.09544086598	protein_codin	XM_023042539.1	photosystem II 5 kDa protein%2C chloroplastic-like
2602	gene-LOC11141190	9.04391927686667	4.3194511668	0.7077986401	0.953057499	1.066100101157	protein_codin	XM_023042562.1	uncharacterized LOC111411904
2603	gene-LOC11141191	22.6151444927	14.236880496033	0.8119054295	0.959986007	0.667656147835	protein_codin	XM_023042580.1	hydroxymethylglutaryl-CoA lyase%2C mitochondrial-like
2604	gene-LOC11141192	3.7119836358	1.1329467819	0.6983515715	0.952495393	1.712110255596	protein_codin	XM_023042602.1	THO complex subunit 7A-like%2C transcript variant X1
2605	gene-LOC11141193	0.5	2.8257812237	-1.119397939	0.975307321	-2.49864977434	protein_codin	XM_023042613.1	F-box/WD-40 repeat-containing protein At5g21040-like
2606	gene-LOC11141195	14.2964363199	7.1708767138333	0.7303855980	0.953319276	0.995434151265	protein_codin	XM_023042659.1	U-box domain-containing protein 43-like%2C transcript variant X1
2607	gene-LOC11141196	0.629428869966667	4.0252013965666	-1.125492704	0.976068240	-2.67694571145	protein_codin	XM_023042675.1	protein trichome birefringence-like 12
2608	gene-LOC11141196	7.37454892256667	1.8730012409	0.7761618586	0.956561460	1.977202951651	protein_codin	XM_023042676.1	peptide methionine sulfoxide reductase B5-like
2609	gene-LOC11141196	15.7759304520333	26.021397254233	-0.821935196	0.959534479	-0.72197333486	protein_codin	XM_023042677.1	malate dehydrogenase%2C glyoxysomal-like
2610	gene-LOC11141197	0.5	4.4250081208666	-1.300597087	0.970778727	-3.14568010286	protein_codin	XM_023042684.1	uncharacterized protein At3g49720-like
2611	gene-LOC11141198	6.59573638573333	15.624187677266	-0.855872294	0.952803713	-1.24417553974	protein_codin	XM_023042698.1	uncharacterized LOC111411984%2C transcript variant X2
2612	gene-LOC11141198	3308.5136125467	4425.6476788284	-1.954766806	0.999983369	-0.41970538888	protein_codin	XM_023042699.1	transcription factor bHLH113-like
2613	gene-LOC11141198	0.629428869966667	2.1861148219	-0.782603758	0.955581572	-1.79625392081	protein_codin	XR_002705929.1	DNA repair protein REV1%2C transcript variant X6
2614	gene-LOC11141200	8.59036172323333	2.1393110495666	0.6874083366	0.951282296	2.005572620301	protein_codin	XM_023042717.1	U-box domain-containing protein 26-like
2615	gene-LOC11141204	0.5	5.7111823296666	-1.075767293	0.976420096	-3.51378944341	protein_codin	XM_023042778.1	putative ribosomal-protein-alanine acetyltransferase
2616	gene-LOC11141205	19.2005440501333	7.6241459375666	1.0146810032	0.970178937	1.332499550526	protein_codin	XM_023042798.1	pentatricopeptide repeat-containing protein At2g29760%2C chloroplastic-like%2C transcript variant X1
2617	gene-LOC11141206	2.97001726753333	0.5	0.9081263456	0.966349227	2.570471318807	protein_codin	XM_023042813.1	calcium-transporting ATPase 5%2C plasma membrane-type-like
2618	gene-LOC11141207	11.4653014516667	6.7718149046666	0.7049285025	0.952931170	0.759659842084	protein_codin	XM_023042828.1	uncharacterized LOC111412077
2619	gene-LOC11141209	2.00974990186667	0.5	0.7043739485	0.952899766	2.007015980117	protein_codin	XM_023042866.1	tRNA-specific adenosine deaminase 2-like%2C transcript variant X2
2620	gene-LOC11141211	1.36395739586667	4.0523964429	-0.740862879	0.951304133	-1.57097673730	protein_codin	XM_023042888.1	bZIP transcription factor 11-like
2621	gene-LOC11141212	5.68064905946667	0.5	1.3677520228	0.955841611	3.506055778563	protein_codin	XM_023042903.1	rop guanine nucleotide exchange factor 1
2622	gene-LOC11141214	3.941569157	0.5	1.2334808834	0.985013379	2.978770087637	protein_codin	XM_023042922.1	probable polyamine transporter At1g31830%2C transcript variant X3
2623	gene-LOC11141214	2.66845099056667	0.5	0.8904698014	0.963535251	2.416002514674	protein_codin	XM_023042924.1	dnaJ homolog subfamily C member 17-like%2C transcript variant X1
2624	gene-LOC11141216	432.5630879593	702.50015722476	-1.573176094	0.999999999	-0.69958798761	protein_codin	XM_023042950.1	uncharacterized LOC111412160
2625	gene-LOC11141216	5.3180435074	12.0499598663	-0.852534737	0.953942027	-1.18006085623	protein_codin	XM_023042957.1	CBL-interacting protein kinase 5-like
2626	gene-LOC11141223	3.45598902633333	0.6531665342333	0.8839842876	0.962850002	2.403575856871	protein_codin	XM_023043070.1	uncharacterized LOC111412230
2627	gene-LOC11141224	0.5	2.3000030115333	-0.746106397	0.952051421	-2.20163575017	protein_codin	XM_023043096.1	CBL-interacting serine/threonine-protein kinase 23-like
2628	gene-LOC11141224	7.77913498126667	2.6131165925666	0.8114298345	0.959966691	1.573838243796	protein_codin	XM_023043099.1	auxin response factor 6
2629	gene-LOC11141225	3.9858531679	0.7067508191	0.8642917795	0.961472910	2.495615009502	protein_codin	XM_023043100.1	hydroxyproline O-galactosyltransferase GALT2-like
2630	gene-LOC11141225	2.7020976264	0.5	0.6901994521	0.951655310	2.434079799970	protein_codin	XM_023043102.1	tyrosine/DOPA decarboxylase 1-like
2631	gene-LOC11141225	11.6606484141667	22.848709568666	-0.977879570	0.964667083	-0.97046467342	protein_codin	XM_023043107.1	dnaJ protein homolog
2632	gene-LOC11141227	10.3002821791333	25.192171335633	-1.221169765	0.984862741	-1.29029161361	protein_codin	XM_023043135.1	secoisolariciresinol dehydrogenase-like
2633	gene-LOC11141227	5.85663314793333	13.8347172409	-0.823368555	0.959500655	-1.24014972410	protein_codin	XM_023043136.1	secoisolariciresinol dehydrogenase-like
2634	gene-LOC11141228	6.63224615203333	2.1928344418	0.7182144067	0.953281872	1.596700659456	protein_codin	XM_023043143.1	fasciclin-like arabinogalactan protein 17
2635	gene-LOC11141230	19.3410019233	10.450854754533	0.8955751547	0.964193524	0.888041590507	protein_codin	XM_023043182.1	feruloyl CoA ortho-hydroxylase 1-like
2636	gene-LOC11141231	26.9022275353667	42.626015795666	-0.818651626	0.959504043	-0.66400858003	protein_codin	XM_023043185.1	BTB/POZ and MATH domain-containing protein 4
2637	gene-LOC11141231	0.629428869966667	3.4721672453333	-0.804788108	0.958405074	-2.46372118310	protein_codin	XM_023043191.1	non-specific lipid-transfer protein-like protein At2g13820
2638	gene-LOC11141234	92.9413364242	118.18208568273	-1.262552532	0.977389882	-0.34661907005	protein_codin	XM_023043231.1	ADP-ribosylation factor GTPase-activating protein AGD12-like%2C transcript variant X2
2639	gene-LOC11141234	354.233314910267	411.86874724116	-1.388299649	0.988858005	-0.21748475715	protein_codin	XM_023043239.1	uncharacterized LOC111412346%2C transcript variant X4
2640	gene-LOC11141234	0.5	2.8069783056333	-0.756836526	0.953241779	-2.48901791369	protein_codin	XM_023043242.1	cell division control protein 48 homolog C-like
2641	gene-LOC11141236	1.06786185923333	4.5924197026333	-0.796313909	0.957307059	-2.10452946722	lncRNA	XR_002706051.1	uncharacterized LOC111412361
2642	gene-LOC11141238	4.38457685813333	1.0930574109666	0.8399045351	0.960568725	2.004068441295	protein_codin	XM_023043289.1	pectinesterase-like
2643	gene-LOC11141239	0.5	4.6460039875666	-1.215715224	0.985234777	-3.21599039238	protein_codin	XM_023043304.1	peter Pan-like protein%2C transcript variant X2
2644	gene-LOC11141240	3.65838220303333	0.5	1.2189499540	0.983729315	2.871205805982	protein_codin	XM_023043331.1	uncharacterized LOC111412407

2645	gene-LOC11141242	3.72587576946667	0.5	1.1296222738	0.9798700494	2.897579571820	protein_codin	XR_002706067.1	plasma membrane ATPase 2-like%2C transcript variant X10
2646	gene-LOC11141242	329.240874279333	402.21347689586	-1.2230734486	0.9847014446	-0.2888179693860	lncRNA	XR_002706069.1	uncharacterized LOC111412428
2647	gene-LOC11141244	7.22990796563333	1.8662816210333	0.8552583084	0.9610497023	1.953810577625	protein_codin	XM_023043374.1	uncharacterized LOC111412440
2648	gene-LOC11141244	4865.71919950087	5336.7081056144	-0.8594020479	0.9515392321	-0.133297039637	protein_codin	XM_023043381.1	uncharacterized LOC111412446%2C transcript variant X2
2649	gene-LOC11141246	3.65838220303333	0.5	1.2189499540	0.9837293155	2.871205805982	protein_codin	XM_023043392.1	uncharacterized LOC111412460
2650	gene-LOC11141248	3.74563027163333	15.9424092624	-1.3818374563	0.9866288867	-2.089589265625	protein_codin	XM_023043418.1	zinc finger A20 and AN1 domain-containing stress-associated protein 5-like
2651	gene-LOC11141256	2.97001726753333	0.7067508191	0.7223405095	0.9533047200	2.071197763603	protein_codin	XM_023043503.1	uncharacterized LOC111412568
2652	gene-LOC11141257	2.0762599173	0.5	0.6996756982	0.9526031653	2.053987059267	protein_codin	XM_023043509.1	probable protein S-acyltransferase 23
2653	gene-LOC11141265	4.2699361503	0.8863065918666	0.8666135479	0.9615979574	2.268336748511	protein_codin	XM_023043584.1	protein NRT1/ PTR FAMILY 4.5-like
2654	gene-LOC11141265	0.5	2.8325008435666	-0.8411716718	0.9572015853	-2.502076385704	protein_codin	XM_023043586.1	subtilisin-like protease SBT5.6
2655	gene-LOC11141269	2.1520873545	6.9045669051	-1.0637899429	0.9789466253	-1.681814284104	protein_codin	XM_023043618.1	uncharacterized LOC111412696
2656	gene-LOC11141275	4.27091970136667	0.9729997351	0.8582046172	0.9611761435	2.134035456419	protein_codin	XM_023043673.1	delta(14)-sterol reductase
2657	gene-LOC11141276	1.43046741126667	4.9311167143	-0.7470715558	0.9521751356	-1.785427769570	protein_codin	XM_023043678.1	serine/threonine-protein kinase CTR1-like
2658	gene-LOC11141287	0.5	2.6734204973	-0.7967574353	0.9573659775	-2.418686774228	protein_codin	XM_023043744.1	40S ribosomal protein S28-2
2659	gene-LOC11141295	2.2586982971	9.1714001655666	-1.0724486053	0.9770553980	-2.021650424486	protein_codin	XM_023043812.1	heme oxygenase 1%2C chloroplastic-like
2660	gene-LOC11141299	2.40561046163333	0.5	0.7495359276	0.9538054195	2.266403047099	protein_codin	XM_023043865.1	hypersensitive-induced response protein 1-like%2C transcript variant X1
2661	gene-LOC11141300	2.0762599173	0.5	0.6996756982	0.9526031653	2.053987059267	protein_codin	XM_023043879.1	protein kinase and PP2C-like domain-containing protein
2662	gene-OleuseuP_p04	76.3384075170333	52.071783848666	0.7905920022	0.9583851089	0.551907260356	protein_codin	YP_004376443.1	ribosomal protein S18
2663	gene-OleuseuP_p05	32.5198702176333	52.924236666933	-0.8180927358	0.9594839712	-0.702607056381	protein_codin	YP_004376450.1	cytochrome b6
2664	gene-OleuseuP_p05	8.52705124053333	18.610037733166	-0.7734824013	0.9546692747	-1.125962149401	protein_codin	YP_004376457.1	ribosomal protein L16
2665	gene-OleuseuP_p06	17.3368102843333	9.5976570211	0.8432320769	0.9606479031	0.853084325124	protein_codin	YP_004376467.1	Ycf1 protein
2666	gene-OleuseuP_t015	22.7739816888667	11.438294318733	0.9171968750	0.9683557060	0.993514613984	tRNA		
2667	gene-OleuseuP_t018	6.5892820944	1.9801698106666	0.7677816268	0.9554817736	1.734497136643	tRNA		
