# Supplementary material for: Bacillus G7 improves adaptation to salt stress in Olea europaea L. plantlets, enhancing water use efficiency and preventing oxidative stress
Source: Sci Rep. 2023 Dec 15;13:22507. doi: 10.1038/s41598-023-49533-z (PMC10728083; doi:10.1038/s41598-023-49533-z)
Supplement: Supplementary file 1 — Supplementary Information. [file 41598_2023_49533_MOESM1_ESM.zip › suplementary material/Figure S1.docx]

**Figure S1.** Differential gene expression of ion transporters SOS1 (*OeSOS1*), NHX (*OeNHX*), ABA receptor PYL-8 (*OePYL*-8), and PR genes PR5 (*OePR5*) and PR10 (*OePR10)*. Control expression values are indicated by the red line. Control expression values are set as 1, and the relative expression of the genes in G7-treated plants versus controls is represented.
