# Supplementary material for: Bacillus G7 improves adaptation to salt stress in Olea europaea L. plantlets, enhancing water use efficiency and preventing oxidative stress
Source: Sci Rep. 2023 Dec 15;13:22507. doi: 10.1038/s41598-023-49533-z (PMC10728083; doi:10.1038/s41598-023-49533-z)
Supplement: Supplementary file 1 — Supplementary Information. [file 41598_2023_49533_MOESM1_ESM.zip › suplementary material/Table S1 and S3.docx]

The activity of antioxidant enzymes Superoxyde dismutase (SOD) and Ascorbate peroxidase (APX) was not affected by G7 (table S1)

**Table S1.** Superoxide dismutase activity (% inhibition mg ^−1^ protein) and Ascorbate peroxidase activity (µmol mg protein^−1^ min^−1^), measured in olive tree leaves treated with G7 and relative change (%) of inoculated vs. control. For each treatment and parameter average value ±SE value is presented (*n* = 6). Asterisks (*) represent significant differences with the control according to the T student test (*p* < 0.05).

| **Parameters** | **Control AQ** | **G7 AQ** | **% G7 vs Control** |
| --- | --- | --- | --- |
| SOD | 97.71 ± 1.84 | 98.97 ± 3.99 | 1% |
| APX | 0.05 ± 0.001 | 0.05 ± 0.003 | 0% |

**Table S3.** Primers designed for RT-qPCR expression analysis in Olea europaea L.

| **Identificador** | **Nombre del gen y**  **referencia** | **Forward Primer** | **Reverse Primer** |
| --- | --- | --- | --- |
| *OeSOS1* | SOS1  (XM_023036083.1) | 5’TTGACATCCGAAACGAGGCA | 5’TCCCGACGAATATCACTGCG |
| *OeNHX* | NHX  (XM_022986695) | 5’TGTGGGATTGTGATGTCGCA | 5’TGTTGCAAAGGTGTGCTTGG |
| *OePYL*-8 | PYL-8  (XM_023040693.1) | 5’ACGACCAGCACTGAGAGGTT | 5’TTAGCCTGTGATCACCACCA |
| *OePR5* | Taumatina o PR5  (XM_023041217.1) | 5’CAGGTGGCTGTGGAAGTACA | 5’TGAACCCATCAACATGGCTA |
| *OePR10* | PR10  (Gómez -Lama, 2015) | 5’GATGTGTGGAGAGGCTTT | 5’CGTCATTTTTCTTCCTAGGT |
| *OeGAPDH2* | GAPDH2  (Nonis et al., 2012) | 5’ CCTTCCGTGTGCCTACTGTT | 5 ‘GATGGCTGCCTTGATTTCAT |
